# Neutrophil-to-high-density-lipoprotein-cholesterol ratio and mortality among patients with hepatocellular carcinoma

**DOI:** 10.3389/fnut.2023.1127913

**Published:** 2023-05-05

**Authors:** Ke Shi, Jie Hou, Qun Zhang, Yufei Bi, Xuanwei Zeng, Xianbo Wang

**Affiliations:** ^1^Center of Integrative Medicine, Beijing Ditan Hospital, Capital Medical University, Beijing, China; ^2^Department of Spleen and Stomach Diseases, Hengshui Hospital of Traditional Chinese Medicine, Hebei, China

**Keywords:** hepatocellular carcinoma, dyslipidemia, high-density lipoprotein cholesterol, inflammation, prognosis

## Abstract

**Background:**

Inflammatory responses and lipid metabolism disorders contribute to the development and prognosis of hepatocellular carcinoma (HCC). This study aimed to investigate the prognostic value of lipid-related inflammatory parameters in patients with HCC.

**Methods:**

From January 2010 to June 2017, we enrolled 1,639 patients with HCC at Beijing Ditan Hospital. Multivariate Cox regression analysis and area under the receiver operating characteristic (AUC) analysis were used to evaluate and compare the predictability and reliability of high-density lipoprotein cholesterol (HDL-C), neutrophil-to-HDL-C ratio (NHR), monocyte-to-HDL-C ratio (MHR), and lymphocyte-to-HDL-C ratio (LHR) values. A restricted cubic spline was used to explore the association between the NHR and 3-year mortality in patients with HCC. Differences in survival rates were estimated using the Kaplan–Meier method and compared using the log-rank test. The results were validated in an internal cohort between July 2017 and October 2019 (*n* = 373).

**Results:**

After adjusting for confounding variables, NHR was independently associated with 3-year mortality, both as a continuous and categorical variable (both *p* < 0.05). The correlation between the mortality and the MHR and LHR was not statistically significant. The NHR showed a suitable prognostic value (AUC at 3 years: 0.740), similar to that of the Model for End-stage Liver Disease (MELD) (AUC at 3 years: 0.761). In the validation cohort, the AUC of the NHR was 0.734 at 3 years. The optimal cut-off values of NHR and MELD were 3.5 and 9, respectively. The 3-year survival rates in the low- (NHR < 3.5 and MELD <9) and high-risk (NHR ≥ 3.5 and MELD ≥9) groups were 81.8 and 19.4%, respectively, in the training cohort, and 84.6 and 27.5%, respectively, in the validation cohort.

**Conclusion:**

Baseline NHR is a promising prognostic parameter for mortality in patients with HCC and patients with NHR ≥ 3.5 and MELD ≥9 have a high mortality rate.

## Introduction

Hepatitis B virus (HBV) is a global public health problem and a major cause of hepatocellular carcinoma (HCC), causing approximately 200 million infected (1, 2). HCC is one of the most commonly occurring cancer and a common cause of cancer-associated mortality, accounting for 782,000 deaths worldwide every year (3). Despite substantial improvements in the treatment of HCC, the prognosis of HCC remains poor owing to a high recurrence rate (4). Given the increasing incidence and high mortality rate of HCC, early identification of the mortality risk of HCC is important to improve therapeutic intervention and long-term prognosis.

The inflammatory response plays an important role in the development and progression of HCC (5). Previous studies have shown that the neutrophil-to-lymphocyte ratio has a good prognostic value for HCC (6, 7). Recent studies have suggested that high-density lipoprotein cholesterol (HDL-C) exerts anti-inflammatory, anti-oxidation, and anti-apoptotic functions (8, 9). Decreased HDL-C levels were proven to be correlated with poor prognosis in several diseases (10–12). The neutrophil-to-HDL-C ratio (NHR), monocyte-to-HDL-C ratio (MHR), and lymphocyte-to-HDL-C ratio (LHR) have emerged as prognostic markers in cardiovascular events, diabetes, nerve diseases, and metabolic syndrome (13–17). However, research on the prognostic potential of these markers for mortality in patients with HCC is limited. Therefore, clarification on the reliability of these markers as prognostic biomarkers of HCC is necessary. In addition, the lack of consistent cutoff points for prognostic markers makes it difficult to distinguish between low-and high-risk mortality.

Patients with type 2 diabetes mellitus have an increased mortality risk due to HCC, in which inflammation and lipid metabolism disorders play important roles (18). Previous reports suggested that patients with diabetes have impaired HDL function and decreased HDL levels (19, 20). Therefore, the utility of lipid-related inflammatory markers in patients with diabetes is worth exploring.

Accordingly, we aimed to evaluate the association between the NHR, MHR, and LHR and mortality in patients with HCC using Cox regression analyses, identify high-risk populations using the Kaplan–Meier method, and conduct an early intervention to reduce mortality.

## Materials and methods

### Study population

**Figure 1:**
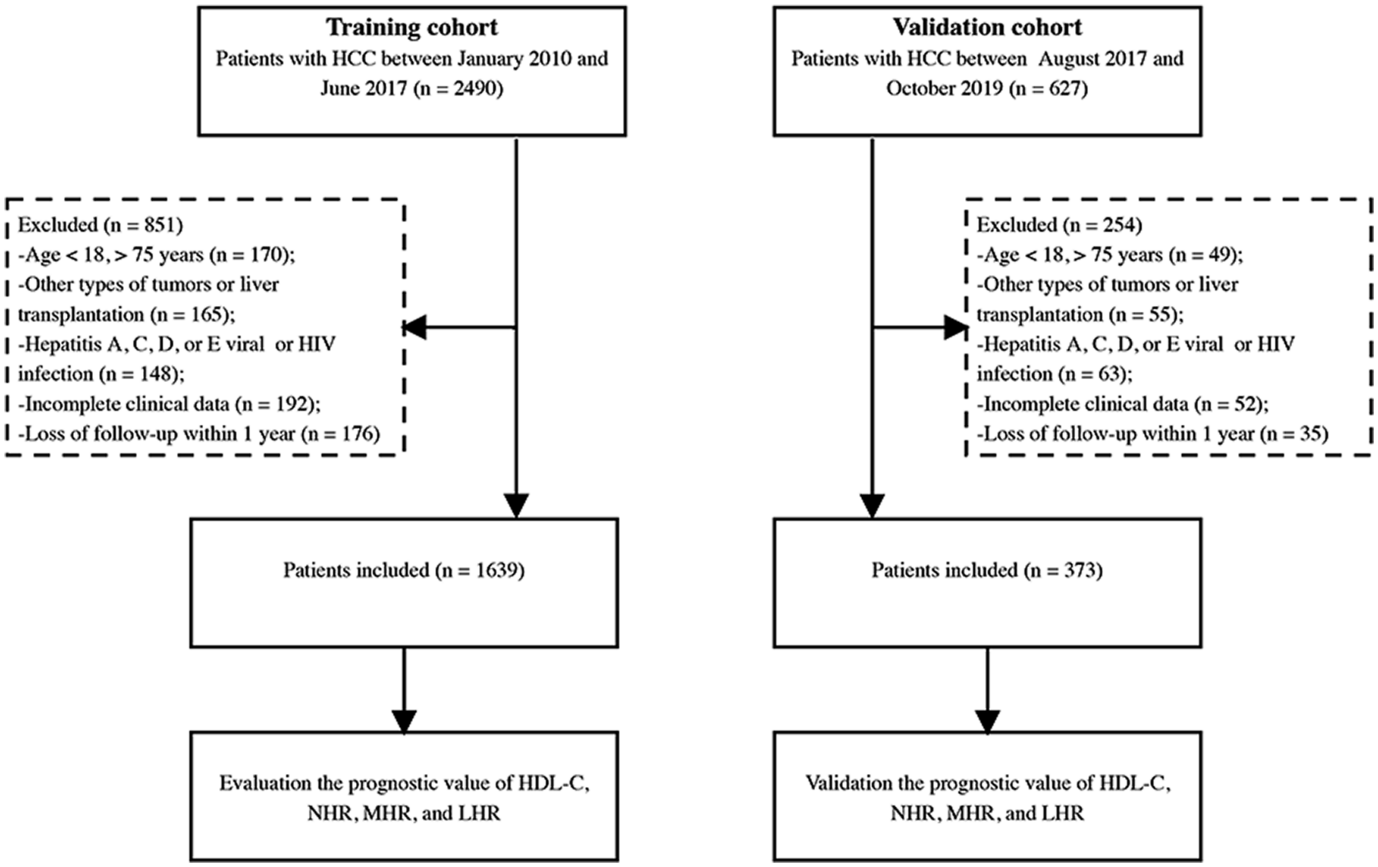
Study flow diagram. HCC, hepatocellular carcinoma; HIV, human immunodeficiency virus; HDL-C, high-density lipoprotein cholesterol; NHR, neutrophil to HDL-C ratio; MHR, monocyte to HDL-C ratio; LHR, lymphocyte to HDL-C ratio.

We screened 2,490 patients diagnosed with HCC between January 2010 and June 2017 at Beijing Ditan Hospital, Capital Medical University. Patients aged between 18 and 75 years diagnosed with HBV-related HCC were recruited for this study. The exclusion criteria were as follows: (1) age <18 or >75 years, (2) presence of other types of tumors or liver transplantation, (3) other viral infections or human immunodeficiency virus infection, (4) incomplete clinical data, and (5) lost of follow-up within 1 year. As per these criteria, 1,639 patients were finally enrolled in the study. We also included 373 patients as an internal validation cohort between July 2017 and October 2019 (Figure 1). This study followed the ethical principles of the Declaration of Helsinki and approval was obtained from the Ethical Review Committee of the Beijing Ditan Hospital.

### Clinical definition and follow-up

Chronic hepatitis B was defined as HBsAg positivity for >6 months (21). The diagnosis of cirrhosis was based on evidence from liver biopsy, endoscopy, ultrasound, or elastography, and/or signs of complications associated with portal hypertension (22). HCC diagnosis was as per the criteria of the Asia-Pacific clinical guidelines (23). Every 3 months, routine laboratory tests [including routine blood examination, liver, renal, coagulation function tests, HBV DNA, and alpha-fetoprotein (AFP)] and radiological examination, such as computed tomography (CT), magnetic resonance imaging (MRI), or ultrasound were performed. The outcome was the occurrence of mortality within 3 years or at the end of the 3 years follow-up period.

### Data collection

Baseline demographic characteristics and laboratory data, including age, sex, hypertension, diabetes, smoking history, alcohol consumption history, complications, liver function, renal function, coagulation function, serum lipid level, and AFP level, were collected from electronic medical records at enrollment. In addition, tumor characteristics, such as tumor number, size, vascular invasion, and tumor metastasis, were recorded based on the imaging data at baseline. NHR was calculated as the neutrophil count divided by the HDL-C value, while MHR as the monocyte count divided by the HDL-C value, and LHR as the lymphocyte count divided by the HDL-C value. The model for end-stage liver disease (MELD) was used to estimate the severity of the liver disease (24).

### Statistical analysis

SPSS (version 25.0; SPSS, Inc., Chicago, IL, United States) and R (version 3.6.3; The R Foundation, Vienna, Austria) software were used for the statistical analyses. Continuous variables were reported as mean ± standard deviation or median with interquartile range (IQR), while categorical variables were reported as frequency (percentage). Continuous variables were compared using Student’s *t*-test or the Mann–Whitney test; the chi–squared test or Fisher’s exact test was used for two groups, as appropriate. Univariate and multivariate Cox regression analyses were used to assess the association between the HDL-C, NHR, MHR, LHR (continuous and tertile), and mortality. Results were considered statistically significant at *p* -value <0.05.

The predictive value of lipid-related inflammatory markers was evaluated using the area under the receiver operating characteristic (ROC) curve (AUC). The prognostic power of these indicators was compared with that of the MELD score at 1, 2, and 3 years using the Delong test (25). The association between the NHR and 3-year mortality were evaluated on a continuous scale using a restricted cubic spline (RCS) with four knots at the 5th, 35th, 65th, and 95th percentiles and to test for nonlinearity (26). The optimal cut-off values were determined for the NHR and MELD scores for mortality using the X-tile software (Yale University School of Medicine, New Haven, CT, United States). Differences in survival rates among the groups were analyzed using Kaplan–Meier curves and compared using log-rank tests.

## Results

### Baseline characteristics

A total of 1,639 and 373 patients in the training and validation cohorts, respectively, were included in the analysis. Baseline characteristics and laboratory data of the patients are shown in Table 1. The median age of the training cohort was 57.0 years (IQR, 50.0–63.0), with male predominance (*n* = 1,563, 77.6%). Of those patients, 181 (9.0%) patients underwent liver resection, 1,358 (67.5%) underwent minimally invasive therapy, whereas 473 (23.5%) received palliative therapy. Of the 1,639 patients in the training cohort, 1,563 patients (77.6%) were male and 1,306 patients (79.6%) were diagnosed with cirrhosis. During the 3-year follow-up period, 666 patients (40.6%) and 138 patients (37.0%) died in the training and validation cohorts, respectively. Overall, the patients in the two cohorts were similar when their baseline characteristics were considered.

**Table 1:** Baseline demographics and clinical characteristics of patients with HCC in the training and validation cohorts.

	**Total (*n* = 2012)**	**Training cohort (*n* = 1,639)**	**Validation cohort (*n* = 373)**	*p* **-value**
**Patients background**				
Age (year)	57.0 (50.0, 63.0)	56.0 (50.0, 63.0)	57.0 (50.0, 62.0)	0.402
Sex (male)	1,563 (77.6)	1,289 (78.6)	274 (73.4)	0.292
Family history of HCC	198 (9.8)	165 (10.1)	33 (8.8)	0.511
Cirrhosis	1,661 (82.6)	1,306 (79.6)	301 (80.6)	0.659
Smoking	895 (44.5)	747 (45.6)	148 (39.7)	0.054
Alcohol consumption	876 (43.5)	735 (44.8)	141 (37.8)	0.052
Hypertension	532 (26.4)	435 (26.5)	97 (26.0)	0.895
Diabetes	455 (22.6)	374 (22.8)	81 (21.7)	0.719
**Laboratory parameters**				
HBeAg (positive)	620 (30.8)	504 (30.7)	116 (31.1)	0.369
MELD score	8.8 (7.0, 11.9)	8.8 (7.0, 11.9)	8.7 (7.0, 10.9)	0.135
ALT (U/L)	32.4 (21.6, 53.8)	32.0 (21.2, 52.4)	34.7 (22.7, 58.6)	0.091
AST (U/L)	39.1 (26.6, 71.1)	39.1 (26.5, 70.4)	38.9 (27.7, 75.7)	0.098
TBIL (μmol/L)	19.5 (12.8, 33.1)	19.8 (12.8, 34.3)	18.7 (13.0, 32.4)	0.697
ALB (g/L)	36.1 ± 6.8	35.6 ± 6.7	36.8 ± 6.9	0.136
γ-GGT (U/L)	58.4 (27.0, 129.6)	58.3 (27.6, 129.1)	61.3 (28.4, 132.4)	0.486
PLT (×10^9^/L)	97.9 (63.3, 148.0)	96.2 (62.4, 147.3)	104.7 (64.5, 151.3)	0.169
INR	1.1 (1.0, 1.3)	1.1 (1.0, 1.3)	1.1 (1.0, 1.2)	0.190
Cr (μmol/L)	67.0 (58.0, 78.2)	67.0 (58.0, 78.0)	66.6 (57.5, 78.6)	0.647
AFP (ng/mL) (≥400)	498 (24.7)	396 (24.1)	102 (27.3)	0.198
Neutrophils (×10^9^/L)	2.7 (1.8, 4.0)	2.6 (1.8, 4.0)	2.8 (1.8, 4.1)	0.741
Lymphocytes (×10^9^/L)	1.1 (0.8, 1.6)	1.1 (0.8, 1.6)	1.1 (0.7, 1.6)	0.645
Monocytes (×10^9^/L)	0.4 (0.3, 0.5)	0.4 (0.3, 0.5)	0.4 (0.3, 0.5)	0.292
TC (mmol/L)	3.7 (3.1, 4.3)	3.6 (3.0, 4.3)	3.7 (3.2, 4.4)	0.798
TG (mmol/L)	0.8 (0.6, 1.1)	0.8 (0.6, 1.1)	0.9 (0.6, 1.1)	0.536
HDL-C (mmol/L)	1.0 (0.7, 1.2)	1.0 (0.6, 1.2)	1.0 (0.8, 1.3)	0.526
LDL-C (mmol/L)	2.0 (1.7, 2.6)	2.0 (1.6, 2.6)	1.9 (1.6, 2.7)	0.199
NHR	2.7 (1.6, 4.8)	2.8 (1.6, 5.0)	2.5 (1.6, 4.4)	0.276
MHR	0.4 (0.2, 0.6)	0.4 (0.3, 0.6)	0.3 (0.2, 0.5)	0.301
LHR	1.2 (0.8, 1.8)	1.2 (0.8, 1.6)	1.1 (0.7, 1.8)	0.321
**Child Pugh class**				0.426
A	1,077 (53.5)	882 (53.8)	195 (52.3)	
B	558 (27.7)	456 (27.8)	102 (27.3)	
C	377 (18.8)	301 (18.4)	76 (20.4)	
**Tumor–related indicators**				
Tumor multiplicity (multiple)	937 (46.5)	753 (45.9)	184 (49.3)	0.237
Tumor size, cm (≥5)	670 (33.3)	555 (33.9)	115 (30.8)	0.291
**BCLC stage**				0.517
0–A	687 (34.1)	564 (34.4)	123 (33.0)	
B	669 (33.3)	539 (32.9)	130 (34.8)	
C	364 (18.1)	292 (17.8)	72 (19.3)	
D	292 (14.5)	244 (14.9)	48 (12.9)	
**Types of treatment**				0.108
Resection	181 (9.0)	151 (9.2)	30 (8.0)	
Minimally invasive	1,358 (67.5)	1,125 (68.7)	233 (62.5)	
Palliative	473 (23.5)	363 (22.1)	110 (29.5)	

Furthermore, we compared the survival and death characteristics of patients in the training cohort (Table 2). Patients who died were older, had a higher proportion of diabetes, tumor size ≥5 cm, AFP ≥ 400 ng/mL, higher total bilirubin (TBIL), γ-glutamyl transferase (GGT), creatinine (Cr), and international prothrombin ratio (INR), and lower albumin levels (all *p* < 0.001) than those who survived. Regarding inflammation and lipid-related markers, dead patients had higher levels of neutrophils, monocytes, NHR, MHR, and LHR, and lower levels of lymphocytes, total cholesterol (TC), and HDL-C compared with the patients who survived.

**Table 2:** Baseline characteristics of survival and death patients in the training cohort.

	**Survived (*n* = 973)**	**Death (*n* = 666)**	*p*-**value**
**Patients background**			
Age (year)	56.0 (50.0, 62.0)	57.0 (50.0, 64.0)	0.054
Sex (male)	754 (77.5)	535 (80.3)	0.169
Family history of HCC	106 (10.9)	55 (8.8)	0.236
Cirrhosis	775 (79.6)	531 (79.7)	0.182
Smoking	432 (44.4)	315 (47.3)	0.251
Alcohol consumption	415 (42.6)	320 (48.0)	0.027
Hypertension	244 (25.1)	191 (28.7)	0.104
Diabetes	195 (20.0)	179 (26.9)	0.001
**Laboratory parameters**			
HBeAg (positive)	308 (31.6)	196 (29.4)	0.241
MELD score	7.7 (6.7, 9.7)	11.3 (8.6, 15.7)	<0.001
ALT (U/L)	28.9 (19.9, 44.3)	38.6 (23.8, 64.0)	0.002
AST (U/L)	30.7 (23.1, 48.1)	62.2 (38.4, 121.1)	<0.001
TBIL (μmol/L)	16.2 (11.3, 24.4)	30.4 (17.3, 56.8)	<0.001
ALB (g/L)	37.7 ± 6.3	32.6 ± 6.3	<0.001
γ-GGT (U/L)	40.8 (23.4, 81.2)	121.0 (51.5, 231.9)	<0.001
PLT (×10^9^/L)	92.2 (60.3, 142.0)	101.4 (64.0, 157.8)	<0.001
INR	1.1 (1.0, 1.2)	1.2 (1.1, 1.4)	<0.001
Cr (μmol/L)	67.0 (58.0, 76.7)	68.0 (58.0, 81.8)	<0.001
AFP (ng/mL) (≥400)	124 (12.7)	272 (40.8)	<0.001
Neutrophils (×10^9^/L)	2.3 (1.6, 3.2)	3.4 (2.2,5.2)	<0.001
Lymphocytes (×10^9^/L)	1.2 (0.8, 1.7)	0.9 (0.7, 1.3)	<0.001
Monocytes (×10^9^/L)	0.4 (0.3, 0.5)	0.5 (0.3, 0.7)	0.011
TC (mmol/L)	3.7 (3.2, 4.3)	3.4 (2.8, 4.2)	0.029
TG (mmol/L)	0.8 (0.6, 1.1)	0.8 (0.6, 1.1)	0.376
HDL-C (mmol/L)	1.0 (0.9, 1.3)	0.8 (0.5, 1.1)	0.004
LDL-C (mmol/L)	2.0 (1.6, 2.6)	2.0 (1.5, 2.7)	0.140
NHR	2.2 (1.4, 3.5)	4.3 (2.3, 9.5)	<0.001
MHR	0.3 (0.2, 0.5)	0.5 (0.3,1.0)	0.011
LHR	1.2 (0.7, 1.7)	1.3 (0.8, 2.2)	<0.001
**Child Pugh class**			<0.001
A	712 (73.2)	170 (25.5)	
B	203 (20.9)	253 (38.0)	
C	58 (5.9)	243 (36.5)	
**Tumor–related indicators**			
Tumor multiplicity (multiple)	303 (31.1)	450 (67.5)	<0.001
Tumor size, cm (≥ 5)	212 (21.8)	323 (48.4)	<0.001
**BCLC stage**			<0.001
0–A	498 (51.2)	66 (9.9)	
B	404 (41.5)	135 (20.3)	
C	34 (3.5)	258 (38.7)	
D	37 (3.8)	207 (31.1)	
**Types of treatment**			<0.001
Resection	138 (14.2)	13 (1.9)	
Minimally invasive	817 (84.0)	308 (46.2)	
Palliative	18 (1.8)	345 (51.8)	

HCC, hepatocellular carcinoma; MELD, Model for End-Stage Liver Disease; ALT, alanine aminotransferase; AST, aspartate aminotransferase; TBIL, total bilirubin; ALB, albumin; GGT, γ-glutamyl transferase; PLT, platelet count; Cr, creatinine; INR, international normalized ratio; AFP, alpha-fetoprotein; TC, total cholesterol; TG, triglyceride; HDL-C, high-density lipoprotein cholesterol; LDL-C, low-density lipoprotein cholesterol; NHR, neutrophil to HDL-C ratio; MHR, monocyte to HDL-C ratio; LHR, lymphocyte to HDL-C ratio; BCLC, Barcelona Clinic Liver Cancer.

### Associations of biomarkers with prognosis in patients

Univariate analysis showed that low HDL-C, high NHR, MHR, and LHR levels significantly increased the risk of 3-year mortality as both continuous and categorical variables (all *p* < 0.001; Table 3). In addition, univariate analysis indicated that age, sex, diabetes, alcohol consumption, alanine aminotransferase, aspartate aminotransferase, platelet count, alpha-fetoprotein, total cholesterol, Child-Pugh class, MELD score, Barcelona Clinic Liver Cancer (BCLC) stage, tumor size, and type of treatment were significantly associated with the 3-year mortality (all *p* < 0.05). These significant factors were included in the multivariate Cox regression analysis. After adjustment for confounding variables, these significant associations were found with low HDL-C (aHR, 0.31; 95% CI: 0.23–0.41, *p* < 0.001) and high NHR levels (aHR, 1.02; 95% CI: 1.01–1.03, *p* < 0.001) as continuous variables. However, the association of 3-year mortality with LHR and MHR was attenuated.

**Table 3:** Univariate and multivariate Cox hazards analysis for 3-year mortality among patients with HCC.

**Variable**	**Univariate HR**	*p* **-value**	**Adjusted HR** *	*p* **-value**
	**(95%CI)**		**(95%CI)**	
HDL-C	0.13 (0.10–0.17)	<0.001	0.31 (0.23–0.41)	<0.001
Q1	Reference		Reference	
Q2	0.21 (0.18–0.25)	<0.001	0.46 (0.37–0.57)	<0.001
Q3	0.21 (0.16–0.26)	<0.001	0.38 (0.28–0.51)	<0.001
NHR	1.04 (1.03–1.05)	<0.001	1.02 (1.01–1.03)	<0.001
Q1	Reference		Reference	
Q2	2.33 (2.05–2.65)	0.014	1.75 (1.25–2.31)	0.001
Q3	5.08 (4.07–6.35)	<0.001	3.08 (2.25–4.22)	<0.001
LHR	1.08 (1.06–1.10)	<0.001	1.00 (0.96–1.04)	0.966
Q1	Reference		Reference	
Q2	1.11 (0.89–1.40)	0.337	1.24 (0.98–1.56)	0.067
Q3	2.13 (1.68–2.70)	<0.001	1.45 (1.13–1.86)	0.003
MHR	1.01 (1.00–1.02)	<0.001	1.00 (0.99–1.01)	0.277
Q1	Reference		Reference	
Q2	0.90 (0.74–1.09)	0.288	0.92 (0.75–1.18)	0.551
Q3	1.58 (1.28–1.93)	<0.001	1.43 (1.19–1.98)	0.001

* Adjusted for age, sex, diabetes, alcohol consumption, ALT, AST, PLT, AFP, TC, Child-Pugh class, MELD score, BCLC stage, tumor size, and type of treatment. HCC, hepatocellular carcinoma; ALT, alanine aminotransferase; AST, aspartate aminotransferase; PLT, platelet count; AFP, alpha-fetoprotein; TC, total cholesterol; HDL-C, high-density lipoprotein cholesterol; NHR, neutrophil to HDL-C ratio; MHR, monocyte to HDL-C ratio; LHR, lymphocyte to HDL-C ratio; MELD, Model for End-Stage Liver Disease; HR, Hazard ratio; CI, confidence interval.

### Prognosis value of lipid-related biomarkers in patients

**Figure 2:**
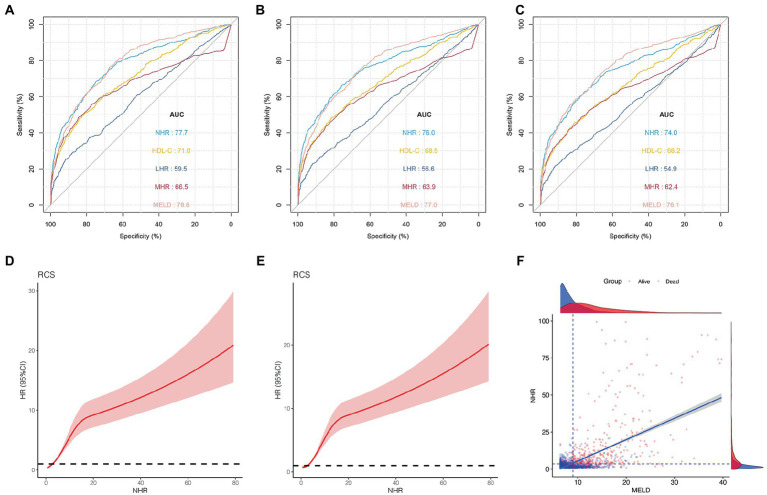
Predictive ability of different indicators for mortality and the association between NHR and outcome in the training cohort. ROC curves of NHR, HDL-C, MHR, LHR, and MELD score predicting 1 **(A)**, 2 **(B)**, and 3  years mortality **(C)**. **(D)** The association between NHR and 1-year mortality in all patients (unadjusted). **(E)** The association between NHR and 3-year mortality in all patients (unadjusted). **(F)** The distribution of survival and death patients in patients with HCC. Scatterplots using axis cut-points of ≥3.5 for NLR and ≥9 for the MELD score. ROC, receiver operating characteristic; HDL-C, high-density lipoprotein cholesterol; NHR, neutrophil to HDL-C ratio; MHR, monocyte to HDL-C ratio; LHR, lymphocyte to HDL-C ratio; MELD, Model for End-stage Liver Disease; BCLC, Barcelona Clinic Liver Cancer.

Figures 2A–C show the 1-, 2-, and 3-year prognostic values of HDL-C, NHR, MHR, and LHR. Moreover, the performance of these markers was compared with that of the MELD score, a well-established prognosis score. In the training cohort, the AUCs of the NHR for mortality were 0.777 (95% CI 0.752–0.802), 0.760 (95% CI 0.722–0.784), and 0.740 (95% CI 0.715–0.767) at 1, 2, and 3 years, respectively. The NHR showed a similar predictive ability, compared to the MELD score (AUCs 1, 2, and 3 years: 0.786, 0.770, and 0.761, respectively). In addition, we compared the NHR with other lipid-related indicators, including HDL-C, MHR, and LHR. The results indicated that the AUC of the NHR was significantly higher than those of the HDL-C, MHR, and LHR at 1, 2, and 3 years (all *p* < 0.05).

As shown in Figures 2D,E, the risk was relatively low in the low NHR range and then increased in patients with HCC. These results indicate that the NHR was associated with the 1-and 3-year mortality risk and the test for nonlinearity was statistically significant (*p* < 0.001).

### Optimal cut-off points for the NHR and MELD score

The optimal cut-off points for the NHR and MELD scores were determined using the X-tile software. When NHR ≥ 3.5 and MELD ≥ 9, the difference was the most statistically significant. The sensitivity and specificity of the NHR ≥ 3.5 were 70.1 and 74.7%, respectively, and those of the MELD ≥ 9 were 76.3 and 67.4%, respectively. Scatterplots were used to visualize the relationship between NHR, MELD score, and mortality in patients with HCC (Figure 2F). The scatterplots revealed that patients with NHR ≥ 3.5 and MELD ≥ 9 had poor outcomes in patients with HCC.

### Risk stratification for patients with HCC

**Figure 3:**
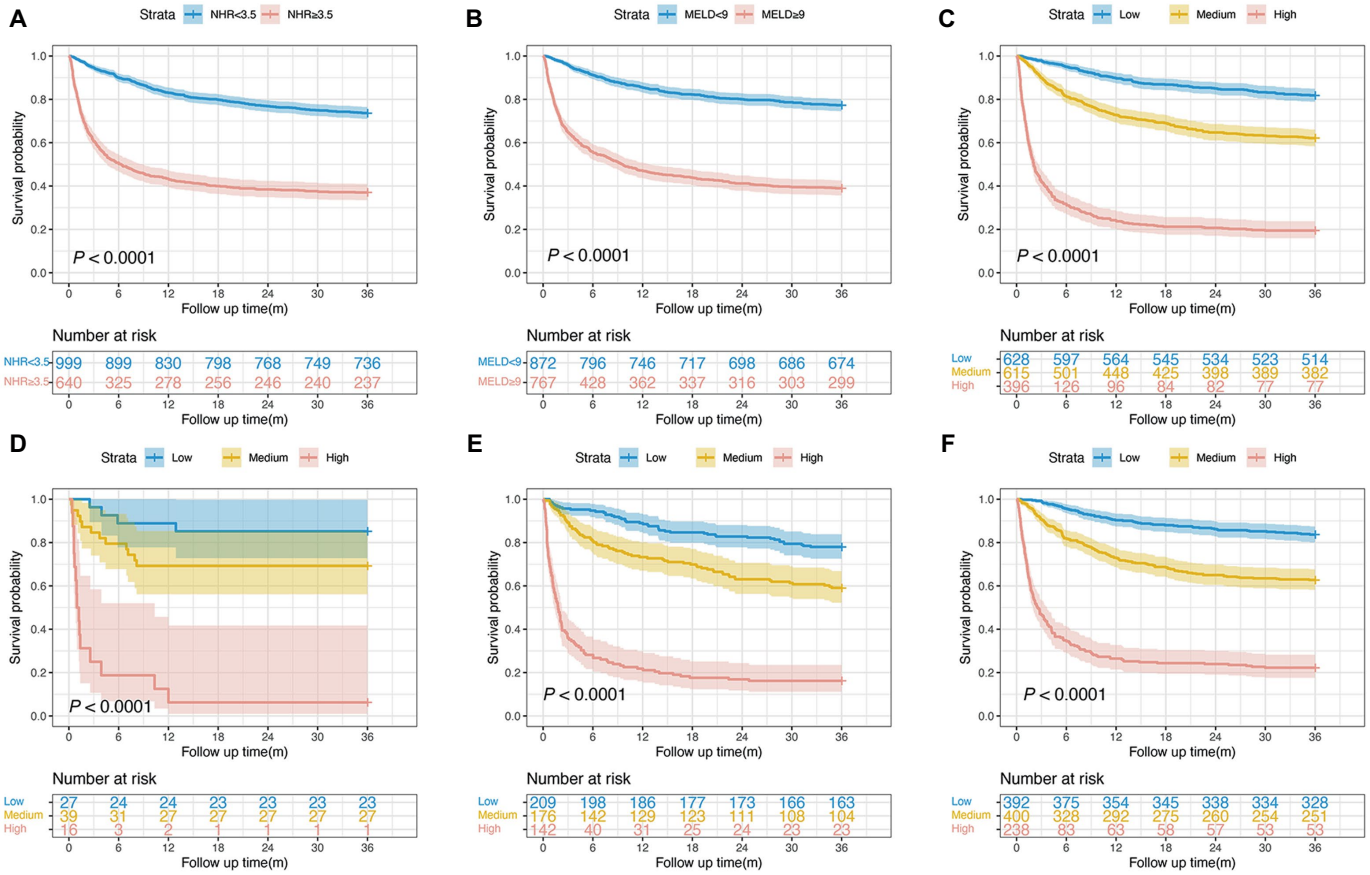
Survival curves of patients with HCC in the training cohort. **(A)** Survival probability in patients with NHR < 3.5 and ≥3.5 (*n* = 1,639, 73.7 vs. 37.0%, *p* < 0.0001). **(B)** Survival probability in patients with MELD <9 and ≥9 (*n* = 1,639, 77.3 vs. 39.0%, *p* < 0.0001). **(C)** Survival probability in the low-, medium-, and high-risk group (*n* = 1,639, 81.8 vs. 62.1 vs. 19.4%, *p* < 0.0001). **(D)** Survival probability of patients aged <40  years in the low-, medium-, and high-risk group (*n* = 82, 85.2 vs. 69.2 vs. 6.2%, *p* < 0.0001). **(E)** Survival probability of patients aged 40–60  years in the low-, medium-, and high-risk group (*n* = 527, 78.0 vs. 59.0 vs. 16.2%, *p* < 0.0001). **(F)** Survival probability of patients aged >60  years in the low-, medium-, and high-risk group (*n* = 1,030, 83.7 vs. 62.7 vs. 22.3%, *p* < 0.0001). HCC, hepatocellular carcinoma; MELD, Model for end-stage liver disease; NHR, neutrophil to high-density lipoprotein cholesterol ratio.

The Kaplan–Meier survival curves based on the NHR and MELD optimal cut-off values are shown in Figure 3. We observed a statistical difference between cut-off values of these two biomarkers and survival probability. The 3-year survival probability was 73.7% in patients with NHR < 3.5, whereas those with NHR ≥ 3.5 were 37.0% ( *p* < 0.0001; Figure 3A). Furthermore, patients with MELD score < 9 had a significantly higher survival probability than those with MELD score ≥ 9 (77.3 vs. 39.0%, *p* < 0.0001; Figure 3B). Next, all patients were divided into three groups: low- (NHR < 3.5 and MELD <9, *n* = 628), medium- (NHR ≥ 3.5 or MELD ≥9, *n* = 615), and high-risk groups (NHR ≥ 3.5 and MELD ≥9, *n* = 396). The 3-year survival probabilities were 81.8, 62.1, and 19.4% in the low-, medium-, and high-risk groups, respectively ( *p* < 0.0001; Figure 3C).

To examine the risk stratification for different ages, we divided patients into the following three subgroups: <40, 40–60, and >60 years. The Kaplan–Meier curve analysis showed that the 3-year survival probability in patients with a NHR < 3.5 and a MELD score < 9 was significantly higher than in patients with a NHR ≥ 3.5 and a MELD score ≥ 9, regardless of patient’s ages (all *p* < 0.0001; Figures 3D–F).

### Prognostic value of NHR in the diabetic subgroup

**Figure 4:**
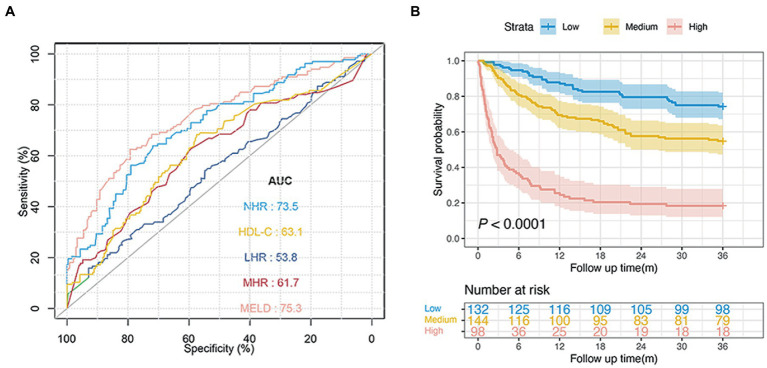
Predictive ability of different indicators for 3-year mortality and risk stratification in patients with diabetes. **(A)** ROC curves of NHR, HDL-C, MHR, LHR, and MELD score predicting 3-year mortality in diabetic patients. **(B)** Survival probability of diabetic patients in the low-, medium-, and high-risk group (*n* = 374, 74.2 vs. 54.9 vs. 18.4%, *p* < 0.0001). NHR, neutrophil to high-density lipoprotein cholesterol ratio; HDL-C, high-density lipoprotein cholesterol; MHR, monocyte to HDL-C ratio; LHR, lymphocyte to HDL-C ratio; MELD, Model for End-stage Liver Disease.

For the diabetic subgroup, the NHR had the highest AUC at 3-year (0.735; 95% CI: 0.681–0.779). The AUCs of the HDL-C, MHR, LHR, and MELD scores were 0.631 (95% CI: 0.573–0.688), 0.617 (95% CI: 0.558–0.675), 0.538 (95% CI: 0.474–0.592), and 0.753 (95% CI: 0.704–0.805), respectively. The NHR showed better performance than HDL-C, MHR, and LHR (all *p* < 0.05, Figure 4A). The 3-year survival probabilities of patients in the low-, medium-, and high-risk groups were 74.2, 54.8, and 18.4%, respectively ( *p* < 0.0001; Figure 4B).

### Validation of prognostic values of the NHR and the MELD score

**Figure 5:**
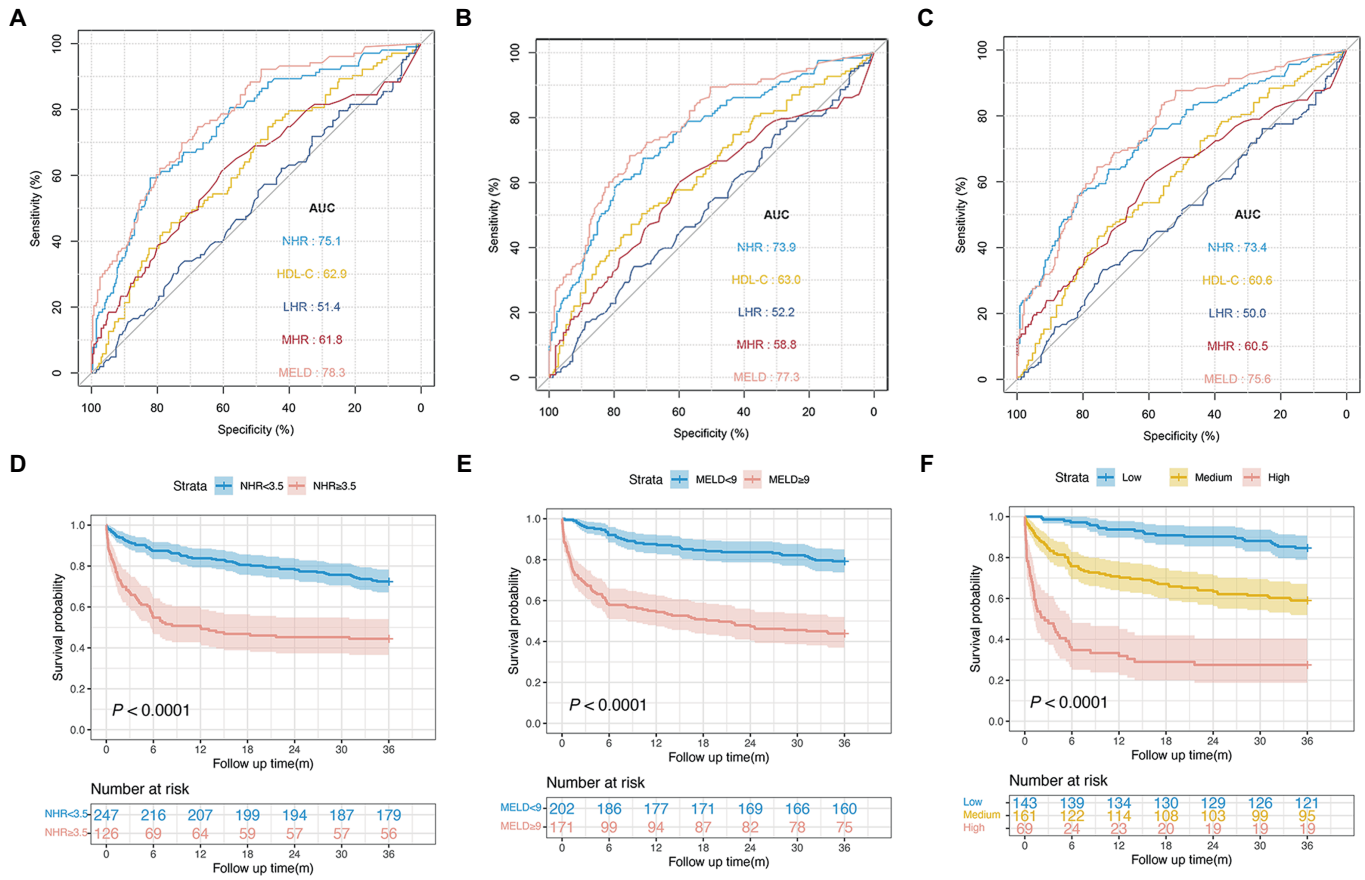
Performance of different indicators and risk stratification in the validation cohort. ROC curves of NHR, HDL-C, MHR, LHR, and MELD score predicting 1 **(A)** , 2 **(B)** , and 3 years mortality ****(C)**. **(D)**** Survival probability in patients with NHR < 3.5 and ≥3.5 (*n* = 373, 72.5 vs. 44.4%, *p* < 0.0001). **(E)** Survival probability in patients with MELD <9 and ≥9 (*n* = 373, 79.2 vs. 43.8%, *p* < 0.0001). **(F)** Survival probability in the low-, medium-, and high-risk group (*n* = 373, 84.6 vs. 59.0 vs. 27.5%, *p* < 0.0001).

In the validation cohort, baseline NHR offered good prognostic value for mortality with the AUC at 1, 2, and 3 years: 0.751 (95% CI: 0.696–0.807), 0.739 (95% CI: 0.695–0.785), and 0.734 (95% CI: 0.684–0.777), respectively. In addition, the NHR showed a performance similar to that of the MELD score (0.756; 95% CI 0.706–0.807) and was noted to have the highest AUC (0.734), which was followed by AUCs of the HDL-C (0.606; 95% CI 0.517–0.640), MHR (0.605; 95% CI 0.542–0.667), and LHR (0.500; 95% CI 0.438–0.561) at 3 years (all *p* < 0.05; Figures 5A–C).

Furthermore, patients with a NHR < 3.5 had a significantly higher survival probability than those with a NHR ≥ 3.5 (72.5 vs. 44.4%, *p* < 0.0001; Figure 5D). Patients with a MELD score ≥ 9 were associated with an increased risk of death compared with those with a MELD score < 9 ( *p* < 0.0001; Figure 5E). The 3-year survival probabilities in patients in the low- (NHR < 3.5 and MELD <9, *n* = 143), medium- (NHR ≥ 3.5 or MELD ≥9, *n* = 161), and high-risk groups (NHR ≥ 3.5 and MELD ≥9, *n* = 69) were 84.6, 59.0, and 27.5%, respectively (*p* < 0.001; Figure 5F).

## Discussion

HCC is the most common fatal malignant tumor with rapid progression and poor prognosis (27). Therefore, identifying high-risk patients and developing individualized therapies is essential. There exists an emerging interest in the relationship between lipid-related inflammatory parameters and the prognosis of liver disease. NHR, MHR, and LHR are novel parameters that can be readily acquired from routine blood examinations. However, there is little evidence regarding their prognostic value for mortality in patients with HCC.

To our knowledge, this study with a large sample size is the first to clarify the relationship between lipid-related inflammatory biomarkers and 3-year mortality in patients with HCC. Multivariate analyses revealed that the NHR was a significant independent factor for 3-year mortality in patients with HCC, regardless of continuous or categorical variables (all *p* < 0.05). Additionally, compared with the HDL-C, MHR, LHR, and MELD scores, the NHR exhibited a better or comparable performance in predicting prognosis. The results indicate that the NHR can effectively predict mortality in patients with HCC. Moreover, we observed that NHR had a nonlinear association with 1-and 3-year mortality (*p* for nonlinearity <0.001). The morbidity and mortality rates of HCC are higher in patients with diabetes than in the general population (28). In the current study, we found that the 3-year mortality rate was significantly higher in diabetic patients than in nondiabetic patients ( *p* < 0.001, Table 2). The NHR and MELD score had excellent discrimination in assessing the 3-year prognosis in patients with diabetes.

The pathogenesis of HCC is closely related to immune status and inflammatory response (29). When immune cells, including lymphocytes and neutrophils, are activated, proinflammatory and anti-inflammatory mediators are released (30). Neutrophils are the first line of the inflammatory response and produce cytokines that affect lymphocytes and monocytes (31), which may explain why the predictive ability of NHR is better than that of single markers (HDL-C) and other ratios (MHR and LHR) in the current study. The NHR is an effective biomarker of systemic inflammation and oxidative stress, and its prognostic power has been investigated (14, 32). Furthermore, as critical oxidative mediators, monocytes reveal the response capacity of the innate immune system (33). A decrease in circulating lipoprotein levels reflects the severity of the dysfunction of liver synthesis. Reduced HDL levels and function may play important roles in the pathophysiology of systemic inflammation (34). Previous studies indicated that HDL-C and apolipoprotein A-I are negatively associated with inflammatory markers (34, 35). HDL-C inhibits the activation and transformation of monocytes, thereby inhibiting the inflammatory response (36). In this study, most patients presented with cirrhosis at the time of diagnosis. Trieb et al. (37) reported that patients with cirrhosis showed reduced levels of HDL-C, which impaired the ability of HDL-C to inhibit monocyte production of proinflammatory factors. In addition, the anti-inflammatory and antioxidant activities of HDLs are impaired in patients with diabetes (38). Proinflammatory cytokines directly inhibit the activity of apolipoprotein synthesis enzymes, leading to reduced production of HDL-C (8, 39). Abnormal activation of neutrophils results in changes in the composition and function of HDL-C and increases neutrophil production (40), thereby, increasing the risk of mortality.

The MELD score is composed of three available biochemical indicators: TBIL, Cr, and INR. The MELD score is an accurate mortality risk assessment tool in chronic liver disease (41, 42). This score has predicted mortality in patients with HCC (43, 44). In the current population-based cohort study, we found that NHR and MELD scores had similar prognostic values. According to the scatter plot distribution and Kaplan–Meier curves, patients with higher NHR and MELD scores had poorer prognoses. In training cohort, patients in the low-risk group (NHR < 3.5 and MELD <9) had a 3-year survival rate of 81.8%, and patients in the high-risk group (NHR ≥ 3.5 and MELD ≥9) had a 3-year survival rate of 19.4%. Since HCC mortality rates remain considerable despite advanced treatments, the NHR and MELD scores are critical for clinicians to identify high-risk patients and facilitate appropriate and timely patient management.

Our study has some limitations. First, this study was a single-institution one with retrospective data collection. However, the result was validated using an internal cohort and NHR showed good discrimination. Second, in the training and validation cohorts, 151 (9.2%) and 30 (8.0%) patients with HCC underwent liver resection, respectively. Most patients receive local treatment, such as TACE or palliative treatment. In the future, a prospective multicenter large-sample study is required to confirm its prognostic value in patients with HCC underwent liver resection. Third, since hepatitis B virus infection is a common cause of HCC in China, whether NHR may be valuable in patients with other etiologies remains unclear.

In conclusion, a high NHR is a powerful independent risk factor for mortality and can be used to evaluate the prognosis of HCC. The association of patients having MELD ≥9 and NHR ≥ 3.5 with poor prognosis can aid clinicians in identifying high-risk patients.

## Data availability statement

The raw data supporting the conclusions of this article will be made available by the authors, without undue reservation.

## Ethics statement

The studies involving human participants were reviewed and approved by The Ethics Committee of Beijing Ditan Hospital. Written informed consent for participation was not required for this study in accordance with the national legislation and the institutional requirements.

## Author contributions

XW and KS conceived and designed the project. KS, JH, QZ, and YB collected the data. KS and JH analyzed and interpreted the data. KS drafted the manuscript. QZ, YB, and XZ was responsible for manuscript modification. All authors contributed to the article and approved the submitted version.

## Funding

This work was supported by the Beijing Municipal Science Technology Commission (no. Z191100006619033).

## Conflict of interest

The authors declare that the research was conducted in the absence of any commercial or financial relationships that could be construed as a potential conflict of interest.

## Publisher’s note

All claims expressed in this article are solely those of the authors and do not necessarily represent those of their affiliated organizations, or those of the publisher, the editors and the reviewers. Any product that may be evaluated in this article, or claim that may be made by its manufacturer, is not guaranteed or endorsed by the publisher.

## References

[1] Kulik L El-Serag HB. Epidemiology and management of hepatocellular carcinoma. Gastroenterology. (2019) 156:477–491.e1. doi: 10.1053/j.gastro.2018.08.065, PMID: 30367835 PMC6340716

[2] Forner A Reig M Bruix J. Hepatocellular carcinoma. Lancet. (2018) 391:1301–14. doi: 10.1016/S0140-6736(18)30010-2 29307467

[3] Bray F Ferlay J Soerjomataram I Siegel RL Torre LA Jemal A. Global cancer statistics 2018: GLOBOCAN estimates of incidence and mortality worldwide for 36 cancers in 185 countries. CA Cancer J Clin. (2018) 68:394–424. doi: 10.3322/caac.21492, PMID: 30207593

[4] De Angelis N Landi F Carra MC Azoulay D. Managements of recurrent hepatocellular carcinoma after liver transplantation: a systematic review. World J Gastroenterol. (2015) 21:11185–98. doi: 10.3748/wjg.v21.i39.11185, PMID: 26494973 PMC4607916

[5] Shawcross DL Davies NA Williams R Jalan R. Systemic inflammatory response exacerbates the neuropsychological effects of induced hyperammonemia in cirrhosis. J Hepatol. (2004) 40:247–54. doi: 10.1016/j.jhep.2003.10.016, PMID: 14739095

[6] Liu J Li H Xia J Wang X Huang Y Li B. Baseline neutrophil-to-lymphocyte ratio is independently associated with 90-day transplant-free mortality in patients with cirrhosis. Front Med (Lausanne). (2021) 8:726950. doi: 10.3389/fmed.2021.726950, PMID: 34532334 PMC8438214

[7] Shi K Li P Xue D Liu Y Zhang Q Ping R. Neutrophil-lymphocyte ratio and the risk of hepatocellular carcinoma in patients with hepatitis B-caused cirrhosis. Eur J Gastroenterol Hepatol. (2021) 33:e686–92. doi: 10.1097/MEG.0000000000002217, PMID: 34074986

[8] Tanaka S Couret D Tran-Dinh A Duranteau J Montravers P Schwendeman A. High-density lipoproteins during sepsis: from bench to bedside. Crit Care. (2020) 24:134. doi: 10.1186/s13054-020-02860-3, PMID: 32264946 PMC7140566

[9] Santos-Gallego CG Badimon JJ Rosenson RS. Beginning to understand high-density lipoproteins. Endocrinol Metab Clin N Am. (2014) 43:913–47. doi: 10.1016/j.ecl.2014.08.001, PMID: 25432389

[10] Habib A Mihas AA Abou-Assi SG Williams LM Gavis E Pandak WM. High-density lipoprotein cholesterol as an indicator of liver function and prognosis in noncholestatic cirrhotics. Clin Gastroenterol Hepatol. (2005) 3:286–91. doi: 10.1016/s1542-3565(04)00622-6, PMID: 15765449

[11] Etogo-Asse FE Vincent RP Hughes SA Auzinger G Roux CWL Wendon J. High density lipoprotein in patients with liver failure; relation to sepsis, adrenal function and outcome of illness. Liver Int. (2012) 32:128–36. doi: 10.1111/j.1478-3231.2011.02657.x 22098564

[12] Mathur K Vilar-Gomez E Connelly MA He H Sanyal AJ Chalasani N. Circulating high density lipoprotein distinguishes alcoholic hepatitis from heavy drinkers and predicts 90-day outcome: lipoproteins in alcoholic hepatitis. J Clin Lipidol. (2021) 15:805–13. doi: 10.1016/j.jacl.2021.10.002, PMID: 34756674 PMC8688310

[13] Chen G Yang N Ren J He Y Huang H Hu X. Neutrophil counts to high-density lipoprotein cholesterol ratio: a potential predictor of prognosis in acute ischemic stroke patients after intravenous thrombolysis. Neurotox Res. (2020) 38:1001–9. doi: 10.1007/s12640-020-00274-1, PMID: 32894456

[14] Jiang M Sun J Zou H Li M Su Z Sun W. Prognostic role of neutrophil to high-density lipoprotein cholesterol ratio for all-cause and cardiovascular mortality in the general population. *Front Cardiovasc Med*. (2022) 9:807339. doi: 10.3389/fcvm.2022.807339, PMID: 35211525 PMC8861276

[15] Li C Fan H Liu Y Zeng L Chen P Duan C. The monocyte to high-density lipoprotein cholesterol ratio and outcomes in type 2 diabetes mellitus patients with non-ST-segment elevation acute coronary syndrome. Ann Transl Med. (2021) 9:1627. doi: 10.21037/atm-21-4876, PMID: 34926671 PMC8640916

[16] You S Zhong C Zheng D Xu J Zhang X Liu H. Monocyte to HDL cholesterol ratio is associated with discharge and 3-month outcome in patients with acute intracerebral hemorrhage. J Neurol Sci. (2017) 372:157–61. doi: 10.1016/j.jns.2016.11.022, PMID: 28017204

[17] Yu S Guo X Li G Yang H Zheng L Sun Y. Lymphocyte to high-density lipoprotein ratio but not platelet to lymphocyte ratio effectively predicts metabolic syndrome among subjects from rural China. Front Cardiovasc Med. (2021) 8:583320. doi: 10.3389/fcvm.2021.583320, PMID: 33778016 PMC7994280

[18] Luo X Sui J Yang W Sun Q Ma Y Simon TG. Type 2 diabetes prevention diet and hepatocellular carcinoma risk in US men and women. Am J Gastroenterol. (2019) 114:1870–7. doi: 10.14309/ajg.0000000000000450, PMID: 31688024 PMC6893135

[19] Parhofer KG. Interaction between glucose and lipid metabolism: more than diabetic dyslipidemia. Diabetes Metab J. (2015) 39:353–62. doi: 10.4093/dmj.2015.39.5.353, PMID: 26566492 PMC4641964

[20] Busnelli M Manzini S Chiara M Colombo A Fontana F Oleari R. Aortic gene expression profiles show how ApoA-I levels modulate inflammation, lysosomal activity, and sphingolipid metabolism in murine atherosclerosis. Arterioscler Thromb Vasc Biol. (2021) 41:651–67. doi: 10.1161/ATVBAHA.120.315669, PMID: 33327742 PMC7837693

[21] Sarin SK Kumar M Lau GK Abbas Z Chan HL Chen CJ. Asian-Pacific clinical practice guidelines on the management of hepatitis B: a 2015 update. Hepatol Int. (2016) 10:1–98. doi: 10.1007/s12072-015-9675-4, PMID: 26563120 PMC4722087

[22] Shiha G Sarin SK Ibrahim AE Omata M Kumar A Lesmana LA. Liver fibrosis: consensus recommendations of the Asian Pacific Association for the Study of the liver (APASL). Hepatol Int. (2009) 3:323–33. doi: 10.1007/s12072-008-9114-x, PMID: 19669358 PMC2716768

[23] Bruix J Sherman M. Management of hepatocellular carcinoma: an update. Hepatology. (2011) 53:1020–2. doi: 10.1002/hep.24199, PMID: 21374666 PMC3084991

[24] Wiesner R Edwards E Freeman R Harper A Kim R Kamath P. Model for end-stage liver disease (MELD) and allocation of donor livers. Gastroenterology. (2003) 124:91–6. doi: 10.1053/gast.2003.50016 12512033

[25] DeLong ER DeLong DM Clarke-Pearson DL. Comparing the areas under two or more correlated receiver operating characteristic curves: a nonparametric approach. Biometrics. (1998) 44:837–45. doi: 10.2307/2531595 3203132

[26] Lee DH Keum N Hu FB Orav EJ Rimm EB Willett WC. Predicted lean body mass, fat mass, and all cause and cause specific mortality in men: prospective US cohort study. BMJ. (2018) 362:k2575. doi: 10.1136/bmj.k2575, PMID: 29970408 PMC6028901

[27] Ma L Chua MS Andrisani O So S. Epigenetics in hepatocellular carcinoma: an update and future therapy perspectives. World J Gastroenterol. (2014) 20:333–45. doi: 10.3748/wjg.v20.i2.333, PMID: 24574704 PMC3923010

[28] Fujita K Iwama H Miyoshi H Tani J Oura K Tadokoro T. Diabetes mellitus and metformin in hepatocellular carcinoma. World J Gastroenterol. (2016) 22:6100–13. doi: 10.3748/wjg.v22.i27.6100, PMID: 27468203 PMC4945972

[29] Yang YM Kim SY Seki E. Inflammation and liver cancer: molecular mechanisms and therapeutic targets. Semin Liver Dis. (2019) 39:026–42. doi: 10.1055/s-0038-1676806, PMID: 30809789 PMC6616367

[30] Li JY Yao RQ Liu SQ Zhang YF Yao YM Tian YP. Efficiency of monocyte/high-density lipoprotein cholesterol ratio combined with neutrophil/lymphocyte ratio in predicting 28-day mortality in patients with sepsis. Front Med (Lausanne). (2021) 8:741015. doi: 10.3389/fmed.2021.741015, PMID: 34722578 PMC8548423

[31] Rosales C. Neutrophils at the crossroads of innate and adaptive immunity. J Leukoc Biol. (2020) 108:377–96. doi: 10.1002/JLB.4MIR0220-574RR, PMID: 32202340

[32] Liu SL Feng BY Song QR Zhang YM Wu SL Cai J. Neutrophil to high-density lipoprotein cholesterol ratio predicts adverse cardiovascular outcomes in subjects with pre-diabetes: a large cohort study from China. Lipids Health Dis. (2022) 21:86. doi: 10.1186/s12944-022-01695-x, PMID: 36057713 PMC9441053

[33] Bar-Or D Bar-Or R Rael LT Brody EN. Oxidative stress in severeacute illness. Redox Biol. (2015) 4:340–5. doi: 10.1016/j.redox.2015.01.006, PMID: 25644686 PMC4326179

[34] Trieb M Rainer F Stadlbauer V Douschan P Horvath A Binder L. HDL-related biomarkers are robust predictors of survival in patients with chronic liver failure. J Hepatol. (2020) 73:113–20. doi: 10.1016/j.jhep.2020.01.026, PMID: 32061870

[35] Pirillo A Catapano AL Norata GD. HDL in infectious diseases and sepsis. Handb Exp Pharmacol. (2015) 224:483–508. doi: 10.1007/978-3-319-09665-0_15, PMID: 25522999

[36] Uca FM. A potential marker of bare metal stent restenosis: monocyte count-to-HDL cholesterol ratio. BMC Cardiovasc Disord. (2016) 16:186. doi: 10.1186/s12872-016-0367-3, PMID: 27716070 PMC5048646

[37] Trieb M Horvath A Birner-Gruenberger R Spindelboeck W Stadlbauer V Taschler U. Liver disease alters high-density lipoprotein composition, metabolism and function. Biochim Biophys Acta. (2016) 1861:630–8. doi: 10.1016/j.bbalip.2016.04.013, PMID: 27106140 PMC5542032

[38] Morgantini C Natali A Boldrini B Imaizumi S Navab M Fogelman AM. Anti-inflammatory and antioxidant properties of HDLs are impaired in type 2 diabetes. Diabetes. (2011) 60:2617–23. doi: 10.2337/db11-0378, PMID: 21852676 PMC3178289

[39] Golucci A Marson FAL Ribeiro AF Nogueira RJN. Lipid profile associated with the systemic inflammatory response syndrome and sepsis in critically ill patients. Nutrition. (2018) 55-56:7–14. doi: 10.1016/j.nut.2018.04.007, PMID: 29960160

[40] Bergt C Marsche G Panzenboeck U Heinecke JW Malle E Sattler W. Human neutrophils employ the myeloperoxidase/hydrogen peroxide/chloride system to oxidatively damage apolipoprotein A-I. Eur J Biochem. (2001) 268:3523–31. doi: 10.1046/j.1432-1327.2001.02253.x, PMID: 11422382

[41] Said A Williams J Holden J Remington P Gangnon R Musat A. Model for end stage liver disease score predicts mortality across a broad spectrum of liver disease. J Hepatol. (2004) 40:897–903. doi: 10.1016/j.jhep.2004.02.010 15158328

[42] Bernardi M Gitto S Biselli M. The MELD score in patients awaiting liver transplant: strengths and weaknesses. J Hepatol. (2011) 54:1297–306. doi: 10.1016/j.jhep.2010.11.008, PMID: 21145851

[43] Cucchetti A Ercolani G Vivarelli M Cescon M Ravaioli M Barba GL. Impact of model for end-stage liver disease (MELD) score on prognosis after hepatectomy for hepatocellular carcinoma on cirrhosis. Liver Transpl. (2006) 12:966–71. doi: 10.1002/lt.20761, PMID: 16598792

[44] Delis SG Bakoyiannis A Biliatis I Athanassiou K Tassopoulos N Dervenis C. Model for end-stage liver disease (MELD) score, as a prognostic factor for post-operative morbidity and mortality in cirrhotic patients, undergoing hepatectomy for hepatocellular carcinoma. HPB (Oxford). (2009) 11:351–7. doi: 10.1111/j.1477-2574.2009.00067.x, PMID: 19718364 PMC2727090

