# Supplementary material for: Neutrophil-to-high-density-lipoprotein-cholesterol ratio and mortality among patients with hepatocellular carcinoma
Source: Front Nutr. 2023 May 5;10:1127913. doi: 10.3389/fnut.2023.1127913 (PMC10198653; doi:10.3389/fnut.2023.1127913)
Supplement: Supplementary file 1 [file Data_Sheet_1.PDF]

ID	Sex	Age	time	status	smoking	drinking	C family hist	DM
341290	1	53	12	0	1	1	0	1
335935	1	53	12	0	0	0	0	0
548492	2	83	9.7	1	0	0	0	0
340503	2	66	12	0	0	0	0	0
329360	2	57	5.47	1	1	0	0	0
234490	1	53	8	1	1	1	0	0
442275	2	57	12	0	0	0	0	1
353992	1	77	12	0	0	0	0	0
248907	1	68	12	0	0	0	0	0
273046	1	43	12	0	1	1	1	0
234732	1	42	12	0	1	0	1	0
236958	1	56	12	0	1	0	1	1
315030	1	39	12	0	1	1	0	0
358659	1	59	8.2	1	0	1	0	0
340824	1	76	12	0	1	1	0	1
245742	1	49	12	0	0	1	0	0
237154	1	68	12	0	1	0	0	0
437847	1	73	12	0	0	0	0	1
237184	1	43	12	0	1	0	0	0
237201	1	49	12	0	1	0	1	0
372701	2	67	2.8	1	0	0	0	1
358662	1	76	12	0	0	0	0	1
273262	1	46	1.3	1	1	0	0	0
252654	1	59	12	0	0	1	1	0
273639	2	48	12	0	0	0	0	0
273659	1	50	12	0	1	1	0	0
273768	1	63	12	0	1	1	0	0
273862	1	45	4.63	1	1	1	1	0
333859	1	63	12	0	1	1	1	1
349208	1	62	12	0	0	0	0	0
274335	1	75	12	0	0	0	0	0
274337	1	55	12	0	0	0	0	0
274492	1	58	12	0	1	1	0	0
275021	1	63	12	0	0	1	0	0
347455	1	55	8.77	1	1	0	0	0
242654	1	52	12	0	1	0	0	0
275152	1	43	12	0	1	0	0	0
399906	2	68	12	0	0	0	0	0
308880	1	47	12	0	0	0	0	0
242708	1	66	12	0	0	0	0	0
234893	1	45	12	0	1	0	0	1
381293	1	61	12	0	1	0	0	0
243856	2	46	12	0	0	0	0	0

244460	1	55	12	0	1	1	0	0
326757	2	65	12	0	0	0	0	0
245141	2	61	9.9	1	0	0	0	0
334624	1	43	5.6	1	1	0	0	1
245455	2	53	12	0	0	0	0	0
302825	1	58	12	0	0	1	0	0
245790	1	62	9.9	1	1	1	0	0
246753	1	59	12	0	0	0	0	0
247079	1	30	12	0	1	0	0	0
246852	2	57	12	0	0	0	1	0
249030	2	63	12	0	0	0	0	0
247521	1	37	12	0	1	1	1	0
248621	1	42	2.9	1	1	1	1	1
404321	1	53	12	0	0	0	0	0
370356	2	68	12	0	0	0	0	1
248331	1	69	12	0	0	0	0	0
422715	2	59	12	0	0	0	1	0
248516	2	78	12	0	0	0	0	0
386349	1	54	12	0	0	0	0	0
248988	1	39	2.5	1	0	0	0	0
248866	2	60	12	0	0	0	0	0
249520	2	59	12	0	0	0	0	0
315494	2	63	12	0	0	0	0	0
361158	2	63	12	0	0	0	0	1
249709	1	43	12	0	1	1	0	0
250222	1	38	12	0	0	1	0	0
249968	1	76	12	0	0	0	0	1
253956	1	68	12	0	1	1	0	0
286399	1	66	12	0	0	0	0	0
340880	1	46	12	0	0	1	0	0
344453	1	54	12	0	1	1	0	0
254531	1		12	0	1	1	0	0
365063	1	54	12	0	1	0	0	0
250465	2	60	12	0	0	0	0	1
373688	1	61	8.8	1	0	1	0	1
302902	1	57	12	0	1	0	0	0
250538	1	55	12	0	1	1	0	1
261376	2	49	12	0	0	0	0	0
292182	1	52	12	0	1	1	0	0
362190	1	50	12	0	1	1	0	1
469620	1	69	12	0	0	0	0	0
337965	1	51	12	0	0	0	0	0
438187	1	46	12	0	0	0	0	0
251993	1	58	2.2	1	1	1	0	0

281299	1	55	12	0	1	1	0	0
332559	1	40	12	0	1	1	0	0
302946	2	55	12	0	0	0	0	0
252181	1	54	12	0	0	0	0	1
252930	1	50	12	0	1	1	0	1
252892	1	47	12	0	1	1	0	0
325026	2	59	12	0	0	0	0	0
338499	1	64	12	0	0	0	0	0
281479	1	52	12	0	0	1	0	0
433935	1	56	12	0	1	1	0	1
252710	1	74	12	0	0	0	0	1
252655	1	50	12	0	1	1	0	0
254805	1		9	1	0	1	0	0
253148	2	58	12	0	0	0	0	0
253274	1	57	12	0	1	1	0	0
254509	2	53	12	0	0	0	0	0
409450	1	51	12	0	0	1	1	0
264428	1	60	12	0	1	1	0	0
254090	2	52	12	0	0	0	0	0
378774	1	54	12	0	0	0	0	0
254204	2	58	12	0	0	0	0	0
254511	1	38	12	0	0	0	1	0
254371	1	54	12	0	1	0	0	1
255182	1	53	12	0	1	0	0	0
254229	1	45	1.27	1	1	1	0	0
269920	1	59	12	0	0	1	0	0
254719	1	59	12	0	1	1	0	0
254636	2	59	12	0	0	0	0	1
255188	1	38	7.13	1	1	1	0	0
255350	1	38	12	0	0	0	1	0
363245	1	54	12	0	0	0	0	0
255617	1	46	12	0	1	1	0	0
258715	2	52	5.83	1	0	0	1	0
258727	1	52	4.2	1	1	1	0	0
255862	1	47	12	0	1	0	0	1
255415	1	38	5.87	1	0	0	0	0
258744	1	65	12	0	1	1	0	1
399316	2	71	12	0	0	0	0	1
262305	1	52	12	0	0	1	0	0
259331	1	57	1.97	1	1	0	0	1
262445	1		12	0	0	1	0	0
322059	1	64	12	0	1	1	0	0
260992	2	80	12	0	0	0	1	0
262123	1	61	12	0	1	1	0	0

386721	2	53	12	0	0	0	1	0
296726	2	56	12	0	0	0	0	1
262311	2	62	12	0	0	0	0	0
308972	2	58	9.4	1	0	0	0	1
427075	1	62	12	0	1	1	0	0
289439	1	66	12	0	1	1	0	1
262390	2	70	12	0	0	0	0	0
263571	2	47	12	0	0	0	0	1
263145	1	40	12	0	1	1	0	0
262921	1	41	12	0	0	0	0	0
262963	1	63	7.53	1	0	0	1	1
262720	1	62	12	0	1	1	0	0
328898	1	44	12	0	0	0	0	0
439932	1	57	12	0	0	0	0	1
333274	1	36	12	0	0	1	0	0
308984	1	60	12	0	0	0	0	0
380067	1	55	12	0	0	0	0	0
344516	1	40	12	0	1	1	0	1
263842	1	52	12	0	0	1	0	0
264542	2	44	12	0	0	0	0	0
264894	1	41	12	0	0	0	0	1
435532	1	56	12	0	1	0	0	0
268223	1	62	12	0	0	0	0	0
265199	1	63	12	0	0	0	0	0
264477	1	72	12	0	0	0	0	0
264364	2	59	8.13	1	0	0	0	0
265067	1	52	12	0	1	1	1	0
266702	1	70	12	0	1	0	0	0
264908	2	52	12	0	0	0	0	0
266129	1	44	5.6	1	1	1	0	0
265026	2	64	12	0	0	0	0	0
322088	2	61	1.8	1	0	0	0	0
265061	1	64	12	0	1	0	0	0
336579	1	69	12	0	0	1	0	0
265329	1	48	2.47	1	1	1	0	1
265891	1	56	12	0	0	0	0	1
265390	1	54	12	0	1	1	0	0
266267	1	57	12	0	0	1	0	0
266223	1	60	12	0	0	0	1	0
351414	1	59	12	0	1	1	0	0
269524	1	56	12	0	1	1	0	1
329498	1	49	12	0	0	0	0	0
266611	1	56	8.1	1	0	0	0	0
424199	1	51	12	0	1	1	0	0

289491	2	63	12	0	0	0	1	0
402440	1	58	12	0	1	1	0	0
266507	1	43	12	0	1	1	1	0
426905	1	60	12	0	0	0	0	0
268317	1	55	12	0	1	1	0	1
430636	1	63	12	0	0	0	0	0
269230	1	49	12	0	1	0	0	1
270003	1	53	6.33	1	1	1	0	0
387662	2	73	12	0	1	1	0	0
453827	1	59	12	0	1	1	0	0
269357	1	59	12	0	1	0	0	1
269936	1	64	12	0	0	1	0	0
433656	1	70	12	0	1	1	0	0
269717	1	42	12	0	0	1	0	0
271921	1	56	12	0	0	1	0	0
269820	1	77	12	0	0	0	0	0
271929	1	53	12	0	1	1	0	0
439885	1	49	12	0	1	1	0	1
271964	1	55	12	0	1	0	1	0
283620	1	55	12	0	1	1	0	1
272032	2	63	12	0	0	0	0	0
269900	1	54	2.07	1	1	1	0	0
309058	1	40	12	0	0	1	0	0
328641	1	61	12	0	1	1	0	1
279310	1	40	12	0	1	1	0	0
272317	1	51	3.27	1	1	1	0	0
272391	2	43	12	0	0	0	0	0
272412	1	57	12	0	1	1	0	0
351597	2	59	12	0	0	0	0	0
402656	1	61	12	0	1	1	0	0
272639	1	47	12	0	1	1	0	0
279437	1	49	12	0	1	1	0	0
279486	1	56	12	0	1	0	1	0
401304	1	62	12	0	0	0	0	1
283686	2	61	12	0	0	0	0	1
344572	2	52	12	0	0	0	0	0
332271	2	57	12	0	0	0	0	0
289599	2	68	12	0	0	0	0	0
452817	1	49	12	0	1	0	0	0
280793	1	50	12	0	1	1	0	0
432231	1	48	12	0	0	1	0	0
280618	1	39	2.57	1	1	1	0	0
280801	2	45	12	0	0	0	0	0
374633	1	45	12	0	0	0	0	0

281184	1	44	3.63	1	1	1	0	0
283777	1	48	5.1	1	1	1	0	0
281502	1	57	12	0	1	1	0	0
450059	2	75	12	0	0	0	0	1
394632	2	60	12	0	0	0	1	1
398495	1	47	12	0	1	1	0	1
341753	1	54	5.3	1	0	0	0	0
286737	2	66	12	0	0	0	0	0
294290	2	61	12	0	0	0	0	0
286769	1	42	12	0	1	1	0	0
292417	1	74	12	0	0	0	0	1
312618	2	62	12	0	0	0	0	0
286963	1	57	12	0	0	0	0	0
287053	1	66	12	0	1	0	0	0
289727	1	69	12	0	1	0	0	0
289729	1	43	12	0	1	1	0	0
287205	1	48	12	0	0	1	0	0
294711	1	56	12	0	0	0	0	0
289766	2	50	12	0	0	0	0	0
289793	1	44	12	0	0	0	1	0
289807	1	46	12	0	0	0	0	1
362761	1	54	8.43	1	1	1	0	1
372003	1	51	12	0	1	0	1	0
383040	1	62	12	0	1	1	0	0
289941	1	66	3.5	1	1	1	0	0
290165	1	46	12	0	1	1	0	0
292518	1	36	12	0	1	0	0	0
292532	2	58	2.4	1	0	0	0	1
430859	1	81	12	0	0	0	0	0
383327	2	57	12	0	0	0	0	0
390715	2	58	12	0	0	0	1	1
292569	1	61	12	0	0	0	0	0
365365	1	56	12	0	0	0	0	0
292950	1	43	12	0	1	1	0	0
399272	1	61	12	0	0	0	0	0
293887	2	54	12	0	0	0	0	0
326570	1	65	12	0	0	0	1	1
293173	1	57	12	0	0	0	1	1
293995	1	61	12	0	1	1	0	0
382831	2	72	12	0	0	0	0	0
456128	2	63	12	0	0	0	0	1
294115	1	54	12	0	0	1	0	1
332431	1	38	3.93	1	1	1	1	0
297071	1	52	12	0	0	0	0	0

297095	1	63	12	0	0	0	0	1
388854	1		12	0	0	0	0	0
360982	2	63	12	0	0	0	0	0
294869	1	61	12	0	1	1	0	1
461650	2		12	0	0	0	0	1
297163	1	42	12	0	1	0	0	0
378010	1	51	6.53	1	1	1	0	1
297283	1	39	12	0	0	1	0	0
297322	1		12	0	1	1	0	0
297355	2	65	12	0	0	0	1	1
303945	1	41	12	0	1	0	0	0
297411	1	63	12	0	1	1	1	0
297575	1	54	12	0	0	0	0	1
303990	2	48	12	0	0	0	0	1
297931	1	58	8.03	1	0	1	0	0
297967	1	48	12	0	0	0	0	0
441336	1	45	6	1	0	1	0	0
304298	1	57	12	0	1	1	0	0
304354	1	67	12	0	1	1	0	1
407147	1	63	12	0	0	0	0	1
304597	1	58	12	0	1	1	0	0
304681	1	59	11.9	1	1	1	0	0
304989	1	52	12	0	0	0	0	0
305175	1	48	3.67	1	0	1	0	0
305372	1	58	12	0	1	1	0	1
305414	1	46	12	0	0	0	0	0
411036	1	55	12	0	1	1	0	0
305512	1	55	6	1	1	1	0	0
305821	1	56	12	0	1	1	0	0
430229	1	57	12	0	1	1	0	0
305972	1	70	12	0	0	0	1	0
371756	1	66	12	0	1	1	1	0
306292	1	72	12	0	0	0	0	0
309471	1	43	12	0	1	1	0	0
309496	2	46	12	0	1	1	0	0
416124	2	55	12	0	0	0	0	0
309560	2	56	12	0	0	0	0	0
309573	1	53	12	0	1	1	0	0
312971	1	58	12	0	0	0	0	1
309798	1	51	12	0	1	1	0	1
361183	1	60	12	0	1	0	0	0
309922	1	56	1.1	1	1	1	0	1
309966	1	56	12	0	1	1	0	1
310089	1	45	2.8	1	1	1	0	0

310124	1	72	12	0	0	0	0	0
310126	2	53	12	0	0	0	0	0
368819	2	53	12	0	0	0	0	0
313118	1	55	12	0	0	0	0	0
313161	1	49	12	0	1	1	0	0
314579	1	48	12	0	0	0	1	0
313330	1	46	12	0	1	0	0	0
324497	1	53	12	0	0	0	0	1
315028	2	54	12	0	0	0	0	0
313620	1	54	12	0	0	0	0	0
313634	1	59	8.07	1	1	1	0	1
313667	2	76	12	0	0	0	0	0
315487	2	60	12	0	0	0	0	0
314987	2	58	12	0	0	0	0	0
368793	1	42	12	0	1	1	1	0
318836	1	51	12	0	0	0	0	1
315193	1	53	3.43	1	1	0	0	1
315428	1	60	12	0	1	1	0	0
318888	2	67	12	0	0	0	1	0
318919	1	48	12	0	1	1	0	0
318926	1	74	12	0	1	0	0	0
318933	1	48	12	0	0	1	0	0
318970	1	50	12	0	1	0	0	0
319049	1	33	12	0	0	0	0	0
322544	1	58	2.4	1	0	0	0	1
344757	1	53	12	0	0	1	1	0
319319	2	52	12	0	0	0	0	0
319344	1	74	12	0		0	0	0
319410	1	60	12	0	1	1	0	0
319420	1	68	12	0	0	0	0	1
425715	1	50	12	0	0	0	0	0
322728	1	46	12	0	0	0	0	0
322731	1	49	12	0	1	1	0	0
322953	2	70	12	0	0	0	0	0
323013	1	53	12	0	1	1	0	0
323123	1	60	12	0	1	0	0	0
323143	2	68	7.3	1	0	0	0	1
323198	1	41	12	0	1	1	0	0
323242	1	48	12	0	1	1	0	0
323251	1	56	3.1	1	0	1	0	0
445949	2	54	12	0	0	0	0	0
352851	1	63	12	0		0	0	1
324629	1	60	5.53	1	1	1	0	0
328259	2	71	12	0	0	0	0	1

413655	1	57	12	0	1	1	0	0
324935	1	54	12	0	0	1	0	1
325137	1	58	12	0	1	0	0	0
325338	1	50	12	0	1	1	0	0
326148	1	57	3.03	1	1	1	0	0
338297	2	73	12	0	0	0	0	0
324954	2	61	12	0	0	0	1	0
438569	2	54	9.47	1	0	0	0	1
327199	1	54	12	0	1	0	0	0
326754	2	62	1.4	1	0	0	0	0
325493	1	48	12	0	1	1	0	0
327960	2	60	12	0	0	0	0	1
327207	1	46	12	0	0	1	0	1
327068	1	63	12	0	1	1	0	1
353032	1	43	12	0	1	1	0	0
327677	1	49	5.7	1	1	1	0	1
328715	2	55	12	0	0	0	0	0
374219	1	46	12	0	0	0	0	0
327846	1	30	3.7	1	1	1	0	0
328517	1	38	12	0	0	0	0	0
328452	1	48	12	0	1	1	0	0
330090	2	72	12	0	1	0	0	0
336864	1	52	12	0	0	1	0	0
330213	1	50	12	0	1	0	1	0
354125	1	64	12	0	1	1	0	0
436137	1	79	2.33	1	1	0	0	1
330461	1	61	12	0	1	1	0	1
330740	1	62	12	0	1	1	0	0
330774	1	52	12	0	1	1	0	0
330848	1	66	12	0	0	0	0	0
331661	1	55	12	0	1	1	0	0
344924	1	59	12	0	1	0	0	0
331889	1	32	12	0	1	0	0	0
333354	1	55	12	0	1	0	0	0
332944	1	44	12	0	0	1	0	0
331085	1	37	12	0	1	0	0	0
331104	1	78	1.07	1	0	0	0	1
332864	1	64	12	0	0	0	0	0
333366	1	51	12	0	1	1	0	0
339097	1	59	12	0	0	0	1	1
332821	1	54	12	0	0	0	0	0
332674	2	61	12	0	0	0	0	0
333655	1	59	12	0	1	0	0	0
332702	1	55	12	0		1	0	0

336151	1	45	11.1	1	1	1	0	0
334074	1	49	5.07	1	1	0	0	0
333715	1	40	12	0	0	0	0	0
333855	1	61	12	0	0	0	0	0
412701	1	51	12	0	1	1	0	0
412429	1	61	12	0	1	1	0	0
397307	1	54	12	0	1	0	0	0
338513	1	56	0.9	1	0	0	0	0
334178	2	61	12	0	0	0	1	0
334917	1	55	4.6	1	1	0	0	1
344996	2	62	12	0	0	0	0	0
385567	1	67	12	0	1	0	0	1
348749	1	62	12	0	0	0	0	0
334862	1	60	12	0	1	1	0	0
335515	1	71	12	0	0	0	0	0
334723	1	47	12	0	0	0	0	0
334821	1	44	12	0	0	0	0	0
372990	1	53	12	0	1	1	0	0
367938	1	54	12	0	0	0	0	0
457199	1	70	2	1	0	0	0	0
359112	1	65	12	0	1	1	0	0
386658	1	54	12	0	1	1	0	1
335859	1	59	8.97	1	0	0	0	0
364177	1	53	12	0	0	1	0	1
337957	1	41	12	0	1	1	0	0
337901	2	57	12	0	0	0	0	0
412443	2	52	12	0	0	0	0	0
338507	1	57	12	0	1	0	0	0
338744	2	59	12	0	0	0	0	0
372998	1	55	12	0	0	1	0	1
338993	2	62	12	0	0	0	0	0
359152	1	46	12	0	0	1	0	0
338859	1	49	12	0	1	0	0	0
347081	1	56	12	0	0	0	0	0
339114	2	52	12	0	0	0	0	0
340747	2	59	12	0	0	0	0	0
339970	1	50	12	0	1	1	0	0
340816	1	42	12	0	0	0	0	0
341245	2	49	12	0	1	0	0	0
372016	1	61	12	0	0	0	0	0
340637	1	65	12	0	1	1	0	0
341578	1	65	12	0	0	0	0	0
340713	1	58	12	0	1	0	0	0
345339	1	46	12	0	1	1	0	0

345340	1	47	12	0	1	1	1	0
345368	1	49	12	0	1	1	0	0
456898	1	45	12	0	1	0	0	0
345464	1	56	12	0	1	1	1	0
345925	1	45	12	0	0	1	0	0
369886	1	55	12	0	0	1	0	0
346306	1	55	12	0	1	1	1	0
372405	1	52	12	0	1	1	0	0
404264	1	59	12	0	0	0	0	0
347146	1	48	12	0	0	0	0	0
347846	1	36	12	0	0	0	0	0
348304	1	57	12	0	1	1	1	0
349339	1	55	12	0	1	1	1	1
347084	1	70	12	0	0	0	0	0
384440	1	55	12	0	1	1	0	1
349007	1	71	12	0	1	0	1	0
348439	1	80	12	0	0	0	0	0
377005	1	65	10.67	1	1	1	0	0
359287	1	52	12	0	0	0	0	0
366720	1	57	12	0	0	0	1	0
349797	2	41	12	0	0	0	0	0
350101	2	56	12	0	0	0	0	0
349785	1	49	12	0	0	0	0	0
351122	1	57	10.87	1	1	1	0	0
350975	1	51	12	0	1	1	0	0
350120	2	50	12	0	0	0	0	1
350608	1	51	12	0	0	0	0	0
390292	1	77	12	0	0	0	1	0
350405	1	55	12	0	0	1	0	0
350717	1	48	6.47	1	1	1	0	0
350897	1	58	12	0	0	1	0	1
406933	1	54	12	0	1	0	1	0
352032	1	42	1.73	1	0	0	0	0
410332	1	75	12	0	0	0	0	0
352447	2	65	12	0	0	0	0	0
352194	2	50	12	0	0	0	0	0
379689	1	46	12	0	1	1	0	0
353759	1	52	12	0	1	1	0	0
352514	1	53	12	0	1	1	0	0
353069	1	47	12	0	0	0	0	0
352980	1		12	0	0	0	0	0
352907	1	46	12	0	0	1	0	0
363323	1	30	12	0	1	0	0	0
359442	2	53	12	0	0	0	0	0

359443	1	56	12	0	1	1	0	0
359464	1	58	12	0	1	1	0	0
394917	1	54	12	0	0	0	0	0
359529	1	52	1.7	1	0	0	0	1
412773	1	35	6.93	1	0	0	0	0
359690	1	58	12	0	1	1	0	0
359827	1	49	12	0	0	0	0	0
379386	1	59	12	0	1	1	0	0
360124	1	62	12	0	0	0	0	0
360144	1	49	12	0	0	1	0	0
361622	1	39	12	0	0	0	0	0
361624	1	53	12	0	1	1	0	0
360370	1	72	1.67	1	1	1	0	0
440062	1	37	12	0	0	0	0	0
361823	1	52	12	0	1	1	0	0
362691	1	63	12	0	0	0	1	0
361933	1	55	5.5	1	1	1	0	0
362346	1	56	12	0	1	1	0	0
362992	2	54	12	0	0	0	0	0
362754	1	54	12	0	1	0	0	0
362860	1	60	12	0	0	0	0	0
363679	1	56	12	0	1	1	0	1
366540	2	54	12	0	0	0	0	1
364744	1	47	12	0	0	0	0	0
364204	1	35	0.67	1	0	0	0	0
364750	1	45	12	0	1	1	0	0
365228	1	63	12	0	1	1	0	0
411155	1	52	12	0	1	1	0	0
389690	1	53	12	0	0	0	0	0
365538	1	66	12	0	1	1	0	1
375281	1	42	12	0	1	1	0	0
366445	2	51	12	0	0	0	0	0
458810	1	67	12	0	0	1	0	0
366255	2	59	12	0	0	0	0	0
367218	1	58	12	0	0	0	0	0
366759	1	42	12	0	1	1	1	0
368374	1	42	12	0	1	0	0	0
367589	1	45	12	0	1	1	0	0
368800	1	59	12	0	0	0	0	1
368378	1	50	12	0	1	1	0	0
367900	2	65	12	0	0	0	0	0
369181	1	30	12	0	0	1	0	0
416200	2	61	12	0	0	0	0	0
368583	1	58	12	0	1	0	0	0

370886	1	56	12	0	1	0	0	0
369577	1	34	12	0	1	1	0	0
372702	1	63	12	0	0	0	0	1
371034	1	53	12	0	1	1	0	1
370794	1	59	12	0	0	1	0	0
370880	1	59	12	0	1	1	0	1
371500	1	73	12	0	1	0	0	0
441607	1	56	12	0	1	1	0	0
397562	1	50	12	0	1	1	0	0
372206	1	41	12	0	0	0	0	0
375787	1	56	12	0	1	1	0	0
373104	1	47	12	0	1	1	0	0
373087	1	55	12	0	1	1	0	0
380347	2	52	12	0	0	0	1	0
373070	1	57	12	0	1	1	0	0
374183	1	53	12	0	1	1	0	0
373012	1	59	12	0	1	1	0	0
374403	2	56	12	0	0	0	0	0
414301	1	30	2.67	1	0	0	0	0
377467	1	58	12	0	0	0	0	0
405386	2	59	12	0	0	0	0	0
375242	1	47	12	0	1	1	0	0
378618	2	54	12	0	0	0	0	1
376942	1	78	12	0	0	0	0	0
377214	1	67	12	0	0	0	0	0
375679	1	50	12	0	1	1	0	0
455726	1	65	12	0	1	1	0	1
378851	1	56	12	0	1	1	0	0
377001	1	79	12	0	1	0	0	0
377632	1	55	7.5	1	1	1	0	1
378343	1	49	12	0	1	1	1	0
377738	1	52	12	0	0	0	0	0
378453	2	63	12	0	0	0	0	0
378567	1	56	12	0	0	0	1	0
378549	1		12	0	1	1	0	0
398576	2	66	12	0	0	0	0	0
379885	1	59	12	0	1	0	0	0
380033	1	59	12	0	0	0	0	0
380040	1	55	12	0	1	1	0	1
385276	1	38	12	0	1	0	0	0
380071	1	49	12	0	1	1	0	1
381117	2	62	12	0	0	0	0	0
381581	1	74	1.7	1	1	1	0	0
381471	1	36	12	0	1	1	0	0

381144	1	65	12	0	0	0	0	0
381054	2	63	12	0	0	0	0	0
381987	1	61	12	0	0	0	0	1
382896	1		12	0	0	0	1	0
382184	1	56	12	0	1	1	0	0
381748	2	65	12	0	0	0	0	0
382254	1	71	4.97	1	0	1	0	0
383479	1	59	12	0	0	0	0	0
383306	1	41	12	0	1	1	1	1
384580	1	52	12	0	0	0	0	0
384204	1	57	12	0	1	1	0	0
385024	1	59	12	0	1	0	0	0
386299	1	61	12	0	0	0	0	0
543414	1	52	11.53	1	0	1	0	0
385449	1	49	3.23	1	1	1	1	0
386667	1	28	12	0	0	1	0	0
386151	2	59	12	0	0	0	0	0
386383	1	52	12	0	1	1	0	0
387648	1	46	12	0	0	0	0	0
387186	1	66	12	0	1	1	0	0
387183	1	51	12	0	0	0	0	0
387846	2	60	12	0	0	0	0	0
415462	1	48	12	0	1	1	0	1
388182	1	39	12	0	0	1	0	0
387061	1	50	12	0	0	1	0	0
439357	2	56	12	0	0	0	1	0
388002	2	59	12	0	0	0	0	0
387964	1	39	12	0	0	1	0	0
387621	1	54	12	0	0	0	0	0
388652	2	52	12	0	0	0	0	0
388579	1	35	12	0	1	1	0	0
387725	1	60	12	0	1	1	0	1
388702	1	67	12	0	0	0	0	0
389614	1	50	12	0	1	1	0	0
389257	2	60	12	0	0	0	0	0
390214	2	63	12	0	0	0	0	0
390135	1	34	8.17	1	0	0	0	0
391380	1	52	12	0	0	0	0	0
390546	1	57	12	0	1	0	0	0
395413	1	44	12	0		0	0	0
390770	1	49	12	0	1	1	0	0
401860	2	61	12	0	0	0	0	1
395430	1	43	12	0	0	1	0	0
391146	2	67	12	0	0	0	0	0

395514	1	59	7.63	1	0	0	0	1
391641	1	49	12	0	0	1	0	0
395840	1	36	12	0	1	0	0	0
395974	1	54	12	0	1	1	0	1
396972	1	63	12	0	1	1	0	1
396312	1	59	12	0	0	0	0	0
396962	1	60	12	0	1	1	0	0
397829	1	456	12	0	1	1	0	0
398071	1	68	12	0	0	1	0	0
397991	1	51	12	0	0	0	0	0
434969	1	55	12	0	0	1	0	0
399269	2	53	12	0	0	0	0	0
398867	1	71	12	0	1	1	0	1
452732	1	47	3.3	1	1	1	0	0
400652	1	50	12	0	0	0	0	0
399875	1	52	12	0	1	0	0	0
446599	2		12	0	0	0	0	0
399344	1	67	12	0	0	0	0	0
400411	1	57	2.23	1	1	1	0	1
401716	1	64	12	0	0	1	0	0
401223	1	73	12	0	0	0	0	0
400187	1	56	12	0	1	1	0	1
402133	1	59	12	0	0	0	0	1
454137	1	70	12	0	1	1	0	0
401290	2	52	12	0	0	0	0	0
402775	1	45	12	0	1	0	0	0
401206	1	70	12	0	0	1	0	1
401501	2	51	12	0	0	0	0	0
401960	1	50	12	0	1	1	0	1
402883	1	45	12	0	1	0	0	0
414572	1	65	12	0	1	1	0	1
421033	2	66	12	0	0	0	0	0
412508	2	76	11.63	1	0	0	0	0
404267	2	49	12	0	0	0	0	0
404893	2	63	12	0	0	0	0	0
404277	1	75	12	0	1	1	0	0
404039	1	46	12	0	0	0	0	0
404534	1	67	12	0	0	0	0	1
406372	1	60	12	0	0	0	0	0
406017	1	47	12	0	1	0	0	0
404443	1	59	12	0	1	1	0	0
405045	1	39	12	0	1	1	0	0
404929	2	48	12	0	0	0	0	0
404510	1	59	12	0	0	0	1	0

408879	1	40	12	0	0	1	0	1
405991	1	71	12	0	1	1	1	1
405637	1	66	12	0	1	1	0	0
406121	1	50	12	0	1	1	0	0
405842	2	64	12	0	0	0	1	0
407132	2	57	12	0	0	0	0	0
406975	2	46	12	0	0	0	1	0
408005	1	43	12	0	1	1	0	0
406282	2	58	12	0	0	0	0	0
406666	1	57	12	0	0	0	0	0
407589	1	57	12	0	1	1	0	0
408382	1	52	12	0	1	1	0	0
408653	2	68	12	0	0	0	0	0
440352	2	55	12	0	0	0	0	0
409228	1	72	12	0	1	0	0	0
410327	2	51	12	0	0	0	0	0
412309	2	31	12	0	0	0	0	0
410919	2	74	12	0	0	0	0	0
411653	1	59	12	0	1	1	0	1
411176	1	46	3.23	1	1	1	0	0
410939	1	54	12	0	0	0	1	0
413247	1	53	12	0	1	1	1	1
412011	1	73	12	0	1	1	0	0
411217	1	51	12	0	1	0	0	0
413772	1	39	12	0	0	0	1	0
412832	1	49	12	0	1	0	0	0
413218	1	45	12	0	0	0	0	0
414021	2	63	12	0	0	0	0	0
413184	1	49	12	0	1	1	0	0
412886	2	60	12	0	0	0	0	0
413029	1	57	12	0	1	0	1	0
413061	1	54	12	0	0	1	0	0
413482	1	30	12	0	1	1	0	0
414173	1	60	12	0	0	0	0	0
415078	1	56	12	0	1	1	0	0
415709	1	45	12	0	0	0	0	0
416048	1	57	12	0	1	1	0	0
414051	2	38	12	0	0	0	0	0
415974	1	59	12	0	1	1	0	0
443457	2	66	12	0	0	0	0	0
415477	2	52	12	0	0	0	0	0
415071	1	46	12	0	1	0	0	0
417258	1	59	12	0	1	1	0	0
414893	2	53	12	0	0	0	1	0

416624	1	66	1.9	1	0	1	0	1
416420	1	46	12	0	0	0	1	0
416431	1	68	12	0	1	1	0	0
417109	1	43	12	0	1	1	0	0
417282	1	59	12	0	1	1	0	0
417037	1	53	12	0	0	0	0	0
431768	1		12	0	1	1	0	0
418115	2	52	12	0	0	1	0	0
427931	1	65	12	0	1	1	0	0
418082	1	48	12	0	1	0	0	0
417326	1	58	12	0	0	0	0	0
419230	2	54	3.77	1	0	1	0	0
418581	2	60	12	0	0	0	0	0
423824	1	61	12	0	1	1	0	0
419845	2	55	12	0	0	0	0	0
419683	1	61	12	0	0	0	0	0
419526	2	49	12	0	0	0	0	0
419572	1	54	12	0	1	1	0	0
419994	2	58	12	0	0	0	0	0
420016	1	62	12	0	0	0	1	0
420307	1	62	12	0	1	0	0	0
420611	1	61	12	0	1	0	1	0
431397	1	37	12	0	0	1	0	0
421830	2	58	12	0	0	0	0	0
421842	2	60	12	0	0	0	0	0
423196	2	72	12	0	0	0	0	1
423432	2	54	12	0	0	0	0	0
422396	1	66	12	0	0	0	0	0
422242	1	50	12	0	1	1	0	1
429698	1	76	12	0	1	1	1	1
422796	1	59	12	0	0	0	0	0
424297	2	51	12	0	0	0	1	1
424450	2	77	12	0	0	0	0	0
424330	1	60	12	0	1	1	0	1
424546	1	68	12	0	1	0	0	1
425987	1	52	12	0	1	1	0	0
425114	1	70	12	0	1	1	0	1
425246	1	61	1.13	1	1	1	0	0
426672	2	61	12	0	0	0	0	0
425584	2	55	12	0	1	1	0	0
427798	1	51	12	0	1	0	0	1
426655	1	42	12	0	1	1	0	1
427323	1	53	12	0	1	1	0	0
427056	1	73	12	0	0	1	0	0

426140	1	47	12	0	1	0	0	0
428469	1	53	12	0	1	0	0	0
427744	1	61	12	0	1	1	0	0
427866	1	65	12	0	1	1	0	0
427187	1	62	12	0	1	1	0	1
429118	1	50	12	0	0	1	0	1
429417	2	76	12	0	0	0	1	0
429282	2	58	12	0	0	0	1	0
429813	1	41	12	0	0	1	0	0
430253	1	65	12	0	0	0	0	0
429498	1	60	12	0	1	1	0	0
430836	1	52	12	0	1	0	1	0
430978	1	64	12	0	0	1	0	1
431179	1	59	12	0	1	0	0	1
432150	1	61	12	0	1	0	0	0
431569	1	24	12	0	0	0	0	0
432158	1	49	12	0	1	1	0	0
431715	1		12	0	1	0	0	0
431815	1	47	1.63	1	1	1	0	0
432347	1	60	11.73	1	1	1	0	0
435648	1	55	11.27	1	0	1	0	1
434604	1	52	12	0	1	0	0	1
435750	2	50	12	0	0	0	0	0
435882	2	55	12	0	0	0	0	0
437445	1	62	12	0	0	1	0	1
436543	2	66	12	0	0	0	1	1
437227	1	59	12	0	1	1	0	1
438474	1	44	12	0	0	0	0	0
438444	1	59	12	0	0	0	0	0
438448	1	52	12	0	1	0	0	1
439263	1	28	12	0	0	0	0	0
440349	1	65	12	0	0	1	1	0
440893	1	55	12	0	1	1	0	1
443393	2	48	12	0	0	0	0	0
441512	1	59	12	0	0	0	0	0
441250	1	42	12	0	0	0	0	0
442537	1	68	12	0	0	1	0	0
442019	1	48	12	0	1	1	0	0
443820	1	46	12	0	1	1	0	0
443001	2		12	0	0	0	0	0
443761	1	59	12	0	0	0	0	0
444950	1	60	12	0	1	1	0	1
444262	1	61	12	0	1	1	0	0
445322	1	59	12	0	1	1	0	0

446132	1	47	12	0	0	1	0	0
445751	1	43	12	0	0	0	1	0
446000	1		12	0	1	0	0	0
447840	1	61	6.4	1	0	0	0	1
447635	1	59	12	0	0	0	0	0
447900	1	64	12	0	1	1	0	0
447875	1	39	12	0	0	1	0	0
448747	1	60	12	0	0	0	0	0
450416	1	19	12	0	0	0	0	0
449880	2	54	12	0	1	1	0	0
449415	2	60	12	0	0	1	0	0
449495	1	62	12	0	1	1	0	0
450255	1	51	12	0	1	1	0	0
451098	1	59	12	0	1	0	1	0
451391	1	58	12	0	0	1	0	0
451941	1	53	12	0	1	1	0	0
452052	1	59	12	0	0	0	0	0
453855	2	24	12	0	0	0	0	0
452824	1	63	12	0	1	1	0	0
453498	1	55	12	0	1	0	0	0
453816	1	48	12	0	1	1	0	0
454507	1	62	12	0	1	1	0	0
454256	1	58	12	0	1	1	0	0
455954	1	57	12	0	0	0	0	0
455210	1	63	12	0	1	0	0	1
455965	1	68	4.33	1	0	0	0	0
455970	1	68	12	0	0	0	0	1
456118	1	47	12	0	1	1	0	0
456753	1	61	12	0	1	1	0	0
456700	2	67	12	0	0	0	0	1
461653	1	65	8.57	1	1	1	1	0
252793	1	53	12	0	1	1	0	0
462969	1	55	3.63	1	1	1	0	1
469175	1	60	12	0		0	0	0
477335	2	77	3.17	1	0	0	0	0
519764	1	53	1.2	1	1	1	0	0
522817	1	67	12	0	1	1	0	1
549622	1	61	9.83	1	1	1	0	0
559097	2	62	7.83	1	0	0	0	1
576228	2	82	0.5	1	0	0	0	0
337424	1	62	12	0	0	0	0	0
346471	1	59	12	0		0	0	0
376244	1	50	12	0	0	1	0	0
520816	1	62	3.7	1	1	1	0	1

254507	2	75	12	0	0	0	0	1
306840	1	58	12	0	0	0	0	0
306841	1	56	12	0	1	1	0	1
255097	1	54	12	0	0	0	0	0
346473	1	47	12	0	0	0	0	0
245047	1	70	12	0	1	0	0	0
459254	1	66	12	0	0	0	0	0
323683	2	73	12	0	0	0	0	1
287718	1	76	12	0	0	1	0	1
325415	2	55	12	0	0	0	0	0
420589	1	63	12	0	1	0	0	0
287722	1	60	12	0	0	0	0	0
261961	1	52	12	0	1	1	0	0
384172	1	70	12	0	1	1	0	0
377704	1	55	7.33	1	1	0	0	0
418762	1	64	12	0	1	1	0	0
242338	2	73	12	0	0	0	0	0
284723	2	53	12	0	0	0	0	0
449216	1	60	12	0	1	1	0	0
427545	1	59	12	0	0	0	0	0
293330	2	52	12	0	0	0	0	0
246048	1	42	12	0	1	0	0	0
269022	1	68	12	0	0	0	0	1
264746	1	59	12	0	1	0	0	0
247606	1	74	12	0	0	0	1	1
252906	1	54	5.67	1	0	1	0	0
346478	2	67	12	0	0	0	0	0
287732	2	73	12	0	0	0	0	1
275504	2		5.6	1	0	0	0	0
251821	2	41	12	0	0	1	0	0
252288	1	58	12	0	1	0	0	0
250438	2	50	12	0	0	0	0	0
252796	1	45	12	0	1	1	0	0
381260	1	61	12	0	0	0	0	0
251608	2	63	12	0				0
223606	1	63	5.67	1	0	1	0	0
284730	1	54	12	0	0	0	0	0
255301	1	48	6.47	1	1	0	0	0
428491	1	71	12	0	0	0	0	0
261904	1	59	12	0	1	1	0	0
272815	1	54	12	0	0	0	1	0
365235	1	53	12	0	1	0	0	1
250823	2	59	12	0	0	0	0	0
280616	1	70	12	0	0	0	0	0

368038	1	63	7.13	1	1	1	0	1
350974	2	43	12	0	0	0	0	0
384757	1	58	7.4	1	0	0	0	0
261219	1	53	12	0	1	0	1	1
423775	1	60	9.67	1	1	0	0	0
347756	1	51	12	0	0	0	0	1
412901	1	64	12	0	0	1	0	0
314126	1	51	7.5	1	1	0	0	0
403131	1	50	0.63	1	0	0	0	0
294902	2	52	12	0	0	0	0	0
298083	1	43	12	0	1	1	0	0
251057	1	42	12	0	0	1	0	0
389499	1	55	12	0	1	1	0	0
368646	1	53	12	0	1	1	1	1
373004	1	39	12	0	0	1	0	0
284744	1	50	8.57	1	0	0	0	0
263780	1	55	9.8	1	1	1	0	0
376023	1	40	12	0	0	1	0	0
337497	1	60	12	0	0	1	0	0
371042	2	63	12	0	0	0	0	0
264835	1	74	12	0	1	1	0	0
340700	1	82	12	0	0	0	0	0
332396	1	59	12	0	0	1	0	0
339624	1	49	5.47	1	0	0	0	0
406120	1	68	12	0	1	1	0	0
437302	1	45	12	0	1	0	1	0
400527	1	56	12	0	0	0	0	0
400387	1	62	9.17	1	1	1	0	0
306960	1	41	9.73	1	1	1	0	0
252650	1	49	12	0	1	1	0	1
246887	2	57	12	0	0	0	0	0
382690	1	47	12	0	0	0	0	1
398754	1	44	12	0	0	0	0	0
349035	1	56	12	0	0	0	0	1
348257	1	71	12	0	0	0	0	0
269052	2	49	5.43	1	0	0	0	0
434535	1	64	12	0	1	1	0	1
418907	1	53	12	0	1	1	0	1
351692	1	51	12	0	0	0	0	0
365809	1	39	12	0	0	0	0	0
415466	1	53	12	0	1	1	0	0
253813	2	58	12	0	0	0	0	0
269700	1	50	12	0	0	1	0	0
242930	2	49	12	0	0	0	0	0

265615	1	56	12	0	0	1	0	1
432363	1	55	12	0	1	1	0	1
270149	1		12	0	1	0	0	0
307012	2	54	12	0	0	0	0	0
346527	1	47	12	0	0	1	0	0
336746	1	46	12	0	0	0	0	0
426286	2	58	12	0	0	0	0	1
307030	1	41	12	0	0	0	0	1
386270	1	45	12	0	0	0	0	0
253644	1	31	12	0	0	0	0	0
244044	1	57	1.57	1	0	1	0	0
389250	1	44	12	0	0	0	0	0
396577	1	66	12	0	0	0	0	1
366464	1	75	12	0	0	0	0	0
237455	1	69	12	0	0	0	0	0
272887	1	50	12	0	1	1	1	0
433627	1	54	12	0	1	1	1	0
270064	1	60	12	0	0	0	0	0
245361	1	54	12	0	0	0	0	1
265405	2	57	12	0	0	0	0	0
408717	1	63	12	0	0	0	0	0
384746	1	60	12	0	1	1	0	0
366542	1	59	12	0	1	0	0	1
234998	2	61	12	0	0	0	0	0
314532	1	45	12	0	0	0	0	0
349574	1	50	12	0	1	0	0	0
425002	2	60	12	0	0	0	0	0
266021	1	58	12	0	1	1	0	0
246106	2	72	12	0	0	0	0	1
280498	1	69	12	0	1	1	0	0
264269	1	50	12	0	1	0	1	0
334776	1	71	12	0	0	0	0	1
378611	1	60	12	0	1	1	0	0
348631	1	59	12	0	0	1	0	0
397605	1	60	11.2	1	1	1	0	0
265359	1	46	12	0	1	0	0	0
320090	1	70	12	0	1	1	0	0
360714	1	67	12	0	0	0	0	0
367439	1	56	12	0	1	1	0	0
262582	1	52	12	0	0	0	0	0
326694	2		12	0	0	1	1	0
246222	1	69	12	0	1	1	0	1
456567	1	61	12	0	0	0	0	0
263703	1	64	12	0	1	1	0	1

253642	1	56	12	0	1	0	0	0
242573	1	62	12	0	1	1	0	0
303373	1	58	12	0	1	1	0	1
319971	2	70	12	0	0	0	0	0
346476	1	61	12	0	1	1	0	0
259683	1	55	10.73	1	0	0	0	1
327544	1	56	12	0	1	1	0	1
325399	1	38	12	0	0	0	0	1
352566	1	54	9.3	1	0	0	0	0
339705	1	62	12	0	1	1	0	0
237119	1	43	3.13	1	0	0	0	0
332243	1	49	4	1	0	0	0	0
359232	1	58	7.9	1	0	0	0	0
275764	1	48	12	0	1	1	0	0
283618	2	64	4	1	0	0	0	1
309131	1	60	12	0	1	1	0	1
340285	1	50	2.67	1	1	1	1	1
335613	1	54	10.3	1	0	0	0	0
327384	1	53	12	0	0	0	0	0
368714	1	44	12	0	1	1	0	1
453886	2	54	5.57	1	0	0	0	0
273613	2	78	12	0	0	0	0	1
265625	1	51	12	0	1	1	0	0
418743	1	53	12	0	0	0	0	1
462377	1	86	8.43	1	1	1	0	0
397648	1	66	12	0	0	0	0	0
390599	2	60	12	0	0	0	0	0
380054	1	32	3.9	1	0	0	0	0
458787	1	54	12	0	0	0	0	0
398188	2	64	12	0	0	0	0	0
404361	2	70	12	0	0	0	0	0
404222	1	59	0.93	1	0	0	0	0
406362	1	66	12	0	1	0	0	0
407251	1	66	12	0	0	1	0	0
408381	1	52	6.4	1	1	1	0	0
421816	1	63	12	0	1	0	0	0
424468	2	64	4.43	1	0	0	0	0
432815	1	53	4.63	1	0	0	0	0
436703	1	53	12	0	1	1	0	0
438099	1	34	12	0	1	1	0	0
445341	1	64	1.5	1	0	0	0	0
451666	1	56	12	0	1	1	0	1
455241	2	85	1.13	1	0	0	0	1
513150	1	64	0.67	1	0	1	0	0

360634	1	72	12	0	1	1	0	0
272800	2	59	12	0	0	0	1	1
360649	2	82	12	0	1	0	1	0
415108	2	59	12	0	0	0	0	0
293452	1	56	8.93	1	1	0	1	0
303680	1	55	3.47	1	1	1	0	0
306102	2	60	12	0	0	0	0	0
348080	1	56	12	0	1	1	0	0
328513	1	47	2.8	1	1	1	0	0
354238	1	63	12	0	0	0	0	1
360010	1	51	1.33	1	0	1	0	0
379958	1	58	1.4	1	0	0	0	0
248425	1	50	10.03	1	0	0	0	1
283338	1	78	12	0	0	0	0	1
248553	1	55	3.7	1	1	1	0	1
255812	1	72	2	1	1	1	0	0
336114	1	46	12	0	0	0	0	1
318918	2	67	5.97	1	0	0	0	1
365091	1	45	12	0	1	0	1	0
376370	2	63	2.27	1	0	0	0	0
524816	1	61	0.8	1	1	1	0	1
364924	1	58	12	0	1	1	0	1
269297	1	66	4.57	1	1	0	0	1
383865	1	44	1.57	1	0	0	0	0
293428	1	54	12	0	0	0	0	1
224714	2	54	1.53	1	0	0	0	0
222844	1	61	0.23	1	1	1	1	1
223341	1	66	0.93	1	1	1	0	1
227438	2	66	1.93	1	0	0	0	0
234892	1	45	1.33	1	1	1	0	0
234680	1	66	0.63	1	0	0	0	0
234562	1	43	1.03	1	1	1	0	0
234731	1	59	0.57	1	0	1	0	0
237349	1	60	1.87	1	1	1	0	0
236947	1	37	12	0	0	1	0	0
237134	1	58	0.83	1	1	1	0	0
275482	1	58	12	0	0	1	0	1
273135	1	57	1.2	1	1	0	0	0
275679	2	73	7.7	1	0	0	0	0
275741	1	40	1.27	1	1	0	1	0
273433	1	60	0.33	1	1	1	0	0
273115	1	57	1.6	1	1	1	0	1
275468	1	67	12	0	1	0	0	0
273384	2	70	0.6	1	0	0	0	0

275915	2	49	12	0	0	1	0	0
275875	1	49	12	0	1	0	0	0
273119	2	75	2	1	0	0	0	0
275473	2	56	9.93	1	0	0	0	0
273182	1	40	1.87	1	0	0	0	0
275442	1	59	12	0	0	0	0	0
273844	1	59	0.67	1	1	1	0	0
273175	1	60	5.53	1	0	0	0	0
273836	2	51	2.8	1	0	0	0	1
273965	2	58	12	0	0	0	1	0
275824	1	49	11.3	1	1	0	1	1
274001	1	65	1.17	1	0	0	0	1
275549	1	87	8.27	1	0	0	0	0
275676	2	78	12	0	0	0	1	0
274944	1	67	0.53	1	0	0	0	0
275593	1	66	12	0	1	1	0	1
275281	1	68	0.47	1	0	0	0	0
242811	1	44	2.2	1	1	1	0	0
242957	1	59	12	0	1	0	1	1
242815	2	46	0.47	1	0	0	0	0
243798	1	50	4.83	1	1	0	0	0
244208	1	72	12	0	0	0	0	0
243753	1	63	2.07	1	1	1	0	1
244324	1	50	12	0	1	1	0	1
244341	1	50	10.8	1	1	1	0	0
244631	1	37	12	0	0	0	0	0
244913	1	62	2.1	1	0	1	0	0
244944	1	67	12	0	0	0	0	0
245845	1	65	0.47	1	0	0	0	0
245952	1	51	0.33	1	1	0	0	0
246077	1	52	4.4	1	1	0	0	0
246440	1	39	0.37	1	1	1	1	0
246524	1	49	1.4	1	0	1	0	0
246600	2	45	12	0	0	0	1	0
246786	1	47	12	0	0	0	0	0
246835	1	53	3.07	1	1	1	0	0
246850	2	68	1.4	1	0	0	0	0
247215	1	45	4.53	1	1	1	0	0
247247	1	50	3.9	1	1	1	0	0
247386	1	37	0.6	1	0	0	0	0
247495	1	82	1.23	1	0	0	0	0
247865	1	46	5.5	1	0	1	0	0
247955	1	47	9.87	1	0	0	0	0
247986	2	54	1.43	1	0	0	0	0

248025	2	72	2.93	1	1	0	0	1
248064	1	41	7.77	1	1	0	0	0
248194	1	66	12	0	1	0	0	1
248283	1	58	1.37	1	1	1	0	0
248849	1	64	0.27	1	0	0	0	0
248864	1	46	0.97	1	0	0	0	0
248902	1	47	9.1	1	1	1	0	0
248921	1	50	5.67	1	1	1	0	0
249350	2	69	6.07	1	0	0	0	0
249377	2	71	2.8	1	0	0	0	0
249913	1	52	9.63	1	0	0	0	0
249997	1	63	0.27	1	1	1	0	0
250553	2	21	1.2	1	0	0	1	0
250599	1	49	3.5	1	0	1	0	0
250629	1	50	12	0	1	1	0	1
250700	1	52	0.97	1	1	0	0	1
251004	1	53	12	0	1	1	0	0
251070	1	52	0.53	1	0	0	0	0
251224	1	58	1.73	1	0	1	0	0
251409	1	71	12	0	0	1	0	0
251552	1	42	12	0	1	1	0	0
251544	1	67	3.9	1	1	0	0	1
251653	2	70	0.17	1	0	0	0	0
251627	1	52	12	0	1	1	0	0
251740	1	56	12	0	1	1	0	1
251877	2	64	0.8	1	0	0	0	0
252730	1	47	0.8	1	0	0	0	0
252795	1	49	12	0	0	0	0	0
252842	1	49	12	0	1	1	0	0
252955	2	64	0.77	1	0	0	0	1
253034	1	61	12	0	0	1	0	0
253024	2	60	0.4	1	0	0	0	0
253129	1	55	9.1	1	1	1	0	1
253194	1	55	12	0	0	0	0	0
253305	1	44	0.63	1	1	0	1	0
253472	1	63	2.23	1	1	1	0	0
253519	1	56	4.83	1	0	1	1	1
253645	1		12	0	1	1	0	1
253632	1	52	0.3	1	1	1	1	0
253729	1	55	4.63	1	0	1	0	0
253826	1	57	7.3	1	0	1	0	1
254027	2	60	0.6	1	0	0	1	0
254192	1	77	4.93	1	1	0	0	0
254224	1	41	12	0	0	0	1	0

254499	2	67	0.47	1	0	0	0	0
254561	2	69	12	0	0	0	0	0
254650	1	54	12	0	0	0	0	0
254806	1	58	2.43	1	0	0	0	0
254953	2	58	0.2	1	0	0	0	0
255053	1	61	12	0	0	1	0	0
255088	1	65	1.87	1	1	0	0	0
255197	1	52	12	0	1	1	0	0
255316	1	38	0.47	1	1	1	0	0
255508	1	60	2.03	1	1	1	0	0
255547	2	63	3.47	1	0	0	0	0
255606	1	50	11.3	1	1	1	0	1
255730	1	63	5.8	1	1	1	0	0
255896	1	40	0.37	1	1	0	0	0
258697	1	57	3.3	1	0	0	0	0
258921	1	57	0.73	1	0	0	0	0
259001	1	52	12	0	1	1	0	0
259451	2	70	0.33	1	0	0	1	0
260692	1	41	1.33	1	0	0	0	1
260931	1	47	2.47	1	1	1	0	0
261373	1	57	12	0	1	1	1	0
261573	1	58	2.97	1	0	0	0	0
261973	1	41	1.43	1	1	1	0	0
262005	1	63	3.83	1	1	1	0	1
262307	1	53	1.67	1	0	1	0	0
262433	1	61	2.63	1	1	0	0	0
262481	1	57	1.63	1	0	0	0	1
262474	2	47	2.37	1	0	0	0	0
262533	1	58	0.7	1	0	0	0	0
262568	1	57	12	0	0	0	0	0
262734	1		12	0	0	1	0	0
262733	1	51	3.67	1	1	1	0	0
262751	1	46	1.23	1	1	1	0	0
262812	1	57	0.33	1	1	1	0	0
262937	1	50	1.73	1	0	1	0	0
263030	1	56	12	0	1	0	0	1
263152	1	59	12	0	1	1	0	1
263276	1	90	0.4	1	0	0	0	0
263349	1	54	2.77	1	1	0	0	1
263555	1	49	1.07	1	0	0	0	0
263567	1	45	0.73	1	0	0	0	0
263625	1	58	4.13	1	1	1	0	0
264460	2	59	0.57	1	0	0	0	0
264434	1	51	0.47	1	0	1	0	0

264625	1	41	12	0	1	1	0	0
265066	1	50	12	0	1	1	0	0
265072	1	54	4.23	1	1	1	0	0
265057	1	63	2.93	1	1	1	0	0
265224	1	71	1.6	1	1	1	0	1
265286	1	64	2.93	1	0	0	0	0
265403	1	43	12	0	1	1	1	0
265404	2	44	12	0	0	1	0	0
266194	1	62	1.23	1	1	0	0	0
266411	1	56	2.17	1	1	1	0	0
266449	1	54	5.4	1	1	1	0	0
266581	2	72	12	0	0		0	1
266634	2	57	1.3	1	0	0	0	0
267269	2	56	12	0	0	0	0	0
268525	2	68	2.07	1	0	0	0	1
268287	1	57	1.13	1	1	1	0	0
268618	1	55	0.07	1	1	1	0	0
268831	2	57	2.77	1	0	0	0	0
268276	1	78	4.57	1	0	0	0	0
268505	2	59	9.4	1	0	0	0	0
268413	1	56	12	0	0	1	0	0
269010	1	48	8.2	1	0	1	0	0
269194	1	57	12	0	1	1	0	0
269433	1	63	12	0	0	0	0	0
271994	1	66	2.6	1	1	0	1	1
271914	1	50	3	1	1	0	1	0
271956	1	40	0.97	1	1	0	0	0
272025	1	77	12	0	0	1	0	0
272919	1	63	12	0	0	0	0	1
272724	1	65	0.17	1	1	1	0	0
279150	2	61	12	0	1	0	1	1
279606	1	59	12	0	0	0	0	0
279330	1	51	6.3	1	0	1	0	1
279347	1	57	2.73	1	1	1	0	0
279535	1	49	1.5	1	0	1	0	0
279329	2	68	12	0	0	0	0	0
280268	1	57	1.57	1	1	1	0	1
280485	2	54	9.1	1	0	0	0	0
280806	2	57	2.33	1	0	0	1	1
280854	1	45	2.23	1	0	0	0	0
281454	1	54	0.67	1	0	0	1	0
283748	1	63	12	0	1	1	0	0
283796	1	48	1.07	1	0	1	0	0
284736	2	54	12	0	0	0	0	0

284006	1	41	0.87	1	1	1	0	0
284314	1	72	0.57	1	1	0	0	0
286739	1	57	11.3	1	1	1	0	0
286339	2	64	12	0	0	0	0	1
287069	1	46	0.97	1	1	1	0	0
286866	1	52	1.27	1	0	0	0	1
287727	1	48	0.13	1	1	0	0	0
287742	1	87	2.27	1	1	1	0	0
289999	1	57	0.27	1	0	1	0	0
289939	1	62	10.03	1	1	1	0	0
290479	1	85	0.87	1	1	0	0	0
290741	2	54	1.33	1	0	0	0	0
290103	1	63	12	0	0	1	0	0
292145	1	65	1	1	1	1	0	0
292128	1	44	12	0	0	1	0	0
292499	1	45	12	0	0	1	0	0
292747	1	47	0.9	1	1	1	0	0
292560	2	45	12	0	0	1	0	0
293264	1	62	0.3	1	1	1	0	1
293885	2	66	8.47	1	0	0	0	0
293935	1	57	0.53	1	1	0	1	0
294218	1	43	12	0	1	1	0	0
294464	1	58	12	0	1	1	0	0
294536	1	56	6.47	1	1	0	1	0
294883	2	52	12	0	0	0	0	0
297066	1	53	12	0	1	1	0	0
296665	2	43	7.53	1	1	1	0	0
298073	1	52	2.07	1	0	0	0	1
296608	1	40	4.4	1	0	0	1	1
297377	1	60	12	0	0	0	0	0
298174	1	43	1.8	1	1	1	0	0
297049	1	52	12	0	0	0	1	0
297204	1	47	1	1	1	1	0	1
297008	2	77	2.7	1	0	0	0	0
306909	1	39	1.07	1	0	0	0	0
304079	1	53	1.3	1	1	1	0	0
304120	1	76	12	0	0	0	0	1
304024	1	51	1.07	1	1	1	0	0
304344	1	42	2.77	1	0	0	0	0
304731	1	60	0.23	1	0	0	0	0
306885	1	52	12	0	0	0	0	0
303830	1	51	12	0	1	1	0	0
303984	2	77	2.07	1	0	0	0	0
304796	2	59	12	0	0	0	0	0

305279	2	62	0.17	1	0	0	0	0
304256	2	62	1.5	1	0	0	0	0
303998	1	59	12	0	0	1	0	0
302995	1	55	7.67	1	0	1	0	1
305332	1	62	0.5	1	1	1	0	0
305537	1	42	0.63	1	0	1	0	1
305818	1	35	0.37	1	0	0	1	0
304714	1	64	9.57	1	1	1	0	0
305669	1	48	5.73	1	1	1	0	0
305835	2	70	1.33	1	0	0	0	0
306008	1	39	12	0	0	0	0	0
306225	1	69	12	0	1	1	0	0
306899	1	54	12	0	1	1	0	0
308976	2	51	12	0	0	0	0	1
309395	1	59	1.37	1	0	1	0	0
310502	1	48	1.07	1	1	0	0	0
310025	2	39	1.37	1	0	0	0	0
309065	1	42	0.53	1	0	1	0	0
310400	1	53	0.77	1	0	0	0	0
309492	2	73	5.7	1	0	0	0	0
309618	1	75	1.13	1	0	0	0	0
310062	1	55	9.23	1	0	0	0	1
313116	1	68	3.13	1	1	1	0	0
312436	1	61	12	0	1	1	0	0
312475	2	59	12	0	0	0	0	0
312970	2	73	2.23	1	0	0	0	1
313493	1	59	0.37	1	0	0	1	1
313057	1	51	9.43	1	1	1	1	0
313364	1	60	1.97	1	0	0	1	0
313094	1	37	1.37	1	0	0	0	0
314007	1	58	3.6	1	0	0	0	0
314236	2	31	1.57	1	0	0	0	0
314554	1	35	12	1	0	0	0	0
314873	1	69	12	0	1	1	0	1
315396	1	61	12	0	1	1	0	1
315907	1	43	12	0	0	1	0	0
318783	1	58	12	0	0	0	0	1
318824	1	55	12	0	1	1	0	0
318750	1	65	12	0	0	1	0	0
318842	1	51	4.87	1	1	1	0	1
319030	2	56	12	0	0	0	0	0
319064	1	38	12	0	1	1	0	0
319640	1	58	0.43	1	0	1	0	0
319882	2	88	0.1	1	0	0	0	0

318134	1	82	6.03	1	0	0	0	0
318832	1	58	12	0	0	0	0	0
319152	1	76	12	0	0	0	0	0
322470	1	58	12	0	1	1	0	0
322599	1	55	12	0	0	0	0	0
323783	1	56	8.57	1	1	0	0	1
322852	1	65	0.9	1	1	1	0	1
323041	1	58	2.17	1	1	1	0	1
323105	1	48	12	0	0	1	1	0
322717	1	48	3.77	1	1	1	0	1
323843	1	63	12	0	1	1	0	0
324571	1	37	1.27	1	0	0	0	0
325531	1	67	0.6	1	1	1	0	1
325532	1	74	12	0	0	0	0	0
325536	1	47	12	0	1	1	1	0
325757	1	63	1.2	1	1	1	0	0
325954	1	62	6.93	1	1	1	0	0
326159	2	61	12	0	0	0	0	0
326956	1	46	12	0	1	1	0	0
327749	2	80	1.2	1	0	0	0	0
327784	1	46	1.63	1	1	0	0	0
328872	1	52	10.17	1	1	1	0	1
330223	2	71	0.3	1	0	0	0	0
329630	1	54	12	0	1	1	0	1
330072	1	54	5.43	1	1	1	0	1
330104	1	63	12	0	1	0	1	0
331497	1	49	12	0	0	0	0	0
331532	1	54	12	0	1	1	0	0
331884	2	78	7.53	1	0	0	0	0
332192	2	71	12	0	0	0	0	0
332221	1	58	1.83	1	0	1	0	0
332624	1	88	2.43	1	0	1	0	0
332753	1	75	12	0	1	0	0	0
333813	2	56	12	0	0	0	1	1
333644	1	84	12	0	0	0	0	0
333070	1	60	1.27	1	0	0	0	1
333417	1	51	12	0	1	1	0	1
333481	1	54	12	0	1	0	0	0
333998	1	66	1.33	1	1	1	0	0
334122	1	70	12	0	1	1	0	1
334151	1	60	2	1	1	1	0	0
334240	1	48	2.27	1	0	0	0	1
334485	2	52	12	0	0	0	0	0
334671	1	53	7.1	1	0	1	0	0

335508	1	75	12	0	0	0	0	1
335503	1	72	9.43	1	1	1	0	1
335978	1	46	12	0	1	1	0	1
336406	1	53	5.5	1	1	1	1	0
336433	2	90	1.43	1	0	0	0	1
336908	2	77	12	0	0	0	0	0
337177	1	61	12	0	1	1	0	0
337310	2	67	0.83	1	0	0	1	1
337636	1	58	4.7	1	1	1	0	1
337903	1	68	12	0	1	0	0	0
339396	1	64	0.77	1	1	0	0	1
339524	1	39	4.43	1	0	0	0	0
339556	2	60	0.23	1	0	0	1	0
340427	1	66	0.83	1	0	0	0	0
340440	1	49	3.73	1	1	1	0	0
340584	2	29	12	0	0	0	0	0
341292	1	58	12	0	0	0	1	1
341918	1	54	0.23	1	1	1	0	0
345351	1	51	12	0	0	0	0	0
345424	2	68	12	0	0	1	0	0
344526	1	75	12	0	1	1	0	1
345920	2	46	0.47	1	0	0	0	0
345717	1	48	12	0	1	1	0	0
346751	1	49	0.67	1	0	0	1	0
347434	1	37	0.97	1	1	1	0	0
347072	1	60	12	0	1	1	0	1
348056	1	65	12	0	1	1	0	0
350631	1	43	12	0	1	1	0	0
349450	1	66	0.47	1	1	1	0	1
349577	1	51	2	1	1	1	0	0
348741	1	73	4.3	1	0	0	0	0
349916	1	46	0.63	1	1	1	0	0
350047	1	60	12	0	0	0	0	0
350281	1	58	12	0	0	0	0	1
350279	1	59	12	0	0	1	0	1
350288	1	41	12	0	1	0	0	0
350464	2	56	12	0	0	0	0	0
350883	1	61	1.77	1	0	0	0	0
354449	1	49	12	0	1	1	0	0
353536	1	63	0.43	1	0	0	0	0
353568	1	32	10.33	1	0	0	0	0
353776	1	57	12	0	1	1	1	1
353895	2	35	12	0	0	0	0	0
354292	1	57	0.57	1	0	0	0	0

359750	1	50	0.27	1	1	0	0	0
359451	1	72	12	0	1	1	0	0
360657	1	57	0.97	1	1	1	0	0
360283	1	68	0.17	1	0	1	0	0
360163	2	85	0.33	1	0	0	0	1
360230	1	71	6.3	1	0	0	0	0
360838	1	83	4.9	1	0	0	0	0
361072	1	51	12	0	0	0	0	1
361168	1	62	12	0	1	1	1	0
361551	1	31	12	0	0	0	0	0
361669	1	49	1.27	1	1	0	0	1
362682	1	65	12	0	0	0	0	0
363114	1	50	0.17	1	0	1	0	0
363739	2	56	0.97	1	0	0	0	0
363705	1	54	0.4	1	0	0	0	0
364103	2	82	0.37	1	0	0	0	0
364830	1	62	2.3	1	0	1	0	0
365081	2	76	0.73	1	0	0	0	0
365684	1	57	7.37	1	1	1	0	0
366988	1	59	0.97	1	1	1	0	0
367057	2	62	12	0	0	0	0	0
367087	1	68	12	0	1	0	0	1
367400	1	56	1.73	1	0	0	0	0
367836	1	65	12	0	0	1	0	0
368252	2	79	12	0	0	0	0	1
369420	1	66	12	0	1	1	0	0
369101	2	63	4.07	1	0	0	0	1
369562	1	63	12	0	1	1	0	0
369927	1	60	12	0	1	0	0	0
370891	1	56	4.2	1	1	0	0	0
371054	2	43	12	0	0	0	0	0
371866	1	75	0.33	1	1	1	0	0
372462	1	55	12	0	0	0	0	1
372716	2	76	12	0	0	0	0	1
372790	1	52	1.13	1	1	1	0	1
373676	1	44	12	0	1	1	0	0
373735	1	42	12	0	0	0	0	0
373714	1	75	1.13	1	0	1	0	0
372932	1	60	12	0	0	0	0	1
372853	1	77	3.2	1	0	1	0	0
374459	1	62	1.47	1	0	1	0	0
375565	1	58	12	0	1	1	0	0
376196	1	48	0.6	1	1	1	0	0
377556	2	52	6.57	1	0	0	0	1

377705	1	55	12	0	0	1	1	1
378015	1	78	1.3	1	1	0	0	0
378907	1	54	12	0	1	1	0	1
379333	2	63	0.47	1	0	0	0	1
379959	1	67	12	0	1	1	0	0
380534	2	57	3.1	1	0	0	0	0
381024	1	45	1.73	1	1	1	1	0
381579	1	41	4.63	1	0	0	0	0
381566	1	58	0.5	1	1	1	1	1
381886	1	59	12	0	1	0	1	0
381983	1	51	0.5	1	1	1	0	0
382130	1	54	2.23	1	1	1	0	0
383640	2	64	0.6	1	0	0	1	0
384695	1	72	12	0	1	1	0	1
385080	1	52	12	0	1	1	0	1
385493	1	51	12	0	1	0	0	1
385496	1	69	12	0	1	1	0	1
385505	1	64	12	0	0	0	0	0
385592	1	53	1.2	1	1	1	0	0
385739	2	66	3.4	1	0	0	0	0
385874	1	71	12	0	0	0	0	0
386127	2	52	0.63	1	0	1	1	0
386128	2	61	0.57	1	1	1	0	0
386215	1	52	1.33	1	0	0	1	0
386571	1	28	0.83	1	1	1	0	0
387174	1	53	12	0	0	1	0	0
387466	1	63	7.97	1	1	1	0	1
387484	1	53	0.5	1	1	1	0	0
388122	1	54	0.3	1	0	0	0	1
387992	1	45	2.13	1	1	0	0	0
387849	1	75	12	0	0	1	0	0
388181	1	64	12	0	1	1	0	0
388230	1	40	12	0	0	0	1	0
388310	1	53	12	0	1	0	1	1
388377	1	60	3.3	1	1	1	0	0
388504	1	42	12	0	1	1	0	1
388658	1	65	2.07	1	1	1	0	1
390025	1	45	2.5	1	1	1	0	1
391237	1	46	0.63	1	0	0	0	0
391746	1	59	12	0	1	1	0	1
395313	1	63	12	0	0	1	0	0
395952	1	58	0.1	1	1	1	0	1
396584	1	58	12	0	0	0	0	1
394916	1	52	2.4	1	1	1	0	0

394656	2	46	10.9	1	0	0	1	1
394653	1	46	12	0	1	1	0	0
397628	2	56	12	0	0	0	0	0
398649	1	49	2.07	1	1	1	1	0
398818	1	48	12	0	1	0	0	1
398822	1	28	12	0	0	0	1	0
399637	1	54	3.27	1	1	1	0	1
399104	1	60	12	0	0	0	0	0
398951	1	55	0.5	1	1	1	0	0
398779	1	68	12	0	0	1	0	0
400309	2	53	12	0	0	0	0	0
400655	1	43	12	0	0	0	0	0
401547	2	77	12	0	1	1	0	1
401461	1	63	0.43	1	1	1	0	0
402325	1	67	0.1	1	0	0	0	0
401964	1	45	12	0	0	0	0	1
402748	1	51	12	0	1	1	0	1
403020	2	53	0.17	1	0	0	0	0
403552	1	47	12	0	1	1	0	0
403278	2	66	2.97	1	0	0	0	0
405190	2	75	12	0	0	0	0	0
405169	1	47	12	0	0	0	0	0
405232	1	71	12	0	1	1	0	1
405711	1	50	12	0	0	0	0	0
406802	2	59	12	0	0	0	0	0
406794	1	51	2.6	1	1	1	0	1
407242	2	56	2.9	1	0	0	0	0
407999	1	39	7.93	1		1	0	0
410019	1	81	1	1	1	1	0	1
410312	1	66	0.37	1	0	0	0	0
410645	1	44	12	0	0	0	0	0
410839	1	58	12	0	1	1	0	1
411458	1	37	0.3	1	0	0	0	0
412602	1	66	0.03	1	0	0	0	1
413080	1	41	3.87	1	0	0	0	1
413823	2	74	12	0	0	0	0	0
413947	1	65	12	0	1	1	0	0
414508	1	71	12	0	1	0	0	0
414724	2	70	12	0	0	0	0	0
414968	1	69	12	0	0	0	1	0
415996	1	38	0.93	1	1	0	0	0
417397	1	53	12	0	1	1	0	1
417943	1	51	12	0	1	0	0	1
418612	1	45	12	0	0	0	0	0

419919	1	57	2	1	1	0	0	0
420064	2	63	12	0	0	0	1	0
420736	2	55	12	0	0	0	1	0
422436	2	61	12	0	0	0	0	1
422603	2	72	12	0	0	0	0	0
422800	1	47	7.57	1	1	1	0	1
423692	1	65	12	0	1	1	0	0
424427	2	58	11.4	1	0	0	0	1
424885	1	52	2.17	1	1	1	0	1
425456	1	51	9	1	1	1	1	0
426493	1	77	12	0	1	1	1	1
427902	1	46	12	0	0	1	0	0
427915	1	53	4.3	1	1	1	0	0
428396	1	63	0.47	1	1	1	0	1
428437	2	58	0.63	1	0	0	0	0
428778	2	61	12	0	0	0	0	1
428779	2	58	12	0	0	0	0	1
428793	1	56	4.63	1	1	1	1	0
429302	1	81	1.87	1	0	0	0	0
430152	1	43	0.47	1	0	1	0	0
430523	1		5.13	1	0	0	0	0
431302	1	62	3.13	1	1	1	0	0
431362	1	45	4.6	1	0	0	0	0
431455	1	48	1.93	1	1	1	0	0
432712	1	60	12	0	1	1	1	1
434153	1	60	3.4	1	1	1	1	0
434737	1		3.37	1	1	1	0	1
435492	2	85	6.5	1	0	0	0	0
435757	1	74	12	0	1	0	0	0
435955	1	61	1.8	1	1	1	0	0
436450	2	70	0.47	1	1	1	0	0
437772	1	55	2.8	1	1	1	1	1
439311	1	70	12	0	1	1	0	0
440169	1	45	1.9	1	0	1	0	1
440341	2	77	12	0	0	0	0	0
441613	1	57	12	0	1	1	0	1
442269	1	51	1.53	1	1	1	0	0
442618	1	54	12	0	1	0	0	0
443400	1	52	7.33	1	1	1	0	1
443836	1	58	12	0	1	1	0	1
444479	1	38	12	0	1	1	0	1
445061	1	72	12	0	1	0	0	0
445950	1	62	0.97	1	0	1	0	0
446594	1	71	11.37	1	0	0	0	0

446965	2	64	4.77	1	0	0	0	1
448552	1	66	2.07	1	1	0	0	0
449481	1	84	7.83	1	1	0	0	1
449764	1	39	12	0	1	1	0	0
449878	1	43	6.23	1	1	1	0	0
452184	2	68	11.6	1	0	0	0	1
452274	1	61	12	0	0	1	0	0
453484	1	52	3.5	1	1	1	0	0
454166	1	53	0.43	1	0	0	1	0
454867	1	75	4.17	1	0	0	0	1
454844	1	56	12	0	1	1	0	0
455118	2	60	11.7	1	0	0	0	1
455411	1	49	12	0	0	1	0	0
455666	1	59	12	0	0	1	0	1
456711	1	51	12	0	1	1	1	1
456888	1	66	1.6	1	1	1	0	0
457384	1	57	3.7	1	0	1	0	0
458041	1	90	12	0	0	0	0	1
459095	1	64	10.23	1	1	1	1	0
459194	1		12	0	1	1	0	1
459919	1	62	12	0	0	1	0	0
461569	1	35	0.6	1	1	0	0	0
468343	1	54	12	0	1	1	0	0
478351	1	63	3.3	1	0	0	0	1
479797	1	46	12	0	0	1	0	0
480148	1	63	2.17	1	0	1	0	0
480712	2	66	0.83	1	0	0	1	1
483456	1	60	0.3	1	1	1	0	1
484487	2	60	2.87	1	0	0	0	0
485288	2	53	5.6	1	0	0	0	1
486657	1	47	0.37	1	0	0	0	0
524449	1	57	0.83	1	1	1	0	0
524151	2	83	12	0	0	0	0	0
517547	1	63	0.47	1	1	1	1	0
517905	1	52	6.97	1	1	1	0	0
515597	1	61	0.93	1	1	1	0	0
516185	1	84	0.73	1	0	1	0	0
512008	1	55	6.1	1	1	1	0	0
509840	1	64	6.03	1	1	1	0	1
510698	1	51	2	1	1	1	0	0
529083	1	79	1.7	1	0	0	0	0
539469	1	44	8.3	1	0	0	0	0
539560	1	42	11.07	1	1	1	0	0
538103	1	84	0.8	1	0	0	0	1

539089	1	63	1.77	1	1	1	0	0
544257	1	73	9.3	1	1	1	0	0
544088	2	81	0.3	1	0	0	0	1
549680	1	60	4.2	1	1	1	0	0
558846	1	59	5.27	1	1	1	0	1
563172	1	83	0.93	1	0	0	0	0
563582	1	59	4.27	1	0	1	0	1
564029	1	62	2.63	1	1	1	0	0
589964	2	61	1.1	1	0	0	1	0
590399	1	61	1.2	1	1	1	0	0
594502	1	62	0.47	1	1	1	0	1
594387	1	48	1.37	1	0	1	0	0

hypertension	ascites	encepha	astroi	ALT	AST	TBIL	ALB	GGT	TC
0	0	0	0	46.2	37	10.5	44.4	25.3	2.42
0	1	0	0	13.9	24.3	24.4	48.8	19.5	6.32
0	3	1	1	9.4	38.5	9.8	33.8	79.4	3.23
1	0	0	0	18.5	29.5	7.8	39.5	90	4.8
1	1	0	0	34.8	41.8	28.2	27.9	49.9	3.77
0	0	0	0	47.4	55.4	18.4	35.1	153.1	3.33
0	0	0	0	21.7	24	8.9	43.8	12.2	2.27
1	0	0	0	21.5	26	17.4	42.7	46.6	4
0	0	0	0	46.3	46.3	32.4	32.8	27.9	2.93
0	0	0	0	30.1	31	10.9	41	127.2	2.06
0	0	0	0	41.5	29.6	12.6	48.5	23.8	3.98
0	0	0	0	72.1	39.1	6.8	46.7	131.7	4.07
0	0	0	0	31	34.7	16.2	36.4	33.4	3.53
0	1	0	0	54.7	64.8	21.4	39.3	223.5	3.18
1	0	0	0	23.7	36	30.5	29.3	20.3	4.16
0	0	0	0	23	27.1	20.5	40	24.6	2.16
0	0	0	0	18.1	16.9	8.1	44.3	29.8	4.18
0	1	0	0	40.6	61.9	31.8	30.5	15	3.41
0	1	0	0	22.2	28.5	8.2	44.2	25.1	3.74
0	1	0	0	36	36	16.5	45.4	97	3.67
1	2	0	0	52.8	70.7	75.1	23.7	137.1	2.99
0	1	0	0	22.1	25.4	14.5	43.4	20.5	4.33
0	1	0	0	45.9	99.6	41.1	36.3	565.2	4.04
0	0	0	0	29.8	40.7	31	32.6	44.7	4.54
0	1	0	0	33.3	33.4	27.1	38.7	51.6	2.63
0	0	0	0	21.6	27.8	10.2	46	126.4	5.33
1	1	0	0	416.4	204.1	400.2	39.4	72.8	2.19
0	0	0	1	56	83.5	32	34.8	160.5	4.6
0	1	0	0	374.8	220.8	31.1	37.8	54.5	2.82
1	0	0	0	23.3	25.4	14.2	39.2	26	3.61
0	0	0	0	61.4	52.9	13.7	37.1	39.9	3.49
0	0	0	0	47.7	36.5	32.8	35.7	69.4	3.25
0	0	0	0	20.7	36	6.8	38.1	46.3	2.72
0	0	0	0	19.3	27.7	10.6	42.7	66.9	2.12
0	2	0	0	35.2	23.3	22.4	31.3	97	3.74
1	0	0	0	55.6	42.2	13.8	38.7	49.1	4.22
0	0	0	0	49.4	34.8	11.1	52.3	84.4	6.92
0	0	0	0	15	16.6	9.4	39.2	11.7	3.53
0	0	0	0	19	26.8	15.8	30.1	10.9	2.98
0	0	0	0	149.9	84.9	16.6	37.2	56.5	4.34
0	1	0	0	59.8	37.2	18.3	44.5	95.2	4.38
0	0	0	0	79.9	49.7	9.7	38.7	21.8	3.46
0	1	0	0	18.3	16.6	11.6	46.6	26.5	4.68

0	0	0	0	19.4	17.5	7.5	41.8	61.7	4.1
1	1	0	0	18.1	34.2	19.5	27.4	36.8	2.9
0	0	0	0	55.9	88.1	27.9	33.7	69.7	4.78
1	0	0	0	61.9	37.7	35.2	41.9	394.7	5.22
0	1	0	0	88.9	99.4	35.6	43.9	73.2	7.23
0	0	0	0	15.4	20.7	30.2	37.1	20.1	3.37
0	1	0	0	38.2	35.6	17	38.9	46.9	3.07
0	0	0	0	19.6	33.1	17.5	37.3	38.4	2.35
0	0	0	0	269	132.7	16.7	44.6	121.8	3.15
0	0	0	0	37.5	33.6	8.7	41.7	23.2	3.52
1	0	0	1	26.1	43.3	19.9	35.6	82.1	2.58
1	0	0	0	32.6	23.3	9.4	42.3	56.3	4.1
0	1	0	0	44.9	32.7	19.3	45.6	251.9	3.32
0	0	0	0	27.2	20.5	8.2	47.3	25.9	3.79
0	1	0	0	35.9	49.7	14.3	29.2	87.5	2.79
0	0	0	0	19	23.9	7.2	40.6	37	3.14
0	0	0	0	20.6	30.3	22	44.6	34	3.69
0	0	0	0	37	36	11.5	46.5	30.6	4.54
0	1	0	0	42.7	41.5	23.3	37.1	159.4	2.79
0	0	0	0	19.4	19	14.5	40.5	29.1	4.01
0	0	0	0	36.2	57.4	14.1	25.3	22.1	3.52
0	0	0	0	29.7	30.1	8.4	44.7	29.5	3.22
0	0	0	0	20.7	29.7	11.6	41.5	35.5	4.92
1	0	0	0	23.6	25.2	31.8	33.3	27.6	2.86
0	0	0	0	31	27.9	4.6	42.5	45.1	5.66
0	0	0	0	306.7	193	26.1	39	125.7	3.84
1	0	0	0	76.9	71.6	13.2	43	128.2	4.12
0	0	0	0	31.6	61.5	39	21.7	43	2.88
1	0	0	0	20	21.7	16.9	40.1	24.4	3.25
1	0	0	0	14.8	18.1	13.6	42.1	27.3	3.36
0	1	2	0	32.8	39.8	133.9	24.7	55.5	2.19
0	0	0	0	12.8	18.5	36.1	40.6	15.7	2.89
0	1	0	0	21.7	30.5	21.4	32.3	32	4.06
0	1	0	0	39.2	52.9	21	32.8	73.9	3.68
1	0	0	0	17.9	32.9	21.6	34.4	26.6	3.35
0	1	1	0	31.1	46.8	47.3	35.3	42.7	5.45
0	0	0	0	32.4	28.1	14.6	41.9	61.5	4.41
1	1	0	0	21.4	34.1	27.7	36.1	26.1	3.2
1	0	0	0	41.5	27.7	9.4	40.5	51	3.14
0	1	0	0	40.9	41	22.1	38.8	100.4	4.1
0	1	0	0	14	31.4	11.6	30.1	38	2.95
0	1	0	1	40.9	31.8	25.8	30.1	30.9	2.65
0	0	0	0	25.6	27.6	8.7	49.1	30.5	3.19
0	1	0	0	504.2	383.9	18.9	34.3	147	1.89

1	1	0	0	42.1	118.1	61.5	20.8	88.1	2.91
0	0	0	0	47.3	21.9	12.9	43.3	32.2	2.47
0	0	0	0	32.3	49.6	20.2	33.5	56.3	4.08
1	0	0	0	14.2	35.4	15.2	35.8	60.5	4
0	0	0	0	19.1	16	21.9	44.9	23	4.1
0	1	0	0	46.2	43.4	29.4	36.6	15.2	2.93
1	0	0	0	15.4	24.2	11.2	41.9	24.9	4.05
0	0	0	0	27.7	31.5	17.4	43.8	19.2	3.54
1	0	0	0	43.7	58.4	27.2	34.5	33	4.47
0	0	0	0	20	16.9	9.8	38.8	16.1	2.9
1	0	0	0	22.3	18.7	11.6	33.4	91	2.72
0	0	0	0	31.9	25.5	22.5	37	26.6	4.19
0	0	0	0	48.4	67.6	44.5	26	61.1	3.54
1	1	0	0	47.7	94.6	35.9	33.3	180.2	3.86
0	1	0	0	33.7	74.9	19.4	36.5	214.7	4.98
0	0	0	0	24.5	27.1	11	35.9	13.4	4.73
0	0	0	0	22.7	29.6	14.6	46.2	89.4	3.44
1	3	0	0	64.9	70.7	50.4	33.2	83.2	2.24
1	0	0	0	32.3	27.6	20.1	38.9	27.5	3.68
0	0	0	0	55.8	93.4	22.7	32	158.9	4.5
1	0	0	0	66	74.8	21.2	39.9	84	4.25
0	0	0	0	155.3	80.7	25.8	38.6	36.3	2.91
0	0	0	0	33.5	60.8	20.8	47	34.9	3.57
1	1	0	0	64	139.7	39.1	25.6	57.6	4.54
0	0	0	0	111.3	134.3	42.8	39.6	644.4	13.95
0	0	0	0	23.3	22.2	13.6	42.7	19.6	3.16
1	0	0	0	38.4	34	13.6	41.9	18.4	2.95
1	0	0	0	45.9	40.3	27.6	38.1	94.6	5.89
1	0	0	0	50.9	52.5	28.9	42.1	276.8	8.13
0	0	0	0	48.7	52	14.6	39.7	50	4.03
0	0	0	0	14.3	22.7	9.1	48.7	14.4	2.85
0	0	0	0	17.7	23	11.3	41.5	9.2	2.88
1	0	0	0	22.8	34.1	26	45.5	75.4	4.79
0	0	0	0	63.2	94	36.1	36.4	553.3	4.72
0	0	0	0	32.4	25.9	8.3	43.3	159.9	2.81
0	0	0	0	64.1	46.7	28.7	44.6	241	4.48
1	1	0	0	30.1	41.4	16.3	36.7	157	4.07
0	1	0	0	30.5	39.1	11	33	28.9	4.05
0	1	0	0	84.2	85.4	74.3	27.5	160.8	3.71
0	0	0	0	65.6	67.4	42.5	34.1	274.2	4.52
0	0	0	0	16.3	22.4	19.7	40.4	45.1	5.25
1	0	0	0	29.7	30.1	19.8	40.3	46.4	4.57
1	0	0	0	23.5	31.2	16.6	41.3	82.7	3.87
1	0	0	0	30.9	27.1	16	39.8	23.4	3.44

0	0	0	0	13.3	22.8	20.5	40.1	18.8	3.51
0	0	0	0	32.8	35.5	15.8	46.1	25.1	6.27
0	0	0	0	15.5	32.7	8.4	38.3	34.5	4.99
1	1	0	0	32.4	42.1	22.2	30.6	226.9	5.66
1	0	0	0	21.9	26	17.4	46.7	19	3.43
1	1	0	0	28	35.9	31.3	35.7	136.1	5.12
1	0	0	0	103.6	76.6	13.5	38.8	35.1	4.65
1	0	0	0	39	33.6	15.9	37.7	33.4	4.16
0	0	0	0	60.2	46.2	33.8	40	69.9	3.79
0	0	0	0	73.8	48.5	18.7	39.2	52.7	3.52
0	0	0	0	49.7	36.7	23.4	48.3	100.7	5.75
1	0	0	0	24.1	18	17.9	37.9	35	4.19
0	1	1	0	21.7	29.8	23.1	28.4	14.9	2.79
0	0	0	0	54.7	40.2	18	40.1	84.7	3.97
0	1	0	0	14.9	19.5	16.7	41.7	16.7	2.37
0	1	0	0	39.5	44.6	15.6	32.5	87.4	4.1
0	0	0	0	20.4	34	18.1	47.8	17.8	3.18
1	0	0	0	43.9	61.3	44.9	25.8	188.3	10.48
0	1	0	0	86.1	69.4	13.4	38	148.3	3.8
0	0	0	0	308.3	210.7	15.1	33.9	221.5	4.65
0	0	0	0	46.7	38.3	25.7	38.3	24.3	3.08
0	0	0	0	21.6	24.9	18.7	38.2	28.9	4.12
0	0	0	0	33.7	38.9	7.4	36.5	54.3	3.61
0	0	0	0	21.2	36.3	33.5	26.3	14.8	3.21
1	0	0	0	21.1	25.7	16.2	41.7	14.9	4.28
0	1	0	0	40.1	71.8	49.9	22.3	42.6	2.76
0	0	0	0	36.4	26	22.9	40.9	17.5	3.64
1	0	0	0	142	165.7	32.1	32	189.7	3.09
1	0	0	0	55.7	58.1	12.5	35	72.9	3.97
0	0	0	0	143.2	83.9	18.7	47.3	212.7	4.99
0	0	0	0	24.4	29.2	15.4	34.7	50.6	4.44
0	1	0	0	44.2	80.7	96.9	23.6	44	3.27
0	0	0	0	38.2	50.7	8.4	40.2	45.1	4.03
1	0	0	0	10.7	17.9	14.3	40.5	17.7	3.05
1	0	0	0	224.4	145.5	29.6	40.3	136.4	4.31
0	0	0	0	40.7	26.8	22.9	40.2	26.8	4.65
0	0	0	0	23.7	31	30.2	40.1	22.9	3.68
0	0	0	0	53.7	40.9	12.5	37.8	51.1	3.3
0	0	0	0	141.5	57.1	15.1	37.5	67.1	3.41
0	0	0	0	31.5	23.1	24.4	43	27.2	3.54
1	0	0	0	93.1	55.8	15.1	43.4	126.4	4.24
1	0	0	0	36	33.6	13.1	41	116.5	4.27
0	0	0	0	29.8	40.3	27.8	46.4	83.6	3.55
0	1	0	0	23.1	29.4	18.1	42.3	66.2	2.93

0	1	0	0	45.3	72.9	27.7	29.4	64.5	3.49
1	0	0	0	14.4	20.4	16.2	40.9	13	3.46
0	0	0	0	76.2	37.5	10.8	37.1	63.8	4.66
0	0	0	0	14.9	24.4	30	32.5	13.8	2.82
0	1	0	0	47.5	56.3	34.4	29.6	284	3.74
1	0	0	0	45.6	33.1	20.2	42	45.7	2.81
0	1	0	0	133.8	69.9	11.2	35.4	199.9	4.67
0	0	0	0	35.2	35.5	8.1	43.6	82.9	4.07
1	2	0	0	76.6	21.4	36.7	26.5	22	2.75
0	0	0	0	35.8	34.8	32.7	50.5	69.5	3.65
0	0	0	0	17.5	15.2	9	33.2	41.9	3.58
0	1	0	0	16.9	29.9	6	30.4	58.3	3.62
0	0	0	0	11.4	18.1	11	36.1	27.8	4.12
0	0	0	0	47.6	28.9	7.7	45.5	34.9	3.85
0	0	0	0	179.3	76.8	13.7	34.2	170.3	4.83
0	2	0	0	16.6	34.4	66.2	21	25.1	1.64
0	1	0	0	55.4	59.1	25.4	38.2	321.9	4.14
0	0	0	0	17.3	20.7	23.7	30.4	11.9	2.5
1	0	0	0	97.9	70.4	18.3	40.7	61.6	4.96
0	0	0	0	34.9	30.8	10.4	28.9	58.6	1.86
1	0	0	0	25.5	31	30.8	44.8	22.9	2.96
0	0	0	0	15.4	48.3	24.3	37.8	193.8	9.1
0	0	0	0	38.2	26.9	5.2	44.9	25.5	4.78
0	1	0	0	24.2	30.9	21.5	37.3	83.5	3.69
0	0	0	0	32.9	25.5	11.6	37.3	42.5	3.77
0	0	0	0	16	18.1	16.7	38.9	102.1	3.43
0	0	0	0	51.9	37.4	21	43.8	19.2	4.12
0	0	0	0	23.3	24.6	30.3	37.3	36	3.67
0	1	0	0	17.5	23.8	13.5	37.2	14.1	2.98
0	0	0	0	23.2	41.2	22	35.2	102.7	3.29
1	0	0	0	56.2	33.7	19.9	45.6	128.4	5.02
0	0	0	0	40	32.8	12.6	37.9	48	3.84
0	0	0	0	19	23.1	14.8	43.2	33.1	4.53
0	0	0	0	24.1	27.5	9.3	37.4	114.6	4.65
1	2	0	1	32.9	53.2	18.6	30.7	245.8	3.47
0	1	0	0	20.9	28.1	19.3	45.7	58.3	3.81
0	0	0	0	21.3	34.4	19.9	29.2	10.4	3.97
0	1	0	0	23.1	84.7	64.2	22.2	66.5	1.83
0	0	0	0	43.2	24.1	9.9	44.7	38.1	3.9
0	0	0	0	36.6	32.4	11.3	36.2	44	3.54
0	1	0	0	35.3	37.4	20.7	37.8	102.5	6.82
0	0	0	0	43.4	97	40	34.2	127.9	1.97
0	0	0	0	19.4	25.9	8.9	38.2	18.9	2.34
0	0	0	0	39.2	17.6	8.8	39.9	63.3	5.02

0	0	0	0	16.2	31.1	10.5	40.1	155.9	3.9
1	1	0	0	36.7	42.7	23.5	38.7	216.6	5.43
0	1	0	0	34.9	48	32.3	33.2	69.4	3.73
1	0	0	0	15.2	24.3	7.8	39.4	24.8	4.59
1	0	0	0	22	24.3	18.2	45.5	27	4.67
0	1	0	0	21.1	20.4	22.6	37.7	22.6	2.56
0	1	0	0	22.2	46.8	36.6	29	246.1	5.95
0	1	0	0	17.9	18.9	17.6	31	19.8	4.2
0	0	0	0	20.1	32.6	15.9	35.2	37.2	4.14
0	0	0	0	23.9	12.9	10.4	41.7	29.9	3.53
1	0	0	0	60.8	101.7	27.5	28	84.5	5.36
0	0	0	0	26.8	41.5	32.9	33.1	22.9	4.44
0	0	0	0	24.7	28.5	15.2	37.6	38.6	3.26
0	0	0	0	27.6	23.5	11.7	37.4	49.4	4.69
0	0	0	0	149.5	119.9	10.8	36.6	108.6	2.47
0	3	0	0	45.6	61	27.4	30.6	21.9	1.84
1	0	0	0	232.8	90.5	20.8	37.1	69.2	3.47
0	0	0	0	44.3	53.4	36.3	30.9	60	4.2
0	0	0	0	18.5	22.2	7.9	37.7	13.6	3.44
0	0	0	0	19.8	41.9	20.7	33.4	244	4.96
1	0	0	0	862.5	357.7	19.9	35.9	223.7	4.53
0	0	0	0	23.9	32.3	35.3	33.7	50.8	3.64
0	0	0	0	15.7	20.8	9.9	41.4	24.5	3.37
0	0	0	0	12.3	23.8	29.2	41.4	45.2	2.9
0	0	0	0	27.1	49.6	15.7	33.8	302.5	3.91
0	0	0	0	41.3	26.5	30	42.2	132.5	3.34
0	0	0	0	46	25.2	14.6	41.3	67.7	4.46
1	2	0	0	15.2	41.2	26.1	28.5	294	4.28
0	0	0	0	9.9	18.7	19.4	34.7	16.7	4.1
0	0	0	0	14	23.4	13.8	42.9	15.4	2.86
1	0	0	0	14.2	20.1	7.8	41.6	24.6	4.41
0	0	0	0	8.9	18.9	15.2	41.7	42.7	3.43
0	0	0	0	13.1	22.1	5.2	35	12.3	2.21
0	0	0	0	25	21.8	12.1	39.1	35.9	4.38
0	0	0	0	27.9	54.5	57.5	21.9	21.8	2.93
0	0	0	0	16.6	23.4	22.4	46.7	17.8	4.75
1	1	0	0	18.4	31.2	9.3	35.5	33.4	5.45
1	0	0	0	50.7	63.7	53.2	35.6	70.6	3.75
0	0	0	0	21.9	28.3	16.1	38.1	26.8	2.71
1	0	0	0	13.2	22.1	11.5	41.3	11.1	3.62
1	0	0	0	29	27.8	23.7	41.6	21.7	3.38
0	0	0	0	43.3	40.7	16.2	41.1	23.9	3.9
0	1	0	0	61.5	46.9	17	36.9	200.5	6.02
0	1	0	0	28.4	73	37.5	26.5	85.3	2.48

1	0	0	0	126.8	92.9	16.8	41.6	89.5	4.23
0	0	0	0	20.9	27.2	8.5	40.5	17.9	3.94
0	0	0	0	13.9	59.3	15.5	42.4	54.5	2.99
0	0	0	0	55.3	40.9	16.1	41.5	112.6	3.56
1	0	0	0	21.9	21.6	8.6	40.9	20.5	3.55
0	0	0	0	69.1	35.8	12.2	39.7	47.3	3.63
0	3	0	0	17	27.2	32.8	27.9	43.4	1.92
0	0	0	0	48.7	33.7	8.5	42.8	121.2	5.55
0	0	0	0	41.1	45.7	20.8	39.1	173.6	3.11
1	0	0	0	11.9	15.3	8.4	39.7	24.9	4.72
0	0	0	0	49.5	28.2	15.5	41.6	41	4.18
1	0	0	0	23.9	14.9	11	36.5	28.5	4.18
0	0	0	0	63.7	27.1	12.6	36.4	68.3	3.55
0	1	0	0	29.2	46.3	15.8	28.5	25.5	3.09
1	0	1	0	41.7	79	65.2	34.3	2192	5.14
0	0	0	0	30.7	20.8	12	39.4	42	3.11
0	1	0	1	54.4	62.1	56.6	37.7	61.5	3.53
0	0	0	0	25.2	20.6	10.4	44.3	40.5	3.79
1	0	0	0	703.5	855.9	91.6	33.7	83.2	3.09
1	0	0	0	18.2	40.4	29.5	30.4	67.7	4.63
0	0	0	0	31.2	34	18.4	30.3	14.1	3
1	0	0	0	28.1	26.9	7.5	30.8	20	3.84
0	0	0	0	31.6	27.3	10	40.5	30.4	4.2
0	3	0	0	156	350.5	163.1	29.4	1049.8	5.7
1	0	0	0	506.5	310.4	24.4	32.4	87	3.06
0	0	0	0	31	33.1	30.3	46.3	66.9	4.36
0	1	0	0	19.5	26.2	19.4	32.5	16.4	3.67
0	0	0	0	38	74.6	22.5	43.8	186.4	3.79
0	2	0	0	31.4	62.7	19.5	23.1	35.6	3.37
0	0	0	0	38	50.5	10.9	36.5	144.6	3.68
0	1	0	1	47.5	69.2	36.4	30.3	67.2	4.83
0	0	0	0	14.1	30.7	25.9	31.7	14.9	3.25
0	0	0	0	59.4	60.1	14.9	39.6	17.7	4.57
0	1	0	1	46.1	35	36.7	34.7	43.8	2.36
0	0	0	0	24.7	32.6	23	30.9	23	3.05
1	0	0	0	12.6	36.6	40	31.9	15.4	3.41
0	0	0	0	25.2	45.6	18.8	32.6	23.2	3.6
0	0	0	0	26.2	32.5	26.1	33.6	64.3	4.63
1	1	0	0	38.8	51.9	14	31.1	62.4	4.45
0	0	0	0	24.7	27.7	18.1	45.4	88.7	5.2
0	0	0	0	71.3	58.4	19.6	31.9	70.1	3.62
1	2	0	0	81.7	99.3	35.5	31.5	488.3	5.6
1	0	0	0	40.6	36.8	9.4	43.9	49.3	4.24
0	0	0	0	46.1	65.8	14.7	38.6	201.3	4.37

1	0	0	0	21.9	29.5	14	42.4	44.9	5.18
0	0	0	0	19.1	23.2	18.5	32.5	23.7	3.38
0	1	0	1	23.3	24	24	27.4	26.9	2.49
0	0	0	0	76.6	84.6	47.8	25.8	69.7	3.58
0	0	0	0	36.1	56.7	25.8	31.6	25.9	3.16
0	0	0	0	42.1	35.1	15.3	34.8	36.6	3.1
0	0	0	0	60.1	44.3	31.7	43.8	177.5	4.51
1	1	0	0	20.5	26.6	12.8	35.4	20.9	4.42
0	1	0	0	48.9	72	46.6	36.5	33.4	2.89
1	0	0	0	17.5	11.5	18.9	38.5	30	4.38
1	1	0	0	68.3	79.2	27.5	24.9	135.3	5.27
1	0	0	0	20.9	22.9	6.9	41.6	36.1	6.24
0	0	0	0	23.4	28.2	25.2	40.9	20.5	3.92
0	0	0	0	22.6	32.2	21.7	38.7	21.9	4.15
1	0	0	0	23.4	16.3	9.4	44.1	27.7	2.99
0	0	0	0	47.5	29.3	15.4	38.4	33.4	2.68
0	0	0	0	19.7	53	26.2	42.8	122.5	3.19
0	0	0	0	28.8	24.3	8.2	39.1	30.1	4.51
0	0	0	0	30.7	37.1	18.2	38.6	125.1	3.46
0	0	0	0	85.2	45.3	12.5	38.6	192	5.73
1	0	0	0	15.7	18.8	20.8	37.9	30.9	3.38
0	0	0	0	24.3	19.8	16.2	46.7	20.5	4.25
1	0	0	0	74.2	45.8	7.4	40.8	89.7	4.15
0	0	0	0	32.1	22	12.7	42.7	31.2	3.27
1	0	0	0	14.4	24.6	9.7	36.8	197.7	4.11
0	0	0	0	25.6	26.1	16	38.4	86.1	4.01
0	0	0	0	34.1	31.6	8.3	39	24	3.85
0	0	0	0	39.1	44.2	16.2	41	110.4	3.98
1	0	0	0	56.7	34.8	8.6	40.1	615.5	3.11
1	0	0	0	18.9	20.4	8.2	42.9	17.5	4.62
0	0	0	0	19.7	21.9	25.9	37.6	17.3	2.95
0	0	0	0	27.1	30	27.1	31.8	63	2.64
0	0	0	0	45.7	28.1	9.4	38.8	39.3	4.38
1	0	0	0	18.5	20.4	8.9	43	17.5	3.6
0	0	0	0	721.5	364.9	22	33.2	188.7	4.33
0	1	0	0	28.9	36.4	8.9	37	196.5	2.92
0	0	0	0	10	23.6	7.6	40.4	34.4	4.44
0	0	0	0	55.9	43.7	28.2	46.5	59.5	3.79
0	0	0	0	35.4	35.4	17.8	35.8	153.7	4.49
0	1	0	0	36.4	70.6	34.1	22.6	152.2	2.85
0	0	0	0	8.9	16.6	10.3	41.6	9.8	4.85
1	0	0	0	29.7	35.5	13.3	30.6	47.1	2.82
1	0	0	0	45.4	83	60.9	33.9	228.6	3.01
1	1	0	1	46	104.4	10.8	35.5	85.8	2.89

0	0	0	0	138.4	102.7	8.1	34.3	34.5	3.15
0	0	0	0	27.1	34.1	41.3	25.2	63.4	2.73
0	0	0	0	17.6	19.9	8.9	47.7	24.8	4.32
0	0	0	0	24.6	29.7	9.5	36.4	18	3.65
0	1	0	0	44.9	65.5	41.8	34.8	310	5.88
0	0	0	0	21	24.9	10.2	36.1	30.5	5.19
1	0	0	0	47.1	54.7	19.7	30.9	121.2	3.71
0	1	0	0	22	52	31.5	32.7	31.5	2.57
0	1	0	0	142.7	49	22.6	34.4	218.7	4.2
1	1	0	0	33.2	54.5	11.9	33.1	125.4	3.44
0	0	0	0	95	35.1	22	33.1	94.8	3.61
1	0	0	0	28.1	27.5	9.8	37.1	19.2	3.87
0	0	0	0	27.6	28.1	29.9	29.3	99.2	3.38
1	0	0	0	223.9	122	19.2	40	115.5	3.33
0	0	0	0	22.8	33.6	18.7	34.8	21.3	4.54
0	0	0	0	41	38.1	16.4	40	152.1	3.64
1	0	0	0	32.7	56.2	31.1	40.6	78.5	3.17
0	0	0	0	32.4	30.8	22.7	43.6	33.8	4.91
0	0	0	0	43.6	37.2	9.6	34.6	195.5	4.91
0	0	0	0	40.9	41.3	12.7	34.5	46.9	3.21
0	1	0	0	65.3	67.7	18.9	34.6	100.6	4.03
1	0	0	0	19.3	43	16.7	41.6	44.1	4.89
0	0	0	0	31.5	30.4	13	39.8	38.1	4.12
0	0	0	0	39	41.3	10.5	36.1	30.8	3.72
0	0	0	0	42.8	55.9	11.2	33.6	48.9	2.64
1	2	0	0	47	129.1	10.8	32.7	245.9	8.23
0	1	0	0	24.6	26.5	14.9	42.7	25.2	3.71
0	0	0	0	23.7	22.5	12.6	45.2	18.2	2.88
0	1	0	0	18	20.6	10.2	36.9	68.9	6.52
1	0	0	0	34.1	47.8	16.7	48	53.2	5.16
0	0	0	0	26.1	21.9	8.4	44	36.9	4.6
0	0	2	0	34.5	52.2	30.7	34.8	57.5	4.22
1	0	0	0	31.1	28.1	12	48.5	73.1	4.01
0	0	0	0	17.5	39.1	11.7	34.2	49	3.82
0	0	0	0	121.1	134.8	23.8	33.6	58.4	6.84
0	0	0	0	47.9	50.8	13.8	39.5	139.5	3.16
1	3	0	0	88.2	198.1	23.6	28.7	480.6	3.61
1	0	0	0	35.1	48.7	36.2	31.4	54.5	4.97
0	0	0	0	164.6	129.9	11	30.4	178.4	4.56
0	1	0	0	25.2	36.9	33.4	35.2	85.3	4.05
0	0	0	0	18.8	26.5	16.6	30.6	105.4	3.48
1	0	0	0	18.9	35.8	12.7	40.2	29.6	6.17
0	0	0	0	98.9	52.1	24.8	36.9	63.8	4.79
0	0	0	0	35.2	45.6	24.1	30.3	31.1	3.04

1	0	0	0	36.5	47.2	9.9	37	128.6	5.31
0	0	0	0	59.9	57.4	12.2	43.9	181.6	7.74
0	0	0	0	34.5	22.5	12	34.4	33.8	3.63
0	0	0	0	46.3	46.5	16.7	35.2	71.5	4.02
0	0	0	0	20.2	22.7	15	43.5	16.6	3.16
0	0	0	0	16.3	21.1	13.7	42.9	18.6	4.58
0	0	0	0	28	51.6	35.2	37.6	18.4	3.56
0	3	0	1	34.1	76.2	149.8	24.8	27.6	0.9
1	0	0	0	48.6	73.3	18.2	31.6	47.5	3.68
1	2	0	0	35.1	39.2	16.6	33.4	197.1	3.36
0	0	0	0	15.1	34.5	19.7	26.2	40.8	3.85
1	0	0	0	16.9	16	5.2	39.7	33.6	3.21
1	1	0	0	18.1	25.3	7.7	45.1	22.7	2.77
1	0	0	0	26.5	22.4	20.5	47.4	25.3	2.63
1	0	0	0	18	28.9	10.3	47.6	34.3	5.04
0	0	0	0	57.4	40.6	9.7	34.7	55.1	4.23
0	0	0	0	22.7	16.3	6.9	39	16.8	4.39
0	0	0	0	42.2	48.2	24.5	38.1	29	4
0	0	0	0	21.8	27.3	24.5	30.7	17.2	4.14
1	0	0	0	9.8	25.7	13.1	40.3	107.3	4.37
0	1	0	0	22.8	27.1	21.5	28.9	27	3.21
0	0	0	0	30.3	30.4	12.1	38.3	53.1	4.77
1	1	0	0	24	33.2	11.6	37.7	48.1	3.74
0	1	0	0	21.6	22.9	14.1	33.3	77.8	2.56
0	0	0	0	25.4	18.1	17.8	41.7	43.1	4.15
0	0	0	0	30.2	52.2	11.5	42.8	20.6	4.54
0	0	0	0	20.7	28.8	9.3	38.5	25.5	4.79
1	0	0	0	39.2	33.7	11.1	34.2	172	4.87
0	0	0	0	30.7	37.7	20.2	41.7	44.4	4.19
0	0	0	0	33.2	36.6	8.7	30.4	43	3.24
0	0	0	0	13.4	22.6	9.7	34.8	50.6	4.71
0	0	0	0	29	27.5	14.4	47	31.6	6.24
1	0	0	0	29.6	27.8	18.7	48.8	64.9	4.26
0	0	0	0	49.4	90.9	29.4	36.4	133.5	5.62
0	0	0	0	14.4	19.3	10.5	39.6	16.1	3.98
0	0	0	0	28.3	37.2	13.2	33.3	34	3.64
0	0	0	0	68.9	57.6	12.3	36.9	93.6	3.36
0	0	0	0	75.9	44.1	23.5	42.7	40.6	3.03
0	0	0	0	22.4	28.1	27.7	38.2	11.9	2.24
0	0	0	0	8.9	14.3	11.3	32.6	24.6	2.36
1	0	0	0	27.1	37.7	15	42.2	89.4	5.77
0	0	0	0	23.7	29.3	19.4	39.6	49.9	3.93
1	0	0	0	35.6	48.5	15.2	44.1	74.2	4.35
0	0	0	0	25.6	26.2	38.9	36.6	31.4	2.6

0	1	0	0	23.3	37.6	14.3	41.1	143.1	4
0	0	0	0	47.2	36.2	18.8	39.3	260.3	3.45
0	1	0	0	31.1	39.6	18.4	37.6	38.3	3.02
1	0	0	0	401.5	231.8	43.7	39.7	421.2	3.76
1	0	0	0	21.1	17.9	9.3	47.6	19.6	4.6
0	0	0	0	22.8	24.8	57.8	27.6	16.7	3.6
0	0	0	0	52.4	30.1	14.4	37.8	34.2	3.88
0	0	0	0	47.2	37.7	8.2	36.5	21.4	3.59
0	0	0	0	23	35.1	32.2	33.3	23.6	3.9
0	0	0	0	50.6	21.8	8.2	42.5	18.4	3.77
0	0	0	0	27.6	17.8	14.4	45.4	24.8	3.68
0	0	0	0	35.7	45.3	20.2	36.5	120.7	4.28
1	0	0	0	63.1	39.5	14.1	40.8	58.9	4.43
1	0	0	0	21.7	14.1	12.3	41.9	37.1	3.99
1	2	0	0	24	29.1	19.4	35.9	135.6	5.19
0	0	0	0	13.8	18.6	8.1	42.2	11.2	3.07
1	0	0	0	46.2	45.6	10.2	41.1	109.7	5.05
0	1	0	1	19.9	69.1	25.5	26.8	48	2.31
1	0	0	0	75.2	130.7	44.6	31.8	459.3	4.85
0	2	0	0	17.3	25.5	17.7	34	39.4	3.89
0	0	0	0	22.8	19.8	12.8	43.9	14.5	5.03
0	0	0	0	13.5	21.2	19.1	38.2	19.3	2.47
1	0	0	0	37.4	17.9	10.1	45.8	42.1	6.22
0	0	0	0	40.4	44.8	17.3	31.8	226	4.3
0	0	0	0	33.9	37.1	15.7	45.5	62.7	3.65
1	0	0	0	34.1	27.5	10.1	37.4	30.8	3.69
0	0	0	0	23	30.6	25.8	34.8	29.7	2.86
0	0	0	0	15.7	18.7	9.3	41.6	86.3	4.77
0	0	0	0	22.4	28	18.2	40.4	18.8	1.9
0	0	0	0	58.6	55.7	22.8	44.3	131.6	3.4
0	0	0	0	13.8	16.1	11.4	43	30.4	4.18
0	1	0	0	16.8	30	13.3	41.8	163.1	4.14
0	1	0	1	46.7	94.3	19.9	33.3	237.1	3.23
1	0	0	0	21	32.5	10.6	43.7	106	4.3
0	0	0	0	7.6	15.5	12.1	40.4	13.3	4.11
0	0	0	0	14	18.8	8.5	44.4	16.3	4.14
0	0	0	0	36.6	35.8	14.8	42.2	138.1	4.17
0	3	0	0	28.3	44	9.7	33.6	21.1	3.83
0	0	0	0	21.4	21.1	9.3	46.6	18.7	4.01
0	0	0	0	37.4	21.1	6.1	41	29.4	3.84
0	2	0	0	19.9	28	12.8	33.7	23.1	4.23
0	1	0	0	25.1	29.7	27.5	30.1	33.5	2.72
0	0	0	0	32.2	38.7	37.8	40.8	32.5	3.21
1	0	0	0	57.5	36.6	9.9	41.6	55.2	3.18

1	1	0	0	35.8	30.9	17.4	38.7	50.7	3.6
0	0	0	0	31.1	26.1	19.4	42.9	18.9	5.55
0	0	0	0	22.9	26.2	20.2	39.7	18.6	4.61
1	2	0	0	78.1	84.5	30.7	33.1	222.5	5.81
0	0	0	0	21.8	56.5	6.1	43.8	73.4	4.48
0	0	0	0	26.9	32.9	21.3	33	31.2	4.13
0	0	0	0	27	25.3	25.4	49.1	25	4.3
0	0	0	0	20.6	38	15	38.6	30.8	5.54
0	0	0	0	55.7	33.3	12.6	42.6	20.6	2.9
0	0	0	0	57	51.6	12	46.4	117.4	6.87
0	0	0	0	39.5	24.9	17.2	40.8	43.1	3.8
0	0	0	0	57.4	64.1	8	40.6	59.2	3.17
0	2	0	0	35	51.7	34	32	82	3.72
0	1	0	0	27.6	27.5	41.7	31.9	24.6	2.98
1	0	0	0	43.2	26.9	10.7	44.5	89.7	4.24
0	0	0	1	21.7	35.6	13.7	36.7	13.8	2.32
0	0	0	0	61	38.4	13.4	36.8	53.3	3.85
1	0	0	0	14.5	26.6	15.2	38.1	163.3	6.77
0	0	0	0	30.2	31.1	15.8	34.8	52.3	3.19
0	0	0	0	35	25.5	9.7	45.2	66.7	4.42
0	0	0	0	31.2	19.5	17	35.5	85.8	4.45
0	0	0	0	18.9	25.7	12.4	51.6	27.6	4.44
1	2	0	0	18.5	30.6	22.5	28.4	20.6	3.34
0	0	0	0	40	46.8	40.1	32.1	22.5	3.98
0	0	0	0	38.7	354.1	26	36.4	451.2	5.94
0	0	0	0	37.5	28.5	7.8	46.3	80.3	5
0	1	0	1	18.2	26.4	17.1	26.3	69.3	2.16
0	0	0	1	26.9	25	13.4	41.1	39.1	3.02
0	1	0	0	38.1	52.4	25.6	29.9	93.5	4.4
1	0	0	0	45.1	39	13.2	33.1	69.4	2.43
0	1	0	0	32.9	30.9	23.4	29.1	109.9	3.8
0	0	0	0	39.3	29.6	10.6	39.3	28.8	4.18
1	1	0	0	19.8	23.8	30.3	35	12.4	3.29
0	0	0	0	20.4	21.5	10.1	43.7	15.4	4.45
0	0	0	0	24.9	37.9	10.8	32.6	101.1	3.05
0	0	0	0	64.8	27.8	13.3	45.9	33.7	6.36
0	0	0	0	289.7	129.4	117.4	33.7	111.5	3.68
0	0	0	0	24.3	14.5	16.7	43.9	22.2	4.45
0	0	0	0	17.9	21.6	10.2	39.7	111.1	4.82
0	0	0	0	25.3	22.4	9.6	41.5	69.2	4.34
0	0	0	0	32.8	109.1	16.2	31.7	66.5	4.85
0	0	0	0	29	26.2	12.5	41.2	31.8	2.87
0	0	0	0	24.3	36.3	19.7	37.6	22.9	2.66
0	0	0	0	27.3	26.4	6.9	42.6	28.9	4.5

0	0	0	0	13.5	14.9	10.1	40.8	29.4	3.35
0	0	0	0	29.9	57	25.1	43.2	37.4	3.75
0	0	0	0	38.3	32.1	13.5	36.9	159.9	4.17
0	1	0	0	47.2	47.4	24.5	37.9	279.2	3.69
0	0	0	0	30.9	23.2	10.6	49	70.2	6.2
0	3	0	0	18.1	28	27.8	35.1	88.7	2.69
0	0	0	0	26	27.8	16.7	42.5	119.8	3.35
0	0	0	0	13.4	23.7	9	37.8	14	4.22
0	0	0	0	85.3	55.5	9.6	43.8	70.4	3.96
0	0	0	0	23.6	21.9	17.1	40.6	24.4	2.88
0	0	0	1	19.7	24.5	12	35.3	50.2	2.43
0	0	0	0	45.5	47.4	9.5	35.8	39.1	4.29
0	0	0	0	38.1	53.5	12.4	42.5	62.1	4.1
1	0	0	0	37.8	29.7	9.7	42.3	31.8	4.45
0	0	0	0	46.2	34.4	7.6	36.8	83.1	4.35
0	0	0	0	12.3	29.2	9.3	43	15.7	4.83
0	0	0	0	25.7	21.6	47	41.5	35.9	3.36
0	0	0	0	45	48.8	6.7	35.8	22.9	3.53
1	0	0	0	27.1	64.5	41.8	25.4	44.4	3.32
0	0	0	0	27.1	62.5	17.4	32.3	25.1	3.43
0	0	0	0	20.5	36.9	7.8	44.5	27.5	3.91
0	0	0	0	79.5	46.1	8.7	42.8	126.3	4.64
1	0	0	0	21.8	21.9	9.5	38.1	48.6	4.03
0	0	0	0	24.2	26.5	16.6	39	10.8	4.82
0	0	0	0	28	24.1	14.1	38.7	20.8	2.85
0	0	0	0	22	21.3	19.5	45.7	18.3	4.83
1	1	0	0	17.4	43	44.5	33	217.5	3.63
1	0	0	0	45.3	48.9	12.5	36.6	51.5	3.91
1	0	0	0	35.8	40.2	12.2	38.2	21.6	3.73
1	1	0	0	88.7	66	22.5	35.4	188.3	3.93
0	0	0	0	86.5	68.4	26.4	32.9	139.6	3.46
0	1	0	0	22.2	21.8	11.1	39.9	30.3	3.15
0	0	0	0	33.2	33.4	17.8	31.3	58.6	3.84
0	0	0	0	57.1	69.5	40.2	29.2	57.7	2.36
1	0	0	0	150.8	88.2	19	44.6	50.9	3.6
0	0	0	0	29.7	32.3	14.4	37.1	23.8	3.69
0	0	0	0	54.4	47.1	8.2	35	58.9	3.18
0	0	0	0	19.2	25.4	22	40.1	22.8	4.59
0	0	0	0	20	14.4	8.5	37.9	45.1	3.14
0	0	0	0	114.5	105	21	33.4	150.3	3.29
0	0	0	0	30.7	25.3	11.8	44.2	50.5	4.39
0	0	0	0	62.7	95.3	6.6	38.8	21.8	3.4
0	0	0	0	51	101.8	16.8	36.6	140.2	5.83
0	0	0	0	86	59	13	43.7	118.9	4.5

1	0	0	0	57.6	47.4	11.5	42.3	77.9	4.24
1	0	0	0	26.6	63.8	16.6	34.9	48.1	4.01
0	1	0	0	40.2	34.8	12.3	37.3	220.2	4.58
1	0	0	0	38.7	56.1	12.7	33.9	113.4	4.09
0	0	0	0	26.6	22.6	8.8	32.9	31.6	2.28
0	0	0	0	24.6	28.6	14.5	47.2	19.8	3.52
0	0	0	0	67.3	37.8	23.3	36.8	316.9	5.13
1	0	0	0	43.9	24.8	16.2	39.3	65.7	4.52
0	0	0	0	46.9	31.1	12.4	43.1	44.4	5.52
0	0	0	0	17.3	19.9	7.1	41.1	20.8	2.98
0	0	0	0	27.9	23.2	13.3	37.4	32.1	2.17
0	0	0	0	57.4	37.2	29.6	40.8	186.2	4.4
0	0	0	0	102.1	80.7	20.8	43.9	81.4	4.78
0	0	0	0	15.4	16.6	12.9	36.8	20.6	3.09
0	3	0	0	61.3	104.4	38.5	31.7	133.9	4.05
0	0	0	0	301.5	112.5	35.2	40.6	133.9	4.03
0	0	0	0	16.4	19.3	14.9	39.6	12.4	4.27
1	0	0	0	941.7	315.3	22.5	44.2	125.3	3.83
0	0	0	0	55	59.7	16.9	34.8	32.4	3.04
1	0	0	0	22.8	20.9	16.8	45.5	48.7	4.26
0	0	0	0	78.8	71.3	31.5	38.4	65.3	4.43
0	1	0	0	47	37.3	37.6	29.7	111.1	4.34
0	0	0	0	19.1	27.6	11.8	38.7	71.5	2.88
0	0	0	0	56.8	33.6	10.1	51.2	39	3.92
0	0	0	0	11.1	16.6	9.2	39.8	16.8	3.75
0	0	0	0	15.3	18.3	10.9	37.9	16.7	3.51
0	0	0	0	33	29.8	9.1	40.3	62.6	5.28
0	0	0	1	72.8	57.9	12.2	35	255.4	2.6
0	0	0	0	29.6	20.9	7.4	46.4	37.8	4.56
0	0	0	0	39.7	55.6	18.5	35.9	75	2.74
0	0	0	0	72.7	64.7	27.7	51.7	257.5	3.72
0	0	0	0	58.9	35.6	13.2	42.3	34.3	3.96
0	0	0	0	99.1	108.2	18.8	37.7	270.9	5.45
0	0	0	0	32.5	29.2	10.4	37.6	47.1	4.04
0	1	0	0	31.3	39.2	37.9	30.4	22.8	3.1
0	1	0	0	99.8	88	22.5	29.6	95.1	2.36
0	0	0	0	35	35.1	21.1	48.8	15.9	2.58
0	1	0	1	139.2	229	10.3	34.6	114	3.44
0	0	0	0	71.8	66.8	16.8	35.6	359.6	4.29
0	0	0	0	49.9	41.9	8.3	43.4	185.9	3.29
0	0	0	0	24.9	21.4	11.8	42.9	38.5	4.27
1	1	0	0	17	47.2	29.7	36.5	75.1	5.01
0	0	0	0	140.7	100.4	27.9	38	135.9	5.47
1	1	0	0	42.5	54.8	20.3	35.3	49.1	3.43

0	0	0	0	17.1	19.5	36.7	31.6	69.4	2.73
0	0	0	0	25.3	29.6	25.8	38.5	176.5	4.58
0	1	0	0	38.1	29.1	11	45.9	31.8	3.8
0	0	0	0	14.9	12.6	11.7	39.8	82.6	4.85
0	0	0	0	30.4	29.1	17.4	39.6	33.3	3.93
0	0	0	0	26.7	33.4	19.4	50	15.6	3.65
0	0	0	0	175.6	67.4	18.6	51.7	237.4	4.43
0	0	0	0	35.5	33.6	11.7	38.8	75.3	4.1
1	1	0	0	80.2	113.2	34.7	33.3	159.4	3.2
0	0	0	0	21.7	22.2	17.3	45	13	3.46
1	0	0	0	18.8	16.8	3.6	42.9	40.7	4.29
0	0	0	0	20.7	30.6	39.8	40.6	15.4	2.85
0	1	0	0	9.5	13	6.8	34	19.9	3.52
1	0	0	0	14.4	17.1	4.2	34	23.9	4.63
0	0	0	0	37.1	23.6	12.5	43	41.5	3.96
1	0	0	0	64.2	42.3	17.1	37.1	46.9	3.92
0	1	0	0	22.1	23.4	14.4	32.2	25.5	4.1
1	0	0	0	23.2	27.2	18.5	46.7	84.7	5.18
0	1	0	0	47.4	46.2	28.3	39	344.4	4.13
0	0	0	0	42.4	40.5	14.2	42.4	44.5	4.46
0	0	0	0	20.2	23.4	12.7	34.9	34.7	4.6
0	0	0	0	35.6	28.9	9.9	47.3	18.5	4.54
1	1	0	0	32.4	36.3	25.5	42.9	234.9	3.69
0	0	0	0	13.4	23.7	9.8	38.9	27.7	4.9
0	0	0	0	40	49	19.1	36.8	150.7	3.17
0	0	0	0	201.9	143.6	56.4	24.8	85.9	2.02
0	0	0	0	14.1	18.7	14.4	34.9	67.5	4.41
0	0	0	0	41.6	39.2	8.4	38.5	116.3	4.1
0	0	0	0	129.2	124.3	17.4	36.7	148.4	3.87
0	0	0	0	25	27.1	10.3	35.5	76.4	4.06
1	0	0	0	26.9	30	11.5	35.7	36.9	2.68
0	0	0	0	52	61.7	40.3	33.3	35.2	2.81
1	1	0	0	27.9	53.2	15.7	28	67.3	3.2
0	0	0	0	23.7	28.9	12.4	39.7	14.7	5.05
0	1	0	0	19.4	49.8	32.4	26.8	17.2	2.54
0	0	0	0	22.4	32.2	18.6	35.4	47.1	4.01
0	0	0	0	44.4	33.1	19.6	41.7	59.1	3.73
0	0	0	0	26.2	18.2	10.6	39.4	20.4	2.77
0	0	0	0	31.4	26.4	15	43.8	22.4	4.25
1	0	0	0	28.5	18.5	19.5	41.7	20.7	3.03
0	0	0	0	33.2	33.3	17	42	281	4.45
0	0	0	0	48.8	27.3	11.4	47	49.5	5.95
0	0	0	0	21.8	28.3	18.3	45.5	15	4.47
0	0	0	0	44.3	28.5	6	42.5	46.9	4

1	0	0	0	95.9	154.2	271	31.4	66.1	3.25
0	0	0	0	128.2	61.8	40.6	36.7	297.2	3.65
0	1	0	0	24.4	29.7	20.5	38.3	22.5	3.15
0	0	0	0	34.5	25.6	13.5	40.5	75.3	5.05
0	1	0	0	54.3	56.7	28.1	30.2	30.6	2.9
1	0	0	0	21.1	23.5	18.2	45.6	18.4	4.31
0	0	0	0	29.1	28.4	17.2	44.5	14.5	4.67
0	0	0	0	52.1	64.7	18.7	39.4	326.8	4.82
0	0	0	0	47.8	67.3	24.7	34.4	71.6	3.4
1	0	0	0	10.9	13.9	18.9	43.4	24	4.14
1	0	0	0	73.2	39	12.1	40.3	326.5	3.65
0	1	0	0	18.9	35	32.6	23.9	30.6	2.64
0	0	0	0	69.9	79.4	16.5	32.6	332.3	6.37
0	1	0	0	24	26.8	18.2	39.4	66.8	3.93
0	1	0	0	29.3	44.8	34.3	25.8	66.4	2.82
0	0	0	0	1192.4	677.8	35.3	42.6	242.2	4.65
0	0	0	0	15.6	18.6	14.4	40	8.8	3.47
0	0	0	0	22.2	23.6	13.1	45.5	20.4	4.52
1	0	0	0	21.2	20.3	12.1	39.1	20.5	3.33
0	0	0	0	82.4	60.1	7	37.1	368.7	4.76
1	0	0	0	13.4	14.7	10.4	39.4	17.7	3.66
0	0	0	0	142.6	68.1	17.5	34	340.8	5.19
1	0	0	0	19.9	18.4	5.3	45.1	486.9	4.58
0	0	0	0	18.3	15.7	13.8	45.4	20.6	4.89
0	0	0	0	24.7	55.3	22.9	31.6	104.7	5.7
0	0	0	0	15.9	16.5	11.4	47.8	15.8	4.06
0	0	0	0	11	28.3	27.2	39.4	38.6	3.15
1	1	0	0	80.2	107	27.3	22.8	61.8	2.41
0	0	0	0	34.7	27.5	8.1	35.4	41.3	3.38
0	0	0	0	18.1	23.4	17.4	47.3	15.3	5.11
1	0	0	0	12.6	14.7	10.7	41.2	12.9	4.38
0	0	0	0	40.2	38.5	7.4	35.4	230.9	4.25
0	0	0	0	24.9	13.8	8.7	47.4	69.1	3.68
1	0	0	0	15.3	18.1	16.4	51	14.2	2.86
1	0	0	0	88.6	57.1	18.9	34.3	437.7	3.76
0	0	0	0	45.4	34.1	8.4	37.9	25.2	3.8
0	0	0	0	17.7	36.1	12.8	31.5	68.1	3.58
0	0	0	0	10.6	17.4	8.6	41.1	15	4.68
0	1	0	0	31.6	49.7	35.8	29.5	22.7	3.7
0	1	0	0	41.2	40.6	9.7	34.7	31.5	3.38
0	0	0	0	5.4	19.1	10.4	43.8	12.9	2.97
0	0	0	0	21.4	21	14.2	43.7	17.9	3.57
0	0	0	0	25.6	25.1	11.7	42	49.5	4.93
0	0	0	0	44.2	71.1	6.8	38.3	16.3	3.79

1	0	0	1	102	175	71.6	34.2	573.8	4.14
0	0	0	0	13.4	14.5	9.6	47.1	27.2	4.39
1	1	0	0	18.1	19.3	37.2	46.5	85.8	3.22
0	1	0	0	80.5	54.8	10.6	34.1	396.1	6.36
0	1	0	0	144	140	44.4	34	55.1	4.57
0	1	0	1	24.5	30.8	24.9	30.8	62.2	2.96
0	1	0	0	15.9	26	28.3	30.6	8.3	3.16
0	1	0	0	16.7	37.3	27.1	32.2	47.2	3.49
0	1	0	0	34	49.9	18.6	35.7	83.6	3.23
0	0	0	0	29.7	26.5	26.1	41.5	66.6	3.82
0	0	0	0	19.7	21.4	11.9	37.1	31.6	4.33
0	2	0	0	91.5	107.6	20.5	30	128.4	3.56
0	0	0	0	19.9	21.2	8.4	40.7	32.4	2.9
0	0	0	0	13.3	15.3	9.6	37.8	32.9	3.37
1	0	0	0	34.2	32.8	16	41	108.5	4.35
1	0	0	0	41.5	42	8.8	40.8	71.7	4.37
0	1	0	0	21.2	17.3	15.3	44.3	86.1	4.94
0	0	0	0	34.7	31.2	22.6	37.3	51.3	3.7
0	1	0	0	24.1	27.9	19.1	34.2	13.1	2.4
0	0	0	0	19	25	14.5	51.4	26.9	4.93
0	0	0	0	9.4	14.1	15.1	45.5	17.4	3.35
0	0	0	0	30.8	28.6	11.3	34.6	132.1	3.42
0	0	0	0	40.4	33.6	8.6	44.9	77.8	3.33
0	0	0	0	16.6	28.6	23.3	34.3	25.2	3.53
0	0	0	0	16.6	29.6	29.6	35.6	17.4	2.85
1	1	0	0	26.7	55.7	23.1	34.7	143.3	5.21
0	0	0	0	26.3	32.8	10	40.4	56.4	4.63
1	0	0	0	12.9	20.3	12.7	47.2	19	3.74
0	0	0	0	30.9	18.4	9.9	42.1	38.7	4.09
1	0	0	0	30.7	16.7	11.7	43.2	19.9	3.97
0	1	0	0	52.4	57.2	31.9	29.1	129.1	4.37
1	0	0	0	32.4	30	10.4	44.7	34.3	3.89
1	0	0	0	41.3	56.1	12.8	40	85.5	4.17
1	0	0	0	18.2	15.7	14.2	40.7	57	3.44
0	0	0	0	21.6	22.2	10.6	39.2	207.6	3.98
0	1	0	0	41.9	40.6	12.4	40.3	109.6	3.18
0	0	0	0	10	25	11.2	37.3	27.2	3.64
1	0	0	0	155.4	126	37	36	1110	3.25
0	0	0	0	36.3	44.2	7.1	40.4	31.3	3.88
1	0	0	0	16.2	27.4	17.9	34.9	14.5	3.87
1	0	0	0	13.1	13	10.4	39.4	28.2	3.63
1	0	0	0	23.3	16.5	17.6	40.6	24.7	3.86
0	0	0	0	214.5	124.9	13.9	39.1	65.6	4.06
0	0	0	0	88.9	78.9	9.8	38.7	47.7	4.91

0	0	0	0	41	22.9	11.2	44.3	24	2.64
0	0	0	0	27.2	17	10.2	41.4	15.6	4.04
0	0	0	0	27.7	25	13.6	40	38.8	3.31
0	0	0	0	23.8	25.4	12.8	42.7	67.1	5.05
1	0	0	0	13.5	9.2	8.2	43.9	21.8	3.22
1	0	0	0	42.1	52.7	21.2	35.2	141.2	4.05
1	1	0	0	23.5	48.7	24.6	29.7	56.9	3.38
0	0	0	0	27.8	22.9	11.2	50	17.1	5.65
0	0	0	0	23.7	20.7	10.2	41.3	34	3.36
1	0	0	0	35.4	30.2	14.7	42.1	30.6	4.02
0	0	0	0	60.3	38.8	12	45.9	58.1	3.4
0	0	0	0	7.8	12.9	14.7	39.8	8.1	2.86
0	0	0	0	77.7	71.5	23.7	37.2	86.6	3.64
0	0	0	0	25.5	20.1	13.6	43.2	19.5	4.66
0	0	0	0	29.4	37.3	10.1	43.1	37.8	3.41
0	0	0	0	19.6	17	19	49.4	28.1	2.83
0	1	0	0	19.7	18.7	20.4	25.3	13.7	2.31
0	0	0	0	9.9	16.2	17.3	38.3	24.4	3
0	3	0	0	13.9	49.4	74.3	27.9	268.4	4.86
1	0	0	0	69.5	57.1	8.8	46.3	180.4	4.14
0	3	0	0	29.7	36.6	23.3	21.7	76	3.4
0	0	0	0	16.6	29.3	15	35.9	239.2	5.17
0	0	0	0	16.2	27.4	14.9	41.1	22.6	4.63
0	0	0	0	17.8	18.9	32	44.9	33	4.34
0	1	0	0	28.5	45.9	32.3	37.6	251	4.93
1	0	0	0	28.2	27	14	37.6	49.1	4.32
0	2	0	1	17.1	18.7	20.4	27.5	49.3	2.32
0	0	0	0	35	26	14.5	47.6	14	3.27
1	0	0	0	11.3	14.1	37.1	34.3	21.4	2.9
1	0	0	0	29.3	23	24.4	43.7	64.8	5.42
0	0	0	0	18.1	23.3	11.2	42.6	41.3	4.3
0	0	0	0	72.7	111.9	34.3	30.6	82.4	3.77
1	1	0	0	31.5	27.6	13.1	40.4	72.5	3.59
0	0	0	0	41.3	38.3	15.9	38.1	26.8	3.51
0	0	0	0	42.9	35.3	15	36.8	41.9	3.65
0	0	0	0	25.9	28.7	9.3	54.6	18.9	2.87
0	0	0	0	17.2	19.3	14.2	41.5	35.4	3.27
1	0	0	0	29.8	21	16.8	50.7	20.6	2.87
0	0	0	0	24.1	20.8	21.7	50	28.2	4.54
0	0	0	0	19.5	37.1	36.7	41.1	35.6	4.09
0	0	0	0	14.6	19.3	22.7	39.8	31	3.31
0	0	0	0	13.7	17	10.9	43.2	36.7	3.62
0	1	0	0	27.7	32	34.1	38.1	21.7	4.43
1	0	0	0	36.5	65.5	23.8	34.7	1068	3.48

0	0	0	0	33.1	22.9	21.3	41.7	175	4.53
0	0	0	0	29.2	18	14.7	42.9	90.9	3.64
0	0	0	0	112.8	55.5	9	36.6	45.1	4.38
0	2	0	0	45.5	48.3	15.9	23.5	273.7	3.2
1	0	0	0	14.2	18.7	6.1	41.3	81.8	3.37
1	0	0	0	25.3	19.5	14.3	47.7	83.3	4.58
0	0	0	0	45.1	62	15.5	41.3	89.3	4.22
1	2	0	0	51.8	68.3	28.9	31.6	82.1	3.98
0	0	0	0	50.3	44.3	9.5	41.7	53.4	3.38
1	1	0	1	52.4	61.3	9.7	33.8	21.5	3.14
0	0	0	0	10.5	18.2	7.3	39.7	19.4	4.56
0	0	0	0	75.3	41.6	9.2	37.9	56.4	3.31
1	0	0	0	26.5	21.6	22.1	41.9	66.8	4.68
0	0	0	0	33.7	32.3	14.4	40.6	118.2	3.83
1	1	0	0	36.2	39.2	27.4	30.1	66.4	4.02
0	1	0	0	80.9	130.7	31.1	30.5	60.2	2.77
0	0	0	0	38.5	47.9	6.2	38.2	46.6	3.3
0	0	0	0	18.8	23.8	16.4	40.3	13.4	3.12
1	0	0	0	18.3	26.8	16.7	32.1	39.1	4.98
0	0	0	0	31.4	22.6	6	43	14.8	4.34
0	0	0	0	15.6	21.9	18.7	45.5	12.3	2.7
0	0	0	0	23.9	25.6	6.2	41.7	33.1	3.5
0	2	0	0	15.5	53.3	28	31.4	341.9	4.1
0	0	0	0	21.8	26.9	14.5	45.2	73.5	3.25
1	0	0	0	16.6	11.7	12.1	41.8	28.2	3.41
1	0	0	0	82.7	66.3	30.3	37.5	100.2	3.39
1	0	0	0	15.5	13.2	15.6	43.1	24.7	2.94
0	1	0	0	40.4	53.7	22.4	23.8	29.6	3.22
0	0	0	0	18.2	18	7.1	46.8	27.6	3.64
1	0	0	0	24.7	25.6	15	36.7	54.1	4.32
1	1	0	0	32.2	36.7	17.1	37.2	210.4	3.77
1	0	0	0	17.5	19.3	16.1	41.9	32.1	3.99
1	1	0	0	219.8	220	16.7	43.3	404.5	6.45
0	3	0	0	30.4	56	22.4	31.7	118.5	4.18
1	2	0	0	21.6	116.4	14.4	29.2	177.9	4.45
0	2	0	0	69.6	45.7	46.5	33.2	187.3	10.68
0	1	0	0	33.7	37.5	13.3	30	76	4.03
1	0	0	0	14.1	29.4	7.8	35.7	74.5	4.19
1	1	0	0	22.9	65.1	21.4	33.6	160.2	4.12
1	3	0	0	104.9	158.2	52.9	26.2	84.3	3.22
0	0	0	0	126.8	149.4	20.4	38.1	126.8	5.66
0	0	0	0	13.2	17.1	15.1	40.9	29.1	3.14
0	0	0	0	71.3	52.7	16.1	38.8	131.7	3.68
1	0	0	0	24.2	50.9	27.1	35.8	217.7	3.33

0	0	0	0	25.9	32.8	7.8	43	42	5.2
0	0	0	0	26.7	18.6	5.3	43.1	17.9	4.01
1	1	0	0	22.5	20.8	6.2	37.3	34.7	4.14
1	0	0	0	105.7	61.3	17.8	40.3	133	3.59
0	0	0	0	27.8	30.8	25.8	41.6	35	3.87
1	0	0	0	12.2	13.5	24.7	43	26.7	3.29
0	0	0	0	17.3	23.7	11.1	40.8	57.4	3.98
1	2	0	0	10.7	20.6	13.8	28.9	30.2	3.47
0	0	0	0	32	23.1	13	39.3	36.7	3.95
1	0	0	0	45.5	35.2	15.7	38.9	33	4.07
0	1	0	0	19.1	19.3	12.1	42.2	23.4	2.94
0	0	0	0	602.9	526.5	52.8	39.9	141.4	3.68
0	3	0	0	21.3	47.7	37.3	23.8	10.9	2.59
1	0	0	0	25.9	18.6	13.4	40.6	19.5	2.81
0	0	0	0	19.3	35.8	10.4	43.7	62.2	2.91
0	0	0	0	22.5	24.5	19.8	44.4	42	5.71
0	0	0	0	23.9	28.2	15.5	35.6	20.5	3.78
0	2	0	0	17.6	30.6	22.1	31.5	36.3	4.06
1	2	0	0	41.4	71	30.7	22.7	47.6	5.14
0	0	0	0	31.9	24.2	17.4	44.5	38.8	4.88
1	0	0	0	26.5	38.1	16.6	18.7	74.8	4.15
0	1	0	1	24.4	31.9	30	34.8	22.4	2.84
0	0	0	0	39.1	22.8	16.2	42.1	23.2	3.96
0	0	0	0	13.1	20.1	21	46.3	16.1	4.37
0	0	0	0	24.2	49.4	11.5	38.6	140.1	5.16
0	0	0	0	72.1	108.7	37.2	37.8	192.2	3.22
1	0	0	0	18.1	22.8	9.7	41.3	17	4.69
1	0	0	0	18.4	50	21	35.1	86	2.67
0	0	0	0	36.8	58.7	258.4	34.5	297.6	5.67
1	0	0	0	18.2	24.3	8.8	35.7	21.8	4.16
0	0	0	0	40.6	46.3	36.1	27.4	17.6	3.05
0	0	0	0	18.8	21.4	7.3	39.3	21.6	4.07
0	0	0	0	20.2	22.6	19.7	46.7	16.8	3.63
0	0	0	0	16.3	25.4	19.3	45.1	16.7	4.15
	0	0		21.8	36.5	53.7	39.9	40.8	4.38
0	0	0	0	58	33	14.3	42.6	17.5	3.45
0	0	0	0	28.9	22.1	16.9	46	26.2	5.31
0	0	0	0	43.5	46	15.9	33.7	268.3	4.67
0	0	0	0	13.7	19.7	10.9	41.6	16.9	3.92
0	1	0	0	35.2	27.3	11.3	36.5	40.7	3.29
0	1	0	0	44.4	62.5	27.8	31.6	50.9	4.11
0	0	0	0	22.3	16.6	17.6	36.5	109.7	4.59
0	0	0	0	28.8	27.2	28.9	47.8	19.4	4.75
0	0	0	0	19	22.2	17.5	39.2	17.8	3.79

1	0	0	0	19.3	24.7	16.5	39.7	88.6	2.74
0	0	0	0	9.5	12.4	11	41.5	15.8	3.49
0	0	0	0	30.1	63.7	14.2	47	65.9	3.84
1	0	0	0	25.9	32.3	20.4	40.8	18.3	3.71
0	0	0	0	22.1	28.4	24.5	41.9	69.8	4.46
1	1	0	0	17.9	28.4	12.6	34.5	66.5	3.71
1	0	0	0	14.8	22.6	15.2	38.5	23.8	3.3
1	0	0	0	41.4	28.6	14.6	41.4	95.6	4.61
1	1	0	0	14.4	40	12.9	35.5	85.1	4.77
1	1	0	0	21.6	52.2	12.8	22.4	36.6	4.42
	0	0	0	47	30.1	8.3	44.1	32.3	4.37
0	0	0	0	49	38	14	44.1	43.2	3.8
0	0	0	0	46.2	40.2	15.9	43.3	35.3	4.32
1	1	0	0	55	47.8	13.1	23.7	103.9	3.11
0	1	0	0	13.6	16.2	11.6	39.4	14.1	2.98
0	0	0	0	27.1	21.8	8	38	54.2	3.15
0	0	0	0	36.1	38.9	33.7	37.2	111.6	2.72
0	0	0	0	19.1	27	13	45.1	29.2	3.12
0	1	0	0	24.6	25	15.7	30.9	106	4.8
0	1	0	0	12	37.6	59.9	36.9	20.7	3.26
0	0	0	0	10	32.8	14.5	33.9	16.3	2.61
0	0	0	0	10.2	18.8	13.7	41.7	11.5	3.43
1	2	0	1	8	13.8	17.1	28.2	18.7	2.4
1	0	0	0	27.4	32.8	18.6	40.3	76.7	4.34
1	0	0	0	15	22.3	13.9	36.4	27.4	3.36
0	0	0	0	24.2	21.3	10.1	49.7	28.7	4.76
0	1	0	0	14.5	16.7	11.9	44.1	21.7	4.75
1	1	0	0	14.4	51.9	8.7	39.1	130.4	3.41
0	1	0	0	28.1	45.1	83.4	23.9	16.6	1.09
0	0	0	0	26.3	21.8	17.8	37.2	46	3.27
0	0	0	0	27.4	30.8	21.9	37.8	22.3	2.1
1	1	0	0	25.8	33.8	13.7	26.6	237.8	5.46
0	0	0	0	21.9	22.8	13.6	46.7	26.5	5.04
0	0	0	0	15.7	13.4	10.3	40.8	38	3.45
0	0	0	0	19.9	25.4	18.8	47.2	12.5	4.29
0	0	0	0	37.8	41.8	10.7	39.8	130	4.78
0	1	0	0	21.8	33.6	30.7	38	51	4.05
0	0	0	0	20.1	28.2	34	32.3	18.4	3.05
0	0	0	0	34	25.2	14.2	53.8	76.6	3.86
0	0	0	0	31.6	30.6	21.7	38.9	41.4	3.52
0	0	0	0	13.7	19.5	10.4	47.5	16.3	5.36
1	0	0	0	27.9	21.7	12.2	39.9	14.7	2.67
1	0	0	0	19.9	19.6	13	40.5	54.5	3.55
0	0	0	0	23	29.1	23.7	49.6	26.4	3.57

1	0	0	0	34.8	23.8	31.5	41.9	17.7	2.49
1	0	0	0	43.8	34.8	16	33.5	129.1	4.15
0	0	0	0	19	23.9	11.8	47.4	71	3.43
1	1	0	0	24.1	34.4	30.3	35.8	18.4	3.74
0	0	0	0	32.2	25	30.7	40.6	85.6	3.39
0	0	0	0	27.9	21.1	18.8	39	16.4	3.44
1	0	0	0	12.2	15.8	10.2	43.9	18.2	3.79
0	0	0	0	17.3	18.8	66.6	42	18.3	2.67
0	0	0	0	24.7	21.2	15.7	44.3	39.7	2.69
0	0	0	0	32.8	24.4	29	36.8	23.4	2.81
0	0	0	0	70.2	281.9	44.2	38.5	498.6	3.44
0	1	0	0	74.8	91.2	72.8	24.3	49.3	3.03
1	0	0	0	14.8	17.5	10	42	46.2	4.58
1	0	0	0	18	20.6	16	39.4	19.6	5.36
0	0	0	0	15.5	16	6.3	40.9	42.1	5.14
0	1	0	0	21	23	13.3	37.9	29.4	2.49
0	1	0	0	11.2	16.1	17.2	39.2	10.3	2.57
0	0	0	0	19.8	28.5	12.3	31.7	38.8	4.08
0	0	0	0	169.9	81.5	26.9	43.2	190.2	3.33
0	0	0	0	13.7	21.7	10.8	40.7	9.3	3.51
0	0	0	0	32	39.4	13.9	35.7	136.7	3.95
1	0	0	0	19	20.2	12.7	43.8	15.1	2.72
0	0	0	0	22.7	12.6	11	43.5	88.5	3.86
0	0	0	0	19.9	25.1	14.4	38	16.6	4.07
0	0	0	0	20.1	21.3	24.2	30	77.7	2.67
1	0	0	0	24.3	25.4	26.2	31.8	38.8	3.57
1	0	0	0	16.9	38.6	36.3	35.1	41.4	4.08
0	0	0	0	29.7	34.1	22.3	37	22.5	1.99
0	0	0	0	27.3	46.3	15.8	39.5	42.3	3.56
1	0	0	0	27.8	54.7	37.9	27.7	18.3	2.89
0	0	0	0	477.8	303.3	19.1	39.6	51.6	3.73
0	1	0	0	8	15.1	21.7	41.6	12.4	3.44
1	0	0	0	16.4	19.3	13	44.5	45.7	5.14
0	1	0	0	28.8	54	16.5	28.7	187.3	3.04
1	0	0	0	28.7	32.9	25	38.6	12.1	3.42
0	0	0	0	24.2	26.8	23.9	42	21.4	2.66
0	0	0	0	9.8	18.7	7.1	38.7	17.8	3.92
1	0	0	0	21.6	24.4	13.9	43.7	48.8	3.58
0	0	0	0	91.6	66.1	19.9	43.5	57.2	4.12
0	0	0	0	25	31.2	50.5	28	18.5	3.21
1	1	0	0	34.8	42.9	37.1	25.8	78.7	3.6
0	1	1	0	41.5	54.2	24.5	33.7	49.5	3.56
1	1	0	0	40.1	110.4	37.9	26.1	78.5	3.21
1	1	0	0	61.4	120.4	172.8	32.6	403.7	4.36

1	0	0	0	18.4	26.6	25.1	35.3	10.5	4.38
0	1	0	1	43.7	50.2	23.3	26	28.9	1.76
1	1	0	0	25.5	43.1	13.4	28.9	90.5	4.56
0	1	1	0	10.7	22.8	22.9	31.5	8.9	2.32
0	0	1	0	18.9	35.9	21.6	30.5	55	3.64
0	1	2	0	14.4	31.3	29.5	28.5	25.1	2.11
1	1	0	0	30.4	50.5	73	23.5	39.5	3.99
1	1	1	0	40	58.4	36.3	21.5	14.9	2.9
0	3	0	0	19.3	49.9	27	24.9	17.3	2.42
0	1	0	0	12	26.5	42.4	27.7	21.5	3.05
0	0	2	0	44.2	26.5	11	33.7	42.2	2.59
0	3	1	1	51.5	45.2	8.9	35	20.8	3.23
0	3	2	0	13.2	55.8	68.7	25.1	221	4.95
0	1	1	0	38.8	39.6	34.5	25.2	31.7	4.92
0	1	1	0	28.5	59.2	48.2	22.8	45.3	2.48
0	1	1	0	19.4	43.4	54.7	33	77.1	2.83
0	2	0	0	220.3	327.7	45.1	32.1	846.4	5.42
0	3	0	0	93.9	148.9	38.6	24.6	18.3	3.9
0	2	0	0	54.6	113.8	34.3	16.1	115.6	5.06
1	1	1	0	86.9	99.7	27	28.6	300.7	4.35
0	1	0	1	24.9	47.5	20.3	31.8	43.4	2.4
0	1	1	1	40.2	51.5	11.8	24.7	82.8	2.58
0	1	0	1	39.9	41.6	56.1	36	121.3	4.51
0	1	0	1	18.8	20.2	5.5	29.6	17.7	2.91
0	1	0	1	14.7	26.9	16.6	25.7	26	2.35
0	2	0	0	19.1	26	21.4	39.1	11.1	2.78
0	1	0	0	26	28.4	9.5	36.8	21.6	3.66
0	1	0	0	91.6	206.8	28.7	33.2	254.8	4.41
0	0	0	0	18.3	25	10	35.6	13.4	2.92
0	0	0	0	8.7	16.9	23	51	12.6	2.74
0	1	0	0	17.8	27.8	29.2	38.9	21.2	3.68
0	0	0	0	108.6	472	58.8	32.5	130.9	5.36
0	0	0	0	27.2	31.4	10.1	39.8	24.8	3.68
0	0	0	0	29.7	25.8	15.9	42.2	24.1	3.63
0	0	0	0	23.8	94.1	21.2	37.5	64.1	2.58
1	0	0	0	21	45.9	11.2	36	308	4.68
0	2	0	0	17.6	40.4	17.9	33.9	103.2	3.46
0	0	0	0	10.3	30.8	10.7	37.4	53	3.39
1	0	0	0	29.2	35.4	13	42.8	58.1	4.52
0	0	0	0	34.7	18.8	7.9	41.9	129.2	5.61
0	1	0	0	46.2	127.3	27.6	32.9	107.5	3.22
0	0	0	0	97.1	54.6	18.1	33.6	446.8	4.57
1	1	1	0	29.3	39.3	19.5	32.5	12.8	1.73
0	3	0	0	101	146.6	155.5	27.9	36.7	2.83

0	0	0	0	19.7	25.5	10.7	40.1	25.3	3.83
1	0	0	0	66.9	62.3	18.6	40.9	74.4	4.81
1	0	0	0	11.5	30.3	6.7	34.7	39.1	3.26
0	0	0	0	17.8	23.2	7.1	38	13.1	3.24
0	0	0	0	46.2	56.9	19.8	40.3	77.9	4.43
0	1	1	0	75.3	101.6	19	38.1	132.3	5.5
0	1	0	0	43.9	66.3	14.6	34.5	53	3.91
0	0	0	0	21.9	21.1	9.1	43.8	19	2.95
1	0	0	0	28.3	29.3	10.4	29.8	161.5	2.65
0	0	0	0	9.6	18.9	25.9	42.3	25.8	3.51
1	2	0	0	43.8	46.4	12.2	36	64	4.18
0	1	0	0	37.6	33.4	11.7	23.3	204.7	2.23
1	0	0	0	34.2	24.2	14.9	47.8	56.6	4.3
0	1	0	0	16.2	23.5	12	37.8	40.5	4.87
0	1	0	0	33.3	51.1	48.9	33.3	126.9	4.65
0	0	0	0	111.7	60.4	45.3	37.9	47.5	3.65
0	1	0	1	29.3	30.5	13.8	38.3	44.2	3.71
0	3	0	1	63.8	44.9	29.8	28.8	78.8	3.08
0	1	0	1	13.1	18.5	18.2	41.5	21.5	2.93
1	1	0	0	17.2	40	34.6	27.2	180.5	2.39
1	3	1	1	49.1	60.6	108.1	29.2	270.7	3.47
0	1	0	0	15.9	18.9	26.9	40.7	27.1	2.55
0	2	1	0	14.4	35.8	48.7	26.1	30	2.53
0	1	0	0	23.3	43	19.8	33.4	276.5	4
1	1	0	0	49.2	46.8	12.4	28.1	36.7	2.92
0	1	0	1	32.3	189.9	52.4	29.1	175.4	1.48
0	2	0	1	54.1	56.7	357.4	32.1	37.2	2.44
0	2	0	0	73	222.1	282.8	30.9	434.3	3.14
0	1	3	1	25.6	70.2	34.8	32.3	193	3.34
1	2	1	0	77.3	564.5	74.6	39.8	250.9	4.07
0	2	2	0	283.1	344.5	399	28.2	163.1	2.4
0	0	1	0	84.1	82.4	603.5	33.3	1287.3	9.87
0	3	0	0	68.6	153.5	68	35	629.9	4.2
0	2	0	0	66.7	95.2	15.8	36.4	100.1	4.03
0	0	0	1	16.5	20.6	15.9	42.4	9.6	1.89
0	2	0	1	78.3	181.4	38.6	24.6	126.7	2.11
0	1	0	0	22.4	40	42.4	27.7	34.1	3.96
0	1	0	1	39.3	119.8	49.9	31.7	254.9	3.84
0	1	2	0	20.2	37.8	25.4	24	131.3	2.88
1	2	3	1	36	117.1	31.9	40.7	369.4	4.3
0	1	0	1	51.8	50.1	7.2	26.8	22.1	1.37
1	3	3	0	45.3	52	29.9	44	261.5	3.35
0	2	0	0	38.3	55.2	75.1	29.6	39.2	3.44
0	0	0	0	59.5	90.1	486.6	29.2	611.2	13

0	1	0	0	479.1	519.9	413.1	34.7	84.9	2.91
0	1	0	0	28.7	35.8	9	40.1	46.6	2.78
0	2	0	0	13.8	64.9	31.3	29.5	157.1	3.71
0	2	0	0	36.1	52.2	39.2	33.7	26.1	3.22
0	3	0	1	70.3	196.7	102.8	29.3	50.9	1.74
0	2	0	0	20.1	24.1	20.2	34.6	17.3	1.81
0	3	0	0	274.3	405.3	116.2	34.8	135.3	4.28
0	3	0	0	12.7	50.8	6.4	35.2	55.6	3.3
1	1	0	0	27.2	87.5	29.3	37.4	110.6	4.09
0	2	0	0	172.3	147.4	127.1	26.9	39.5	2.66
0	1	0	0	53	74.5	77.7	23.1	33.1	1.99
0	3	0	1	51.8	124.7	64.4	32	82.8	2.83
0	2	0	1	24	39.9	29	29.1	182.8	3.76
0	2	0	0	12.5	33.8	52.2	29.4	20.7	2.69
0	2	3	0	203.6	639.8	434.6	23.5	173.5	2.03
0	2	0	0	23.9	30.7	17.9	31.5	10.8	2.46
1	2	4	1	147.4	731.7	102.8	40	289.8	3.36
0	3	2	1	111	113.9	40.9	35.7	85	2.51
1	1	0	0	32	34.9	10.1	37.2	34.6	2.34
0	2	2	1	31.9	132.1	435.5	23.7	140.9	1.95
0	1	0	0	34.9	49.3	31.9	29.9	93.9	3.67
0	2	0	0	74.6	113.7	32.9	25.8	74.7	3.17
1	3	0	0	14.1	32.5	9.1	34.7	48.2	2.6
0	2	0	0	67.5	82	35.7	30.1	115.7	3.21
1	2	0	1	31.2	68.3	110.2	33	159.9	2.89
0	3	0	0	56.6	51.9	35.7	32.7	95.2	2.57
0	1	0	0	61.1	60.2	30.4	35	136	2.72
1	1	0	0	14.1	28.7	46.2	28.6	41.8	2.07
0	2	1	0	187.1	373.6	323.1	44	755.5	3.69
0	2	0	1	32.6	46.3	91	26.7	58.6	2.02
0	1	3	0	45.4	85.6	12.9	34	134	4.21
0	2	2	0	94.3	259.9	128.6	36.9	533.5	5.49
0	3	2	0	35.5	94	49.1	40.7	373.8	3.53
0	1	0	1	34.5	37.8	18.6	31.1	19.6	2.66
0	2	0	0	20.2	40.9	28.2	27.5	12	2.27
0	1	0	0	22.9	30	17.9	48.7	65.6	4.22
0	1	0	0	65.7	448.3	23.7	29.7	374.5	3.61
0	1	0	1	11.9	16.6	17.5	26.7	175.5	1.68
0	1	0	1	106.3	72.5	34.5	41.2	108.5	3.83
0	3	1	0	48.1	255.2	393.9	27.8	92.3	2
1	2	0	1	27.4	38.7	17.4	32.7	40.5	2.5
0	1	0	0	33.2	61.5	23.9	25.5	42.1	2.74
	2	0	0	16.1	45.3	52.5	26	22.8	3.02
0	1	0	0	46	427.1	274.6	23.8	205	1.61

1	1	0	0	22	67	35.6	33.2	312.9	3.61
0	0	0	0	36.5	81.1	20.7	40.5	331.8	5.74
1	0	0	0	51.2	18.7	29.8	38.2	448.2	5.74
0	2	0	0	102.5	317.1	155.5	29	505	3.18
0	2	2	0	20.6	286	36.4	35.5	422	4.25
0	3	0	0	58.7	165.5	58.7	27.9	414.6	4.13
0	2	0	1	23.8	34.3	9.6	38.6	63.5	2.36
0	1	1	0	160	324.9	314.5	26.7	37	1.09
0	2	0	0	44.7	58.6	72.9	30.2	31.5	2.85
1	2	0	0	58	67.3	35.9	42.3	59.5	4.07
0	3	0	0	50	133.2	46.6	24	93.4	3.3
0	1	3	1	285.3	255.9	63.4	27.1	60.5	2.37
0	2	0	0	161.2	633.6	130.3	32	535.4	2.92
0	3	0	0	29.1	52.5	93.4	23.7	16.9	1.77
0	2	0	0	39	39.3	11.9	24.3	213.1	3.92
0	1	0	0	261.9	346.7	81.8	34.2	153.6	3.58
0	2	0	0	526.1	823.7	195.4	30.6	353.5	2.73
0	1	0	0	160.2	169.9	334.6	28	327.3	4.93
0	2	0	0	46.7	121.3	269.3	31.9	408.5	3.88
0	1	0	0	38	77.6	19.8	27.9	106.3	3.82
0	1	0	0	43.8	38.4	23	41.1	86.6	3.04
0	1	0	1	39.2	73.9	45.5	32	111.2	3.24
0	2	3	1	419.7	1071	91.9	23.7	14.2	0.66
0	1	0	0	46.1	52.4	35.7	33.6	35.9	3.38
0	3	0	0	107.4	152.9	332.4	21.6	527.7	2.68
0	1	3	0	80.5	191.1	69.3	28.5	37	3.62
0	2	0	0	50.5	170.6	25.9	29.2	257.1	3.31
0	0	0	1	58.1	54.3	20.1	22.9	21.5	2.1
0	2	0	0	21.4	71.1	37.8	16.3	43.2	3.07
1	1	0	0	37.9	93.8	276.5	26.4	501	3.76
1	2	0	1	71.8	89.4	28.7	37.8	18.6	2.5
1	2	0	0	417.9	928.5	38.1	34.3	126.5	3.15
0	3	2	0	62.2	139.5	351.2	28.9	36.9	2.41
0	0	0	0	26.3	63.2	71.3	28.7	174.5	3.01
0	2	0	0	57.3	115.1	68.2	22.2	318.8	3.57
0	2	0	0	39.8	41.4	48.9	35.3	68.2	3.56
0	3	0	1	27.4	34.6	16.4	27	54.7	1.92
0	1	0	0	66.2	80.3	44.9	30.5	55.8	3.25
0	1	3	0	37.6	132.3	22.4	36.1	165.7	3.22
0	2	0	0	37.8	58.3	26	29.6	115.9	3.78
0	0	2	0	39.2	59.4	82.8	29	14.7	3.25
0	0	0	1	27.4	28.9	15	28.4	33.7	2.88
0	2	0	0	136	242.1	74.1	30.8	18	4.65
0	1	0	1	41.7	43.1	13.3	39.1	206.9	2.52

0	2	0	1	21.9	51.5	29.8	21.6	12.5	2.5
0	1	0	0	32.8	69.1	29.9	24.9	35.8	3.21
1	0	0	0	101	96.8	43.9	37.7	105	3.83
0	2	0	0	27.3	41.8	23.2	29	11.3	2.75
1	1	0	1	54	219.6	615.3	26	309	1.59
0	0	0	0	34.4	30.4	35.5	40.8	77.2	4.46
0	1	0	0	35	87.3	74.5	29.1	136.9	3.72
0	2	0	0	21.3	47.7	37.3	23.8	10.9	2.59
0	1	1	0	60.1	175.5	534.8	26.1	320.6	3.07
0	1	0	0	27.6	58.3	32.6	36.4	344.7	4.19
0	3	0	0	33.2	228.2	48.4	29.7	394.8	5
0	3	0	0	45.1	48.1	26.7	28.2	122.4	3.16
1	1	0	0	21.8	55.8	24.3	32	56.2	3.53
0	0	0	0	98.2	187.6	118.3	46.4	961.5	5.81
0	2	0	0	54.5	131	42	29	71.1	3.09
0	3	0	1	116.5	1130.9	35	24.2	109.5	4.13
0	2	0	0	771.1	435.6	552.8	31.2	103.3	2.05
0	2	0	0	156.9	515.2	564.2	27.6	280.1	3.48
0	2	0	1	117.9	148.4	47.1	28.5	272.1	3.78
0	1	0	0	74.4	184.9	35.4	37	15.8	4.5
0	1	0	0	23.7	33.3	29.9	27.2	21.2	3.55
0	1	0	0	30.6	42	47.3	27.6	138.4	3.15
0	3	0	1	47.6	204.7	566.5	22.8	158.7	1.07
0	0	0	0	52.2	71.5	441.5	33.4	134.7	3.73
0	1	0	0	48.2	99.1	34.5	28.1	413.5	3.05
1	0	0	0	42.2	60.9	30.8	32.7	120.1	3.54
0	1	0	0	57	131.7	49	30.2	256.7	4.68
0	1	0	0	20.1	219.4	21.8	28.6	195	5.64
0	3	1	0	24.9	68	31.8	30	193.6	2.97
0	0	0	0	167.9	132.4	41.9	41	185.4	3.73
0	0	0	1	20.3	21.5	40.9	34.4	13.3	2.8
0	0	0	0	28.9	46.2	32.9	30.5	279.2	2.32
0	2	0	0	63.4	109.8	171.9	23	30.3	1.54
0	3	0	0	26.5	103.3	104.4	22.1	110.4	1.77
0	2	0	0	96.9	115.8	213.1	32.1	419.2	4.17
1	2	0	0	32.5	52.1	8.5	25.8	37	3.99
0	2	0	0	40.3	45.8	17.3	29.3	121.6	3.97
1	0	0	0	21.7	55.9	15.2	30.8	457.8	5.51
1	2	0	0	100.1	144.1	48	19.5	154.2	5.17
0	2	0	0	383	186.5	81.8	26.6	305.8	5.22
1	2	0	1	130.6	129	33.8	33.9	322.5	3.44
0	2	0	0	34.5	52.2	37.4	30.9	105.2	3.45
1	2	0	0	54	149.2	552.2	25	113.7	1.59
0	1	0	0	280.9	479.1	1064.1	40.2	519.7	3.71

0	2	0	0	33.4	39.8	49.9	29.5	32.8	2.65
0	0	0	0	23.8	120.3	648.3	25.8	182.3	1.93
0	3	0	0	27.6	104.2	64.3	25.3	83.7	3.38
1	3	0	0	149.1	201.6	561.3	29.5	138.7	3.08
0	2	0	0	43.9	110.9	77.9	33.7	81.6	2.84
1	2	0	1	63.9	135.3	12.5	22.9	98.1	1.94
0	0	0	0	36.4	26.5	18.5	42.5	63	3.31
0	1	0	0	25.1	31.9	29.4	41	18.5	2.88
1	3	0	0	86.9	155.1	207.6	26.9	319.6	3.73
0	2	0	0	45.1	102	20.4	27.8	23.5	3.11
0	1	0	0	45.5	63.6	20.9	31.6	65.9	3.78
1	2	0	0	19.3	27.6	13.7	31.2	14.8	3.22
1	2	0	0	63.1	167	26.8	28.1	117.2	3.48
1	0	0	0	18.7	50.7	16.1	29.8	72.9	4.34
1	3	0	0	28	78.3	35.5	27.6	188.9	6.17
0	2	0	0	31.3	87.3	49.8	24.7	93.3	2.33
0	2	0	0	46.7	166.6	83.3	25.9	211.1	2.29
0	3	0	0	17.7	27.9	30.5	23.6	18.5	1.63
1	2	0	0	63.4	129.2	50.8	26.4	284.6	3.4
1	3	0	0	38.5	83.4	24.4	27.5	58.6	4.45
1	3	0	0	26.7	93	53.3	23.5	44.8	2.72
0	3	0	0	45.4	67.1	382.3	27.9	77.8	1.94
1	2	0	0	86.6	76.8	30	29.6	93.6	3.95
0	2	0	0	72.1	76.5	28	25.3	54.5	2.39
1	2	0	0	78.6	60	17.3	32.2	261.6	3.99
0	2	0	0	63	80.7	29.1	34.6	155.3	5.12
0	3	0	0	71.1	110	55.8	37	270.9	3.94
0	1	0	0	33.7	29.1	23.2	39.5	103	3.69
0	1	0	0	59.1	45.7	23.7	38.2	30.6	2.38
0	2	0	0	229.6	627.7	424.1	23.8	291.1	2.23
0	0	0	0	10	22.5	7.6	39.6	24.4	3.24
0	1	0	0	30.5	38.6	15.6	34.8	117.7	2.92
0	0	0	0	58.6	30.7	24.2	42.2	143.6	3.91
0	3	0	0	29	147.1	120	29.5	771	4.04
0	2	0	0	26	108.1	59.1	24.5	303.4	3.59
0	1	0	0	56.4	63.3	22.5	33	20.4	3.13
0	1	0	0	62.8	80.2	196.2	35.8	345.9	7.13
0	0	0	0	49	64.9	46.5	24.6	33.2	3.93
1	1	0	0	30.6	74.2	12.6	39.1	90.5	5.31
1	3	0	0	53.6	140.5	67.1	18.8	80.3	3.75
0	2	0	0	106.9	268.4	153.8	28.6	336.8	4.32
1	0	0	0	100.3	121.7	15.1	42.7	315.9	4.52
0	1	0	0	98.9	177.9	27	37.2	171.8	3.32
0	0	0	0	17.5	29.4	25.2	26.6	15.2	2.44

0	2	0	0	58.6	196.9	100.6	25.7	621.4	3.34
1	2	0	0	106.1	203.6	33.2	34.5	222.4	3.6
0	0	2	0	16.6	26.7	20.7	32.2	40.3	3.19
1	0	0	0	14.1	21.9	13.9	40.2	18.5	4.09
0	1	0	0	297.7	779.2	78.4	33.6	283	2.44
0	1	0	0	19.4	37.7	31.1	28.2	36.8	3.04
0	1	0	0	155.3	262.3	376.9	37.2	190	2.19
1	2	3	0	19.7	23.7	18	27	51.7	2.72
0	3	0	0	21.5	89	32.4	32.4	803.7	2.98
0	1	0	0	56.9	76.4	18.3	31.7	64.5	3.98
0	2	0	0	68.6	111	99	34.5	186.4	5.14
0	0	0	0	29.1	76.6	20.2	32.5	89	4.75
0	1	0	0	20.1	54.9	38.9	33	179.5	3.62
1	1	0	0	9.5	18	63.9	26.6	65.2	1.65
0	1	0	0	26.8	44.9	52.1	25.9	27.5	3.48
0	0	0	0	44.1	26.6	6.6	40.7	87.1	3.96
0	2	0	0	178.7	258.2	41	26.4	132.8	3.43
0	0	0	0	63.5	42	266.6	36.2	112.9	6.61
0	3	2	0	48.7	74.1	183.7	28.5	54.8	2
0	2	0	0	39.1	95	47	27.6	40.9	3.08
0	1	0	0	96.1	133.8	262.5	34.6	319.4	3.3
0	3	0		54.8	88.7	55.1	28.2	90	2.91
	0	0	0	37.8	27.8	19.7	39.1	144.6	4.92
0	0	0	0	23.5	79.1	22	44	17.7	6.14
0	0	0	0	11.5	19.3	24.9	28.8	15.2	1.91
0	2	0	0	50.9	52.1	21.7	17.3	33	3.57
0	2	0	0	24	39.5	56.5	24.3	23.5	1.83
0	2	0	0	45.7	87.1	69.4	31.6	153.9	3.66
0	0	0	1	22	23	15.5	35.5	22.1	3.03
0	0	0	0	71.4	77.2	42.8	31.2	41.4	3.2
0	2	0	0	701.4	688.7	49.6	46.3	179.9	3.59
0	1	0	0	57.9	72.6	30.2	32.9	21.9	4
0	2	0	0	60.2	59.3	20	32.9	245.9	3.53
1	2	0	0	13.9	45.7	15	35.7	100.9	2.57
0	2	0	0	40	61.1	41.9	25.9	51	2.42
0	2	0	0	123	204.2	101.6	31.2	58.5	2.91
0	2	0	0	32	29.8	12.2	34.3	46	3.44
0	2	1	1	92.4	154.5	59.9	31.3	446.5	2.13
0	2	0	0	124.6	227.4	38.7	36.8	435.8	8.7
0	3	0	0	63.2	152.4	106.8	26.5	33.4	1.82
0	3	0	0	40.3	48.3	28.5	32	43.9	4.88
0	1	0	0	25	28.1	34.3	30	64.8	4.07
1	1	0	0	31.3	18.6	12.4	28.8	258.7	3.96
0	2	0	0	63.6	283.3	20.7	23.7	28	2.42

0	2	0	1	13	93.4	691.5	32.7	30.8	3.67
0	3	0	0	40.7	190.3	49.9	29	57	3.03
0	0	0	0	59.9	36.7	372.9	30.6	104.7	4.72
0	2	1	0	39.9	60.2	89.9	25.9	31.9	2.47
1	3	0	1	8.3	45.9	4.6	10.9	263	1.92
0	3	0	0	63.6	89.1	15.8	37	207.8	4.08
0	0	0	0	224.1	151.5	35.7	42.6	271.5	4.41
0	2	0	0	37.8	70.9	86.9	25.5	164.3	4.09
1	1	0	0	44.9	85.5	40.3	29.4	165.6	3.27
1	3	0	0	19.1	54.5	28.4	19.2	31.8	2.11
0	0	0	0	31.1	27.5	18.2	43.8	74.3	5.04
1	0	0	0	12.8	18.9	13.7	35.6	36.6	3.87
0	1	1	0	16.2	24.2	22.3	25.8	61.3	3.04
1	0	0	0	97.8	94.7	9.8	42.5	179.3	3.69
0	2	0	0	44.4	91.1	23	30.8	352	3.57
0	2	0	0	76	63.6	23.8	39.9	158.4	2.52
0	3	0	0	37.3	111.1	41	21.7	102.3	2.08
0	2	0	0	68.1	116.3	32.7	28.2	107	4.14
0	2	0	0	142.3	301	26.8	31.4	11.1	3.1
1	0	0	0	27.9	33.9	2.7	32.3	79.4	3.91
0	0	0	0	254.9	128	152	37.2	541.2	3.31
0	2	0	1	12.8	59.1	13.4	27.8	151.2	2.44
0	1	0	0	14.7	31.2	78.1	28.5	39.5	1.91
0	0	0	0	575.7	575.7	51.2	35.4	11.8	3.75
1	0	0	0	18.2	39.3	15.3	36.1	39.1	4.85
1	3	0	0	108.4	167.7	26.6	24.2	192.1	5.01
0	3	0	1	27.1	48.4	36.4	26.1	19.6	2.6
0	2	0	0	29.8	40.4	22	28.8	63.3	2.77
1	1	0	0	50.5	47.7	17.2	37.9	117.5	4.35
0	3	0	0	33.8	64.1	109.1	19.8	18.9	1.3
0	1	0	0	40.8	104.9	22.1	31.7	180	4.34
0	3	0	0	20.5	66.5	24.4	40.9	92.9	3.98
0	2	0	0	521.5	1803.3	250.6	30.4	309.5	3.08
1	2	0	0	19.1	36.1	9.1	37.4	70.2	3.3
1	1	0	1	11.9	14.8	30.8	27.7	15.3	1.92
0	0	0	0	31	62.3	40.6	36.3	268.1	3.94
0	0	0	0	116	81	15.1	41.5	91.6	4.03
0	0	0	0	46.1	35.4	21.6	39.3	49	3.28
0	0	0	0	30.2	39.3	25.6	36.2	164.8	4.86
1	2	0	0	27.6	39	16	39.2	8.8	4.95
0	1	0	0	26	44.6	20.9	29.6	27.7	5.24
0	2	1	1	53.9	68.9	35.4	21.1	159.8	1.74
1	2	0	0	301.3	486.8	27	34.4	46.8	4.31
0	0	1	0	479.4	531.9	190.1	20.6	20.2	2.73

1	1	0	0	39	23.5	9.7	30.3	55.9	3.44
0	0	0	0	151	83.7	234.2	38.9	496	6.56
0	0	0	0	77.6	50.7	20.9	45.1	22.8	3.28
0	0	0	0	17.5	19.4	18.8	40.3	34.1	3.04
0	1	0	0	27.3	33.3	31.2	36.9	24.4	3.93
0	2	0	1	17.1	26.6	16.9	25.2	17.9	1.94
0	3	0	0	48	208.1	125.5	31.1	81.7	3.93
	2	0	1	17.7	130.9	29	29.4	132.8	3.54
0	3	0	0	35.2	63.5	35.6	32.6	24.3	3.6
0	0	0	0	41.9	37.7	23.4	42.8	22.7	2.53
0	2	0	0	52.7	54.8	81.5	29.9	23.2	3
0	2	0	1	32.7	71.5	193.5	19.7	25.4	1.22
1	1	1	0	87.5	459.1	58.5	26.9	466.1	3.71
1	1	0	1	19.3	35.4	20.8	24	64.5	2.75
0	0	0	0	23.5	26.6	13.7	44.1	132.6	4.08
0	1	0	0	21.6	71.7	54.8	22.1	136.3	3.66
0	2	0	0	48.9	93.5	36.8	21.6	58.1	2.79
0	0	0	0	94.6	77	15.9	35.2	84.1	4.19
0	0	0	0	283.4	198.7	18.5	37.2	204.3	4.25
1	2	0	0	15.2	27.6	14.8	27.8	73.9	3.6
1	1	0	0	42.7	160.8	45.6	31.1	296.7	4.16
1	1	0	0	14.6	15.7	13.3	31.5	15.3	2.46
0	2	0	0	79.4	216	414	22.4	127.7	1.37
0	2	2	1	53	60.6	42.6	23.5	20.8	2.64
1	2	0	0	13.5	37.7	12.1	27.9	215.8	3.34
0	0	0	0	16.8	24.2	10	38.3	23.9	3.53
0	1	0	0	41.1	35.5	21.1	34.8	35.5	3.14
0	0	0	0	35.6	28.7	15.1	46.3	91.2	7.77
1	0	0	0	9.6	28.4	11.5	38.9	29.1	3.98
0	2	0	1	53.8	92.6	37.1	33.5	17.5	3.57
0	1	0	1	20	39	78.3	31	141.8	3.15
1	1	2	0	39.9	65.3	38.2	22.9	37.5	3.66
1	0	0	0	6.8	21.5	10.1	38.5	90	2.71
1	0	0	0	36.3	33.1	14.8	42.6	23.8	4.19
1	3	0	0	20.6	30.5	12.4	29.3	28.9	2.97
1	2	0	1	62.2	110.9	43.2	31.9	221.6	5.4
0	1	2	0	23.4	29	26.5	33.1	45.4	3.05
0	1	0	0	10.8	23.7	18.8	31.8	21	2.27
0	2	0	0	260.5	211.7	24.6	38.5	660.1	3.62
0	2	3	0	26.1	49.6	39	30.3	31	2.44
1	1	2	0	81.6	269.6	32.3	28.4	10.5	2.01
0	2	0	0	46.1	143	37.7	33.9	148.4	2.5
0	2	0	0	180.4	280.3	39.7	25.1	67.2	2.86
0	0	0	0	21	31.6	11.2	45.6	127.2	5.64

1	1	0	0	31.4	52.2	12.4	32.9	90.5	4.64
0	0	0	0	138.7	84.4	24.6	38.9	307.9	4.17
0	0	0	0	116.5	64	12	38.9	130	3.23
0	2	0	1	54.3	119.5	20.5	34.6	31.1	2.42
0	3	0	0	24.4	46.1	40.9	24	99.3	2.25
1	0	0	0	24.6	31.5	9.9	33.5	42.7	3.81
0	3	0	0	26	20.3	13.4	28.5	73	2.16
1	1	0	0	27.8	57.4	28.3	31.3	82.2	3.02
0	2	0	1	54.2	71.1	18.6	30.1	610.2	1.88
0	0	0	0	32.3	35.4	17	37.3	93.4	3.2
1	3	0	0	29.3	56.2	11.4	29.4	180.5	3.3
1	1	0	1	19.9	34.4	11.4	26.4	63.8	1.6
0	2	1	0	76.2	118.9	403.2	26.8	21	1.36
0	1	0	0	29	34.3	11.9	34.3	177.3	4.09
0	1	0	0	128.4	114.5	69.3	37.4	143.1	3.79
0	0	0	0	11.7	12.1	7	38.4	14.1	3.81
0	0	0	0	21.6	18	16.6	38	33.4	3.61
0	2	0	1	53.2	134.9	50.1	18.3	93.9	1.28
0	0	0	0	119.2	81.3	20.5	42.9	85	3.14
0	1	0	0	30.2	75.9	20.1	21.4	118	2.07
0	0	0	0	33.2	43.8	33.7	28.5	78.6	2.74
0	2	0	0	45.5	251.7	20	34.2	245.6	4.87
0	0	0	0	15.1	21.5	11.8	40.7	19.1	3.4
0	2	0	0	175	161.9	44.5	36	268.1	3.36
0	1	0	0	43.5	55.3	25.8	32.4	257.3	2.86
1	1	2	0	16.8	21.8	21.5	35.7	46.3	2.81
0	0	0	0	31.7	76.5	34.3	39.2	42.9	4.15
0	0	0	0	152.3	61.4	22.1	40.5	172.9	4.6
1	3	0	0	21.9	109.9	51.6	27	69.1	4.42
0	2	0	0	36.2	63	25.3	28	320.8	5.58
1	0	0	0	71.2	62.8	217.9	36.7	539.9	4.81
0	2	0	0	40.5	83.5	336.5	34.3	92.5	2.42
0	0	1	1	21.1	30.2	28.8	26.9	50.3	3.07
0	0	0	0	39	127.4	36.9	35.9	11	3.4
1	2	1	0	483.8	466.5	100.5	22.8	73.7	3.39
0	0	0	0	26.1	23.4	18	45.3	32.2	4.98
0	3	0	0	35.4	68.4	44.2	22.2	20.2	3.65
0	3	0	0	34.2	59.9	42.9	32.9	69	3.99
0	0	0	0	32.9	28.9	7.1	41.9	119.2	4.96
0	3	0	0	87.4	136.4	32.2	34.2	225.6	3.97
0	2	0	0	33.6	36.5	41.9	36.1	55	3.04
0	2	0	0	18	99.6	110.3	23.9	521	2.98
0	0	0	0	21.4	38.9	10	34.2	89.8	5.08
0	3	0	1	35.1	58	9.6	22.3	121	5

0	3	0	0	31.5	65.9	41.9	25.7	57	2.94
1	0	0	0	12	24.1	10.5	37.6	26.9	4.74
0	2	0	0	76.4	120.7	60.1	37	478.2	5.77
1	2	0	0	407.9	445.1	82.6	25.9	169.9	1.16
1	2	0	0	100.7	339.7	116.3	32.1	511.2	3.45
0	2	0	0	31.9	99	15.4	19.2	42.6	5.01
1	2	0	0	19.8	40.4	13.2	32.4	121.5	2.37
1	1	0	0	54.7	53.3	34	28.5	121.6	4.29
0	0	0	0	72	71.8	6.3	40.3	255	5.95
0	0	0	0	30.8	21.2	7.2	50.1	70.9	4.04
0	0	0	0	44.8	40.7	382.3	42.2	485.9	6.52
0	0	0	0	32.6	40.3	24.9	35	75.7	4.54
0	2	0	0	46.4	137.3	46.2	31	750	2.65
0	3	0	0	32.6	207.4	20.6	34.7	251.3	4.66
0	2	0	1	31.9	34.8	34	25.2	58.6	2.87
0	0	0	0	123.6	190.1	287.3	25.4	496.1	3
0	2	0	0	51.5	197.8	121.7	24.2	263.3	4.46
1	2	0	0	35.8	145.6	26.4	32.2	474.5	4.39
0	3	0	0	120.6	115.1	41.9	30	86.7	3.66
0	3	0	0	104.6	502.7	42	42.4	186.7	3.71
1	0	0	0	11.5	17.1	10.6	40.8	13.1	3.77
0	2	0	0	13.7	23.4	10.5	29.6	43.4	3.46
1	2	0	0	47	61.7	22.9	36.8	147.1	4.11
1	2	0	0	23.3	30.3	40.6	34.8	26.5	3.58
1	0	0	0	33.5	33.4	12.9	47.2	23.5	4.67
0	3	0	0	61.8	106.1	42.8	20.2	33.9	2.11
0	0	0	0	24.8	37.5	17.7	40.3	121	4.28
0	2	0	0	49.1	51.4	25.5	31.1	93.8	3.51
0	2	0	1	26.6	31.6	16.7	31.8	36.2	3.48
0	0	1	0	61	95.2	84.1	27.9	550.7	3.28
0	0	0	0	28.5	26.7	12.4	41.7	50.3	2.7
0	3	0	0	463.7	623.3	70.1	27.4	368.3	4.35
0	0	0	0	3.4	18.5	13.9	40.9	27.1	2.47
1	0	0	0	12.9	20.4	26.3	36.8	197.9	3.82
0	3	0	0	97.7	226.3	182.7	26.7	387.3	1.51
0	2	0	0	12.6	19.1	22.6	31.3	25.5	1.7
0	0	0	0	73.4	100.3	35.2	43.3	253	4.46
0	2	0	0	19.8	64.8	20.2	29.8	61.8	3.31
0	2	0	0	35.9	35.2	28.6	27.3	35.3	2.85
0	1	0	0	8.2	60.4	18	28	101.5	2.98
0	1	0	0	63.3	127.8	35.6	33.4	72.6	2.84
1	2	0	0	445.9	408.6	22.7	33.2	16.7	1.84
0	1	0	0	116.3	276.1	508	32.1	867.4	2.18
1	2	0	0	25.3	25	15.3	36.1	54.9	4.4

0	3	0	0	31.4	37.6	25.2	33.6	43.2	3.35
0	3	0	0	37.9	65.8	22.4	30.8	109.3	3.99
1	3	0	0	47.8	165.1	67.2	27.1	821.1	3.29
1	3	0	1	424.6	312.6	39.3	26.5	106.4	2.91
0	3	0	0	12.2	41	29	29.2	34.1	2.07
1	2	0	0	34	72	11.3	37.4	28.5	3.2
0	1	0	0	68.7	88.8	33	32	188.6	3.61
0	2	0	0	64.7	133.8	28.6	34.9	375.7	3.86
1	3	1	0	84	166.7	41.2	25.7	122.4	2.73
0	1	0	0	33.4	32.2	17.2	37.3	57.5	6.15
0	3	0	0	38.1	49	37.4	19.4	50.9	1.69
0	2	0	0	140.6	205.2	67	34.9	502	3.46
0	3	0	0	104	349.6	40.1	34.1	69.8	11.23
0	0	0	0	26.8	55.9	15.8	41.3	103.6	4.09
1	1	0	0	182.3	121.4	34.4	35	257.7	3.87
0	1	0	0	38.6	65.9	79.9	36.9	79.2	2.96
0	0	0	0	17.9	23.7	16.8	46.6	19.8	4.52
0	1	0	0	19.9	23.8	8.8	40.2	18.7	3.61
0	1	0	0	30.9	69.8	50.9	24.6	132.5	2.5
0	0	0	0	15.3	34.6	23.5	38.9	27.6	3.78
0	0	0	0	42.1	53.4	9.4	42.6	28.2	3.38
0	3	0	0	14	46.3	42.3	28.8	45	1.68
0	2	0	0	81.9	439.1	25.3	28.5	343	5.95
0	2	0	0	34.9	78.5	42.1	38.7	148.7	3.49
0	3	0	0	44.9	135.5	39.4	32.2	402.1	4.51
0	0	0	0	30.2	31.9	7.4	35.6	106.3	5
0	2	0	1	33	39.1	17.6	24.7	141.6	2.1
0	2	0	0	86.3	178.5	256.1	22.6	362.8	2.43
0	3	0	0	133	208.1	538	27.6	103.6	1.83
0	3	0	1	48	58.6	41.7	26.5	67.4	2.52
0	2	0	0	431	399.5	93.2	35	83.2	3.14
0	2	2	0	28.3	51.5	42.6	22.6	7	1.85
1	1	0	0	18.6	31.7	12.5	24.8	150.6	5.43
0	1	0	1	17.8	27.5	23.5	28.1	50.2	1.85
0	0	0	0	146.3	229.4	11.5	38.3	104	4.05
0	0	0	0	14.4	27	34.8	25.6	62.6	3.36
0	2	1	0	52.9	81.9	100.3	28.3	18.5	1.28
0	2	0	0	56.4	84.6	129.1	36.5	165.5	2.83
0	1	0	0	94.6	186.3	76.3	30.7	319.1	5.01
0	1	0	0	15.2	19.8	10.9	39.8	136.5	4.44
0	0	0	0	292.6	180.6	26.8	40.3	191.4	3.28
0	3	0	0	161.8	418.2	193	26.3	21.6	4.19
1	0	0	0	22	15	11.5	34.7	55.6	4.05
0	2	0	0	16.1	51	134.2	24.7	14	1.92

0	1	0	0	23.3	59.6	11.2	42.2	20.9	4.35
0	1	0	0	50.6	89.6	67.6	21	35.7	2.23
1	0	0	0	92.2	139.1	15.9	42	811.3	5.81
0	2	0	0	120.8	216.2	114.2	25.3	135.4	6.81
0	0	1	0	13.1	20.5	46.5	30.1	13.9	2.01
0	0	0	0	65	24.9	7.6	42.1	73.4	5.97
0	2	0	0	430.2	579.9	30.2	24	118.3	3.31
0	1	0	1	63	47.7	25.2	29.1	59	3.38
0	1	0	1	280	338.6	170	33	473.3	2.91
0	1	0	0	72.4	77.7	122.2	40.9	79.7	3.89
0	0	0	0	43.2	50.7	18.4	34.9	25.5	3.95
1	2	0	0	141.7	75.4	46.5	30.8	239.8	3.71
0	0	0	0	11.4	25.3	12.4	33.3	17.7	3.13
0	2	0	0	96.5	352.4	91.6	30.1	23.4	4.02
0	1	0	0	72.4	133.8	44.5	21.9	145	3.95
0	0	0	0	17.6	14.5	9.5	44.7	39.1	3.6
0	0	0	0	79.3	43.8	12.8	37.7	70.3	3.02
0	2	0	0	119.1	405	158	43.6	21.1	2.17
0	3	0	0	17.2	91.5	20.2	7.7	83.5	1.45
1	1	0	0	31.2	93.1	32.4	34	22.4	4.73
0	2	0	0	13.5	26	18.3	30.2	21.4	4.49
0	0	0	0	45.3	91.8	23.9	32	183.4	3.06
1	0	0	1	25.4	21.2	40	31.4	78.7	2.6
0	1	0	0	31.8	40.7	17.5	41.4	107.6	3.88
1	0	0	0	21.3	25.9	24.4	38.6	24.5	3.34
0	2	2	0	9.9	25.5	63.7	29	14.4	1.34
1	0	0	0	13.1	26.9	7.6	40.6	50	7.74
0	1	0	0	20.6	56.3	35.3	31.9	111	4.44
1	0	0	0	236.5	281.5	98.7	31.7	1562.6	7.3
0	1	0	0	222.9	321.2	290.5	26.6	339.3	2.72
0	1	0	0	23.4	22.5	19.8	40.7	63.8	3.23
1	0	0	0	18.4	19.9	12.4	41.2	59	4.51
0	3	0	0	28.5	244.7	24.5	28.7	152.3	5.28
1	3	1	0	50.1	90.4	459.8	24.9	26.9	1.64
1	1	0	0	21.7	33.9	7.1	32.1	224	4.06
1	2	0	0	30.2	25.8	7.4	40.5	27.4	3.57
0	1	0	0	14	35.7	25.4	28.6	33.6	1.58
0	0	0	0	19.2	21.2	11	42.1	29	3.97
0	3	0	0	67.5	97.1	90.4	31.5	73	5.21
0	0	0	0	40.9	24.5	12.9	44.1	75.4	5
0	3	0	0	68	89.5	53.5	31.6	256.7	3.98
1	0	0	0	107.1	84.1	11.3	34.4	109.8	3.76
0	2	0	1	45.4	23.6	34.9	28.6	35.6	3.73
0	0	0	0	23.4	25.9	17.9	38.3	27.6	2.68

0	2	0	0	21.3	66.8	11.4	27.6	60.3	3.33
1	1	0	0	30.2	44.4	19.9	40.7	36.7	4.16
0	0	0	0	21.1	25.7	10.8	42.8	19.8	4.47
0	1	0	0	66.7	61.7	14.5	27.2	60.5	2.13
1	0	0	0	11.2	22	7.5	40.2	12.1	4.56
0	1	0	0	16.6	36.1	13.1	46.2	222	4.78
0	2	0	0	20.6	76.1	108.9	30	139.5	2.88
1	2	0	0	19.2	33.6	4.4	31	22.3	2.91
0	2	0	0	95.7	99.3	84.6	37.1	2514.2	4.8
0	0	0	0	28.1	20.4	20.8	42.4	51.4	4.36
0	3	0	0	20	31.7	28.3	26.5	124.9	2.8
0	1	0	0	44.5	45.9	29.4	31.7	44.7	2.86
0	1	0	0	21.8	41.3	81.3	24.9	23.8	2.33
1	2	0	0	3	11.2	4.4	31.3	68.2	3.1
0	3	0	0	44.6	121.1	148	26.5	67.9	3.26
1	1	0	1	69.7	53.2	23	25.7	10.6	1.99
1	1	0	0	28.6	33.3	6.5	29.3	77.9	2.87
0	0	0	0	30.6	75.3	24.7	41.2	226.5	4.82
1	1	0	0	59.6	64.2	26.9	41.8	235.3	8.68
0	2	0	0	81.6	221.5	59.5	30.8	507.9	8.12
0	0	0	0	61.4	52.2	335.4	39.8	54.9	2.57
0	1	0	0	82.7	227.1	37.2	30.3	12.3	3.78
0	1	0	0	58.3	121	219.3	26.9	156.1	2.82
0	3	0	0	56.3	200.2	250	30.3	307.5	4.32
1	0	0	0	21.8	18.9	15.3	47.5	16.3	3.31
0	2	0	0	52.4	71.9	20.4	34.3	23.7	3.03
0	2	0	0	6.6	18.4	17.8	31.7	146.3	2.89
1	1	0	0	26.7	51.7	48.4	29.5	130.9	2.25
0	0	0	0	74.1	62.4	20.2	37.5	86	3.66
1	2	0	0	24.7	63.7	40	21.7	21.4	3.08
1	2	0	0	85	248.5	64.8	37	23.4	4.04
0	3	0	0	16.5	62.1	19.5	35.1	34	3.16
0	1	0	0	17.8	42.5	25.6	32.5	18.9	4.06
0	2	0	0	85.8	133.3	49.2	27.2	350.4	4.19
0	0	0	0	16.8	24.7	17.2	46.3	18.3	4.66
0	3	0	0	14.7	27.6	24.6	33.8	57.3	3.81
0	3	0	0	59.3	133.5	60.5	31.7	101.8	1.94
1	1	0	0	43.4	131.8	8.5	29.4	56.8	3.31
0	2	0	0	14.3	25.8	58.1	30	34	2.31
0	1	1	0	31.6	47.3	53.1	35.9	23.1	2.61
1	0	0	0	19	50.4	29.6	36.1	516.5	8.13
1	0	0	0	5.4	16.1	10	43.3	17.5	4.84
0	1	0	0	34.9	75.6	50.2	34.3	298.1	3.42
1	1	0	0	18.7	24.1	10.6	30.9	70.3	3.23

1	3	0	0	20	77	15.8	28.3	381.3	2.52
1	2	0	0	17.4	41.3	29.8	34.2	125.2	2.8
1	3	0	0	12.2	28.3	13.1	28.2	29.2	2.82
0	0	0	0	17.4	63.7	53.4	29.7	245.2	3.33
1	0	0	0	25.1	36.7	22.5	31.3	204.3	4.11
1	0	0	0	8.6	18.5	4.5	38.4	24.6	3.71
0	0	0	0	10.6	20.7	42.2	27.4	20.2	2.26
0	2	0	0	25.4	48.3	30	36.6	24.5	3.87
0	2	0	0	63.8	103.4	11.6	36.4	119.8	3.11
1	2	0	0	35.9	58	28.5	33.8	379.9	5
0	0	0	0	42.5	36	15.4	34.9	75.6	4.13
1	0	0	0	52.3	49.1	7.1	34	80	2.97
0	2	0	0	139.5	104.1	59.2	26.8	80.2	3.84
1	0	0	0	29.7	18.6	80.4	43.1	10.7	4.06
1	0	0	0	114	164.9	19.8	32.9	128.6	3.57
0	1	0	0	63.4	244.8	40.2	32	214.9	3.19
1	2	0	0	11.9	37.6	27	41.3	397.1	2.7
1	0	0	0	19.1	24.2	9.3	31.2	33.8	6.43
0	2	0	0	24.8	43.7	32.3	35.3	77.6	3.13
0	1	0	0	19.7	31.4	25.8	31.2	58.6	2.98
0	2	0	0	23.6	42.6	24.6	26.5	31.3	2.55
0	3	0	0	82.2	449.7	83.4	39.8	200.8	5.63
1	1	0	0	16.6	24.1	39.4	30	70.8	3.54
1	2	0	0	47.1	45.7	12.5	35.7	224.2	3.57
0	0	0	0	45.7	31.2	14.9	43.1	15.9	4.64
1	2	0	0	36.3	92.9	51.4	28.7	335.4	3.85
1	1	0	0	129.2	174.6	14.1	26.9	45.6	3.54
1	1	1	0	47.3	97.9	130.6	31.1	209.3	2.1
0	2	0	1	10.1	26	6.4	31.8	64.9	3.52
0	1	0	0	26.6	49.5	63.6	24.8	20.5	1.63
0	3	1	0	97.4	412.7	155.3	29.5	569.6	2.94
0	2	0	0	40.9	97.4	49.3	35.4	209.3	3.42
1	1	0	0	15.5	24.3	9.4	26.9	16	2.9
0	3	0	0	93	191.3	57.6	22.1	58.3	2.37
0	0	0	0	14.2	40.3	14.5	35.8	383.6	2.8
0	2	0	0	33.6	91.2	24	34	316.3	5.13
0	1	1	0	32.4	42.3	58.3	25.8	62.5	2.21
0	1	0	0	16	33.2	13.6	30.8	20.9	2.55
0	2	0	0	175.4	210.6	66.4	25.1	114.4	3.15
0	1	0	0	22	60.4	43.1	34.7	397.4	3.96
1	2	0	0	68.7	93.7	153.9	31	438.3	2.84
1	1	0	0	123.6	197.5	16.2	34.1	123.2	2.87
0	1	0	0	13.5	23.2	27.1	31.3	83.9	3.36
1	1	0	0	49	122.2	40.5	33	494.1	2.74

1	1	0	0	19.8	52.4	157.9	28.4	112.9	2.48
1	3	0	0	81.1	89.4	18.7	32	329.7	6.95
1	2	3	1	35.5	59.5	143.9	22.7	33.4	3.21
1	1	0	0	70	112.2	43.7	35.2	208.2	3.77
0	1	0	0	65	50.8	416.3	32.7	30.5	4.29
0	1	0	0	106.1	553	32.5	28.1	110.2	3.85
1	1	0	0	25.5	37.4	25	44.2	70.6	3.09
1	2	0	0	77.9	117.8	70.6	28.8	33.6	2.58
0	2	0	0	18	197.9	89.7	28.8	114	1.56
0	3	0	0	22	48.5	25.7	34.1	127.7	3.9
1	2	1	0	90.5	132.7	35.6	26.7	138.7	2.87
0	3	0	0	62.1	184.6	154.8	24.8	207	2.95

TG	HDL	LDL	NC	LC	MC	NHR	LHR	MHR	BUN
0.51	0.68	1.54	3.41	0.74	0.28	5.01	1.09	0.41	6.02
1.32	0.92	4.59	3.98	1.8	0.64	4.33	1.96	0.70	7.26
1.05	0.64	1.86	2.91	0.98	0.43	4.55	1.53	0.67	12.68
0.98	0.96	2.93	1.32	1.56	0.17	1.38	1.63	0.18	5.5
0.6	1.08	1.68	1.28	0.75	0.22	1.19	0.69	0.20	4.53
0.67	0.86	2.75	2.62	1.43	0.55	3.05	1.66	0.64	4.25
0.7	0.77	1.21	1.45	0.66	0.13	1.88	0.86	0.17	6.2
1.66	1.12	2.26	3.32	1.16	0.34	2.96	1.04	0.30	5.75
0.66	1.76	1.05	2.06	0.95	0.37	1.17	0.54	0.21	5.39
0.62	0.63	1.63	1.79	1.03	0.46	2.84	1.63	0.73	4.41
0.86	1.16	3.23	3.4	1.47	0.33	2.93	1.27	0.28	3.94
0.8	1.46	2.93	2.38	1.43	0.28	1.63	0.98	0.19	5.15
1.01	0.77	2.02	1.64	0.76	0.28	2.13	0.99	0.36	5.48
0.73	0.9	1.79	1.43	1.33	0.29	1.59	1.48	0.32	4.25
1.23	0.9	2.65	1.05	0.91	0.21	1.17	1.01	0.23	4.5
1.1	0.78	1.44	1.91	0.99	0.36	2.45	1.27	0.46	4.03
0.72	1.29	2.87	3.53	1.55	0.47	2.74	1.20	0.36	6.71
0.38	1.09	1.72	1	0.28	0.13	0.92	0.26	0.12	7.73
1.05	1.16	2.63	2.34	2.45	0.42	2.02	2.11	0.36	4.5
0.71	1.51	2.45	3.27	0.92	0.4	2.17	0.61	0.26	3.59
0.46	0.49	1.35	4.25	0.66	0.47	8.67	1.35	0.96	24.69
0.28	1.88	1.9	1.89	1.68	0.24	1.01	0.89	0.13	6.14
1.78	0.74	2.91	2.79	0.59	0.34	3.77	0.80	0.46	6.5
0.8	1.49	2.48	0.85	0.83	0.25	0.57	0.56	0.17	5.29
0.63	1.14	1.51	0.94	0.54	0.18	0.82	0.47	0.16	3.89
2.19	1.6	3.18	1.8	0.6	0.17	1.13	0.38	0.11	5.61
2.05	0.2	1.16	3	0.8	0.23	15	4.00	1.15	7.08
0.86	1.55	2.82	7	2.6	0.35	4.52	1.68	0.23	5.34
0.95	0.72	1.66	1.85	0.56	0.23	2.57	0.78	0.32	4.63
0.69	0.88	2.16	3.66	0.92	0.67	4.16	1.05	0.76	6.41
0.66	1.52	2.15	2.76	0.7	0.28	1.82	0.46	0.18	7.08
1.09	1.51	1.64	1.69	0.43	0.22	1.12	0.28	0.15	4.48
0.66	1.11	1.97	1.48	0.49	0.51	1.33	0.44	0.46	3.74
0.81	0.78	1.7	3.4	1.88	0.4	4.36	2.41	0.51	8.21
0.44	1.16	2.04	3.65	0.67	0.47	3.15	0.58	0.41	7.38
1.16	1	3.65	2.02	1.59	0.39	2.02	1.59	0.39	4.2
1.29	1.53	6.22	3.18	2.05	0.46	2.08	1.34	0.30	10.3
0.79	1.45	1.66	1.2	1.13	0.17	0.83	0.78	0.12	7.62
0.4	0.91	1.62	0.99	0.29	0.23	1.09	0.32	0.25	3.22
0.9	1.45	2.94	2.44	2.49	0.59	1.68	1.72	0.41	7.28
0.95	1.1	2.78	3.77	1.99	0.5	3.43	1.81	0.45	5.41
1.32	0.8	2.25	2.52	1.27	0.23	3.15	1.59	0.29	7.94
1.84	1.25	3.8	3.71	1.97	0.5	2.97	1.58	0.40	4.2

1.09	1.2	2.96	3.77	2.25	0.48	3.14	1.88	0.40	5.29
0.63	0.74	1.67	1.02	1.31	0.19	1.38	1.77	0.26	7.13
0.86	1.21	3.46	1.32	1.99	0.26	1.09	1.64	0.21	3.85
1.27	1.06	3.29	3.34	1.11	0.72	3.15	1.05	0.68	8.36
0.89	2.68	3.91	1.45	1.21	0.24	0.54	0.45	0.09	3.76
0.56	1.24	1.61	1.44	0.82	0.17	1.16	0.66	0.14	4.36
0.82	0.89	2.24	2.72	1.44	0.4	3.06	1.62	0.45	5.14
0.43	1.17	1.33	1.68	0.6	0.15	1.44	0.51	0.13	4.06
0.6	1.21	2.05	2.07	1.99	0.44	1.71	1.64	0.36	4.61
0.8	1.19	2.44	1.26	1.35	0.36	1.06	1.13	0.30	9.01
0.98	0.86	1.53	2.7	1.5	0.54	3.14	1.74	0.63	5.94
0.78	1.16	3.03	2.81	1.65	0.35	2.42	1.42	0.30	4.83
0.73	0.72	2.58	5.36	1.75	0.71	7.44	2.43	0.99	4.74
1.06	1	2.46	4.77	1.52	0.43	4.77	1.52	0.43	4.16
0.63	1.06	1.33	2.35	0.45	0.33	2.22	0.42	0.31	13.46
0.52	1.48	1.48	2.43	0.75	0.33	1.64	0.51	0.22	6.49
0.76	1.34	1.96	2.1	0.7	0.2	1.57	0.52	0.15	6.24
0.7	2.24	2.24	2	0.7	0.51	0.89	0.31	0.23	8.39
1.1	0.99	1.09	1.85	0.48	0.09	1.87	0.48	0.09	5.1
0.74	1.38	2.4	4.48	1.32	0.57	3.25	0.96	0.41	4.01
0.4	1.38	1.88	1.47	1.61	0.5	1.07	1.17	0.36	4.98
1.13	1.18	1.72	1.82	1.05	0.28	1.54	0.89	0.24	4.33
1.45	1.28	2.75	3	1.61	0.35	2.34	1.26	0.27	8.03
0.87	0.86	1.64	1.3	1.05	0.18	1.51	1.22	0.21	4.97
1.08	1.41	4.08	1.84	1.45	0.4	1.3	1.03	0.28	5.03
1.02	1.85	1.84	1.3	0.69	0.23	0.7	0.37	0.12	3.98
1.12	1.55	2.16	3.18	1.85	0.47	2.05	1.19	0.30	5.69
0.75	0.66	1.67	1.24	1.01	0.44	1.88	1.53	0.67	4.41
0.6	1.06	1.84	1.33	1.09	0.16	1.25	1.03	0.15	7.31
0.55	0.88	2.09	5.4	1.91	0.57	6.14	2.17	0.65	7.59
0.47	0.74	0.59	6.05	0.1	0.2	8.18	0.14	0.27	10.26
0.72	1.08	1.65	2.01	0.68	0.17	1.86	0.63	0.16	5.28
0.58	1.09	2.49	2.59	2.05	1.49	2.38	1.88	1.37	4.2
0.76	1.69	1.58	2	1.3	0.12	1.18	0.77	0.07	3.27
0.78	1.18	1.23	3.43	1.7	0.71	2.91	1.44	0.60	8.59
0.53	1.74	2.98	2.87	1.04	0.42	1.65	0.60	0.24	4.95
1.07	1.36	2.86	2.6	1.6	0.47	1.91	1.18	0.35	4.43
0.63	1.65	1.46	1.93	1.18	0.32	1.17	0.72	0.19	4.85
2.07	0.56	1.9	2.24	1.98	0.38	4	3.54	0.68	3.39
0.98	1.06	2.62	1.76	0.88	0.21	1.66	0.83	0.20	5.25
0.33	1.34	1.22	2.01	1	0.58	1.5	0.75	0.43	4.66
0.26	1.37	0.82	17.6	1.5	0.34	12.85	1.09	0.25	13.29
1.02	0.99	1.55	2.71	1.92	0.44	2.74	1.94	0.44	4.04
0.77	0.78	0.83	2.94	0.7	0.2	3.77	0.90	0.26	4.42

0.56	0.31	1.83	1.8	1.57	0.47	5.81	5.06	1.52	7.29
1.2	0.77	1.03	4.73	2.06	0.34	6.14	2.68	0.44	4.51
0.71	2.15	1.41	1.3	0.9	0.22	0.6	0.42	0.10	3.93
0.73	1.78	1.99	1.43	0.54	0.22	0.8	0.30	0.12	4.33
1.2	1.09	2.73	4.25	2.51	0.48	3.9	2.30	0.44	7.01
0.66	1.32	1.34	1.09	0.67	0.12	0.83	0.51	0.09	6.03
0.65	1.15	2.48	2.19	2.06	0.42	1.9	1.79	0.37	4.17
0.53	1.24	1.75	2.35	1.82	0.45	1.9	1.47	0.36	7.43
1.29	1.43	2.31	2	1.28	0.26	1.4	0.90	0.18	2.93
0.8	0.68	1.88	2.98	2	0.44	4.38	2.94	0.65	7.43
0.48	1.33	1.18	7.3	1.2	0.32	5.49	0.90	0.24	4.26
0.81	1.05	2.81	2.9	1.42	0.4	2.76	1.35	0.38	5.57
0.88	1.8	1.03	1.82	2.33	0.68	1.01	1.29	0.38	7.08
1.11	1.67	1.7	1.2	1.4	0.35	0.72	0.84	0.21	6.08
0.57	2.12	2.47	1.6	1.6	0.37	0.75	0.75	0.17	4.68
0.7	1.64	2.6	1.6	1.7	0.45	0.98	1.04	0.27	4.92
0.75	1.11	2.09	3.8	1.44	0.28	3.42	1.30	0.25	6.36
0.73	0.76	0.88	1.3	0.8	0.71	1.71	1.05	0.93	8.5
0.76	0.98	2.67	2.64	1.4	0.29	2.69	1.43	0.30	6.04
0.47	1.61	1.95	0.95	0.36	0.18	0.59	0.22	0.11	4.11
0.87	1.56	2.19	2.54	0.94	0.22	1.63	0.60	0.14	4.26
0.62	0.83	1.64	1.9	1.45	0.22	2.29	1.75	0.27	3.3
0.52	1.3	2.13	2.06	0.66	0.16	1.58	0.51	0.12	5.79
0.8	1.51	1.99	2.1	1.5	0.35	1.39	0.99	0.23	5.92
1.56	1.76	8.48	4	1.4	0.07	2.27	0.80	0.04	4.14
0.9	0.85	1.96	1.41	0.87	0.22	1.66	1.02	0.26	5.69
0.96	1.04	1.58	5.36	1.67	0.42	5.15	1.61	0.40	4.41
1.45	1.79	1.96	1.63	1.38	0.26	0.91	0.77	0.15	5.41
0.89	1.27	6.24	4.59	1.47	0.56	3.61	1.16	0.44	5.88
0.61	1.4	2.42	2.16	2.05	0.34	1.54	1.46	0.24	4.57
0.44	1.32	1.06	5.98	1.46	0.34	4.53	1.11	0.26	11.54
0.67	0.93	1.77	3.23	2.08	0.35	3.47	2.24	0.38	5.06
0.67	1.74	3.03	2.33	0.86	0.35	1.34	0.49	0.20	6.62
0.8	1.07	3.22	4.3	1.5	0.28	4.02	1.40	0.26	5.36
1.84	0.74	1.59	2.93	1.29	0.31	3.96	1.74	0.42	4.71
0.58	1.38	2.72	3.51	1.59	0.45	2.54	1.15	0.33	5.7
0.95	1.41	2.03	2.47	2.18	0.29	1.75	1.55	0.21	6.04
0.51	1.6	1.99	1.42	0.69	0.24	0.89	0.43	0.15	8.23
0.5	2.28	0.91	1.67	1.37	0.2	0.73	0.60	0.09	3.88
1.07	0.89	3.49	3.71	1.03	0.41	4.17	1.16	0.46	5.01
0.82	1.54	3.29	3.28	1.72	0.28	2.13	1.12	0.18	5.95
0.73	1.39	2.5	2.91	2.13	0.61	2.09	1.53	0.44	4.56
1.14	1.31	2.02	2.37	1.29	0.35	1.81	0.98	0.27	5.05
0.83	0.98	2.17	3.18	1.57	0.22	3.24	1.60	0.22	4.41

0.63	1.14	1.96	1.86	0.97	0.3	1.63	0.85	0.26	3.86
0.92	1.87	3.38	2.01	1.16	0.21	1.07	0.62	0.11	2.34
1.04	1.54	3.02	1.63	1.1	0.3	1.06	0.71	0.19	7.96
1.1	1.66	2.81	3.34	2.11	0.5	2.01	1.27	0.30	3.49
0.47	1.08	1.98	3.42	0.91	0.45	3.17	0.84	0.42	8.51
1.44	1.41	2.43	1.87	1.65	0.35	1.33	1.17	0.25	4.7
0.9	1.27	2.95	3.96	0.9	0.5	3.12	0.71	0.39	3.13
2.06	1.37	2.01	4	1.39	0.42	2.92	1.01	0.31	5.53
0.62	1.33	2.17	1.58	0.92	0.16	1.19	0.69	0.12	4.15
1.24	1.19	2.03	1.8	2.01	0.4	1.51	1.69	0.34	5.59
0.88	1.53	4.01	3.3	1.4	0.23	2.16	0.92	0.15	8.18
0.64	1.17	2.73	1.3	1.15	0.24	1.11	0.98	0.21	5.19
0.62	0.84	1.27	1.61	1.57	0.68	1.92	1.87	0.81	2.98
0.51	1.47	2.13	3.01	0.63	0.37	2.05	0.43	0.25	8.93
0.56	0.77	1.15	1.54	0.65	0.27	2	0.84	0.35	7.47
0.33	1.62	1.89	1.25	1.76	0.6	0.77	1.09	0.37	4.83
0.43	1.34	1.46	1.38	0.62	0.18	1.03	0.46	0.13	6.22
1.9	1.14	4.71	2.81	1.32	0.39	2.46	1.16	0.34	6.06
0.92	1.32	2.12	1.21	0.76	0.34	0.92	0.58	0.26	5.46
1.42	1.23	2.78	1.72	0.97	0.22	1.4	0.79	0.18	3.52
0.64	1.26	1.37	1.68	0.79	0.2	1.33	0.63	0.16	4.57
0.69	1.31	2.24	1.46	0.51	0.26	1.11	0.39	0.20	4.26
0.61	1.02	2.09	2.11	1.04	0.34	2.07	1.02	0.33	6.27
0.46	1.26	1.27	1.11	0.6	0.28	0.88	0.48	0.22	4.01
1.12	1.54	2.16	1.45	1.37	0.43	0.94	0.89	0.28	4.08
0.56	0.97	1.45	1.35	1.01	0.21	1.39	1.04	0.22	5.49
0.7	1.11	2.55	2.39	1.09	0.32	2.15	0.98	0.29	4.98
1.29	0.62	1.68	1.94	0.8	0.3	3.13	1.29	0.48	3.38
1.4	1.41	2.06	1.87	1.39	0.47	1.33	0.99	0.33	3.84
1.13	0.96	3.27	2.87	1.5	0.4	2.99	1.56	0.42	4.85
1.04	1.59	1.96	2.21	0.76	0.25	1.39	0.48	0.16	5.13
0.51	0.25	1.95	2.27	0.84	0.28	9.08	3.36	1.12	9.27
1.2	1.09	2.15	2.1	1.6	0.43	1.93	1.47	0.39	9.42
0.87	0.66	1.83	2.75	1.6	0.19	4.17	2.42	0.29	7.81
1.15	0.97	2.89	3.69	1.71	0.63	3.8	1.76	0.65	6.3
1.38	1.04	2.86	5.47	1.67	0.4	5.26	1.61	0.38	4.13
1.17	0.98	2.13	2.5	1.17	0.22	2.55	1.19	0.22	4.81
0.75	0.83	2.11	6.46	2.6	1.05	7.78	3.13	1.27	5.78
0.65	1.18	1.72	2.46	1.01	0.29	2.08	0.86	0.25	7.78
0.89	0.66	2.2	3.33	1.7	0.68	5.05	2.58	1.03	5.8
1.14	1.25	2.37	3.7	1.1	0.28	2.96	0.88	0.22	5.68
1.92	0.59	2.83	3.32	1.2	0.4	5.63	2.03	0.68	6.14
1.28	0.79	2.17	2.78	1.07	0.2	3.52	1.35	0.25	4.3
0.47	1.07	1.58	2.79	0.82	0.06	2.61	0.77	0.06	5.01

0.56	0.68	2.16	1.13	1.01	0.18	1.66	1.49	0.26	6.67
0.7	1.14	1.87	2.88	1.44	0.37	2.53	1.26	0.32	6.15
1.93	1.04	2.7	2.37	2.29	0.46	2.28	2.20	0.44	5.2
0.54	1.25	1.3	1	0.2	0.2	0.8	0.16	0.16	5.13
1.3	1.03	1.74	2.19	1.47	0.41	2.13	1.43	0.40	3.27
0.52	1.25	1.13	1.93	0.49	0.19	1.54	0.39	0.15	8.94
1.43	1.61	2.07	2.39	1.59	0.42	1.48	0.99	0.26	4.71
1.19	0.82	2.61	2.4	2.22	0.38	2.93	2.71	0.46	5.67
0.54	0.75	1.45	1.61	0.8	0.58	2.15	1.07	0.77	3.77
0.81	1.31	1.87	5.25	0.69	0.33	4.01	0.53	0.25	5.9
0.83	0.69	2.53	5.25	0.69	0.33	7.61	1.00	0.48	5.01
0.55	0.66	2.88	5.25	0.69	0.33	7.95	1.05	0.50	4.07
1.26	0.82	2.41	5.25	0.69	0.33	6.4	0.84	0.40	4.75
1.17	0.75	2.48	5.25	0.69	0.33	7	0.92	0.44	4.58
0.79	0.8	3.41	5.25	0.69	0.33	6.56	0.86	0.41	4.59
0.41	0.29	0.81	5.25	0.69	0.33	18.1	2.38	1.14	5.84
2.1	0.78	2.28	1.03	1.34	0.31	1.32	1.72	0.40	4.65
0.27	0.89	1.4	1.15	1.51	0.3	1.29	1.70	0.34	4.77
1.46	1.15	3.06	2.5	1.45	0.3	2.17	1.26	0.26	6.37
0.92	0.6	0.73	0.74	0.51	0.12	1.23	0.85	0.20	5.46
1.7	0.83	1.35	2.37	1.15	0.22	2.86	1.39	0.27	6.03
0.95	0.63	7.72	4.7	1.35	0.46	7.46	2.14	0.73	1.3
1.98	0.78	2.88	1.86	1.36	0.3	2.38	1.74	0.38	6.13
0.86	1.2	1.78	2.1	0.61	0.33	1.75	0.51	0.28	8.57
1.39	1.01	1.98	1.03	1.13	0.31	1.02	1.12	0.31	6.37
1.01	0.72	2.3	5.48	1.84	0.51	7.61	2.56	0.71	5.8
0.86	1.39	2.19	2.53	0.47	0.29	1.82	0.34	0.21	4.78
1.03	0.93	2.24	2.61	2.21	0.39	2.81	2.38	0.42	4.74
0.83	0.83	1.62	2.25	0.64	0.25	2.71	0.77	0.30	5.7
0.96	1.17	1.47	2.2	0.8	0.4	1.88	0.68	0.34	5.72
1.2	1.46	3.01	3.55	1.69	0.38	2.43	1.16	0.26	6.74
0.76	1.05	2.48	2.13	1.93	0.4	2.03	1.84	0.38	5.34
1.54	0.98	3	3.76	1.46	0.51	3.84	1.49	0.52	5.48
1.04	1.21	3.1	2	1.9	0.7	1.65	1.57	0.58	4.75
1.16	0.84	2.06	4.13	0.75	0.58	4.92	0.89	0.69	3.06
1.11	1.13	1.99	0.62	0.46	0.04	0.55	0.41	0.04	4.42
0.29	1.26	2.1	0.84	1.27	0.24	0.67	1.01	0.19	5
0.33	0.6	0.77	1.11	0.6	0.18	1.85	1.00	0.30	5.7
0.97	0.83	2.76	2.76	4.39	0.63	3.33	5.29	0.76	5.11
1.05	1.29	1.53	4.65	0.96	0.56	3.6	0.74	0.43	4.44
0.96	2.04	3.46	1.99	1.62	0.22	0.98	0.79	0.11	5.63
1.1	0.25	1.32	6.54	2.22	1.68	26.16	8.88	6.72	3.32
0.52	0.97	1.1	1	0.43	0.17	1.03	0.44	0.18	5.32
0.88	1.45	3.42	5.02	1.19	0.37	3.46	0.82	0.26	5.48

0.75	0.59	2.81	4.09	2.04	0.51	6.93	3.46	0.86	4.41
0.55	1.91	2.61	1.74	0.74	0.29	0.91	0.39	0.15	3.68
0.93	1.19	1.72	1.99	1.04	0.25	1.67	0.87	0.21	5.59
1.25	1.1	2.74	2.02	1.88	0.35	1.84	1.71	0.32	3.5
1.67	0.98	2.97	3.24	2.11	0.4	3.31	2.15	0.41	4.77
0.52	1.09	1.28	1.24	0.65	0.15	1.14	0.60	0.14	5.05
0.64	1.88	2.62	4.93	0.66	0.88	2.62	0.35	0.47	5.05
0.77	1.24	2.19	1.92	1.27	0.36	1.55	1.02	0.29	4.93
0.5	1.19	2.38	1.87	1.27	0.39	1.57	1.07	0.33	3.52
1.35	0.82	2.05	3.49	1.75	0.32	4.26	2.13	0.39	4.95
4.04	0.56	1.87	1.21	1	0.23	2.16	1.79	0.41	7.52
0.42	1.03	2.69	1.51	1.46	0.48	1.47	1.42	0.47	6.41
0.43	1.05	1.76	1.74	1.47	0.44	1.66	1.40	0.42	7.61
1.34	1.19	2.75	2.63	2.03	0.35	2.21	1.71	0.29	7.53
1.35	0.77	1.01	2.83	1.07	0.4	3.68	1.39	0.52	5.51
0.97	0.35	0.87	1.9	0.58	0.29	5.43	1.66	0.83	4
0.47	1.32	1.75	2.08	1.13	0.39	1.58	0.86	0.30	3.67
0.73	1.58	1.69	2.56	1.06	0.68	1.62	0.67	0.43	4.14
0.41	1.47	1.68	1.48	1.07	0.3	1.01	0.73	0.20	4.86
1.81	1.26	2.3	0.81	1.04	0.18	0.64	0.83	0.14	4.7
2.01	0.55	2.88	3.95	1.39	0.49	7.18	2.53	0.89	4.52
0.74	1.3	1.99	1.48	1.56	0.37	1.14	1.20	0.28	2.92
0.73	0.83	2.02	4.17	2.36	0.7	5.02	2.84	0.84	6.2
0.46	1.04	1.64	2.28	0.94	0.3	2.19	0.90	0.29	5.47
1.81	0.62	2.37	3.32	1.01	0.31	5.35	1.63	0.50	5.08
0.57	1.2	1.72	3.15	0.64	0.22	2.63	0.53	0.18	5.51
1.05	1.02	2.81	3.77	2.36	0.75	3.7	2.31	0.74	4.47
1.27	0.52	2.89	5.39	1.64	0.89	10.37	3.15	1.71	3.82
0.89	1.34	2.29	1.78	1.61	0.33	1.33	1.20	0.25	5.23
0.54	0.86	1.66	2.02	0.93	0.15	2.35	1.08	0.17	1.67
2.22	0.95	2.62	2.53	1.31	0.27	2.66	1.38	0.28	7.83
0.68	0.99	2.02	2.53	1.15	0.37	2.56	1.16	0.37	4.17
0.45	0.7	1.23	3.02	1.17	0.48	4.31	1.67	0.69	8.29
1.07	0.93	2.97	5.9	2.22	0.63	6.34	2.39	0.68	5.99
0.51	1.18	0.79	1.6	1	0.4	1.36	0.85	0.34	3.29
1.05	1.23	2.93	1.55	0.61	0.17	1.26	0.50	0.14	6.22
2.35	0.57	3.18	1.45	0.78	0.19	2.54	1.37	0.33	12.64
1.16	1.45	1.14	2.64	0.85	0.52	1.82	0.59	0.36	5.23
0.76	1.07	1.1	1.28	1.81	0.28	1.2	1.69	0.26	5.69
0.83	0.89	2.23	5.35	0.28	0.13	6.01	0.31	0.15	7.78
0.68	1.22	1.97	0.91	0.55	0.13	0.75	0.45	0.11	4.43
0.75	1.58	1.72	1.5	0.64	0.21	0.95	0.41	0.13	4.18
0.47	1.72	3.42	3.55	1.63	1.28	2.06	0.95	0.74	3.22
0.94	0.65	0.8	2.36	3.31	0.83	3.63	5.09	1.28	9.45

1.05	1.69	1.84	2.33	1.13	0.35	1.38	0.67	0.21	4.27
0.81	0.92	2.6	2.26	1.4	0.27	2.46	1.52	0.29	5.23
0.53	0.98	1.75	3.36	1.5	0.39	3.43	1.53	0.40	4.03
0.44	1.33	1.89	1.68	1.15	0.42	1.26	0.86	0.32	4.82
1.36	0.77	1.94	1.98	0.73	0.25	2.57	0.95	0.32	4.97
1.44	0.87	2	1.73	1.2	0.18	1.99	1.38	0.21	4.95
0.59	0.42	1.23	4.22	0.86	0.37	10.05	2.05	0.88	15.29
0.88	1	3.83	7.17	1.64	0.4	7.17	1.64	0.40	5.37
0.73	0.69	2.05	2.68	1.31	0.56	3.88	1.90	0.81	5.89
3.09	0.93	2.76	4.02	1.61	0.54	4.32	1.73	0.58	4.41
0.79	0.91	2.82	3.29	1.83	0.68	3.62	2.01	0.75	4.28
1.21	0.71	2.74	5.47	1.62	0.88	7.7	2.28	1.24	6.92
0.74	1.06	1.9	3.36	1.06	0.31	3.17	1.00	0.29	6.02
0.65	1	1.59	1.25	1.07	0.37	1.25	1.07	0.37	2.64
1.58	1.27	2.93	2.52	1.06	0.5	1.98	0.83	0.39	3.24
1.52	0.58	1.9	2.69	1.34	0.27	4.64	2.31	0.47	4.22
0.89	0.88	2.01	3.3	0.69	0.21	3.75	0.78	0.24	8.6
0.78	0.96	2.22	1.85	1.5	0.28	1.93	1.56	0.29	5.85
1.19	0.39	1.68	2.21	1.33	0.29	5.67	3.41	0.74	4.62
1.37	1.26	1.88	2.1	0.8	0.5	1.67	0.63	0.40	6.82
0.76	1.06	1.38	1.64	1.84	0.45	1.55	1.74	0.42	5.84
0.89	0.46	2.67	1.44	1.24	0.23	3.13	2.70	0.50	3.3
0.55	1.09	2.55	4.21	2.35	0.67	3.86	2.16	0.61	6.06
0.36	0.37	4.39	1.95	1.07	0.37	5.27	2.89	1.00	7.47
0.88	0.73	1.77	3.9	1.85	0.63	5.34	2.53	0.86	5.79
0.82	1.38	2.59	2.24	1.6	0.38	1.62	1.16	0.28	4.91
0.29	1.59	1.69	1.5	0.4	0.2	0.94	0.25	0.13	6.4
0.93	0.79	2.5	5.5	1.24	0.8	6.96	1.57	1.01	5.34
0.35	1.26	1.45	1.22	0.9	0.24	0.97	0.71	0.19	7.98
0.45	1.29	2	2.9	2	1.1	2.25	1.55	0.85	6.41
0.74	1.23	2.59	2.28	2.34	0.53	1.85	1.90	0.43	3.62
0.71	1	1.59	1.43	1.01	0.28	1.43	1.01	0.28	3.68
0.66	1.55	2.36	1.87	1.27	0.32	1.21	0.82	0.21	5.83
0.35	0.8	1.24	3.95	0.99	0.57	4.94	1.24	0.71	7.92
0.3	1.49	1.15	1.3	0.48	0.16	0.87	0.32	0.11	3.5
0.39	1.02	2.11	1.48	1.39	0.45	1.45	1.36	0.44	5.33
0.61	1.29	1.5	0.69	1.92	0.88	0.53	1.49	0.68	4.51
0.84	1.38	2.26	1.37	0.73	0.25	0.99	0.53	0.18	5.48
0.9	1.13	2.23	1.17	1.06	0.42	1.04	0.94	0.37	4.04
0.91	1.63	2.9	4.33	1.29	0.28	2.66	0.79	0.17	4.36
0.76	1.03	1.9	1.3	1.03	0.17	1.26	1.00	0.17	7.08
1.35	0.5	3.95	8.49	1.49	1.13	16.98	2.98	2.26	4.14
1.2	0.83	2.97	3.75	1.05	0.32	4.52	1.27	0.39	5.23
1.15	0.97	2.66	3.57	1.13	0.35	3.68	1.16	0.36	4.52

2.06	0.84	3.31	2.54	1.57	0.45	3.02	1.87	0.54	6.16
0.63	1.47	1.17	0.91	1.32	0.18	0.62	0.90	0.12	4.82
0.58	0.94	1.07	0.84	0.23	0.15	0.89	0.24	0.16	6.69
0.5	1.31	1.36	1.79	0.73	0.5	1.37	0.56	0.38	2.4
0.9	1.02	1.31	1.67	1.03	0.45	1.64	1.01	0.44	4
0.84	1.06	1.49	1.49	1.46	0.38	1.41	1.38	0.36	4.34
1.08	1.31	2.6	1.87	1.91	0.36	1.43	1.46	0.27	7.26
0.92	1.12	2.17	1.55	0.87	0.28	1.38	0.78	0.25	3.99
0.41	1.14	1.11	1.95	0.92	0.29	1.71	0.81	0.25	3.35
0.8	0.98	2.66	3.13	3.89	0.68	3.19	3.97	0.69	4.61
1.36	1.25	2.28	1.53	1.08	0.45	1.22	0.86	0.36	5.03
1.73	1.32	3.82	4.97	2.31	0.3	3.77	1.75	0.23	4.18
1.02	1.13	2.04	1.95	1.05	0.25	1.73	0.93	0.22	4.98
0.49	1.3	2.3	1.69	0.68	0.24	1.3	0.52	0.18	7.37
1.34	0.5	2.06	8.09	3.15	0.63	16.18	6.30	1.26	4.55
0.93	0.93	1.04	2.23	0.79	0.27	2.4	0.85	0.29	3.33
0.71	0.72	2.05	3.52	0.5	0.25	4.89	0.69	0.35	11.59
0.86	1.28	2.57	2.92	3.33	0.58	2.28	2.60	0.45	5.66
0.72	1.18	1.69	1.36	0.93	0.27	1.15	0.79	0.23	4.43
1.2	0.96	3.94	4.05	3.44	0.78	4.22	3.58	0.81	7.72
0.68	0.86	2.15	2.08	1.56	0.22	2.42	1.81	0.26	7.89
1.08	0.93	2.74	3.25	1.22	0.33	3.49	1.31	0.35	3.85
0.77	1.12	2.33	2.31	1.21	0.26	2.06	1.08	0.23	3.82
1.35	0.78	1.65	2.09	1.75	0.47	2.68	2.24	0.60	4.62
0.74	0.89	2.58	4.1	1.14	0.48	4.61	1.28	0.54	3.55
0.94	1.31	1.98	1.49	0.85	0.23	1.14	0.65	0.18	6.81
0.68	1.22	2.03	1.11	1.38	0.33	0.91	1.13	0.27	6.79
0.83	0.87	2.32	6.3	1.48	0.87	7.24	1.70	1.00	11.93
0.96	0.98	1.48	2.75	2.28	0.42	2.81	2.33	0.43	6.48
1.52	1.09	2.75	2.45	1.56	0.26	2.25	1.43	0.24	9.25
0.4	1.25	1.35	1.55	0.99	0.23	1.24	0.79	0.18	3.92
0.49	0.69	1.46	1.18	1.2	0.17	1.71	1.74	0.25	3.04
0.62	0.99	2.88	3.29	1.68	0.59	3.32	1.70	0.60	4.65
1.28	0.96	1.76	1.48	1.51	0.34	1.54	1.57	0.35	8.02
0.73	1.01	2.67	2.49	2.38	0.46	2.47	2.36	0.46	6.89
0.56	0.78	1.72	1.34	1.06	0.34	1.72	1.36	0.44	8.9
0.35	1.27	2.88	2.66	1.3	0.46	2.09	1.02	0.36	7.29
1.02	1.13	2.15	3.36	2.17	0.44	2.97	1.92	0.39	5.21
1.01	1.05	2.67	1.02	1.22	0.24	0.97	1.16	0.23	5.28
0.72	0.63	1.59	2.23	0.74	0.36	3.54	1.17	0.57	2.99
0.75	1.44	2.97	2.9	0.53	0.05	2.01	0.37	0.03	7.67
1.21	0.57	1.57	2.11	2.6	0.82	3.7	4.56	1.44	3.99
0.89	0.9	1.23	2.18	0.73	0.26	2.42	0.81	0.29	4.69
1.07	0.45	1.73	7.03	0.97	0.54	15.62	2.16	1.20	8.56

1.36	0.67	2.02	3.83	2.44	1.01	5.72	3.64	1.51	5.31
0.47	1.13	1.05	1.43	1.46	0.39	1.27	1.29	0.35	4.58
2.12	0.81	2.4	5.61	2.71	0.67	6.93	3.35	0.83	7.1
1.09	0.94	2.03	2.73	1.91	0.29	2.9	2.03	0.31	4.95
0.95	1.42	2.91	3.3	1.09	0.37	2.32	0.77	0.26	3.69
1.17	1.73	2.15	1.87	1.46	0.38	1.08	0.84	0.22	7.27
0.85	1.29	1.63	1.47	1.36	0.39	1.14	1.05	0.30	5.4
0.51	1.2	1.1	1.13	0.59	0.22	0.94	0.49	0.18	2.75
0.54	0.75	3	8.32	1.62	0.66	11.09	2.16	0.88	5.15
0.58	0.62	2.24	6.23	0.95	0.76	10.05	1.53	1.23	5.23
1.16	0.86	2.03	1.9	1.7	0.34	2.21	1.98	0.40	3.92
0.82	1.04	2.2	2.69	1.73	0.49	2.59	1.66	0.47	9.31
0.5	1.38	1.58	1.37	0.75	0.25	0.99	0.54	0.18	6.17
0.77	1.17	1.68	3.17	2.21	0.6	2.71	1.89	0.51	7.03
0.76	1.38	2.45	0.96	2.92	0.89	0.7	2.12	0.64	3.98
0.92	0.72	2.29	4.61	1.3	0.39	6.4	1.81	0.54	5.19
0.57	0.71	2.09	1.1	0.78	0.21	1.55	1.10	0.30	4.35
1.03	1.11	3.24	2.27	1.5	0.3	2.05	1.35	0.27	4.68
0.79	1.37	2.56	6.87	1.87	0.74	5.01	1.36	0.54	4.57
0.6	1.33	1.48	2.73	0.92	0.27	2.05	0.69	0.20	3.69
0.79	1.44	1.75	1.78	1.3	0.26	1.24	0.90	0.18	6.25
0.78	1.33	2.79	3.76	2.17	0.49	2.83	1.63	0.37	4.08
0.83	1.05	2.43	1.64	2.28	0.3	1.56	2.17	0.29	5.36
0.46	1.32	1.74	1.52	1.3	0.32	1.15	0.98	0.24	7.11
0.42	0.81	1.37	1.01	0.72	0.16	1.25	0.89	0.20	8.33
1.39	0.96	5.74	2.75	0.4	0.49	2.86	0.42	0.51	12.39
0.73	1.26	1.77	2.55	1.4	0.35	2.02	1.11	0.28	8.85
1.27	0.75	1.56	3.48	1.33	0.35	4.64	1.77	0.47	5.65
0.83	0.71	5.04	5.93	0.56	0.58	8.35	0.79	0.82	6.67
1.57	0.81	3.32	3.83	1.44	0.51	4.73	1.78	0.63	4.45
0.49	1.11	3.04	5.2	1.89	0.44	4.68	1.70	0.40	5.13
0.65	1.18	2.24	4.24	0.82	0.38	3.59	0.69	0.32	3.29
2.51	0.73	2.23	5.41	2.76	0.39	7.41	3.78	0.53	5.18
0.73	1.36	1.9	2.08	2.47	0.49	1.53	1.82	0.36	7.04
1.8	1.41	3.23	0.84	1.06	0.43	0.6	0.75	0.30	6.44
0.48	0.97	1.79	3.13	0.56	0.46	3.23	0.58	0.47	4.43
0.56	1.16	1.71	4.11	0.87	0.82	3.54	0.75	0.71	6.57
1.74	1.19	2.32	1.89	2.29	0.44	1.59	1.92	0.37	5.18
1.15	1.47	2.07	1.67	1.9	0.51	1.14	1.29	0.35	5.5
0.43	1.53	1.71	2.03	0.48	0.21	1.33	0.31	0.14	6.22
0.82	0.74	1.95	1.7	2.84	0.9	2.3	3.84	1.22	3.67
1.24	1.39	3.68	3.96	1.51	0.4	2.85	1.09	0.29	5.12
1.13	1.1	2.85	3.3	1.8	0.5	3	1.64	0.45	4.41
0.43	1.29	1.21	1.07	0.34	0.14	0.83	0.26	0.11	4.41

5.34	0.64	1.72	1.38	0.89	0.27	2.16	1.39	0.42	5.74
0.78	1.03	5.89	5.05	2.37	0.67	4.9	2.30	0.65	4.97
0.82	0.82	2.26	4.99	1.37	0.83	6.09	1.67	1.01	5.33
0.81	1.49	1.77	3.41	0.84	0.37	2.29	0.56	0.25	6.79
0.57	0.75	2.19	1.2	0.8	0.1	1.6	1.07	0.13	9.23
1.39	0.93	2.98	3.06	1.45	0.31	3.29	1.56	0.33	3.96
0.57	1.18	1.83	1.8	2.7	0.6	1.53	2.29	0.51	5.63
0.27	0.08	0.21	1.99	0.63	0.14	24.88	7.88	1.75	3.39
0.85	1.13	1.75	0.69	0.89	0.13	0.61	0.79	0.12	3.5
0.91	0.9	1.6	1.4	1.45	0.36	1.56	1.61	0.40	5.1
0.4	1.53	1.6	1.32	1.29	0.26	0.86	0.84	0.17	6.65
1.02	0.77	2.03	2.6	1.36	0.36	3.38	1.77	0.47	5.47
1.13	0.91	1.38	1.51	0.62	0.18	1.66	0.68	0.20	4.92
0.59	0.77	1.78	2.9	1.12	0.36	3.77	1.45	0.47	5.53
1.34	0.81	3.04	1.89	1.41	0.25	2.33	1.74	0.31	8.08
1	1.39	1.78	2.42	2.06	0.58	1.74	1.48	0.42	6.58
0.66	1.37	2.48	2.04	1.73	0.57	1.49	1.26	0.42	5.78
0.57	1.09	2.29	2.2	1.21	0.31	2.02	1.11	0.28	4.37
0.84	1.25	2.12	1.66	0.91	0.28	1.33	0.73	0.22	5.41
0.85	1.11	2.78	2.51	1	0.26	2.26	0.90	0.23	4.49
0.22	1.01	1.77	2.64	0.67	0.34	2.61	0.66	0.34	4.84
1.26	1.08	3.13	2.46	0.65	0.26	2.28	0.60	0.24	5.63
0.59	1.24	1.92	1.71	1.3	0.21	1.38	1.05	0.17	5.26
0.39	1.1	1.12	1.29	0.9	0.29	1.17	0.82	0.26	7.86
0.54	1.04	2.48	2.23	1.74	0.22	2.14	1.67	0.21	5.21
0.86	1.28	2.25	1.34	1.61	0.23	1.05	1.26	0.18	5.36
0.44	1.88	2.69	0.9	2.22	0.66	0.48	1.18	0.35	3.69
1.2	0.92	3.08	2	2.12	0.46	2.17	2.30	0.50	5.81
0.52	1.74	1.91	1.36	1.29	0.3	0.78	0.74	0.17	7.97
1	0.74	2.07	2.58	0.58	0.39	3.49	0.78	0.53	7.66
1.23	1.29	2.33	0.81	1.83	0.34	0.63	1.42	0.26	5.28
1.08	1.53	3.75	2.22	2.13	0.45	1.45	1.39	0.29	4.86
1.68	0.82	2.43	3.04	1.57	0.52	3.71	1.91	0.63	6.71
1.05	1.27	2.96	1.73	0.91	0.2	1.36	0.72	0.16	5.39
0.67	1.07	2.3	2.63	1.35	0.39	2.46	1.26	0.36	3.67
0.74	0.64	2.34	1.65	0.94	0.42	2.58	1.47	0.66	3.28
0.81	0.71	1.93	1.81	1.19	0.27	2.55	1.68	0.38	6.16
0.5	1.1	1.46	1.86	0.74	0.15	1.69	0.67	0.14	4.65
0.58	0.66	1.12	1.22	0.66	0.14	1.85	1.00	0.21	2.75
0.44	0.91	1.09	0.51	0.21	0.09	0.56	0.23	0.10	4.66
1.48	1.01	3.45	3.51	2.23	0.54	3.48	2.21	0.53	6.44
1.43	0.72	2.42	1.24	1.14	0.23	1.72	1.58	0.32	5.63
0.74	0.96	2.73	4.49	2.15	0.45	4.68	2.24	0.47	6.96
0.7	0.86	1.23	2.72	1.09	0.35	3.16	1.27	0.41	5.9

0.61	1.05	2.2	1.41	0.82	0.27	1.34	0.78	0.26	3.53
0.45	0.8	2.34	3.83	1.16	0.33	4.79	1.45	0.41	5.48
0.31	1.17	1.53	1.55	0.59	0.32	1.32	0.50	0.27	3.54
1.9	0.51	2.2	2.21	0.88	0.52	4.33	1.73	1.02	6.95
1.36	0.92	2.91	4.45	2.83	0.65	4.84	3.08	0.71	4.99
0.65	1.32	1.31	1.82	0.7	0.31	1.38	0.53	0.23	5.54
0.68	1.06	2.33	3.68	1.46	0.52	3.47	1.38	0.49	6.52
0.54	0.84	2.21	3.27	1.07	0.39	3.89	1.27	0.46	6.87
0.45	1.36	2.23	1.69	0.61	0.29	1.24	0.45	0.21	5.55
1.63	0.65	2.25	3.72	2.76	0.47	5.72	4.25	0.72	6.37
1.28	0.77	2.33	2.76	2.05	0.41	3.58	2.66	0.53	6.84
0.76	1.48	2.22	3.23	2.03	0.74	2.18	1.37	0.50	4.66
1.38	1.03	2.63	3.22	1.99	0.42	3.13	1.93	0.41	4.44
1.56	0.89	2.23	2.39	1.25	0.27	2.69	1.40	0.30	7.22
0.88	1.22	3.38	3.41	1.21	0.4	2.8	0.99	0.33	5.46
1.18	0.72	1.71	2.41	0.83	0.28	3.35	1.15	0.39	6.48
0.98	1.01	3.49	4.75	1.63	0.67	4.7	1.61	0.66	7.36
0.55	0.6	1.34	2.02	0.7	0.3	3.37	1.17	0.50	9.84
1.28	0.24	2.93	2.26	2.31	0.44	9.42	9.63	1.83	3.45
0.61	1.12	1.93	1.5	0.76	0.35	1.34	0.68	0.31	3.89
1.29	1.25	3.17	3.61	2.2	0.22	2.89	1.76	0.18	3.87
0.47	1.12	1.03	0.82	0.4	0.17	0.73	0.36	0.15	4.5
2.01	0.96	3.98	2.65	1.91	0.33	2.76	1.99	0.34	5.66
0.71	1.44	2.14	1.8	1.75	0.39	1.25	1.22	0.27	5.26
0.74	0.83	2.43	3.75	2.4	0.53	4.52	2.89	0.64	4.61
1.52	0.88	2.18	1.8	1.54	0.28	2.05	1.75	0.32	2.7
0.95	0.81	1.39	1.89	0.82	0.28	2.33	1.01	0.35	4.37
0.68	2.18	1.93	3.34	1.09	0.43	1.53	0.50	0.20	5.51
0.79	0.93	0.49	2.22	1.05	0.35	2.39	1.13	0.38	6.37
0.64	1.05	1.94	4.11	1.02	0.42	3.91	0.97	0.40	4.37
0.99	0.94	2.83	3.58	2.25	0.36	3.81	2.39	0.38	7.93
0.89	1.53	2.15	2.5	2.4	0.4	1.63	1.57	0.26	4.5
0.8	0.59	2.16	2.28	0.43	0.21	3.86	0.73	0.36	4.09
0.67	1.29	2.64	4.27	1.01	0.98	3.31	0.78	0.76	2.77
1.85	0.93	2.37	2.58	1.84	0.26	2.77	1.98	0.28	3.53
2	1.03	2.26	2.3	1.43	0.37	2.23	1.39	0.36	3.73
0.59	1.26	2.45	1.75	0.83	0.2	1.39	0.66	0.16	5.56
0.67	1.04	2.23	1.7	0.86	0.32	1.63	0.83	0.31	3.92
0.47	1.43	2.15	1.97	0.95	0.3	1.38	0.66	0.21	7.07
0.69	0.65	2.81	2.2	1.14	0.26	3.38	1.75	0.40	5.1
0.48	1.51	2.04	4.13	1.56	0.42	2.74	1.03	0.28	4.8
0.55	0.7	1.53	2.27	1.29	0.55	3.24	1.84	0.79	5.06
1.12	1.16	1.49	1.87	0.61	0.23	1.61	0.53	0.20	4.72
0.73	1.01	1.79	2.85	1.22	0.23	2.82	1.21	0.23	5.26

0.71	1.21	2.02	1.71	1.04	0.28	1.41	0.86	0.23	6.21
0.95	1.54	3.23	2.1	1.32	0.38	1.36	0.86	0.25	5.35
0.43	1.51	2.1	1.03	0.52	0.15	0.68	0.34	0.10	5.81
0.74	0.86	4.21	3.29	0.91	0.7	3.83	1.06	0.81	7.03
1.61	0.73	2.92	4.06	0.95	0.4	5.56	1.30	0.55	4.08
0.85	1.33	2.04	7.4	0.72	0.49	5.56	0.54	0.37	4.65
0.97	0.97	2.77	3.64	1.83	0.32	3.75	1.89	0.33	7.43
1.02	1.3	3.68	2.82	0.55	0.21	2.17	0.42	0.16	6.36
0.77	1.07	1.32	2.17	1.85	0.59	2.03	1.73	0.55	5.3
1.14	1.2	4.71	4.19	1.7	0.64	3.49	1.42	0.53	5.78
1.01	0.93	2.48	2.23	1.95	0.24	2.4	2.10	0.26	5.86
1.52	0.81	1.71	0.55	0.73	0.25	0.68	0.90	0.31	4.31
0.58	1.2	1.98	2.23	0.61	0.46	1.86	0.51	0.38	6.2
0.32	1.17	1.32	1.21	0.51	0.29	1.03	0.44	0.25	4.7
0.92	0.87	2.77	2.32	2.74	0.51	2.67	3.15	0.59	5.22
0.31	0.9	1.3	2.93	0.54	0.24	3.26	0.60	0.27	7.11
0.58	1.23	2.28	2.78	1.96	0.54	2.26	1.59	0.44	7.4
1.67	1.58	4.49	2.06	2.99	0.43	1.3	1.89	0.27	6.4
0.66	1.48	1.42	1.76	1.07	0.23	1.19	0.72	0.16	6.09
1.12	1.1	3.12	1.69	1.19	0.38	1.54	1.08	0.35	4.49
0.86	0.86	2.65	2.2	0.8	0.27	2.56	0.93	0.31	7.26
0.82	1.31	2.58	4.35	1.72	0.34	3.32	1.31	0.26	7.09
0.4	1.04	1.75	1.05	0.93	0.28	1.01	0.89	0.27	4.44
0.37	1.59	1.68	1.44	2.14	0.35	0.91	1.35	0.22	4
0.79	0.74	4.74	5.08	0.93	0.64	6.86	1.26	0.86	3.46
2.38	1.08	2.61	2.43	2.22	0.53	2.25	2.06	0.49	4.7
0.57	0.63	1.07	7.67	0.66	1.35	12.17	1.05	2.14	12.39
0.7	0.85	2	1.3	1.2	0.5	1.53	1.41	0.59	4.05
0.37	2.23	1.53	1.6	2.92	0.54	0.72	1.31	0.24	5.43
0.43	1.14	0.97	3.31	1.25	0.99	2.9	1.10	0.87	4.3
0.77	1.15	1.97	7.3	1.27	0.59	6.35	1.10	0.51	4.19
2.29	0.89	1.89	2.29	1.08	0.27	2.57	1.21	0.30	3.5
0.73	0.97	1.9	0.98	0.28	0.09	1.01	0.29	0.09	5.89
1.34	0.92	2.9	2.42	0.86	0.29	2.63	0.93	0.32	4.28
0.67	0.97	1.58	1.26	0.38	0.12	1.3	0.39	0.12	6.96
0.8	1.6	4.21	5.24	3.16	0.62	3.28	1.98	0.39	5.72
1.4	0.3	2.73	3.19	2.04	0.67	10.63	6.80	2.23	4.02
2.16	0.84	2.89	3.21	2.09	0.32	3.82	2.49	0.38	5.29
0.91	1.15	3.03	1.76	1.08	0.26	1.53	0.94	0.23	6.23
0.56	0.97	2.83	2.12	0.97	0.32	2.19	1.00	0.33	8.43
0.72	1.59	2.28	1.46	1.24	0.23	0.92	0.78	0.14	5.99
0.7	1	0.9	3.82	1.29	0.27	3.82	1.29	0.27	5.2
0.78	1.18	0.93	1.44	1.06	0.14	1.22	0.90	0.12	4.27
2.03	0.75	2.71	5.7	1.29	0.38	7.6	1.72	0.51	10.25

1.25	0.98	1.95	4.24	1.93	0.54	4.33	1.97	0.55	5.05
0.65	1.22	2.06	1.99	0.69	0.27	1.63	0.57	0.22	3.42
0.81	1.25	2.23	2.98	2.12	0.48	2.38	1.70	0.38	6.56
0.81	1.44	1.55	2.2	1.05	0.34	1.53	0.73	0.24	4.71
1.83	1.19	3.11	4.96	2.64	0.6	4.17	2.22	0.50	6.96
0.66	0.78	1.48	2.48	1	0.44	3.18	1.28	0.56	5.79
0.99	1.03	1.81	1.93	1.24	0.29	1.87	1.20	0.28	5.73
0.99	1.13	2.47	1.12	0.56	0.13	0.99	0.50	0.12	5
0.77	0.97	2.42	1.75	0.82	0.52	1.8	0.85	0.54	4.54
0.9	0.82	1.69	2.53	1.85	0.55	3.09	2.26	0.67	4.63
0.49	0.96	1.13	0.9	0.72	0.17	0.94	0.75	0.18	6.69
1.15	1	2.72	3.15	2.32	0.55	3.15	2.32	0.55	4.16
1.43	0.76	2.54	3.89	1.13	0.83	5.12	1.49	1.09	4.28
0.6	1.88	1.93	2.79	1.54	0.24	1.48	0.82	0.13	5.5
1.17	0.84	2.79	7.43	2.03	1.21	8.85	2.42	1.44	2.83
0.87	1.61	2.71	7.74	0.93	0.5	4.81	0.58	0.31	4.88
1.01	0.89	2.04	4.32	1.27	0.24	4.85	1.43	0.27	5.42
0.56	1.22	1.76	2.81	1.52	0.4	2.3	1.25	0.33	6.54
0.63	1.26	1.65	1.4	1.2	0.4	1.11	0.95	0.32	3.6
0.39	1.64	1.15	1.16	0.82	0.35	0.71	0.50	0.21	4.75
0.92	1.03	2.51	4.16	1.38	0.53	4.04	1.34	0.51	4.63
3.35	0.76	2.32	4.89	2.45	0.44	6.43	3.22	0.58	4.79
1.26	0.92	2.61	4.78	2.54	0.68	5.2	2.76	0.74	2.29
0.66	1.16	2.92	3.69	1.76	0.44	3.18	1.52	0.38	8.15
0.62	0.61	1.91	3.15	1.94	0.33	5.16	3.18	0.54	7.65
0.89	1.09	3.11	6.83	0.88	0.3	6.27	0.81	0.28	4.81
1.64	0.76	2.13	1.86	1.07	0.55	2.45	1.41	0.72	4.71
1.49	0.75	2.14	2.99	3.35	0.68	3.99	4.47	0.91	6.08
0.87	1.18	2.2	3.33	1.58	0.76	2.82	1.34	0.64	3.82
1.1	1.19	1.84	3.07	0.87	0.28	2.58	0.73	0.24	7.62
0.79	0.92	1.89	4.02	1.51	0.72	4.37	1.64	0.78	5.99
0.68	0.94	1.93	1.88	0.73	0.27	2	0.78	0.29	4.08
0.4	1.2	2.23	1.57	0.75	0.23	1.31	0.63	0.19	5.36
0.78	0.61	1.31	3.04	0.43	0.37	4.98	0.70	0.61	6.25
0.76	1.12	2.13	2.04	1.03	0.39	1.82	0.92	0.35	6.81
0.47	1.54	1.77	1.6	1.5	0.3	1.04	0.97	0.19	4.52
0.56	0.97	1.94	1.9	1.32	0.39	1.96	1.36	0.40	5.08
0.73	1.23	2.86	3.07	2.19	0.62	2.5	1.78	0.50	7.45
2.29	0.63	1.87	3.16	1.59	0.47	5.02	2.52	0.75	5.78
1.28	0.7	1.85	1.28	2.12	0.24	1.83	3.03	0.34	4.71
0.49	0.87	3.27	2.21	1.38	0.42	2.54	1.59	0.48	4.8
0.86	0.78	2.15	1.52	1.14	0.18	1.95	1.46	0.23	4.24
0.53	0.89	4.59	1.54	0.49	0.13	1.73	0.55	0.15	8.75
1.19	0.86	3.02	3.04	2	0.53	3.53	2.33	0.62	6.88

1.39	0.9	2.87	2.15	0.63	0.29	2.39	0.70	0.32	7.92
0.73	1.33	2.08	1.45	0.59	0.17	1.09	0.44	0.13	4.94
0.73	1.26	2.7	1.63	1.13	0.33	1.29	0.90	0.26	7.57
0.59	1.07	2.61	1.67	1.65	0.35	1.56	1.54	0.33	4.99
1.27	0.65	1.05	1.1	0.49	0.33	1.69	0.75	0.51	5.59
0.99	0.77	2.3	1.63	0.78	0.19	2.12	1.01	0.25	3.3
0.88	1.12	3.53	4.26	1.56	0.57	3.8	1.39	0.51	4.52
0.83	1.41	2.65	2.72	1.45	0.49	1.93	1.03	0.35	5.59
1.39	0.88	3.85	3.48	1.59	0.42	3.95	1.81	0.48	7.01
0.44	0.93	1.91	4.17	1.01	0.39	4.48	1.09	0.42	5.43
0.51	0.62	1.32	5.65	2.9	0.43	9.11	4.68	0.69	5.31
0.79	1.33	2.76	3.58	0.98	0.36	2.69	0.74	0.27	6.67
0.83	0.97	3.34	2.43	1.26	0.68	2.51	1.30	0.70	4.42
0.46	0.88	1.95	2.85	1.07	0.26	3.24	1.22	0.30	4.98
0.71	0.67	2.85	6.94	1.21	0.85	10.36	1.81	1.27	3.04
0.38	1.56	2.13	3.01	2.14	0.95	1.93	1.37	0.61	3.27
0.5	1.3	2.69	1.14	1.15	0.17	0.88	0.88	0.13	6.47
0.96	0.77	2.63	4.51	2.71	0.82	5.86	3.52	1.06	8.54
0.81	0.86	1.68	1.2	1.5	0.22	1.4	1.74	0.26	4.76
1.68	1.06	2.48	3.57	2.31	0.55	3.37	2.18	0.52	7.46
0.52	1.37	2.66	1.56	0.67	0.19	1.14	0.49	0.14	5.24
0.62	1.47	2.15	3.73	0.63	0.32	2.54	0.43	0.22	4.41
0.4	1.1	1.5	0.95	0.71	0.29	0.86	0.65	0.26	3.86
0.75	1	2.39	3.78	2.31	0.22	3.78	2.31	0.22	4.35
0.58	0.82	2.56	3.47	1.15	0.57	4.23	1.40	0.70	3.54
1.23	0.95	1.94	1.68	1.87	0.34	1.77	1.97	0.36	3.24
0.9	0.96	3.77	2.35	1.75	0.33	2.45	1.82	0.34	6.44
0.66	0.95	1.36	1.78	1.69	0.46	1.87	1.78	0.48	3.47
1.12	1.08	2.85	2.87	1.58	0.27	2.66	1.46	0.25	5.97
0.47	1.04	1.44	1.46	1.04	0.2	1.4	1.00	0.19	5.62
2.45	0.81	1.83	3.32	1.41	0.27	4.1	1.74	0.33	4.86
0.8	1.15	2.23	1.81	1.04	0.21	1.57	0.90	0.18	6.71
0.8	0.85	4.16	2.6	1.63	0.26	3.06	1.92	0.31	4.21
0.55	1.16	2.54	3.23	1.94	0.46	2.78	1.67	0.40	6.36
0.55	1.07	1.26	1.37	0.5	0.2	1.28	0.47	0.19	2
0.45	0.79	1.14	1.38	1	0.32	1.75	1.27	0.41	5.64
0.53	0.89	1.3	3.11	2.06	0.41	3.49	2.31	0.46	2.27
0.29	1.07	2.13	6.52	0.56	0.27	6.09	0.52	0.25	8.04
0.87	1.44	2.09	2.29	0.9	0.3	1.59	0.63	0.21	6.23
0.98	0.97	1.87	3.2	0.94	0.47	3.3	0.97	0.48	6.22
1.28	0.99	2.73	4.48	1.99	0.37	4.53	2.01	0.37	4.4
1.22	1.23	2.68	3.33	1.44	0.38	2.71	1.17	0.31	5.05
1.42	1.25	3.34	1.58	2.16	0.26	1.26	1.73	0.21	5.69
0.69	1	1.94	0.87	0.99	0.12	0.87	0.99	0.12	3.93

0.37	0.89	1.86	2.46	0.54	0.34	2.76	0.61	0.38	3.67
0.79	1.04	3.12	2.23	1.02	0.34	2.14	0.98	0.33	4.69
0.69	0.7	2.76	3.65	2.17	0.47	5.21	3.10	0.67	3.93
3.22	0.85	2.74	2.58	2.27	0.28	3.04	2.67	0.33	5.68
0.64	1.78	1.65	2.18	1.29	0.36	1.22	0.72	0.20	5.57
0.78	1.18	2.01	2.36	1.32	0.37	2	1.12	0.31	6.14
0.81	1.49	2.18	8.29	1.54	0.64	5.56	1.03	0.43	9.66
0.68	1.58	2.18	2.75	2.35	0.53	1.74	1.49	0.34	3.86
1.02	0.95	1.54	1.8	1.2	0.5	1.89	1.26	0.53	4.61
1.04	0.85	2.08	4.74	1.2	0.36	5.58	1.41	0.42	5.36
3.35	0.89	2.58	3.55	2.85	0.35	3.99	3.20	0.39	6.56
0.6	1.25	1.25	1.41	0.51	0.23	1.13	0.41	0.18	5.6
0.39	1.08	1.95	1.32	0.44	0.36	1.22	0.41	0.33	8.16
1.64	0.83	3.04	4.54	1.09	0.56	5.47	1.31	0.67	6.07
1.25	0.8	2.5	3.49	1.41	0.24	4.36	1.76	0.30	6.09
0.55	1.11	2.5	2.38	1.7	0.44	2.14	1.53	0.40	6.9
0.41	1.42	1.88	2.07	0.73	0.38	1.46	0.51	0.27	4.58
1.25	0.89	3.64	1.56	2.16	0.28	1.75	2.43	0.31	8.63
0.5	0.75	3.04	3.9	1.1	0.6	5.2	1.47	0.80	6.06
0.67	1.37	2.61	1.84	1.24	0.28	1.34	0.91	0.20	3.23
1.83	1.01	2.71	2.4	0.9	0.3	2.38	0.89	0.30	5.55
1.72	0.92	3	2.56	1.6	0.29	2.78	1.74	0.32	8.28
0.61	1.09	2.13	1.38	1.15	0.33	1.27	1.06	0.30	3.57
1.05	1.1	3.32	2.95	2.08	0.55	2.68	1.89	0.50	3.76
0.71	0.8	2.07	4.54	1.85	0.49	5.68	2.31	0.61	6.01
0.87	0.11	1.08	2.69	1.98	0.37	24.45	18.00	3.36	2.96
2.23	0.85	2.28	2.8	1.6	0.5	3.29	1.88	0.59	6.08
2.66	0.94	0.99	4.09	0.78	0.16	4.35	0.83	0.17	4.39
0.69	1.34	1.92	2.8	1.6	0.5	2.09	1.19	0.37	6.09
0.98	1.23	2.39	1.85	1.86	0.4	1.5	1.51	0.33	4.89
0.44	1.19	1.23	1.36	0.61	0.21	1.14	0.51	0.18	6.32
1.21	1.27	0.82	1.75	0.94	0.23	1.38	0.74	0.18	7.12
1.16	0.74	1.78	1.47	1.79	0.53	1.99	2.42	0.72	6.08
0.58	1.89	2.65	2.9	2.1	0.5	1.53	1.11	0.26	3.56
0.52	0.86	1.14	1.3	1.5	0.5	1.51	1.74	0.58	2.78
1	0.93	2.62	2.7	1.6	0.4	2.9	1.72	0.43	7.21
0.67	1.29	2	3.43	1.31	0.37	2.66	1.02	0.29	6.46
1.17	0.73	1.48	4.52	1.17	0.43	6.19	1.60	0.59	4.47
1.23	0.85	2.88	4	1.7	0.5	4.71	2.00	0.59	7.74
0.98	0.77	1.82	3.35	1.69	0.38	4.35	2.19	0.49	4.62
1.15	1.14	2.64	2.36	0.8	0.41	2.07	0.70	0.36	5.27
1.53	0.92	4.46	2.86	2.01	0.62	3.11	2.18	0.67	3.98
0.54	1.16	2.9	5.03	1.26	0.49	4.34	1.09	0.42	4.34
1.29	1.16	2.08	8.36	0.9	0.35	7.21	0.78	0.30	6.52

3.62	0.12	0.96	2.39	0.41	0.24	19.92	3.42	2.00	3.68
0.91	0.94	2.15	3.8	1.7	0.5	4.04	1.81	0.53	5.65
0.36	1.22	1.65	1.05	0.54	0.21	0.86	0.44	0.17	5.38
1.78	1.4	3.17	3.4	2.2	0.6	2.43	1.57	0.43	4.11
0.5	0.94	1.56	2.19	0.56	0.42	2.33	0.60	0.45	4.58
1.08	0.95	2.92	1.9	1.3	0.3	2	1.37	0.32	2.55
0.67	1.35	2.96	3.11	1.8	0.33	2.3	1.33	0.24	5.05
2.51	0.73	2.99	2.72	1.78	0.47	3.73	2.44	0.64	4.58
0.72	1.32	1.07	1.1	1	0.2	0.83	0.76	0.15	3.04
0.8	1.02	2.82	2.5	2	0.3	2.45	1.96	0.29	5.49
1.37	1.03	1.98	2.02	2.25	0.41	1.96	2.18	0.40	5.69
0.34	1.16	0.95	2.54	0.51	0.24	2.19	0.44	0.21	5.5
0.63	2.17	3.32	2.9	1.8	0.4	1.34	0.83	0.18	3.79
1.18	1.31	1.74	1.74	1.46	0.2	1.33	1.11	0.15	4.13
0.51	0.83	1.49	2.65	1.14	0.57	3.19	1.37	0.69	6.31
0.98	1.54	2.41	3.74	1.83	0.27	2.43	1.19	0.18	4.75
0.22	1.4	1.99	5.53	0.83	0.17	3.95	0.59	0.12	5.26
1.29	1.11	2.91	5.5	2.6	0.6	4.95	2.34	0.54	4.72
0.92	0.74	2.2	2.7	2	0.3	3.65	2.70	0.41	5.43
1.67	1.12	2.84	1.89	1.65	0.4	1.69	1.47	0.36	4.39
0.4	0.88	2.63	1.9	1.1	0.4	2.16	1.25	0.45	5.12
1.23	1.13	3.33	3.85	1.3	0.76	3.41	1.15	0.67	4.16
0.87	1.37	2.7	4.81	2.28	0.55	3.51	1.66	0.40	7.52
0.69	1.37	3.19	1.41	1.35	0.42	1.03	0.99	0.31	3.91
0.97	1.25	3.53	0.89	0.75	0.26	0.71	0.60	0.21	3.56
0.58	1	2.95	6.81	2.39	0.49	6.81	2.39	0.49	3.65
0.52	1.21	1.63	1.8	1.09	0.31	1.49	0.90	0.26	5.77
0.65	0.57	1.27	1.36	1.24	0.31	2.39	2.18	0.54	5.39
0.91	0.79	2.33	2	1.4	0.3	2.53	1.77	0.38	4.67
0.96	1.6	3	3.6	1.2	0.3	2.25	0.75	0.19	5.3
1.72	0.62	3.18	4.01	2.63	0.46	6.47	4.24	0.74	4.55
0.55	1.44	2.36	1.46	2.13	0.59	1.01	1.48	0.41	5.26
142	102.6	2.42	4.66	2.42	0.54	0.05	0.02	0.01	6.01
0.41	0.69	1.97	5.64	0.94	0.37	8.17	1.36	0.54	5.72
0.82	1.32	1.95	12.21	1.96	0.85	9.25	1.48	0.64	5.86
0.35	0.92	2.67	2.3	1.66	0.25	2.5	1.80	0.27	6.63
0.28	1.53	1.57	2.14	1.84	0.62	1.4	1.20	0.41	5.75
1.34	1.05	2.78	3.23	1.68	0.35	3.08	1.60	0.33	4.64
0.44	1.2	1.81	1.48	0.53	0.17	1.23	0.44	0.14	7.15
0.51	0.77	2.14	1.27	0.59	0.21	1.65	0.77	0.27	5.88
0.98	0.99	1.59	1.73	2.01	0.27	1.75	2.03	0.27	3.95
1.04	0.71	2.55	6.7	2.2	0.5	9.44	3.10	0.70	5
1.79	0.77	3.28	3.4	2.8	0.6	4.42	3.64	0.78	5.22
1.05	0.98	2.25	2.56	1.63	0.3	2.61	1.66	0.31	5.1

0.47	0.9	2.82	2.9	0.97	0.48	3.22	1.08	0.53	3.87
0.89	1.23	2.72	2.38	1.21	0.48	1.93	0.98	0.39	5.2
0.61	1.28	1.58	6	0.96	0.41	4.69	0.75	0.32	3.98
1.12	1.24	4.4	5.53	1.82	0.93	4.46	1.47	0.75	5.91
0.95	1.06	2.53	1.98	0.98	0.37	1.87	0.92	0.35	7.76
0.2	1.12	1.55	1.5	0.51	0.22	1.34	0.46	0.20	4.62
0.53	0.98	1.81	1.97	0.65	0.39	2.01	0.66	0.40	6.93
0.79	0.86	2.01	0.95	0.85	0.19	1.1	0.99	0.22	5.27
0.77	1.22	1.47	2.8	1.7	0.8	2.3	1.39	0.66	3.32
0.78	1.09	2.24	2.6	1.7	0.5	2.39	1.56	0.46	3.53
0.61	1.03	2.69	1.42	2.12	0.35	1.38	2.06	0.34	4.57
0.53	1.41	1.59	2.13	0.89	0.21	1.51	0.63	0.15	3.96
0.97	1.26	1.33	1.4	0.9	0.2	1.11	0.71	0.16	5.98
0.91	0.65	2.58	1.35	0.99	0.29	2.08	1.52	0.45	4.3
1.57	1.33	2.28	3.77	0.95	0.38	2.83	0.71	0.29	3.74
1.59	1.41	2.05	1.48	1.1	0.21	1.05	0.78	0.15	6.19
1.06	1	3.26	1.5	0.96	0.28	1.5	0.96	0.28	4.6
0.95	1	2.39	1.76	0.93	0.33	1.76	0.93	0.33	4.05
0.34	0.8	1.35	1.4	1	0.2	1.75	1.25	0.25	5.56
1.03	1.36	2.94	2.55	1.49	0.16	1.88	1.10	0.12	6.06
1.09	0.85	2.05	4.42	1.68	0.31	5.2	1.98	0.36	3.49
0.81	0.8	2.27	2.67	1.68	0.48	3.34	2.10	0.60	5.58
0.96	0.8	2.02	1.51	1.03	0.67	1.89	1.29	0.84	5.52
0.94	1.11	1.76	1.56	0.93	0.17	1.41	0.84	0.15	2.22
0.09	1.42	1.04	1.38	1.01	0.2	0.97	0.71	0.14	3.89
1.13	1.46	3.01	1.54	1.66	0.35	1.05	1.14	0.24	3.89
0.53	1.28	2.8	2.36	0.91	0.22	1.84	0.71	0.17	4.6
1.61	0.84	2.33	4	1.5	0.4	4.76	1.79	0.48	5.7
0.94	1.36	2.35	5.44	2.15	0.86	4	1.58	0.63	4.88
1.51	0.66	2.56	3.55	1.57	0.56	5.38	2.38	0.85	5.26
1.13	1.54	2.05	2.76	0.93	0.5	1.79	0.60	0.32	4.53
1.83	0.98	2.22	2.68	1.3	0.27	2.73	1.33	0.28	8.2
0.91	1.36	2.5	5.55	2.89	1.34	4.08	2.13	0.99	5.93
1.26	0.96	2.16	1.89	1.78	0.22	1.97	1.85	0.23	5.85
1.65	1.11	2.33	3.86	0.85	0.34	3.48	0.77	0.31	5.66
0.81	1.41	0.81	1.47	1.09	0.15	1.04	0.77	0.11	5.64
0.48	1.22	2.08	3.59	2.99	0.6	2.94	2.45	0.49	4.38
0.85	0.8	1.91	4.3	1.2	0.5	5.38	1.50	0.63	6.55
0.79	0.9	2.63	2.99	1.57	0.35	3.32	1.74	0.39	3.86
0.38	1.58	1.93	1.69	1.08	0.34	1.07	0.68	0.22	3.09
1.92	0.73	2.29	7.29	1.13	0.27	9.99	1.55	0.37	8.3
1.85	0.62	2.44	2.82	2.05	0.25	4.55	3.31	0.40	5.15
0.69	0.98	2.96	2.57	1	0.53	2.62	1.02	0.54	6.53
1.63	1.32	2.54	3.31	3.37	0.73	2.51	2.55	0.55	6.29

1.07	0.65	1.51	6.48	1.59	0.51	9.97	2.45	0.78	4.49
2.33	0.54	2.61	2.93	1.25	0.41	5.43	2.31	0.76	6.26
2.03	0.69	1.61	2.19	1.3	0.31	3.17	1.88	0.45	4.99
0.85	1.34	2.91	2.65	1.54	0.55	1.98	1.15	0.41	5.14
1.38	0.62	2.16	5.29	0.88	0.42	8.53	1.42	0.68	4.36
0.75	1.15	2.38	1.53	0.93	0.54	1.33	0.81	0.47	3.38
0.66	1.13	1.57	1.27	0.79	0.32	1.12	0.70	0.28	4.73
1.87	1.08	3.62	3.99	1.97	0.38	3.69	1.82	0.35	5.04
0.91	0.92	1.98	3.26	1.48	0.41	3.54	1.61	0.45	5.55
0.88	1.16	2.27	1.5	1.8	0.4	1.29	1.55	0.34	7.67
0.98	0.94	2.13	3.8	2.7	0.6	4.04	2.87	0.64	5.48
0.53	1.09	1.45	2.98	1.31	0.5	2.73	1.20	0.46	5.56
0.73	1.13	1.86	3.73	2.29	0.63	3.3	2.03	0.56	4.7
2.27	0.72	3.09	3.82	2.43	0.66	5.31	3.38	0.92	6.25
0.8	1.01	2.08	3.95	2.03	0.6	3.91	2.01	0.59	6.78
0.63	1	1.65	2.3	1.5	0.4	2.3	1.50	0.40	4.56
0.27	1.01	1.11	1.31	0.45	0.2	1.3	0.45	0.20	4.67
0.46	0.76	2.06	2.17	1.13	0.25	2.86	1.49	0.33	5.21
1.81	0.39	2.78	2.05	1.07	0.49	5.26	2.74	1.26	3.81
1.21	1.03	2.32	4.95	2.58	0.42	4.81	2.50	0.41	5.9
0.68	0.84	1.82	4.2	1.1	0.5	5	1.31	0.60	6.49
1.02	1.18	3.05	2.07	2.24	0.55	1.75	1.90	0.47	4.44
1.28	0.85	2.75	1.95	1.15	0.44	2.29	1.35	0.52	5.5
0.8	1.23	2.55	2.27	3.06	0.36	1.85	2.49	0.29	3.78
1.26	1.17	3.01	2.35	1.26	0.65	2.01	1.08	0.56	4.9
0.42	1.88	2	1.96	2.32	0.48	1.04	1.23	0.26	3.6
0.79	0.7	1	1.7	0.8	0.3	2.43	1.14	0.43	13
0.58	1.02	2.01	3.8	1.1	0.4	3.73	1.08	0.39	4.85
0.33	1.25	1.38	1.64	0.74	0.21	1.31	0.59	0.17	4.05
1.09	1.09	3.88	2.75	1.83	0.43	2.52	1.68	0.39	5.26
1.88	0.87	2.56	2.9	1.1	0.3	3.33	1.26	0.34	5.7
0.85	1.02	1.66	1.19	1.25	0.37	1.17	1.23	0.36	4.77
0.62	1.06	2.11	2.15	0.63	0.21	2.03	0.59	0.20	5.73
0.47	1.36	1.9	1.55	1.87	0.32	1.14	1.38	0.24	4.04
0.75	1.26	1.84	2.69	1.19	0.41	2.13	0.94	0.33	5.36
0.36	1.02	1.63	4.94	1.5	0.49	4.84	1.47	0.48	5.8
0.34	1.09	1.97	3.68	0.8	0.35	3.38	0.73	0.32	5.81
1.15	0.84	1.85	8.59	1.31	1.2	10.23	1.56	1.43	4.49
0.85	1.16	2.9	1.59	0.87	0.22	1.37	0.75	0.19	5.53
1.1	0.56	2.92	3.32	1.36	0.62	5.93	2.43	1.11	5.47
0.71	1.12	1.68	1.22	1.16	0.22	1.09	1.04	0.20	5.78
1.26	0.85	2.24	2.63	1.57	0.3	3.09	1.85	0.35	5.07
0.31	1.89	1.94	2.53	0.9	0.26	1.34	0.48	0.14	4.47
0.96	0.8	2.27	2.68	1.67	0.29	3.35	2.09	0.36	6

1.01	0.77	3.02	2.07	1.36	0.48	2.69	1.77	0.62	4.62
0.58	0.91	2.48	5.41	1.18	0.58	5.95	1.30	0.64	5.39
0.81	0.91	2.96	2.6	2.17	0.53	2.86	2.38	0.58	4.08
0.79	0.96	1.53	2.02	1.15	0.35	2.1	1.20	0.36	3.64
1.03	1.04	1.84	5.07	2.05	0.68	4.88	1.97	0.65	5.75
1.43	2	1.82	4.79	1.89	0.44	2.4	0.95	0.22	7.12
1.35	1.36	2.09	2.68	2.91	0.53	1.97	2.14	0.39	3.99
0.85	1.1	2	1.94	1.14	0.26	1.76	1.04	0.24	3.36
0.57	0.79	2.29	4.5	1.6	0.68	5.7	2.03	0.86	3.6
0.54	1.15	1.54	3.06	2.08	0.46	2.66	1.81	0.40	5.8
1.76	0.89	2.68	2.02	1.82	0.42	2.27	2.04	0.47	5.1
0.88	0.89	2.01	1.38	1.18	0.34	1.55	1.33	0.38	5.14
0.86	1.13	3.02	2.01	2.13	0.42	1.78	1.88	0.37	3.88
1.04	1.35	1.84	3.11	1.65	0.43	2.3	1.22	0.32	4.62
0.6	1.01	2.36	2.96	1.1	0.3	2.93	1.09	0.30	4.58
0.58	1.23	0.94	1.08	0.5	0.2	0.88	0.41	0.16	5.03
0.63	1.03	2.08	1.44	0.99	0.24	1.4	0.96	0.23	5.61
0.74	1.35	1.57	2.72	1.92	0.41	2.01	1.42	0.30	2.98
0.56	1.71	2.81	3.05	1.49	0.36	1.78	0.87	0.21	7.83
0.95	0.81	3.26	1.84	2.77	0.33	2.27	3.42	0.41	5.35
0.56	0.7	1.8	2.75	1.44	0.39	3.93	2.06	0.56	5.79
0.95	0.8	2.52	1.28	1.42	0.3	1.6	1.78	0.38	6.88
1.17	0.7	2.76	2.67	1.2	0.22	3.81	1.71	0.31	3.03
0.39	1.06	2.07	4.78	3.17	0.58	4.51	2.99	0.55	7.53
0.65	0.73	2.3	1.9	1.56	0.34	2.6	2.14	0.47	5.1
0.58	1.27	1.75	3.12	1.27	0.46	2.46	1.00	0.36	4.47
0.71	0.74	2.03	4.23	1.15	0.33	5.72	1.55	0.45	6.25
0.58	1.09	1.47	1.2	0.83	0.26	1.1	0.76	0.24	4.26
1.02	1.1	2.3	2.42	1.31	0.31	2.2	1.19	0.28	6.13
1.06	1.12	2.89	3.36	1.85	0.32	3	1.65	0.29	5.57
0.64	1.27	2.11	1.78	0.6	0.38	1.4	0.47	0.30	2.63
1.38	0.86	2.54	3.64	2.23	0.51	4.23	2.59	0.59	5.44
1.04	1.28	4.32	2.54	0.91	0.32	1.98	0.71	0.25	7.95
6.35	1.15	2.16	2.68	0.96	0.44	2.33	0.83	0.38	5.69
1.01	0.77	3.03	2.42	0.81	0.47	3.14	1.05	0.61	3.69
1.32	0.66	7.39	3.51	0.79	0.26	5.32	1.20	0.39	11.97
0.65	0.76	2.86	1.61	0.89	0.35	2.12	1.17	0.46	5.1
0.97	1.18	2.43	4.65	0.78	0.35	3.94	0.66	0.30	6.46
0.87	0.43	3.67	2.39	0.78	0.47	5.56	1.81	1.09	2.07
0.56	0.23	2.21	21.58	3.98	0.78	93.83	17.30	3.39	9.83
1.48	1.58	2.77	2.56	1.59	0.4	1.62	1.01	0.25	3.89
0.93	0.86	1.68	1.64	0.93	0.3	1.91	1.08	0.35	5.26
1.17	0.74	2.23	2.65	2.14	0.39	3.58	2.89	0.53	4.66
1.17	0.71	1.98	5.07	0.81	0.86	7.14	1.14	1.21	5.63

1.81	1.44	3.16	2.97	1.91	0.34	2.06	1.33	0.24	4.05
1.09	0.79	2.75	3.07	1.94	0.32	3.89	2.46	0.41	6.8
1.3	0.93	2.41	3.85	3.23	0.49	4.14	3.47	0.53	5.14
1	1.23	1.84	9.9	0.9	0.13	8.05	0.73	0.11	10.23
0.51	1.04	2.1	2.61	0.45	0.36	2.51	0.43	0.35	6.29
1.11	0.86	2.59	2.97	1	0.43	3.45	1.16	0.50	4.6
0.96	0.75	2.76	3.94	2.71	0.8	5.25	3.61	1.07	5.38
0.93	0.91	1.78	1.44	1.09	0.4	1.58	1.20	0.44	6.4
1.88	0.76	2.39	2.97	2.06	0.5	3.91	2.71	0.66	7.59
0.71	1.49	1.97	2.2	1.57	0.37	1.48	1.05	0.25	3.9
0.25	0.75	1.89	4.74	1.88	0.11	6.32	2.51	0.15	7.07
1.36	0.96	0.96	2.17	1.24	0.4	2.26	1.29	0.42	4.47
0.41	1.44	0.99	2	0.5	0.26	1.39	0.35	0.18	3.91
1.02	1.15	1.2	3.16	1.81	0.4	2.75	1.57	0.35	4.49
1.22	0.77	1.81	5.18	1.92	0.62	6.73	2.49	0.81	5.01
0.96	1.18	4	4.8	1.1	0.52	4.07	0.93	0.44	6.28
0.51	1.35	1.87	1.55	1.33	0.26	1.15	0.99	0.19	5.09
0.41	1.27	2.13	0.72	0.22	0.12	0.57	0.17	0.09	10.3
0.5	1.43	2.22	1.24	0.76	0.23	0.87	0.53	0.16	6.97
0.66	0.94	3.65	2.89	1.3	0.43	3.07	1.38	0.46	7.42
0.7	1.36	1.93	1	0.79	0.21	0.74	0.58	0.15	6.59
1.08	0.78	1.99	2.3	0.61	0.36	2.95	0.78	0.46	10.72
0.81	1.09	2.28	1.53	1.67	0.36	1.4	1.53	0.33	5.45
1.27	1.32	2.59	2.83	1.5	0.6	2.14	1.14	0.45	5.77
1.45	1.8	2.93	2.25	1.92	0.44	1.25	1.07	0.24	3.58
0.65	1.43	1.59	3.49	1.1	0.51	2.44	0.77	0.36	5.94
0.56	1.16	2.78	1.57	1.69	0.3	1.35	1.46	0.26	7.23
1.07	0.81	1.2	0.74	1.03	0.19	0.91	1.27	0.23	7.74
2.03	0.26	3.58	3.58	1.13	0.43	13.77	4.35	1.65	4.61
0.87	1.32	2.69	2.65	1.94	0.5	2.01	1.47	0.38	3.2
0.49	1.41	1.24	1.63	0.77	0.27	1.16	0.55	0.19	9.26
1.5	1.39	2.16	2.64	2.12	0.48	1.9	1.53	0.35	4.63
1.16	1.08	2.25	3.55	3.09	0.59	3.29	2.86	0.55	3.68
1.05	1.02	2.73	3.35	2.01	0.5	3.28	1.97	0.49	4.77
0.71	1.98	2.45	2	0.7	0.43	1.01	0.35	0.22	4.33
0.75	1.03	2.74	2.83	1.16	0.36	2.75	1.13	0.35	5.46
1.09	0.94	3.86	2.89	2.93	0.49	3.07	3.12	0.52	7.33
0.67	1.62	2.76	5.71	1.58	0.46	3.52	0.98	0.28	4.75
2.61	0.84	2.17	3.95	1.04	0.55	4.7	1.24	0.65	4.71
1.05	1.13	1.76	4.3	1.1	0.35	3.81	0.97	0.31	9.89
0.93	1.43	1.54	1.31	0.93	0.11	0.92	0.65	0.08	5.95
0.59	1.2	2.81	2	0.91	0.32	1.67	0.76	0.27	4.55
1.04	1.45	2.93	2.88	1.12	0.13	1.99	0.77	0.09	4.6
0.58	1.41	1.96	1.7	1.41	0.33	1.21	1.00	0.23	6.1

0.69	0.77	1.6	2.81	0.71	0.36	3.65	0.92	0.47	4.15
0.9	0.82	2.24	5.16	2.85	0.76	6.29	3.48	0.93	5.41
0.69	1.17	2.3	3.79	0.68	0.26	3.24	0.58	0.22	6.13
0.63	1.69	2.06	4.25	1.63	0.42	2.51	0.96	0.25	6.25
0.52	1.45	2.37	2.5	1	0.3	1.72	0.69	0.21	5.89
0.88	1.08	1.86	2.34	0.72	0.28	2.17	0.67	0.26	8.45
0.67	1.31	1.64	2.2	1.3	0.6	1.68	0.99	0.46	5.44
0.57	1.05	3.3	2.96	2.07	0.51	2.82	1.97	0.49	5.36
0.84	1.06	3.22	4.1	1.2	0.7	3.87	1.13	0.66	6.73
0.76	0.99	2.65	0.95	0.93	0.23	0.96	0.94	0.23	6.11
1.09	0.84	2.95	4.12	1.99	0.51	4.9	2.37	0.61	6.7
1.04	0.93	2.68	3.8	1.29	0.28	4.09	1.39	0.30	2.89
0.87	1.19	2.6	3.81	2.42	0.7	3.2	2.03	0.59	4.4
1.09	1.12	1.37	2.77	2.31	0.38	2.47	2.06	0.34	5.02
0.83	0.8	1.91	1.06	0.52	0.12	1.33	0.65	0.15	6.09
0.47	0.71	2.12	2.21	1.01	0.43	3.11	1.42	0.61	8.69
1.35	0.87	1.37	3.34	1.68	0.65	3.84	1.93	0.75	21.82
0.3	1.24	1.8	2.88	2.14	0.49	2.32	1.73	0.40	3.38
0.82	1	2.8	4.29	0.57	0.43	4.29	0.57	0.43	9.88
0.33	1.08	1.4	1.57	1.54	0.21	1.45	1.43	0.19	5.75
1.66	0.8	1.13	1.42	0.25	0.36	1.78	0.31	0.45	5.12
0.86	1	1.85	1.65	1.5	0.28	1.65	1.50	0.28	6.85
0.5	0.87	1.12	1.32	0.53	0.21	1.52	0.61	0.24	8.12
0.7	1.49	2.1	6.12	1.86	0.74	4.11	1.25	0.50	4.52
0.77	0.83	2.23	2.6	1.2	0.4	3.13	1.45	0.48	4.16
1.52	1.07	3.05	2.8	1.3	0.4	2.62	1.21	0.37	5.13
1.44	0.8	3.31	3.3	1.3	0.3	4.13	1.63	0.38	5.57
0.64	1.02	2.04	2.93	0.95	0.38	2.87	0.93	0.37	5.33
0.1	0.47	0.43	0.61	0.31	0.25	1.3	0.66	0.53	2.96
0.88	0.95	2.04	3.4	1.02	0.24	3.58	1.07	0.25	4.21
0.6	1.04	1.11	0.99	0.72	0.25	0.95	0.69	0.24	4.98
1.05	1.4	3.35	5.08	1.15	0.65	3.63	0.82	0.46	4.19
1.21	1.03	3.34	4.2	1.2	0.7	4.08	1.17	0.68	5.58
0.89	0.9	2.08	3	3.51	0.48	3.33	3.90	0.53	4.92
0.31	1.26	2.55	2.94	2.14	0.51	2.33	1.70	0.40	5.88
1.95	1.41	2.01	1.46	1.78	0.33	1.04	1.26	0.23	3.56
0.48	1.91	1.44	2.27	1.79	0.52	1.19	0.94	0.27	3.82
0.48	1.33	1.3	0.7	0.99	0.21	0.53	0.74	0.16	5.9
1.89	0.85	2.23	2.45	0.88	0.24	2.88	1.04	0.28	4.01
0.41	0.94	2.11	1.29	0.38	0.17	1.37	0.40	0.18	5.04
0.91	1.21	3.5	2.43	1.93	0.36	2.01	1.60	0.30	6.56
0.94	0.78	1.51	1.66	0.57	0.16	2.13	0.73	0.21	2.86
0.82	0.78	2.25	2.44	2.17	0.45	3.13	2.78	0.58	6.51
0.64	1.85	2.32	2.19	1.63	0.19	1.18	0.88	0.10	4.72

0.83	0.68	1.37	2.87	1.15	0.24	4.22	1.69	0.35	8.45
0.81	1.22	2.57	2.7	1.7	0.4	2.21	1.39	0.33	6.46
0.34	0.95	2.21	4.13	1.22	0.3	4.35	1.28	0.32	6.27
0.49	1.37	1.77	1.06	0.44	0.18	0.77	0.32	0.13	3.7
0.46	1.06	1.85	1.4	1.07	0.21	1.32	1.01	0.20	5.21
0.8	0.92	1.92	2.74	1.61	0.63	2.98	1.75	0.68	6.48
1.28	1.03	2.33	3.07	1.77	0.47	2.98	1.72	0.46	3.66
0.76	0.81	1.53	1	0.65	0.18	1.23	0.80	0.22	3.22
0.68	0.64	1.81	1.65	0.52	0.14	2.58	0.81	0.22	5.77
0.63	0.73	1.86	1.93	0.85	0.24	2.64	1.16	0.33	4.24
0.86	0.75	2.77	4.22	0.86	0.39	5.63	1.15	0.52	5.76
0.55	0.8	1.34	0.7	0.43	0.1	0.88	0.54	0.13	5.1
1.8	0.99	2.8	3.51	0.76	0.25	3.55	0.77	0.25	9.62
0.53	1.83	2.64	1.57	1.25	0.38	0.86	0.68	0.21	3.97
0.96	1.05	4.69	2.44	1	0.35	2.32	0.95	0.33	4.5
0.58	0.93	1.17	1.55	0.61	0.17	1.67	0.66	0.18	4.4
0.39	0.86	1.33	1.31	0.72	0.27	1.52	0.84	0.31	4.66
1.04	1.04	2.37	1.56	0.5	0.18	1.5	0.48	0.17	8.68
2.19	1.2	1.54	2.22	1.16	0.41	1.85	0.97	0.34	6.74
0.56	1.17	2.05	2.44	1.16	0.17	2.09	0.99	0.15	6.61
0.57	1.29	2.46	1.7	2.04	1.32	1.32	1.58	1.02	4.38
0.54	0.84	1.62	2.29	1.63	0.16	2.73	1.94	0.19	7.79
0.84	0.76	2.45	5.55	1.22	0.53	7.3	1.61	0.70	5.48
0.5	1.97	2.44	1.61	0.78	0.23	0.82	0.40	0.12	4.87
0.51	0.85	1.38	1.15	0.42	0.19	1.35	0.49	0.22	4.58
0.83	1.06	1.83	2.52	1.31	0.3	2.38	1.24	0.28	7.15
0.56	1.61	2.08	1.7	0.9	0.3	1.06	0.56	0.19	4.1
1.04	0.72	0.77	1.12	0.38	0.18	1.56	0.53	0.25	6.27
0.92	1.6	1.97	2.43	1.05	0.24	1.52	0.66	0.15	4.03
0.39	1.11	1.16	0.88	1.33	0.3	0.79	1.20	0.27	3.39
0.67	1.88	1.65	1.12	2.9	0.9	0.6	1.54	0.48	3.95
0.82	1.22	1.81	1.23	0.24	0.13	1.01	0.20	0.11	11.8
2.11	1.1	2.9	2.16	0.98	0.58	1.96	0.89	0.53	5.58
0.85	0.71	1.84	1.33	2.8	0.73	1.87	3.94	1.03	5.86
0.67	1.36	1.68	1.52	0.52	0.27	1.12	0.38	0.20	4.3
1.21	0.78	1.43	1.42	0.58	0.22	1.82	0.74	0.28	5.13
0.97	0.8	2.44	1.98	0.91	0.25	2.48	1.14	0.31	10.71
0.62	1.02	2.21	4.33	0.83	0.48	4.25	0.81	0.47	6.75
0.58	1.27	2.23	2.22	2.2	0.4	1.75	1.73	0.31	5.4
0.8	1.24	1.62	0.83	0.52	0.18	0.67	0.42	0.15	4.42
0.62	1.11	1.93	2.18	0.64	0.21	1.96	0.58	0.19	7.7
0.84	1.76	1.59	3.2	1	0.58	1.82	0.57	0.33	14.53
0.65	1.03	1.48	1.56	0.48	0.29	1.51	0.47	0.28	4.71
2.32	0.32	2.64	2.9	1.4	0.17	9.06	4.38	0.53	6.82

0.82	1.67	2.45	1.22	0.62	0.16	0.73	0.37	0.10	3.48
0.55	0.95	0.84	5.3	1.1	0.4	5.58	1.16	0.42	12.02
1.83	1.07	2.28	1.59	0.84	0.28	1.49	0.79	0.26	8.26
0.41	1.31	0.69	1.4	0.3	0.17	1.07	0.23	0.13	4.94
1.03	1.17	1.59	1.02	2.26	0.84	0.87	1.93	0.72	5.72
0.44	1.19	0.82	1.66	1.02	0.58	1.39	0.86	0.49	3.99
1.03	0.61	1.69	4.01	2.13	1.2	6.57	3.49	1.97	4.86
0.34	0.79	1.5	2.4	1.73	0.23	3.04	2.19	0.29	5.36
0.25	0.94	0.85	1.28	0.3	0.21	1.36	0.32	0.22	19.42
0.55	1.36	1.07	2.05	1.22	0.17	1.51	0.90	0.13	3.83
0.29	0.98	1.97	16.4	0.5	0.41	16.73	0.51	0.42	8.76
0.67	0.79	1.74	6.11	1.14	0.31	7.73	1.44	0.39	8.59
0.79	0.72	3.28	1.96	0.6	0.34	2.72	0.83	0.47	10.17
1.42	1.06	1.65	3.07	1.64	0.77	2.9	1.55	0.73	5.61
0.84	0.42	1.41	4.16	0.66	0.56	9.9	1.57	1.33	4.51
1.48	0.56	1.53	2.91	0.93	0.5	5.2	1.66	0.89	3.25
0.28	1.62	2.71	2.1	1.45	0.66	1.3	0.90	0.41	7.39
0.48	0.81	2.23	1.67	0.9	0.29	2.06	1.11	0.36	5.94
0.58	1.55	2.54	2.4	1.3	0.52	1.55	0.84	0.34	8.09
2.36	0.75	1.98	1.14	1.01	0.29	1.52	1.35	0.39	2.14
0.44	0.76	1.43	1.27	0.24	0.05	1.67	0.32	0.07	9.41
0.54	1.27	1.45	3.71	0.34	0.49	2.92	0.27	0.39	14.84
2	1.66	1.82	1.75	1.63	0.57	1.05	0.98	0.34	5.21
1.91	0.9	1.52	1.67	0.41	0.27	1.86	0.46	0.30	11.32
0.44	0.7	1.36	3.1	0.82	0.78	4.43	1.17	1.11	6.63
0.59	1.31	0.97	1.09	0.6	0.21	0.83	0.46	0.16	7.69
0.57	1.51	1.76	1.58	0.52	0.15	1.05	0.34	0.10	8.35
0.48	0.73	3.43	2.57	1.2	0.48	3.52	1.64	0.66	4.23
0.51	0.93	1.73	1.15	1.89	0.63	1.24	2.03	0.68	5.55
0.64	1.33	1.08	1.7	0.24	0.18	1.28	0.18	0.14	6.3
0.64	1.62	1.63	1.54	1.23	0.39	0.95	0.76	0.24	6.36
0.7	0.51	3.96	6.5	0.59	0.61	12.75	1.16	1.20	5.29
0.67	1.34	1.78	1.3	0.7	0.24	0.97	0.52	0.18	5.94
0.83	1.17	2.1	2.01	0.67	0.18	1.72	0.57	0.15	3.79
0.45	0.8	1.55	12.45	1.02	1.16	15.56	1.28	1.45	4.97
2	0.61	3.08	0.89	1.07	0.3	1.46	1.75	0.49	5.41
0.62	1.07	1.99	4.03	1.38	0.71	3.77	1.29	0.66	6.11
0.95	0.8	2.22	3.3	1	0.5	4.13	1.25	0.63	4.13
1.46	1.06	2.85	2.93	1.64	0.48	2.76	1.55	0.45	4.82
0.88	0.96	3.95	2.34	1.1	0.28	2.44	1.15	0.29	4.54
0.45	0.64	2.31	4.93	1.51	1.23	7.7	2.36	1.92	5.14
0.8	0.86	3.21	1.78	1.67	0.43	2.07	1.94	0.50	3.2
1.33	0.42	0.78	5.73	1.71	0.61	13.64	4.07	1.45	6.31
0.47	0.29	1.87	8.96	1.19	0.71	30.9	4.10	2.45	8.43

1.11	0.96	2.48	2.64	0.94	0.57	2.75	0.98	0.59	8.13
0.77	1.71	2.29	4.9	2.2	0.06	2.87	1.29	0.04	6.33
0.69	1.24	1.44	1.89	0.71	0.34	1.52	0.57	0.27	6.31
0.58	0.98	1.85	1.95	1.13	0.26	1.99	1.15	0.27	7.29
0.53	0.79	3.18	5.11	1.26	0.41	6.47	1.59	0.52	5.55
0.98	0.5	4.5	3.01	1.4	0.5	6.02	2.80	1.00	6.24
0.95	1.31	1.81	0.9	0.52	0.16	0.69	0.40	0.12	3.5
0.84	0.76	1.64	1.71	1.67	0.26	2.25	2.20	0.34	6.55
0.88	0.47	1.7	7.84	2.01	1.52	16.68	4.28	3.23	4.83
1.47	0.86	1.92	4.11	1.96	0.61	4.78	2.28	0.71	4.21
0.47	0.84	2.84	3.32	0.73	0.42	3.95	0.87	0.50	10.65
0.49	0.45	1.52	11.9	0.73	0.86	26.44	1.62	1.91	3.87
0.99	1.1	2.82	3.04	0.96	0.46	2.76	0.87	0.42	5.86
0.93	0.99	3.09	2.36	1.05	0.6	2.38	1.06	0.61	11.83
1.08	1.48	2.61	1.62	1.1	0.68	1.09	0.74	0.46	4.76
0.73	0.96	2.39	4.02	1.4	0.58	4.19	1.46	0.60	5.45
0.99	0.94	2.06	1.27	0.91	0.16	1.35	0.97	0.17	4.72
0.82	0.87	1.37	2.53	0.37	0.24	2.91	0.43	0.28	10.29
0.84	0.84	1.56	0.9	0.48	0.14	1.07	0.57	0.17	4.02
0.51	0.55	1.5	5.17	1.39	0.77	9.4	2.53	1.40	4.77
2.76	0.27	1.75	13.62	1.95	0.74	50.44	7.22	2.74	24.69
0.58	0.81	1.17	1.55	0.29	0.16	1.91	0.36	0.20	6.11
0.54	0.72	1.28	2.6	0.5	0.42	3.61	0.69	0.58	11
0.46	1.22	2.45	7.63	0.64	0.74	6.25	0.52	0.61	4.44
0.68	0.9	1.35	0.69	0.37	0.14	0.77	0.41	0.16	10.22
0.38	0.46	1.2	1.04	0.4	0.58	2.26	0.87	1.26	6.56
1.72	0.3	2.3	2.83	0.39	0.42	9.43	1.30	1.40	9.84
1.89	0.22	1.86	7.5	1.01	0.67	34.09	4.59	3.05	5.25
0.96	0.96	1.55	3.26	0.65	0.27	3.4	0.68	0.28	3.57
0.88	0.52	2.95	7.13	1.08	1	13.71	2.08	1.92	4.6
0.79	0.38	2.22	18.81	1.26	2.25	49.5	3.32	5.92	11.54
2.1	0.31	12.89	3.2	0.8	0.28	10.32	2.58	0.90	2.36
0.69	0.64	3.58	3.79	0.7	0.48	5.92	1.09	0.75	3.83
0.74	1.58	2.17	3.4	1	0.28	2.15	0.63	0.18	4.67
0.62	0.97	0.97	5.5	1.2	0.39	5.67	1.24	0.40	10.08
0.15	0.49	1.73	4.74	0.62	0.35	9.67	1.27	0.71	16.86
0.62	1.9	2.06	1.92	0.99	0.35	1.01	0.52	0.18	3.12
0.6	0.62	3.45	5.79	1.2	0.9	9.34	1.94	1.45	7.61
0.67	0.75	2.08	3.2	0.6	0.04	4.27	0.80	0.05	9.89
1.8	0.7	2.81	4.3	1.3	0.26	6.14	1.86	0.37	6.9
0.19	0.8	0.67	7	0.8	0.23	8.75	1.00	0.29	11.98
0.47	1.39	2.25	5.92	1.88	1.02	4.26	1.35	0.73	9.59
0.67	1.47	1.96	1.5	0.55	0.44	1.02	0.37	0.30	7.4
2.86	0.43	15.01	11.1	1.3	0.7	25.81	3.02	1.63	3.94

0.84	0.71	2.22	3.09	0.76	0.32	4.35	1.07	0.45	4.82
0.55	1.47	1.42	3.94	1.03	0.47	2.68	0.70	0.32	8.01
0.89	0.46	2.91	4.3	1.5	0.42	9.35	3.26	0.91	6.2
0.95	1.39	1.5	2.1	0.3	0.29	1.51	0.22	0.21	4.55
0.44	0.39	1.3	1.3	0.3	0.17	3.33	0.77	0.44	7.89
0.69	0.68	1.04	1.66	0.15	0.13	2.44	0.22	0.19	9.5
1.56	0.7	1.52	2.88	1.28	0.43	4.11	1.83	0.61	11.39
1.13	1.47	0.94	1.2	1.2	0.45	0.82	0.82	0.31	11.33
1.05	0.64	3.42	3.49	0.69	0.24	5.45	1.08	0.38	3.47
0.58	0.54	2.01	2.91	1.31	0.43	5.39	2.43	0.80	5.56
0.79	0.46	1.04	2.6	0.3	0.21	5.65	0.65	0.46	8.9
0.45	0.47	2.49	3.72	1.04	0.65	7.91	2.21	1.38	9.7
0.36	1.89	2.13	2.49	0.71	0.49	1.32	0.38	0.26	10.67
0.72	0.49	1.49	4.75	0.51	0.28	9.69	1.04	0.57	7.81
0.59	0.29	2.12	7.76	0.45	1.67	26.76	1.55	5.76	14.05
0.86	1.28	1.13	3.34	0.65	0.53	2.61	0.51	0.41	9.01
0.93	0.63	2.57	6.63	0.62	0.61	10.52	0.98	0.97	5.63
0.81	0.49	2.19	4.03	1.58	0.69	8.22	3.22	1.41	3.94
0.71	1.16	1.46	1.36	0.69	0.22	1.17	0.59	0.19	5.68
0.86	0.12	1.79	7	0.5	0.33	58.33	4.17	2.75	30.3
0.65	1.75	1.89	1.6	0.61	0.22	0.91	0.35	0.13	2.83
0.59	1.1	1.69	2.5	1.4	0.27	2.27	1.27	0.25	10.62
1.37	0.54	1.52	6	0.8	0.12	11.11	1.48	0.22	13.85
0.87	1.24	1.84	2.47	0.98	0.44	1.99	0.79	0.35	4.88
0.54	1.29	1.52	4.13	1.42	0.53	3.2	1.10	0.41	5.5
0.55	1.53	1.02	3.61	0.55	0.3	2.36	0.36	0.20	3.91
0.77	0.82	1.84	2.08	1.03	0.45	2.54	1.26	0.55	3.93
0.93	0.74	0.99	1.54	0.31	0.15	2.08	0.42	0.20	8.58
1.1	0.23	3.57	3.8	0.4	0.37	16.52	1.74	1.61	6.65
0.59	0.58	1.18	1.6	0.7	0.42	2.76	1.21	0.72	5.01
0.86	1.15	2.88	1.23	0.45	0.16	1.07	0.39	0.14	6.72
1.67	0.2	5.32	3.96	1.13	0.59	19.8	5.65	2.95	2
1.25	1	2.57	4.88	1.05	0.54	4.88	1.05	0.54	6.13
0.86	1.06	1.62	3.5	0.7	0.21	3.3	0.66	0.20	5.63
0.64	0.88	1.13	2.1	0.91	0.41	2.39	1.03	0.47	4.16
0.89	1.31	3.1	3.63	0.61	0.26	2.77	0.47	0.20	6.27
0.79	0.96	2.76	3.21	0.8	0.38	3.34	0.83	0.40	4.71
0.46	0.64	1.13	1.43	0.68	0.23	2.23	1.06	0.36	1.5
1.13	0.71	3.17	3.21	1.13	0.35	4.52	1.59	0.49	3.94
1.49	0.28	1.48	5.3	2	98	18.93	7.14	350.00	6.9
0.55	1.47	1.11	1.48	0.84	0.34	1.01	0.57	0.23	14.22
0.41	0.85	1.87	1.5	1.4	0.29	1.76	1.65	0.34	3.37
0.33	1.26	1.82	1.62	0.38	0.41	1.29	0.30	0.33	5.69
0.62	0.43	1.22	2.15	0.52	0.35	5	1.21	0.81	7.94

1.53	1.54	1.49	4.44	1.4	0.43	2.88	0.91	0.28	3.98
1.64	1.16	4.46	3.9	1.8	0.8	3.36	1.55	0.69	6.49
3.44	0.49	3.44	7.8	1.7	0.17	15.92	3.47	0.35	7.01
1.19	0.27	2.67	4.1	1.1	0.32	15.19	4.07	1.19	7.51
2.57	0.39	2.57	19.8	2.1	0.28	50.77	5.38	0.72	16.86
0.96	0.91	2.57	4.5	0.8	0.19	4.95	0.88	0.21	4.83
0.61	0.71	1.4	3.82	2.99	1.14	5.38	4.21	1.61	10.34
0.46	0.3	0.5	8.1	1.3	0.16	27	4.33	0.53	5.71
1.07	1.02	1.53	2.12	1.29	0.36	2.08	1.26	0.35	3.71
0.78	1.32	2.53	1.48	0.91	0.19	1.12	0.69	0.14	3.73
1.02	0.72	1.82	7.2	1.3	0.72	10	1.81	1.00	10.14
0.52	0.89	1.33	15.6	1.3	0.45	17.53	1.46	0.51	5.43
1.1	0.71	1.45	3.2	1	0.45	4.51	1.41	0.63	6.27
0.37	0.83	0.76	1.2	0.6	0.29	1.45	0.72	0.35	3.41
0.72	1.84	1.82	2.9	0.8	0.11	1.58	0.43	0.06	6.1
0.79	0.63	2.63	4.6	1.6	0.3	7.3	2.54	0.48	3.65
1.36	0.48	1.23	3.96	2.3	0.5	8.25	4.79	1.04	4.81
0.45	1.38	2.65	4.4	0.3	0.37	3.19	0.22	0.27	6.94
1.92	0.33	2.16	4.77	2	0.52	14.45	6.06	1.58	5.43
1.05	1	2.08	2.62	1.11	0.18	2.62	1.11	0.18	7.08
0.84	0.99	1.69	1.12	0.76	0.19	1.13	0.77	0.19	4.09
0.99	1.13	1.7	1.9	1.54	0.38	1.68	1.36	0.34	8.05
0.12	0.26	0.49	5.14	0.24	0.54	19.77	0.92	2.08	22.32
0.48	1.58	1.7	3.42	1.86	0.6	2.16	1.18	0.38	3.5
1.15	0.3	1.32	2.47	1.38	0.47	8.23	4.60	1.57	4.56
0.77	1.17	2.1	2.74	0.5	0.38	2.34	0.43	0.32	4.84
1.67	0.55	2.05	7.9	0.5	0.36	14.36	0.91	0.65	4.01
1.06	0.75	0.92	4.7	1.2	0.34	6.27	1.60	0.45	10.02
0.62	1.43	1.21	2	0.93	0.48	1.4	0.65	0.34	9.98
2.39	0.14	2.19	6.8	0.5	0.17	48.57	3.57	1.21	5.36
0.39	1.13	1.15	1.3	0.7	0.74	1.15	0.62	0.65	4.54
0.53	1.01	1.91	4.59	0.52	0.6	4.54	0.51	0.59	11.19
0.81	0.44	0.99	3.5	0.6	0.31	7.95	1.36	0.70	14.03
1.09	1.54	1.08	2.17	0.77	0.38	1.41	0.50	0.25	3.43
1.73	0.36	2.29	5.7	1.2	0.3	15.83	3.33	0.83	7.14
0.67	0.71	2.56	5.2	1.5	0.28	7.32	2.11	0.39	7.26
0.36	0.54	1.07	2.89	0.16	0.25	5.35	0.30	0.46	8.76
0.8	1.08	1.38	2	1.1	0.38	1.85	1.02	0.35	6.37
0.77	0.5	2.22	3.7	0.5	0.44	7.4	1.00	0.88	5.91
0.74	1.38	2.02	4.3	0.7	0.69	3.12	0.51	0.50	5.32
0.5	1.83	1.43	2.21	0.97	0.26	1.21	0.53	0.14	5.19
0.78	0.81	1.66	2.3	0.3	0.41	2.84	0.37	0.51	2.15
1.3	0.6	3.08	6.1	1.6	0.27	10.17	2.67	0.45	7.83
0.97	0.9	1.37	1.78	0.75	0.22	1.98	0.83	0.24	6.76

0.47	0.85	1.36	8.5	0.4	0.17	10	0.47	0.20	5.93
0.77	1.23	1.58	0.9	0.75	0.15	0.73	0.61	0.12	4.02
0.43	1.21	2.21	6.3	0.8	0.21	5.21	0.66	0.17	4.75
0.34	1.13	1.67	1.49	0.55	0.25	1.32	0.49	0.22	5.56
0.6	0.13	0.69	11.37	1	0.49	87.46	7.69	3.77	23.82
0.94	1.08	3.02	4.9	1.9	0.49	4.54	1.76	0.45	4.75
0.7	0.86	2.54	3.82	1.38	0.56	4.44	1.60	0.65	7.28
0.41	1.44	0.99	2	0.5	0.89	1.39	0.35	0.62	3.91
0.77	0.61	1.56	5.4	0.5	0.36	8.85	0.82	0.59	6.33
0.8	1	2.86	2.15	1.26	0.52	2.15	1.26	0.52	7.78
1.09	0.88	2.82	1.55	0.65	0.24	1.76	0.74	0.27	5.69
0.44	1.6	1.57	2.9	0.72	0.69	1.81	0.45	0.43	16.86
0.96	1.72	1.4	2.1	0.5	0.41	1.22	0.29	0.24	4.02
1.9	0.6	3.23	4.04	2.09	0.38	6.73	3.48	0.63	4.01
0.39	0.85	2.15	6.1	1.2	0.69	7.18	1.41	0.81	7.55
1.03	0.44	3.01	17.64	0.95	0.98	40.09	2.16	2.23	13.82
1.08	0.22	0.95	2.6	0.6	0.24	11.82	2.73	1.09	4.3
1.28	0.39	2	6.54	0.72	0.55	16.77	1.85	1.41	4.69
1.34	0.37	2.26	4.1	1.1	0.25	11.08	2.97	0.68	6.7
1.03	0.78	3.22	6.4	0.8	0.17	8.21	1.03	0.22	5.67
0.44	1.44	1.91	3.61	0.73	0.4	2.51	0.51	0.28	8.46
0.71	1.2	1.68	4.8	0.5	0.5	4	0.42	0.42	4.87
0.63	0.12	0.34	7.64	0.84	0.59	63.67	7.00	4.92	6.39
1.5	0.24	2.34	3.9	0.9	0.21	16.25	3.75	0.88	5.66
0.84	0.62	1.7	4.6	1.41	0.62	7.42	2.27	1.00	6.41
1.1	1.24	1.8	2.39	0.79	0.33	1.93	0.64	0.27	4.82
1.33	0.91	3.01	4.7	1.2	0.31	5.16	1.32	0.34	4.03
2.22	1.14	3.29	4.8	0.8	0.34	4.21	0.70	0.30	3.59
1.33	0.42	2.05	4.3	0.21	0.28	10.24	0.50	0.67	10.32
0.71	1.21	2.2	3.9	1.3	0.36	3.22	1.07	0.30	7.17
0.56	0.92	1.6	1.8	0.8	0.45	1.96	0.87	0.49	2.79
0.81	0.95	0.9	5.6	1.2	0.29	5.89	1.26	0.31	5.67
0.38	0.28	0.85	1.26	1.66	0.09	4.5	5.93	0.32	5.23
0.69	0.24	1.05	7.8	0.4	0.21	32.5	1.67	0.88	6.06
1.14	0.43	2.89	5.2	0.7	0.27	12.09	1.63	0.63	4.67
0.93	1.22	2.17	1.62	1.64	0.45	1.33	1.34	0.37	10.49
0.66	1.38	1.52	1.31	0.59	0.22	0.95	0.43	0.16	6.63
1.41	1.49	3.16	6.5	0.3	0.42	4.36	0.20	0.28	23.46
1.2	0.96	2.5	6.02	0.64	0.45	6.27	0.67	0.47	14.44
1.31	0.37	3.88	3.5	0.7	1.01	9.46	1.89	2.73	2.39
1.59	0.78	1.81	5.7	0.6	1.47	7.31	0.77	1.88	5.46
0.68	1.02	1.78	4.54	0.7	0.42	4.45	0.69	0.41	16.73
1.03	0.13	0.7	10.03	1.24	1.61	77.15	9.54	12.38	5.76
0.86	0.39	2.07	7.34	0.69	0.67	18.82	1.77	1.72	4.77

0.66	1.16	1.29	0.75	0.6	0.19	0.65	0.52	0.16	4.09
0.85	0.2	0.86	7.9	1.3	0.37	39.5	6.50	1.85	3.71
0.67	0.6	1.96	3.61	1.85	0.61	6.02	3.08	1.02	3.14
2.7	0.17	1.42	4.17	1.27	0.68	24.53	7.47	4.00	5.2
0.49	1.14	1.53	4.33	0.93	0.79	3.8	0.82	0.69	7.33
4.98	0.46	1.07	2	0.2	0.2	4.35	0.43	0.43	12.8
0.71	1.39	1.68	2.3	1.8	0.27	1.65	1.29	0.19	5.24
2.28	0.87	1.06	1.57	0.61	0.25	1.8	0.70	0.29	2.99
1.36	0.17	2.17	3.9	0.6	0.22	22.94	3.53	1.29	4.93
0.68	0.63	2.08	2	0.8	0.21	3.17	1.27	0.33	4.18
0.5	1.12	2.15	1	0.5	0.42	0.89	0.45	0.38	5.55
0.78	1.15	1.51	1.51	0.71	0.31	1.31	0.62	0.27	9.31
0.97	0.54	2.03	2.6	0.9	0.52	4.81	1.67	0.96	4.93
0.67	1.19	2.65	1.9	2.9	0.85	1.6	2.44	0.71	3.06
1.63	0.63	4.13	5.3	0.8	0.4	8.41	1.27	0.63	8.04
0.5	0.65	1.02	7.1	2.12	0.86	10.92	3.26	1.32	10.81
0.75	0.27	1.47	11.5	1.8	0.4	42.59	6.67	1.48	4.72
0.27	0.79	0.42	1.6	0.3	0.32	2.03	0.38	0.41	8.03
0.68	0.65	2.16	10.25	2.37	1.42	15.77	3.65	2.18	10.04
0.75	0.69	2.92	0.76	0.38	0.19	1.1	0.55	0.28	4.92
0.61	0.36	1.39	2.06	1.07	0.45	5.72	2.97	1.25	10.13
0.46	0.11	1.01	6.8	0.5	0.31	61.82	4.55	2.82	10.29
0.76	1.13	2.09	1.57	0.81	0.28	1.39	0.72	0.25	3.17
0.52	0.78	1.15	2.3	1	0.25	2.95	1.28	0.32	7.66
1.01	1.77	1.53	9.81	0.92	0.56	5.54	0.52	0.32	13.71
1.04	1.26	3.13	1.61	0.58	0.25	1.28	0.46	0.20	4.2
1.18	0.5	2.56	1.96	0.8	0.23	3.92	1.60	0.46	8.63
0.8	1	2.32	5.59	1.51	0.74	5.59	1.51	0.74	5.5
1.03	0.73	1	2.33	0.8	0.29	3.19	1.10	0.40	8.99
0.6	0.11	1.3	7.09	0.52	0.73	64.45	4.73	6.64	21.73
0.94	1.05	1.82	1.77	0.92	0.35	1.69	0.88	0.33	4.02
0.65	0.93	1.68	1.32	0.52	0.28	1.42	0.56	0.30	8.4
0.89	1.01	2.33	6.97	1.42	0.66	6.9	1.41	0.65	6.38
0.94	0.57	2.25	2.33	1.66	0.74	4.09	2.91	1.30	5.19
1.12	0.39	2.46	9.04	1.17	0.95	23.18	3.00	2.44	3.66
0.73	1.43	1.2	1.7	0.4	0.45	1.19	0.28	0.31	4.4
5.42	0.22	4.48	7.22	0.9	0.83	32.82	4.09	3.77	4.18
0.99	0.35	2.43	1.17	0.84	0.4	3.34	2.40	1.14	4.29
1.11	0.81	3.77	2.43	0.71	0.23	3	0.88	0.28	6.17
0.92	0.64	1.82	2.74	1.01	0.54	4.28	1.58	0.84	6.12
1.87	0.18	2.35	2.69	0.74	0.4	14.94	4.11	2.22	4.36
1.12	1.15	2.72	1.64	1.31	0.41	1.43	1.14	0.36	6.69
0.91	0.82	1.88	12.45	0.49	1.24	15.18	0.60	1.51	12.8
0.4	0.66	1.42	0.65	2.44	0.89	0.98	3.70	1.35	2.29

0.87	0.42	1.94	3.11	0.82	0.49	7.4	1.95	1.17	4.48
1	0.61	2.37	5.47	1	0.9	8.97	1.64	1.48	5.54
0.4	1.36	1.55	1.05	0.85	0.41	0.77	0.63	0.30	5.84
2.04	0.96	2.04	2.05	1.59	0.32	2.14	1.66	0.33	3.4
0.98	0.25	1.53	5.22	1.63	1.04	20.88	6.52	4.16	3.71
1.44	0.59	1.65	0.82	0.83	0.23	1.39	1.41	0.39	4.96
0.54	0.13	0.9	8.9	1.68	0.69	68.46	12.92	5.31	7.68
1.14	0.42	1.84	14.61	1.39	0.8	34.79	3.31	1.90	13.5
1.85	0.24	1.45	15.61	0.98	0.93	65.04	4.08	3.88	24.91
0.47	1.08	2.35	1.1	0.64	0.25	1.02	0.59	0.23	5.24
1.27	0.29	3.42	4.72	0.58	0.28	16.28	2.00	0.97	9.17
0.95	0.65	3.29	2.99	1.72	0.59	4.6	2.65	0.91	2.29
0.83	1.09	1.76	2.1	1.77	0.53	1.93	1.62	0.49	4.93
0.39	0.4	0.92	12.19	0.92	1.46	30.48	2.30	3.65	4.61
0.67	1.17	1.75	3.5	0.56	0.28	2.99	0.48	0.24	2.45
0.49	0.97	2.68	2.27	1.39	0.32	2.34	1.43	0.33	6.95
0.59	0.61	2.45	5.27	0.74	0.51	8.64	1.21	0.84	6.87
3.83	0.28	3.9	3.15	1.55	0.59	11.25	5.54	2.11	2.13
0.46	0.09	1.04	2.91	0.95	0.85	32.33	10.56	9.44	15.6
0.55	0.91	1.54	1.2	0.58	0.19	1.32	0.64	0.21	5.91
1.1	0.16	1.77	4.77	1.03	0.41	29.81	6.44	2.56	3.94
0.47	1.21	1.31	3.84	1.08	0.32	3.17	0.89	0.26	3.63
0.72	1.47	2.96	2.26	1.25	0.43	1.54	0.85	0.29	4.04
0.82	0.71	4.75	6.25	1.29	0.73	8.8	1.82	1.03	4.67
0.24	0.94	0.62	1.11	0.37	0.07	1.18	0.39	0.07	3.19
0.67	0.84	1.88	5.63	0.92	0.69	6.7	1.10	0.82	5.02
0.32	0.46	0.95	5.78	0.26	0.35	12.57	0.57	0.76	4.82
0.58	0.99	2.12	3.04	0.43	0.56	3.07	0.43	0.57	5.79
0.41	0.85	1.44	3.55	0.38	0.21	4.18	0.45	0.25	8.24
0.58	1.08	1.43	1.34	0.47	0.22	1.24	0.44	0.20	5.38
1.66	0.26	1.59	1.96	1.13	0.43	7.54	4.35	1.65	5.93
1.48	0.96	1.83	2.09	0.78	0.22	2.18	0.81	0.23	3.5
0.7	0.66	2.46	6.02	1.15	0.78	9.12	1.74	1.18	3.12
1.27	0.88	0.76	5.38	1.44	0.52	6.11	1.64	0.59	4.33
0.46	0.77	1.21	1.09	0.51	0.2	1.42	0.66	0.26	6.1
0.97	0.13	1.64	14.91	1.8	1.07	84.69	13.85	8.23	6.07
0.59	0.97	2	2.58	1.6	0.38	2.66	1.65	0.39	5.57
0.7	0.33	1.35	2.91	1.04	0.27	8.82	3.15	0.82	4.8
1.94	0.8	5.7	3.22	0.67	0.56	4.03	0.84	0.70	6.62
0.42	0.11	0.86	3.18	1.12	0.34	28.91	10.18	3.09	7.07
0.38	1.51	2.58	1.25	0.68	0.25	0.83	0.45	0.17	5.24
0.9	1.17	1.85	1.35	0.72	0.23	1.15	0.62	0.20	4.3
1.2	0.7	2.53	11.88	1.24	0.86	16.97	1.77	1.23	4.35
0.4	0.85	0.87	3.25	0.83	0.24	3.82	0.98	0.28	7.67

1.18	0.49	3.6	11.29	1.35	0.11	23.04	2.76	0.22	19.69
0.59	0.47	1.7	5.61	0.96	0.6	11.94	2.04	1.28	4.89
2.58	0.17	2.66	8.83	0.24	0.28	51.94	1.41	1.65	10.39
1.12	0.36	1.08	1.71	0.59	0.57	4.75	1.64	1.58	7.46
0.66	0.45	1.06	1.93	0.61	0.31	4.29	1.36	0.69	6.57
0.7	0.92	2.58	6.78	0.71	0.8	7.37	0.77	0.87	6.15
0.73	1.49	2.24	3.91	1.45	0.72	2.62	0.97	0.48	11.95
0.64	0.93	2.17	3.25	1.33	0.95	3.49	1.43	1.02	7.01
0.48	1.08	1.69	2.16	0.63	0.35	2	0.58	0.32	4.65
0.49	0.41	1.23	3.94	0.83	0.63	9.61	2.02	1.54	2.7
0.92	1.93	2.25	2.29	1.65	0.28	1.19	0.85	0.15	6.62
0.61	1.2	2.2	6.37	1.05	0.62	5.31	0.88	0.52	7.02
0.78	1.01	1.22	1.23	0.58	0.28	1.22	0.57	0.28	2.02
0.83	0.81	2.31	2.94	1.75	0.34	3.63	2.16	0.42	4.62
0.85	0.46	2.64	5.61	1.29	0.63	12.2	2.80	1.37	3.17
0.51	0.89	1.05	5.22	1.18	0.76	5.87	1.33	0.85	7.88
0.34	0.53	1.18	1.84	0.68	0.21	3.47	1.28	0.40	4.1
0.72	0.59	2.7	4.43	0.98	0.51	7.51	1.66	0.86	6.82
0.43	0.62	1.74	2.89	0.81	0.02	4.66	1.31	0.03	23.73
0.52	1.41	2.09	4.6	0.11	0.31	3.26	0.08	0.22	30.38
1.3	0.49	1.73	5.21	1.79	0.34	10.63	3.65	0.69	3.95
0.62	0.98	0.9	4.53	0.71	0.45	4.62	0.72	0.46	13.36
0.59	0.51	0.73	1.44	0.59	0.41	2.82	1.16	0.80	4.31
1.52	0.71	1.5	2.56	0.85	0.29	3.61	1.20	0.41	5.23
0.92	1.51	2.19	2.26	1.03	0.43	1.5	0.68	0.28	8.31
0.68	0.59	3.12	2.9	1.37	0.71	4.92	2.32	1.20	15.04
0.26	0.21	1.7	1.98	0.31	0.17	9.43	1.48	0.81	33.52
0.99	0.61	1.28	1.35	0.57	0.22	2.21	0.93	0.36	24.13
0.72	1	2.76	2.8	0.75	0.21	2.8	0.75	0.21	5.56
0.36	0.13	0.44	8.61	0.31	0.51	66.23	2.38	3.92	3.7
0.55	0.88	2.93	3.2	0.91	0.62	3.64	1.03	0.70	3.32
0.58	1.3	2	1.54	0.54	0.23	1.18	0.42	0.18	4.53
1.1	0.1	1.91	25.91	2.67	2.06	79.1	26.70	20.60	27.21
1.78	0.39	2.04	3.37	1.15	0.54	8.64	2.95	1.38	9.78
0.51	0.58	1	3.14	0.29	0.12	5.41	0.50	0.21	12.44
2.3	1.21	1.21	2.69	0.44	0.48	2.22	0.36	0.40	4.33
1.05	1.16	2.24	2.2	1.7	0.28	1.9	1.47	0.24	4.57
0.55	1.38	1.37	2.67	1.6	0.5	1.93	1.16	0.36	7.05
1.1	1.19	3.01	5.87	1.42	0.53	4.93	1.19	0.45	5.89
0.99	0.65	3.53	1.9	1.06	0.43	2.92	1.63	0.66	6.99
0.64	1.81	2.02	0.77	0.56	0.2	0.43	0.31	0.11	5.41
0.71	0.24	1.03	1.95	0.37	0.39	8.13	1.54	1.63	10.35
1.42	0.66	1.92	4.78	0.51	0.3	7.24	0.77	0.45	31.55
1.31	0.08	1	16.85	4.87	1.37	90.63	60.88	17.13	16.95

1.05	0.92	1.59	3.27	2.13	0.65	3.55	2.32	0.71	15.97
2.91	0.19	4.52	3.92	1.97	0.72	20.63	10.37	3.79	5.93
0.56	1.02	1.9	3.03	1.89	0.4	2.97	1.85	0.39	4.51
0.66	1.1	1.45	2.77	1.11	0.38	2.52	1.01	0.35	6.33
0.61	1	2.28	3.43	0.57	0.19	3.43	0.57	0.19	5.2
0.83	0.46	1.11	4.55	1.39	0.56	9.89	3.02	1.22	8.73
0.89	0.33	2.64	7.4	0.87	0.34	22.42	2.64	1.03	9.07
0.79	0.74	1.84	2.17	1.34	0.37	2.93	1.81	0.50	6.45
0.33	1.17	1.83	2.55	1.13	0.38	2.18	0.97	0.32	4.86
1.76	0.66	0.91	1.14	1.2	0.21	1.73	1.82	0.32	5.7
0.64	1.04	1.05	6.43	0.45	0.41	6.18	0.43	0.39	3.79
0.24	0.18	0.4	3.56	1	0.47	19.78	5.56	2.61	10.05
1.77	0.27	2.16	7.62	1.56	0.82	28.22	5.78	3.04	15.13
0.21	1.2	1.3	2.96	1.21	0.34	2.47	1.01	0.28	11.02
0.91	0.98	2.52	3.1	1.38	0.41	3.16	1.41	0.42	5.32
0.88	0.4	2.65	5.05	0.73	0.49	12.63	1.83	1.23	8.97
0.45	0.52	1.53	2.02	2.69	0.57	3.88	5.17	1.10	3.38
1.25	1.33	1.86	1.56	2.64	0.35	1.17	1.98	0.26	3.69
1.38	1.11	2.36	2.16	1.16	0.37	1.95	1.05	0.33	4.6
0.78	1.28	1.63	3.87	1.28	0.46	3.02	1.00	0.36	5.72
0.95	0.57	2.77	2.23	1.06	0.33	3.91	1.86	0.58	3.99
0.44	0.52	1.63	1.4	0.31	0.38	2.69	0.60	0.73	3.56
0.41	0.09	0.22	11.5	0.47	0.37	87.78	5.22	4.11	15.97
0.45	0.77	1.33	5.71	1.46	1.05	7.42	1.90	1.36	7.92
1.22	0.67	2.05	5.1	0.98	0.34	7.61	1.46	0.51	6.25
0.67	1.04	2.15	2.45	1.31	0.4	2.36	1.26	0.38	7.95
0.48	1.31	1.24	4.43	0.77	0.27	3.38	0.59	0.21	5.13
1.59	1.47	5.01	4.34	1.27	0.74	2.95	0.86	0.50	5.42
0.71	1.09	2.27	2	0.61	0.26	1.83	0.56	0.24	7.4
0.79	0.88	1.68	2.55	0.91	0.58	2.9	1.03	0.66	4.94
0.61	0.32	2.33	2.57	0.71	0.41	8.03	2.22	1.28	2.81
0.33	1.08	1.76	2.77	0.99	0.75	2.56	0.92	0.69	15.83
0.88	0.91	1.33	2.03	0.99	0.27	2.23	1.09	0.30	7.1
0.72	1.07	2.52	2.44	1.85	0.24	2.28	1.73	0.22	4.26
0.83	0.8	1.36	4.59	0.42	0.34	5.74	0.53	0.43	9.8
1.44	0.69	3.28	4.37	0.64	0.28	6.33	0.93	0.41	8.14
1.61	0.71	1.47	3.78	1.25	0.46	5.32	1.76	0.65	4.45
0.59	0.9	0.84	2.1	0.79	0.37	2.33	0.88	0.41	4.43
0.62	0.73	2.15	3.35	0.8	0.37	4.59	1.10	0.51	8.17
0.43	0.96	0.94	3.97	0.32	0.19	4.14	0.33	0.20	27.7
0.68	0.53	0.86	1.6	0.86	0.2	3.02	1.62	0.38	4.5
0.6	0.6	1.33	3.14	1.25	0.61	5.23	2.08	1.02	6.36
0.47	0.46	1.77	1.79	0.72	0.13	3.89	1.57	0.28	6.19
1.11	1.5	3.22	2.22	2.03	0.38	1.48	1.35	0.25	4.98

0.84	1.27	2.08	1.16	0.58	0.14	0.91	0.46	0.11	4.93
2.1	0.64	2.08	1.59	0.84	0.15	2.48	1.31	0.23	4.01
1.23	0.7	1.77	3.46	2.3	0.47	4.94	3.29	0.67	6.75
0.67	0.29	1.56	2.41	0.81	0.4	8.31	2.79	1.38	3.2
0.75	0.52	1.03	3.59	0.66	0.36	6.9	1.27	0.69	3.16
1.42	0.66	2.16	4.99	0.82	0.15	7.56	1.24	0.23	4.34
0.44	0.65	1.25	1.68	0.66	0.28	2.58	1.02	0.43	3.77
0.97	0.9	1.33	1.11	0.27	0.09	1.23	0.30	0.10	3.8
0.5	0.49	1.1	3.79	1.04	0.29	7.73	2.12	0.59	3.87
0.58	1.04	1.43	3.04	2.5	0.83	2.92	2.40	0.80	8.2
0.75	0.81	1.98	1.75	1.56	0.49	2.16	1.93	0.60	7.35
0.59	0.42	0.84	1.34	0.19	0.24	3.19	0.45	0.57	26.6
0.39	0.13	0.29	14.82	1.75	0.66	74	13.46	5.08	23.33
1.38	1.24	1.74	2.39	1.56	0.36	1.93	1.26	0.29	5.15
1.3	0.43	3.08	1.97	0.78	0.36	4.58	1.81	0.84	3.94
0.31	1.09	2.1	3.67	2.38	0.72	3.37	2.18	0.66	4.51
0.79	1.21	1.7	2.68	1.44	0.48	2.21	1.19	0.40	4.68
0.15	0.14	0.56	7.19	0.55	0.29	51.36	3.93	2.07	13.42
0.67	0.87	1.8	1.74	1.21	0.33	2	1.39	0.38	5.59
0.46	0.47	1.26	2.34	0.47	0.09	4.98	1.00	0.19	3.76
0.58	0.62	1.69	1.12	0.63	0.56	1.81	1.02	0.90	22.38
1.28	0.26	3.72	6.89	0.94	0.47	26.5	3.62	1.81	3.39
0.83	1.05	1.86	5.36	0.83	0.59	5.1	0.79	0.56	29.46
0.51	1.02	1.7	4.2	1.54	0.54	4.12	1.51	0.53	3.79
1.13	0.39	1.55	5.06	0.54	0.32	12.97	1.38	0.82	5.36
0.54	0.9	1.53	1.84	1.63	0.32	2.04	1.81	0.36	9.97
0.62	2.03	1.42	2.21	1.25	0.33	1.09	0.62	0.16	6.35
1.08	1.27	2.51	2.71	2.01	0.53	2.13	1.58	0.42	5.23
1.08	0.36	3.14	3.45	0.52	0.41	9.58	1.44	1.14	9.69
0.79	1.17	3.47	6.73	1.51	0.93	5.75	1.29	0.79	5.34
2.16	0.37	2.85	4.9	1.56	1.24	13.24	4.22	3.35	2.89
1.11	0.14	1.44	3.2	0.93	0.47	22.86	6.64	3.36	5.97
0.63	0.9	1.51	1.96	1.09	0.51	2.18	1.21	0.57	5.61
0.35	1.22	1.77	6.08	0.53	0.5	4.98	0.43	0.41	3.79
1.48	0.25	1.87	2.2	0.89	0.29	8.8	3.56	1.16	4.59
1.23	1.01	3.38	4.17	1.87	0.46	4.13	1.85	0.46	5.56
0.73	0.64	1.8	1.75	0.31	0.2	2.73	0.48	0.31	3.74
0.88	0.73	2.76	3.21	0.58	0.34	4.4	0.79	0.47	6.28
0.76	1.31	3.27	2.44	1.99	0.45	1.86	1.52	0.34	4.63
1.1	0.74	2.39	3	0.99	0.35	4.05	1.34	0.47	8.16
0.12	1.21	1.74	9.29	1.29	0.81	7.68	1.07	0.67	6.17
0.59	0.68	1.9	5.05	0.47	1.09	7.43	0.69	1.60	4.13
0.43	1.01	3.4	4.76	1.8	0.91	4.71	1.78	0.90	3.38
1.74	0.42	2.96	6.41	1.03	0.25	15.26	2.45	0.60	28.75

0.7	0.7	1.15	2.4	2.02	0.83	3.43	2.89	1.19	7.41
0.62	1.97	1.95	1.6	1.53	0.28	0.81	0.78	0.14	7.64
1.29	0.7	3.97	2.5	1.63	0.48	3.57	2.33	0.69	4.48
0.47	0.2	0.62	19.88	1.05	1.68	99.4	5.25	8.40	16.3
0.79	0.32	1.94	8.74	0.89	0.61	27.31	2.78	1.91	27.29
1.03	0.75	3.24	0.98	0.8	0.18	1.31	1.07	0.24	6.74
0.76	0.47	1.58	2.83	0.24	0.27	6.02	0.51	0.57	10.33
0.94	1.06	2.24	2.05	1.8	0.61	1.93	1.70	0.58	5.28
1.07	1.03	4.13	2.8	1.93	0.64	2.72	1.87	0.62	6.52
0.84	0.94	2.48	3.48	2.31	0.36	3.7	2.46	0.38	3.49
3.1	0.26	4.75	5.19	0.84	0.74	19.96	3.23	2.85	4.13
1.4	1.45	1.95	1.13	0.94	0.16	0.78	0.65	0.11	4.38
1.67	0.36	1.04	7.2	2.65	1.15	20	7.36	3.19	12.33
1.37	0.51	3.32	2.63	0.65	0.44	5.16	1.27	0.86	2.51
0.57	0.4	1.81	2.99	1	0.48	7.48	2.50	1.20	8.29
1.3	0.14	1.32	7.84	1.03	1.08	56	7.36	7.71	6.76
1.03	0.57	2.01	4.74	0.6	0.46	8.32	1.05	0.81	8.65
0.83	1.04	2.61	4.76	1.37	0.72	4.58	1.32	0.69	4.74
0.56	0.86	2.09	2.14	1.74	0.48	2.49	2.02	0.56	5.5
1	0.32	2.58	4.65	0.64	0.46	14.53	2.00	1.44	3.26
0.78	1.21	1.99	3.76	1.47	0.5	3.11	1.21	0.41	4.53
0.77	0.67	2.14	1.59	1.06	0.33	2.37	1.58	0.49	3.35
0.53	1.12	2.63	4.71	0.91	0.5	4.21	0.81	0.45	3.98
0.52	1.71	1.37	3.59	0.31	0.63	2.1	0.18	0.37	6.39
1.86	0.84	3.1	4.98	2.17	0.35	5.93	2.58	0.42	4.3
0.42	0.42	0.7	1.57	0.95	0.31	3.74	2.26	0.74	3.56
0.81	1.05	2.84	3.94	0.83	0.35	3.75	0.79	0.33	4.97
0.73	0.95	1.87	1.54	1.07	0.2	1.62	1.13	0.21	3.9
1.36	0.9	1.64	3.09	0.68	0.41	3.43	0.76	0.46	9.19
1.05	0.25	2	4.54	1	0.56	18.16	4.00	2.24	2.7
0.47	0.66	1.59	2.92	1.32	0.35	4.42	2.00	0.53	3.37
1.94	0.32	2.27	7.85	0.67	0.63	24.53	2.09	1.97	29.16
0.7	0.83	1.31	1.42	0.99	0.31	1.71	1.19	0.37	6.17
1.2	0.86	2.29	1.88	0.79	0.16	2.19	0.92	0.19	8.3
0.43	0.12	0.74	8.52	1.07	0.62	71	8.92	5.17	8.07
0.41	0.46	0.93	1.65	0.59	0.26	3.59	1.28	0.57	3.84
0.51	1	3.02	6.08	1.62	0.58	6.08	1.62	0.58	4.35
0.79	0.75	2.09	2.27	0.53	0.29	3.03	0.71	0.39	6.02
0.89	0.68	1.49	7.82	1.23	0.65	11.5	1.81	0.96	8.6
0.68	0.71	1.67	3.19	0.62	0.64	4.49	0.87	0.90	2.64
0.52	0.74	1.78	3.87	1.56	0.52	5.23	2.11	0.70	7.88
0.28	0.74	0.9	8.05	1.01	0.86	10.88	1.36	1.16	5.71
0.44	0.18	1.1	6.01	0.75	0.7	33.39	4.17	3.89	4.75
0.86	0.96	2.9	2.2	0.48	0.37	2.29	0.50	0.39	4.27

0.54	0.98	1.89	2.34	0.97	0.24	2.39	0.99	0.24	4.81
0.79	0.7	2.76	6.11	1.56	0.77	8.73	2.23	1.10	10.57
0.93	0.88	1.86	1.42	0.64	0.27	1.61	0.73	0.31	2.33
0.95	0.41	1.42	18.84	0.5	1.11	45.95	1.22	2.71	22.27
0.67	0.52	1.26	4.08	0.63	0.29	7.85	1.21	0.56	7.32
1.1	0.6	2.1	1.96	0.64	0.22	3.27	1.07	0.37	5.25
1.09	0.5	2.11	3.25	0.77	0.32	6.5	1.54	0.64	6.03
0.87	0.85	2.41	7.49	1.1	0.68	8.81	1.29	0.80	4.65
1.2	0.43	1.55	8.59	1.06	1.19	19.98	2.47	2.77	5.55
1.08	1.53	3.56	2.09	0.59	0.25	1.37	0.39	0.16	6.32
0.66	0.27	1.01	3.39	0.78	0.86	12.56	2.89	3.19	3.93
0.55	0.89	2.05	2.36	0.48	0.28	2.65	0.54	0.31	6.41
1.45	0.69	8.52	6.53	1.03	0.26	9.46	1.49	0.38	4.42
0.74	1.81	1.47	2.9	1.09	0.32	1.6	0.60	0.18	9.44
1.59	0.46	2.84	4.3	1.33	0.63	9.35	2.89	1.37	5.44
0.39	0.28	1.56	0.96	1.03	0.22	3.43	3.68	0.79	7.08
1.39	0.91	2.99	2.61	1.31	0.41	2.87	1.44	0.45	3.26
0.45	1.29	2.09	1.72	0.62	0.3	1.33	0.48	0.23	5.21
0.75	0.76	1.19	3.69	1.04	0.79	4.86	1.37	1.04	4.03
0.87	1.12	2.14	5.36	0.74	0.55	4.79	0.66	0.49	4.12
1.32	1.08	1.76	2.28	0.7	0.16	2.11	0.65	0.15	6.99
0.68	0.27	1.07	0.83	0.36	0.22	3.07	1.33	0.81	6.01
1.17	0.32	4.56	6.54	1.23	0.53	20.44	3.84	1.66	3.53
0.79	0.6	2.37	1.66	0.51	0.37	2.77	0.85	0.62	7.11
0.64	0.64	3.46	2.29	0.7	0.52	3.58	1.09	0.81	2.71
0.76	0.86	3.78	7	0.65	0.66	8.14	0.76	0.77	6.25
0.72	0.44	1.33	4.91	0.84	0.51	11.16	1.91	1.16	10.17
0.81	0.11	1.32	2.11	0.58	0.52	19.18	5.27	4.73	5.78
0.71	0.12	0.93	9.13	0.83	0.49	76.08	6.92	4.08	21.29
0.63	0.49	1.51	5.61	0.49	0.26	11.45	1.00	0.53	7.34
1.14	0.25	2.03	2.77	2.65	0.55	11.08	10.60	2.20	4.82
0.5	0.69	0.55	4.06	0.29	0.17	5.88	0.42	0.25	13.04
1.09	1.47	3.61	0.84	0.48	0.13	0.57	0.33	0.09	6.08
0.53	0.33	1.06	5.75	1.18	0.75	17.42	3.58	2.27	2.04
0.77	0.83	2.82	5.44	1.45	0.85	6.55	1.75	1.02	6.01
0.58	1.18	1.58	2.43	0.7	0.37	2.06	0.59	0.31	7.83
0.24	0.14	0.67	18.22	0.89	0.8	70	6.36	5.71	22.95
0.85	0.58	1.92	6.56	0.68	0.64	11.31	1.17	1.10	7.29
0.76	1.35	2.73	2.1	0.38	0.17	1.56	0.28	0.13	8.26
0.72	1.35	2.56	2.32	1.54	0.39	1.72	1.14	0.29	6.08
2	0.59	1.77	2.27	1.15	0.3	3.85	1.95	0.51	3.78
1.36	0.19	3.19	16.49	1.29	0.12	86.79	6.79	0.63	15.6
1.13	0.99	2.58	3.23	1.16	0.24	3.26	1.17	0.24	7.2
0.24	0.37	1.02	4.67	0.68	0.86	12.62	1.84	2.32	3.16

0.8	1.24	2.43	4.88	0.89	0.29	3.94	0.72	0.23	10.27
0.23	0.54	1.17	2.25	0.95	0.65	4.17	1.76	1.20	5.36
0.95	2.08	2.7	2.47	1.28	0.18	1.19	0.62	0.09	5.33
0.72	0.31	4.69	3.87	1.28	0.01	12.48	4.13	0.03	5.48
0.52	0.72	1.11	1.39	0.21	0.24	1.93	0.29	0.33	5.87
1.14	1.08	4.3	6.51	3.55	0.92	6.03	3.29	0.85	5.34
1.21	0.75	1.65	5.5	0.6	0.8	7.33	0.80	1.07	9.97
0.74	1.32	1.22	6	0.82	0.42	4.55	0.62	0.32	11.39
1.64	0.35	1.45	3.81	0.87	0.45	10.89	2.49	1.29	10.82
0.48	1.52	1.78	7.72	1.95	2.53	5.08	1.28	1.66	6.18
0.84	1.25	2	1.8	0.55	0.19	1.44	0.44	0.15	6.73
0.82	0.96	2.18	1.77	1.69	0.43	1.84	1.76	0.45	2.26
0.81	0.96	1.84	2.2	0.81	0.26	2.29	0.84	0.27	5.71
0.62	0.32	2.79	15.63	2.27	1.39	48.84	7.09	4.34	4.82
0.45	0.61	2.08	1.61	0.38	0.18	2.64	0.62	0.30	11.43
7.45	0.56	1.1	2.16	0.94	0.21	3.86	1.68	0.38	3.88
0.77	0.55	2	6.2	1.9	0.5	11.27	3.45	0.91	3.99
0.44	0.18	1.46	12.75	1.72	0.18	70.83	9.56	1.00	7.38
0.37	0.12	0.8	11.93	4.12	1.82	99.42	34.33	15.17	13.21
0.7	0.57	3.9	5.37	0.88	0.54	9.42	1.54	0.95	11.37
0.83	2.02	1.64	1.2	0.6	0.1	0.59	0.30	0.05	9.78
0.56	0.85	2.02	4.69	0.63	0.49	5.52	0.74	0.58	3.84
0.82	0.74	1.45	1.82	0.41	0.06	2.46	0.55	0.08	12.04
0.82	0.95	2.59	2.05	1.2	0.34	2.16	1.26	0.36	7.09
0.57	1.2	1.95	2.01	1.7	0.37	1.68	1.42	0.31	4.37
0.38	0.32	0.76	3.73	0.44	0.51	11.66	1.38	1.59	4.51
1.02	0.94	6.03	3.4	1.5	0.3	3.62	1.60	0.32	6.66
1	1.38	2.11	0.9	0.3	0.1	0.65	0.22	0.07	2.85
1.04	0.24	1.57	13.5	1.01	0.7	56.25	4.21	2.92	14.68
0.64	0.14	2	7.87	0.78	0.59	56.21	5.57	4.21	4.84
0.45	0.63	2.6	1.74	1.24	0.17	2.76	1.97	0.27	4.31
1.23	1.05	2.84	2.03	1.2	0.62	1.93	1.14	0.59	3.47
1.5	0.27	4.22	5.55	0.94	0.51	20.56	3.48	1.89	1.46
0.66	0.11	0.86	7.86	0.94	1.04	71.45	8.55	9.45	33.72
1.3	1.04	2.37	4.99	0.91	0.41	4.8	0.88	0.39	11.89
1.44	0.97	1.88	1.8	0.7	0.2	1.86	0.72	0.21	5.32
0.55	0.5	0.91	1.76	0.74	0.3	3.52	1.48	0.60	5.52
0.99	0.95	2.61	1.4	1.9	0.4	1.47	2.00	0.42	4.16
0.78	0.91	3.13	3.58	1.8	0.91	3.93	1.98	1.00	4.95
2.15	0.95	3.11	3.69	1.92	0.43	3.88	2.02	0.45	6.43
0.57	0.89	2.71	3.46	0.38	0.22	3.89	0.43	0.25	5.11
1.33	0.94	2.22	2.64	1.25	0.55	2.81	1.33	0.59	4.28
0.78	0.55	2.58	2.14	0.51	0.23	3.89	0.93	0.42	4.07
3.28	0.97	1.3	1.16	0.75	0.24	1.2	0.77	0.25	6.18

1.17	0.9	1.74	6.39	2.36	1.45	7.1	2.62	1.61	13.57
0.36	1.87	1.64	2.21	0.71	0.37	1.18	0.38	0.20	10.09
0.57	1.35	2.65	2.1	1.7	0.4	1.56	1.26	0.30	8.53
0.66	1.05	0.53	1.17	0.55	0.19	1.11	0.52	0.18	6.18
0.84	1.38	2.66	2.97	1.14	0.36	2.15	0.83	0.26	5.45
1.12	0.79	3.69	4.51	1.6	0.55	5.71	2.03	0.70	5.51
1.66	0.18	1.15	7.02	0.51	0.26	39	2.83	1.44	13.45
1.32	0.58	1.5	0.78	0.41	0.03	1.34	0.71	0.05	21.86
1.87	0.81	2.97	1.49	0.9	0.43	1.84	1.11	0.53	4.75
2.7	0.69	2.14	3.58	2.99	0.55	5.19	4.33	0.80	5.48
0.34	1.32	1.22	0.79	0.35	0.18	0.6	0.27	0.14	8.83
0.43	1.31	1.04	8.92	0.48	0.56	6.81	0.37	0.43	4.56
0.4	0.65	1.22	2.92	0.78	0.55	4.49	1.20	0.85	4.82
1.25	0.61	1.71	6.04	2.11	0.87	9.9	3.46	1.43	16.79
0.43	0.17	1.97	10.85	1.08	0.75	63.82	6.35	4.41	9.61
0.57	0.64	1	3.37	0.75	0.29	5.27	1.17	0.45	10.44
0.85	0.52	1.99	5.29	1.28	0.72	10.17	2.46	1.38	3.04
0.94	0.86	3.37	3.18	0.68	0.36	3.7	0.79	0.42	5.8
1.35	1.05	6.44	6.11	2.09	0.73	5.82	1.99	0.70	2.25
2.33	0.51	5.69	10.26	0.5	0.63	20.12	0.98	1.24	7.79
2.28	0.14	1.01	7.65	0.5	0.4	54.64	3.57	2.86	4.72
0.81	1.16	1.84	4.66	0.9	0.38	4.02	0.78	0.33	3.8
1.85	0.11	1.75	3.58	0.37	0.33	32.55	3.36	3.00	8.68
1.59	0.13	2.87	2.16	0.31	0.31	16.62	2.38	2.38	9.25
1.25	1.09	1.83	4.54	1.45	0.5	4.17	1.33	0.46	5.35
0.55	0.82	1.89	2.39	1.35	0.29	2.91	1.65	0.35	3.15
1.46	0.44	1.65	3.53	0.5	0.47	8.02	1.14	1.07	5.09
0.46	1.08	0.53	3.04	1.35	0.49	2.81	1.25	0.45	6.29
0.85	0.94	2.05	2.94	1.41	0.38	3.13	1.50	0.40	6.44
0.6	0.72	1.81	3.03	0.69	0.56	4.21	0.96	0.78	3.49
0.72	0.29	2.91	8.45	0.98	0.85	29.14	3.38	2.93	10.02
2.28	0.68	1.67	4.52	1.61	0.54	6.65	2.37	0.79	4.45
0.92	1.28	2.12	3.36	0.95	0.38	2.63	0.74	0.30	5.56
1.08	0.57	3.11	9.35	0.99	0.82	16.4	1.74	1.44	8
1.91	1.14	2.91	2.64	2.19	0.33	2.32	1.92	0.29	3.53
0.56	1.27	1.93	2.11	1.38	0.54	1.66	1.09	0.43	8.69
0.41	0.33	1.27	3.27	1.08	0.41	9.91	3.27	1.24	7.58
0.8	0.78	2.02	3.2	3.25	0.89	4.1	4.17	1.14	4.51
0.94	0.45	1.21	3.18	0.29	0.25	7.07	0.64	0.56	5.86
0.8	0.76	1.04	2.9	1.57	0.28	3.82	2.07	0.37	6.85
3.61	0.7	5.31	11.31	0.88	0.5	16.16	1.26	0.71	5.28
0.83	1.27	3.02	2.79	1.73	0.53	2.2	1.36	0.42	6.44
0.72	1.02	2.03	3.03	0.87	0.39	2.97	0.85	0.38	5.35
0.93	0.62	2.08	5.76	0.47	0.27	9.29	0.76	0.44	5.86

0.78	0.92	0.92	0.72	0.52	0.16	0.78	0.57	0.17	11.12
0.91	0.54	1.86	6.56	1.6	0.92	12.15	2.96	1.70	6.39
0.67	0.73	1.53	3.03	0.74	0.44	4.15	1.01	0.60	7.18
2.07	0.63	1.74	0.8	0.71	0.18	1.27	1.13	0.29	2.53
0.73	0.63	3.02	6.93	1.33	0.64	11	2.11	1.02	6.93
0.58	1.28	1.94	2.26	0.82	0.22	1.77	0.64	0.17	28.64
0.43	0.66	1.28	1.88	0.65	0.47	2.85	0.98	0.71	2.97
0.49	0.86	2.65	3.71	0.68	0.35	4.31	0.79	0.41	3.38
0.97	0.49	2.19	5.6	1.08	1	11.43	2.20	2.04	6.82
0.5	1.41	2.7	4.73	0.68	0.43	3.35	0.48	0.30	5.41
1.18	0.88	2.66	1.64	1.37	0.2	1.86	1.56	0.23	5.05
1.19	0.88	1.53	4.66	0.9	0.32	5.3	1.02	0.36	4.49
0.86	0.81	2.05	1.45	0.3	0.02	1.79	0.37	0.02	3.64
2.25	0.63	2.8	3.94	2.16	0.44	6.25	3.43	0.70	4.57
1.31	0.81	1.94	1.59	0.9	0.35	1.96	1.11	0.43	3.8
0.93	0.33	2.27	3.18	0.85	0.37	9.64	2.58	1.12	8.02
0.56	1.06	1.28	6.76	1.62	0.7	6.38	1.53	0.66	6.11
2.1	0.92	4.38	8.16	1.32	0.69	8.87	1.43	0.75	2.13
0.33	1.14	1.49	1.49	0.77	0.32	1.31	0.68	0.28	4.65
0.72	1.2	1.08	1.58	0.61	0.23	1.32	0.51	0.19	4.21
0.63	0.93	1.05	1.61	0.82	0.24	1.73	0.88	0.26	4.3
2.82	0.13	2.94	6.9	1.53	0.93	53.08	11.77	7.15	7.04
1.65	0.54	1.94	0.81	0.43	0.27	1.5	0.80	0.50	3.54
1.02	0.96	1.95	1.94	0.94	0.35	2.02	0.98	0.36	7.58
1.11	0.89	2.99	2.62	1.9	0.39	2.94	2.13	0.44	5.36
0.47	0.97	2.21	4.05	0.73	0.26	4.18	0.75	0.27	9.77
0.96	0.64	2.25	1.41	0.8	0.13	2.2	1.25	0.20	3.87
0.99	0.06	1.36	8.63	1.21	1.55	74	20.17	25.83	22.94
1.67	0.5	2.15	2.72	0.9	0.09	5.44	1.80	0.18	6.3
0.4	0.51	0.9	3.11	0.86	0.31	6.1	1.69	0.61	8.73
0.94	0.11	1.99	11.06	0.57	0.45	90.55	5.18	4.09	58.82
0.84	0.5	2.27	7.59	0.92	0.73	15.18	1.84	1.46	5.34
0.73	0.72	1.64	0.92	0.64	0.3	1.28	0.89	0.42	4.55
0.55	0.42	1.11	2.29	1.33	0.21	5.45	3.17	0.50	6.26
0.96	0.26	2.25	3.58	1.18	0.58	13.77	4.54	2.23	3.26
0.48	1.14	3.52	3.51	1.22	0.52	3.08	1.07	0.46	5.79
0.81	0.68	1	5.51	2.09	0.79	8.1	3.07	1.16	8.85
0.31	0.8	1.52	1.94	0.68	0.4	2.43	0.85	0.50	3.16
1.24	0.58	1.42	2.42	0.92	0.2	4.17	1.59	0.34	6.16
1.08	0.51	3.07	6.23	1.16	0.85	12.22	2.27	1.67	3.53
0.9	0.26	1.81	15.54	1.23	0.71	59.77	4.73	2.73	10.95
0.51	0.61	2.1	8.16	1.3	1.03	13.38	2.13	1.69	3.73
0.67	0.8	2.24	0.86	0.71	0.16	1.08	0.89	0.20	3.97
0.75	0.56	1.72	3.79	0.75	0.56	6.77	1.34	1.00	9.83

0.91	0.43	1.19	2.36	0.97	0.44	5.49	2.26	1.02	4.87
1.07	0.86	4.87	3.17	0.6	0.64	3.69	0.70	0.74	12.16
1.01	0.07	1.95	4.95	0.65	0.26	70.71	9.29	3.71	29.56
0.98	0.51	2.8	2.38	0.76	0.49	4.67	1.49	0.96	4.67
2.24	0.23	3.33	4.31	0.43	0.35	18.74	1.87	1.52	5.01
0.63	0.59	2.96	6.5	2.12	0.65	11.02	3.59	1.10	8.8
0.89	0.7	2.32	7.17	1.37	0.81	10.24	1.96	1.16	4.6
0.76	0.24	1.72	3.69	0.69	0.45	15.38	2.88	1.88	15.48
0.5	0.14	0.77	12.05	1.04	1.66	86.07	7.43	11.86	10.2
1.23	0.52	2.78	4.93	1.22	0.31	9.48	2.35	0.60	17.02
0.98	0.33	2.07	11.2	2.14	1.09	33.94	6.48	3.30	26.16
1.07	0.11	1.92	4.07	0.68	0.48	37	6.18	4.36	5.63

Cr	PT	PTA%	INR	WBC	PLT	HBeAg	IBV-DN	AFP	aryMult	izecm1(
63.8	11.6	93.1	1	4.52	109.4	1	2	2.1	1	1
94.4	11.5	79	1	6.53	103	2	2	210.2	1	1
56.8	14.4	68	1.33	4.46	93	2	2	19.2	1	0
57.6	13.5	73.3	1.16	3.15	84	2	2	37.7	1	1
61	13	82	1.1	2.37	79	2	2	4.2	2	0
67	14	59	1.09	4.86	72.9	2	3	38.3	2	1
72	11.7	102	1.08	2.29	73	2	2	213.6	1	1
95	12.2	89	1.05	4.93	143.5	2	2	5.4	1	0
72	12.2	86	0.95	3.94	78.4	2	1	9.6	1	1
59	11.4	85	0.88	3.51	77.4	2	2	12.7	1	1
80	10.9	92	0.84	5.23	152.3	2	2	3.2	1	1
66	12.3	74	0.95	4.81	120.9	2	2	2.3	2	1
100	15.5	59	1.33	2.77	51.9	2	2	8.6	1	1
110	12.4	87	1.07	3.17	48.2	2	2	84.1	2	1
53	14.3	67	1.23	2.29	69.5	2	2	4.7	2	1
56	14	68	1.09	3.35	37.6	2	1	279.8	1	1
79	11.5	97	0.89	5.69	119.8	2	3	>350.0	2	1
65	16.1	60	1.4	1.48	37.2	2	2	47.5	2	1
72	12	77.8	1.12	5.33	124.4	2	3	188.9	1	1
64	15.5	51.9	1.25	4.73	86.2	2	2	41.2	1	1
209.2	17.5	48.7	1.5	5.45	69	2	1	42.9	1	1
61	11.2	102	0.96	3.98	123.3	2	3	21.2	1	1
58	13.5	73	1.05	3.79	184.2	2	1	>350.0	2	0
81	13.5	83	1.05	1.96	68.3	2	1	14	1	1
54	14.3	66	1.11	1.7	64.6	2	2	198.2	2	1
53.5	13.6	71.7	1.11	2.6	52	2	1	28.4	2	1
66.7	14	68.5	1.14	4.1	178	2	1	57.2	1	1
72.3	13.9	69.3	1.13	10.31	112	2	1	>350.0	1	1
51	12.2	91	1.05	2.68	41.2	2	1	4.4	1	1
86	14.2	67.7	1.22	5.34	82	2	2	2.3	1	1
68	12.1	88	0.94	4.6	72.9	2	3	165.9	2	1
81	13.5	73	1.05	2.9	36.1	2	1	6.2	2	1
73	11.1	103	0.86	2.59	77.9	2	3	3.4	2	0
88	10.8	109	0.84	5.9	96.6	2	3	1032	1	0
116.2	14.1	68.5	1.21	5.06	93	2	2	473.9	1	0
63	11.4	98	0.88	4.14	121.9	1	1	327.5	1	1
88	12	89	0.93	6.14	159.8	2	3	13.2	1	1
42	11.6	90	1.07	2.55	62.6	2	2	1.5	2	1
54	12.9	83	1	1.6	66.4	1	2	590.5	1	1
82	12.4	84	0.96	5.74	121.8	2	2	18.3	2	1
72	11.1	89	0.86	6.41	83.3	1	2	>350.0	1	1
55	11.6	87	1.07	4.09	68.2	2	1	181	1	1
60	10.9	107	0.84	6.3	160.1	1	3	28.5	1	1

67	11	105	0.85	6.64	167.5	2	2	31.3	1	0
75	14.3	71	1.19	2.59	35.2	1	3	25.6	2	1
56	12.6	82	0.98	3.63	65.1	1	1	323.5	2	0
78.9	13.1	76.9	1.13	5.27	58.7	1	2	183.5	2	1
30	12.9	79	1	2.94	65.6	1	1	99.5	1	1
84	13.3	80	1.02	2.54	52	2	2	5.2	1	1
62	12.5	83	0.97	4.67	95.2	2	2	101	2	1
67	12.9	79	1	2.51	82.7	1	1	5.6	2	1
67	11.7	94	0.91	4.66	143.3	2	2	4223	1	1
56	12.8	77	0.99	3.27	89.5	1	3	161.7	1	1
37.4	14.4	65.5	1.17	4.5	109	2	2	6.4	1	0
78	11.6	90	0.9	4.94	134	2	2	>350.0	1	0
44	13.9	68	1.08	9.06	145.5	2	1	213.2	1	0
70	11.1	96	1.03	6.78	172.7	2	2	127.3	1	0
91	12.6	76	1.16	3.27	55.6	2	3	143.2	2	1
80	13.8	70.1	1.13	3.56	61.8	2	2	2.5	1	1
61	12.9	78	1.19	3.1	56	2	3	2.9	1	1
49.5	12.3	84.1	1.01	2.9	93	2	3	>350.0	1	1
65	11.7	91.8	1.01	2.45	41.8	2	3	5.6	1	1
66	12.1	87	0.94	6.43	161.5	2	1	97.3	2	0
72	13.3	75	1.03	3.72	114.6	2	2	6.5	1	1
56	11.4	96	0.88	3.25	86.6	2	2	8.2	1	1
88	10.6	113	0.85	5.13	114.9	2	2	45.7	2	1
42	16.2	55	1.39	2.58	39.7	2	3	13.3	2	1
77	10.5	111	0.81	4.07	196.2	2	1	60.4	1	1
63	13	77	1.01	2.24	63.6	2	1	66.9	1	1
79	12	88	0.93	5.65	155.1	2	1	575.3	2	1
85	14.2	77	1.1	2.8	59	2	1	14.3	1	1
78	12.3	90	0.96	2.74	103.2	2	2	4.3	1	1
79.2	12.6	81.7	1.09	7.94	165	2	2	51.6	1	1
98.2	21.8	34.5	1.87	6.41	12.4	2	2	4.3	1	0
90	13.4	72.9	1.09	3.13	75.2	2	2	112.5	1	0
76	13.2	77	1.13	6.49	211.5	2	2	1	1	1
42.5	16.5	52.8	1.34	3.7	73	2	1	9	1	1
126	13	72	1.2	6.75	191	2	2	2.9	1	1
58.1	14.4	66.7	1.24	4.7	121	2	3	>1000.0	2	1
70.8	13.4	85	1.04	4.5	94	2	1	12.5	1	0
59	16	55.4	1.3	3.5	19	2	2	42.6	1	1
56	11.1	105	0.89	4.75	92.8	2	2	7.3	1	1
58	13	82	1.01	2.93	66.5	2	2	8.8	1	0
76	12.5	88	1.09	3.68	187	2	2	>2000.0	1	1
79	13.4	63	1.13	19.61	116	2	1	2.2	1	1
70	11.5	105	1.06	5.18	241	2	1	6231	1	0
69	13.4	85	1.04	4.12	82.9	2	1	9.8	2	1

114.2	20	41.2	1.72	4.04	71.9	1	1	6.7	2	1
77	10.8	88	0.95	7.41	209.9	1	2	2.3	1	1
52	12.8	92	0.99	2.46	65.6	1	2	3.2	1	1
58	14.1	67.4	1.15	2.26	44.2	2	2	>350.0	2	0
64	10.9	124	0.84	7.33	151.6	1	2	2.7	1	1
63	13.4	85	1.04	1.99	29.8	1	1	331.8	1	1
52	11.3	103	0.99	4.85	141.3	1	3	69.3	2	1
76	12.3	84.9	1.06	4.82	187.5	1	2	3.3	1	1
43.5	11.2	104	0.89	3.62	53	1	3	188.5	1	1
63	10.5	117	0.91	5.73	130.3	2	2	787.7	1	1
97.6	12.6	80.2	1.03	9.2	190	2	2	3.4	2	1
79	13	77.8	1.12	4.81	118.3	1	3	23.4	1	1
76	13.9	79	1.08	5.11	84.4	1	3	31	1	0
73.8	12.6	80.2	1.03	2.8	53	1	1	8.2	2	1
80.7	12.6	80.2	1.03	3.82	249	2	2	12.1	2	1
57.1	11.8	88.8	0.97	3.62	175	0	1	>350.0	1	0
69.6	12.1	87.1	1.04	5.63	134	0	2	90.3	1	0
83	13.3	86	1.03	2.22	28.2	1	2	42.1	1	0
69	12.6	94	0.98	4.38	108.9	1	1	37	1	0
46	12.1	81	1.12	1.56	21.8	0	2	1.5	2	1
54	13.3	86	1.03	3.75	97	0	1	10.9	1	0
70	13.2	87	1.02	3.65	84.8	1	1	4.1	1	1
80	12.6	94	0.98	2.9	87.6	0	3	5	1	1
61.2	14.7	63.2	1.2	4.1	67	1	3	206.6	2	1
65.5	13.2	74.6	1.08	5.9	183	0	1	>58344.[illegible]	2	0
67	12.1	92	0.95	2.54	74.6	0	2	5.3	1	1
79	12.4	82.2	1.02	8.3	207.7	0	2	37.5	1	1
52	13.3	86	1.03	3.4	70.8	1	1	13.6	2	1
69	12.1	85.4	0.99	6.76	229.1	1	3	7813	1	0
68	11.8	106	0.91	4.63	153.3	0	2	>350.0	1	0
63.9	13	93.1	1	7.87	180	0	1	2.1	1	1
81	11.3	116	0.88	5.82	190.2	0	3	6148	1	1
77	11.4	114	0.88	3.61	133.8	1	2	6.6	1	0
74	13.8	80	1.07	6.1	122	0	1	>350.0	2	1
59	11.4	114	0.88	4.61	75.8	1	2	9.5	2	1
74	11.8	106	0.91	5.71	180.3	1	2	50.4	2	0
62	13.5	72.1	1.1	6.17	127.3	0	1	199.4	1	0
55	11.6	91	1.07	2.42	73	1	3	34.3	2	1
54	16.8	59	1.3	3.31	56.1	0	1	16.6	2	1
56	13.1	75.5	1.07	5.3	126.6	0	2	3.7	2	0
68	12.2	100	0.95	5.49	163.1	1	3	61.4	1	0
66	11	108	0.97	5.71	91.4	1	3	12.9	1	1
46	11.6	110	0.9	4.23	72	0	2	6.7	1	1
64	12.1	101	0.94	5.11	138.1	0	1	3.5	1	1

54	12.8	79.7	1.1	3.24	51.2	1	2	7.2	1	1
54.2	11.8	95	0.93	3.43	71	0	2	19.5	1	0
65	11	122	0.87	3.15	122.1	0	2	>350.0	1	0
43	14.1	73	1.07	6.08	87.3	0	1	134.8	1	1
113	11.5	91	1.06	4.93	126	0	2	1.4	1	1
62.1	13.1	78	1.13	3.95	73.7	1	2	14.3	1	1
60	12.6	80.2	1.03	5.42	300.2	0	1	1977	1	0
44	12	103	0.94	6.03	122.3	0	1	35.1	1	1
85	13.6	71.2	1.11	2.73	89.8	1	1	3.8	1	1
78	12.4	97	0.97	4.48	129.6	1	2	5.4	1	1
86.4	13	76.4	1.06	4.9	177	1	3	>350.0	2	0
52	11.3	116	0.89	2.86	89	0	2	95.7	1	1
64	15.2	65	1.25	4.14	82.1	0	1	53.4	2	1
49	12.6	86	1.1	4.02	62	0	2	1.8	2	1
85	13.1	65	1.11	2.5	37.6	0	3	32.6	1	1
69	11.5	97	0.99	4.27	138.3	1	3	1.3	1	0
78	14.3	67	1.23	2.43	112.4	1	2	1.1	1	0
78	13.7	75	1.18	4.72	48.5	0	2	7.4	1	0
81	12.8	92	0.99	2.33	43.8	1	2	>350.0	2	1
52	12.1	85.4	0.99	2.92	65.9	0	2	325.7	1	1
59	14.2	77	1.08	2.76	38.2	0	1	6.9	1	1
65	13.5	78	1.18	2.32	36	0	2	106.3	2	1
68	11.5	99	0.91	3.6	130.8	0	2	46	1	1
62	15.8	63	1.18	2.24	57.5	0	2	10.2	1	1
83	11.9	105	0.93	3.43	159.9	0	2	936.2	1	1
55	17.9	54	1.32	2.68	92.1	0	1	>350.0	2	1
59	13.2	74.6	1.14	3.9	92.2	1	2	19068	1	1
60	12.1	92	0.95	3.18	57.1	1	1	170.3	1	1
67	12.5	96	0.97	3.86	149	0	1	86.7	2	1
65	11.6	110	0.91	5.01	157	1	1	20496	1	0
52	13.4	85	1.03	3.28	80	1	1	>350.0	2	1
80.8	19.9	46	1.41	3.58	60	1	3	63.7	2	0
88.9	12.3	83.2	1.06	4	201	0	2	>350.0	1	0
72	12.6	81.7	1.09	4.59	87.2	0	2	2.4	1	1
66	10.5	134	0.84	6.17	168.5	0	1	15.1	2	0
84	12.9	90	1	7.64	148.1	0	1	8.7	2	1
63	10.8	127	0.86	4.18	92.6	1	2	26763	1	0
93	12.6	86	0.98	10.3	483.9	1	1	7.2	2	0
67	14.6	63.8	1.26	3.84	72.4	0	1	2.9	2	0
112	12.4	87	1.07	5.95	87.5	0	2	630	1	1
40.4	10.9	108	0.87	5	101.3	0	1	80	1	1
77	11.2	104	0.98	5.08	75.4	1	2	5.6	1	0
78	12.5	87	0.98	4.13	66.7	0	2	4.7	1	1
59.5	13.3	80	1.23	3.75	82.4	1	2	2	2	1

55	11.5	99	0.91	2.49	96.5	1	2	152.5	1	1
84	12.2	84	1.13	5	76.5	1	2	232.4	2	1
74.9	13.6	71.3	1.17	5.27	68.8	1	1	94.8	2	1
92	13.7	73	1.27	1.4	38	1	2	2.9	1	1
71	15.8	56.5	1.36	4.13	55.8	1	1	76.2	1	0
83	12.6	80	1.16	2.69	39	1	2	294.9	1	0
68	11.7	97	0.92	4.52	144.2	1	1	31.1	1	1
81	11.2	104	0.89	5.23	168.1	0	2	51.9	1	1
56.1	17.5	48.7	1.5	3.12	49.9	1	3	1.6	1	0
65	13.4	77	1.24	6.33	34.4	0	2	2.4	1	1
58	11.9	94	0.94	6.33	14.2	0	2	18.1	2	1
56	14.6	70	1.1	6.33	14.2	1	3	5.4	2	1
78	13.4	79	1.17	6.33	14.2	1	3	37.5	1	1
63	11.9	86.2	1.03	6.33	14.2	1	3	5	1	1
70	10.7	111	0.86	6.33	14.2	0	1	5.9	2	0
58	18.6	51	1.34	6.33	14.2	0	3	3.2	2	1
50.7	12.9	83	1	2.71	29.2	1	2	9.2	2	1
83	16.5	58	1.44	3.02	47	1	2	10.5	2	1
75	13.3	73.6	1.15	4.48	95	0	1	13.3	1	1
67	10.7	111	0.86	1.38	37.2	1	3	15.4	1	1
59	11.3	102	0.9	3.82	67.9	1	2	4.6	1	1
40	13.5	72	1.16	6.74	493	1	3	>1000.0	1	0
87	9.7	131	0.8	3.62	135.9	0	2	5.5	2	1
79	10.8	111	0.95	3.26	97	0	2	17	2	1
81	12.4	88	0.97	2.52	66.5	1	2	488.9	1	1
81	12.5	87	0.98	8.02	238.8	0	2	31.4	2	1
53	10.7	111	0.86	3.36	97.6	1	2	576.1	1	1
44.8	14.6	64.4	1.26	5.4	87	0	2	3.9	1	1
60	12.7	84	1.1	3.27	64.1	1	2	107	1	1
93	13.2	75	1.22	3.5	51	1	2	8.1	2	1
75	12.3	82.2	1.06	5.74	103.3	1	1	7.7	2	1
77	11.5	99	0.91	4.65	117.3	1	1	217.4	1	1
52	10.9	108	0.87	5.97	190.2	1	1	5.1	2	1
73	11	97	1.02	4.8	143	0	2	1.5	1	1
42	11.2	104	0.89	5.58	219.8	0	2	92.8	1	0
52.8	15.2	60.8	1.31	1.16	38.4	0	2	34.7	1	1
40	15.9	49	1.3	2.45	45.8	1	2	1928	1	0
41	17	57	1.25	1.92	32.1	0	2	9.3	2	1
85	11.7	97	1.02	7.97	192	0	2	3.4	1	1
73	14.6	64.4	1.26	6.37	68	1	1	12.3	1	1
63	12.8	78	1.18	3.92	98.4	1	2	527.3	1	0
71	16.8	58	1.24	10.52	151.5	0	1	8496	2	0
63	12.4	88	0.97	1.64	67.1	1	2	29.7	1	1
65	10.6	101	0.98	6.6	115.9	0	2	3.7	1	1

60.4	14.2	67	1.22	6.81	138.2	0	2	>1000.0	2	0
63	12.3	90	0.96	2.81	103.1	1	1	>1000.0	2	1
70.5	11.7	97	0.92	3.41	53.2	0	1	43.2	1	0
48	11.8	101	1.09	4.52	201.8	0	2	4.3	1	1
69	11.3	93	1.05	5.93	200	0	2	52.7	1	1
51	12.1	85	1.12	2.14	51.5	0	2	58.8	1	1
73	14.9	62.8	1.28	6.59	48.2	1	2	4.5	2	1
56	14.9	62.6	1.28	3.62	49.7	0	2	6.6	1	1
56	13.3	76.1	1.15	3.81	142.3	1	2	297.4	2	1
62	10.7	111	0.86	5.7	194.7	0	2	3	1	1
56	13	82	1.01	2.54	27	1	3	98.7	1	1
70	12.6	86	0.98	3.57	77	1	2	5.5	1	1
72	12.4	88	0.97	3.83	36.7	0	2	2.6	2	1
55	13	92	1.11	5.34	109.2	1	1	5938	1	0
73	11.2	104	0.89	4.39	75.8	0	1	33.4	1	1
62.8	19.7	39.7	1.69	3.66	21.4	1	1	20.4	1	1
86	12.8	84	0.99	3.65	146.3	1	1	54.8	1	0
53	14	74	1.07	4.51	58	0	2	60.7	1	1
49	12.2	91	0.96	2.9	162.7	1	1	4.4	1	0
66	15	67	1.13	2.1	44.8	0	3	12.5	1	0
62	10	125	0.82	5.97	116.1	0	1	5.9	1	1
66	13.4	76	1.15	3.7	61.7	1	2	151.4	2	1
77	11	95	1.02	7.51	175.5	0	2	187	1	1
89	14	65	1.29	3.65	58.9	0	2	3.4	2	1
70	11.2	104	0.89	4.82	88.8	1	1	483.60	2	1
80	14.2	73	1.08	4.05	58.6	1	1	804.9	1	1
72	11	107	0.88	7.1	172.8	1	1	71.7	1	1
59	17.7	47.4	1.52	8	208.3	0	1	>1000.0	2	0
73	11.6	98	1.07	3.93	124	1	3	52.3	1	1
61	11.6	87	1.07	3.18	56.9	0	2	>2000.0	1	1
94	11.9	87	1.1	4.3	138	1	2	3	1	1
67	13.6	73.3	1.17	4.13	73.3	0	2	182.3	1	1
99	11.2	102	0.97	4.78	132.1	1	2	0.7	2	1
64	10.8	110	0.87	8.9	113.8	1	2	2.7	1	0
52	18.9	49	1.75	3.1	33	0	2	2.3	1	1
65	11.3	102	0.9	2.35	69.1	0	2	431.1	1	1
121	12.8	79.7	1.1	2.45	40	0	2	4.6	1	1
65	12.8	84	0.99	4.03	129.3	1	1	39.9	1	1
71	12.7	85	0.98	3.48	146.6	0	2	42.3	1	1
88	10.9	96	1.01	5.79	72	1	2	143.3	1	1
55.2	12.6	85	1.1	1.9	40	1	3	6.6	1	1
59	12.1	92	0.95	2.43	63.8	0	1	26.3	2	1
60	13	77.8	1.12	6.89	244	1	2	3.7	1	0
67	14.3	72	1.09	6.6	82.1	1	1	9.1	1	1

73	11.1	105	0.89	3.88	163.1	0	1	11457	2	0
42	9.9	114	0.92	4.05	143	0	2	506	1	0
54	11.9	92	1.03	5.41	30.1	1	3	33.2	2	0
58	12.4	88	0.97	3.39	122.1	0	1	17.7	1	1
89.9	11.4	102	0.99	3.11	81.7	1	1	3	1	0
73	11.9	94	0.94	3.18	90	1	1	21.5	1	1
138.8	15	62.1	1.29	5.65	86	0	2	2.8	1	1
56	13.8	71.6	1.19	9.31	149	1	1	319.6	1	0
63	10.6	113	0.85	4.89	110.5	1	1	852.2	2	1
52	11	107	0.88	6.39	319.2	0	2	414.1	1	1
81	10.9	10.9	10.9	5.9	161.8	1	1	3.3	2	1
110	11.6	98	0.92	8.17	196.1	0	2	3	1	1
44	11	107	0.88	4.77	165.4	0	3	353.4	2	0
45	15.1	67	1.13	2.72	54.3	1	3	7.4	2	1
52	11.9	91.5	1.03	4.49	70.4	0	2	699.5	2	0
73	12.6	83.2	1.09	4.42	89	1	1	119.1	2	1
62.2	16.7	61	1.55	4.43	54	0	2	>2000.0	2	1
76	13.4	75.2	1.15	3.68	197.1	0	1	4.6	2	1
77.9	15.5	59	1.33	3.86	104	1	1	281.6	2	1
64	15.6	60	1.44	4.1	49	1	3	4	2	1
71	12.3	90	0.96	4.08	55.8	1	1	55.1	2	1
69	13.5	78	1.04	2.95	108.1	1	3	8658	1	1
77.5	10.2	121	0.83	7.46	254.2	0	1	2359	1	1
67	14.6	65.2	1.26	3.46	83.6	0	2	7.6	2	1
94	13.8	76	1.06	6.78	148.3	1	1	7.9	2	1
60	11.9	94	0.94	4.47	142.5	0	2	13.3	1	1
84	15.3	61	1.42	2.2	27	1	2	431.5	1	0
100.3	12.5	87	0.98	7.68	211	0	1	>1000.0	2	1
71	17	57	1.25	2.63	37	0	1	31	1	1
61	11.4	92	1.05	6.3	373	1	2	4.6	1	0
54	13.2	81	1.02	5.45	117.5	1	2	3.2	1	1
53	12.9	73	1.19	2.84	76.2	1	2	2.7	1	0
66.1	13.6	73.3	1.17	3.53	79.3	0	1	8.2	2	1
56.8	15.2	61	1.31	5.57	57.3	0	1	6.8	1	1
43.3	16.3	60	1.21	1.99	46.4	2	1	99	1	0
49	15.1	62	1.4	3.42	107.4	2	1	3.1	1	1
49	13.9	75	1.06	3.6	139.8	2	1	29.1	1	1
68	14.7	64.5	1.26	2.61	37.6	2	2	5.8	1	0
65	12.1	92	0.95	2.7	76.3	0	2	18.5	1	1
71.3	12.6	83.2	1.09	5.93	103	2	2	8.1	1	1
60	15.3	65	1.31	2.55	39.4	0	1	156.5	1	0
53	13.3	76.1	1.15	11.33	261.7	0	1	738.1	1	1
74	14.6	65.2	1.26	5.19	132.5	2	2	11.3	2	0
98	12.5	84.3	1.08	5.18	135.3	2	1	25167	2	1

53.8	12.9	80.1	1.11	4.62	177	0	2	77.9	2	1
60	15.3	66	1.15	2.48	52	2	1	97.1	1	1
55	15.7	57.8	1.35	1.28	38.6	2	2	21.8	1	1
68	15	62.3	1.29	3.09	42.1	0	1	324.1	1	1
61	16.9	51.2	1.45	3.22	56	0	1	777.4	2	1
71	14.2	68.3	1.22	3.37	61.5	2	1	165.7	2	1
68	12.3	90	0.96	4.22	67.5	2	1	29	1	1
87	13.9	74	1.16	2.76	44.7	2	2	18	1	1
53.9	14.8	69	1.12	3.26	29.4	2	1	10.1	1	1
84	12.4	88	0.97	7.88	199.5	2	3	13.6	1	1
69	13.3	80	1.02	3.1	63.3	2	1	21.4	2	1
68.7	12.1	89	1.04	7.64	167	2	3	631.7	1	1
42.9	15.5	59	1.33	3.31	78	2	2	15.7	1	1
80	13	82	1.01	2.67	60.2	2	2	9286	2	1
88	10.3	105	0.95	12.14	171.2	2	2	1.5	2	1
77	12.8	84	0.99	3.39	83.2	2	1	8.8	1	1
130	13.8	70.8	1.19	4.3	50.3	2	2	>1000.0	2	1
64	10.6	113	0.85	7.07	146.1	2	1	3.5	1	0
40	14.5	65.5	1.25	2.6	55.9	2	2	722	1	1
69	13	77.8	1.12	8.5	124.1	2	1	191.9	2	1
91	11.1	105	0.89	3.95	81	2	2	5.7	1	1
75	11.3	102	0.9	4.95	94.1	2	2	3.7	1	1
72	10.4	117	0.84	3.9	85.3	1	1	33.4	2	1
84	12.3	90	0.96	4.55	104.5	1	2	71.2	1	1
60.5	14.4	66.2	1.24	5.83	118	1	2	>1000.0	2	1
63	12.3	88	1.06	2.65	67.5	1	2	9	2	1
57	13.7	71.7	1.18	2.89	99.3	1	2	163.6	1	1
111	11.5	99	0.91	9.07	224.2	1	1	7.3	2	0
76	9.3	142	0.77	5.65	85.9	1	3	5.8	1	1
81	10.3	119	0.84	4.45	116.9	1	2	4.4	1	1
73	13.2	75	1.22	2.84	87	1	2	1.9	2	1
62	15	67	1.13	2.58	43.4	1	2	21.2	1	1
64	10.8	110	0.87	5.66	96.2	1	2	3698	2	1
81	10.8	110	0.87	3.44	107.2	1	3	5.7	1	1
59	11.7	97	0.92	5.51	201.8	1	1	20.2	1	1
75	9.6	134	0.79	2.8	112.8	1	2	647.2	1	1
43	10.7	101	0.99	4.5	119.4	0	2	59.8	2	0
79	11.5	99	0.91	6.07	123.5	1	1	18	1	1
64	12.9	78.8	1.11	2.66	68.7	1	2	6.2	2	1
55	13.2	81	1.02	3.37	65.5	1	1	73.2	2	0
73	12.4	93	1.15	3.49	73	1	2	129.3	1	1
48	13.3	78	1.15	5.63	160.4	1	3	6.9	1	1
64	14.2	67.7	1.22	3.3	92.4	1	1	>1000.0	1	0
58.6	16.8	52	1.44	8.59	291	0	2	45.3	1	0

67	11.3	93	1.05	7.73	144	1	1	4.9	1	1
75	15.1	66	1.24	3.43	61.8	1	2	35.6	2	1
133	10.7	113	0.94	9.1	211	1	2	19.1	1	1
66	13	77	1.26	5.01	169.4	1	2	5	1	1
50	14.7	64.1	1.26	4.97	112	0	1	414.5	2	0
89	11.6	78	1.01	3.83	85.2	1	2	16.4	1	1
39	15	62.1	1.29	3.38	84.8	1	1	36.6	1	1
47.7	14.9	65	1.3	1.99	21.4	1	3	1610	1	0
61	12.9	83	1.1	10.77	361.7	1	2	3	2	0
64	14.6	64.8	1.26	7.99	208.4	1	3	20241	2	1
67	11.8	96	1.02	4.03	102.3	1	1	199.8	2	1
51	10.1	125	0.9	5.03	123.8	1	1	3.1	1	1
48	12.4	89	1.06	2.47	43.3	1	2	10.6	2	0
56	13.1	76.9	1.13	6.04	79.8	1	1	17.6	2	1
46	14.5	61	1.33	5.21	270	1	3	1.9	1	0
59	14.3	67	1.23	6.37	99.9	1	1	31	1	0
56	13.3	75.1	1.15	2.17	49.2	1	2	6.8	1	0
82	12.1	81	1.12	4.14	122.8	1	1	3231	1	0
67	12.1	87.1	1.04	9.77	102	1	2	15.8	2	1
53	13.3	80	1.12	4	45.4	1	2	27.96	1	1
79	13.8	75	1.16	3.71	57.4	1	1	58.1	1	1
48	13.1	76.9	1.13	6.55	144	1	1	161.6	1	1
85	11.9	92	1.03	4.3	107.4	1	2	7.7	1	1
66	11.8	96	1.02	3.2	71.1	1	1	5.8	1	1
72	13.6	76	1.18	1.92	41	1	2	4.2	1	1
89	11.7	97	1.02	3.8	135	1	1	>80000.	2	0
82	11.5	100	1	4.34	67.6	1	2	5.2	2	1
83	12.3	84.9	1.06	5.38	123	1	3	139	1	1
65	12.4	89	1.06	7.12	88	1	2	18.2	1	1
80	14.8	63.5	1.27	5.84	168	1	2	4.9	2	1
61	12.6	81.7	1.09	7.65	292.4	1	1	11.6	1	1
72	13.4	77	1.16	5.5	80	1	3	79	1	1
80	11.4	101	0.99	8.77	230.8	1	1	>1000.	1	0
60.1	12.9	78.8	1.11	5.14	65.9	1	1	28.7	2	1
60	11.5	100	1	2.42	54.3	1	1	29.2	1	1
61	12.8	84	1.09	4.3	56.6	1	1	4.2	2	0
68	12.7	85	1.08	7.09	128	0	1	>1000.	2	0
74	12.3	90	1.06	4.75	89.3	1	1	2075	1	1
69	13.5	73.3	1.16	4.17	123	1	1	24.7	1	1
70	12.4	87	1.07	2.79	18	1	2	6.8	1	0
62	11.4	80	0.99	5.73	176	1	3	69.3	2	1
59	12.5	82.8	1.08	6.01	160.3	1	1	270.6	1	1
80	12.5	70	1.07	5.73	77.3	1	1	>1000.	1	1
52	13.8	60	1.16	1.72	40.4	1	2	5.8	1	1

61	13.2	76	1.14	2.68	33	1	2	40.6	1	0
65	11.9	89.4	1.03	8.26	229.8	1	1	594.8	2	0
122	12.6	81.7	1.09	7.33	124.7	1	2	103.3	2	1
68.4	14.4	66.2	1.24	4.67	130	1	1	10.2	2	1
66	13.1	76	1.21	2.2	49	1	2	10.8	1	1
67	12.6	80	1.17	5.04	125.3	1	2	2.1	1	1
65	14.1	68	1.31	5.3	182	1	2	1.7	1	1
41	22.9	31.9	1.96	2.79	53	1	3	1.6	2	1
39	13.7	61	1.15	1.74	52	1	1	24.2	2	1
66	12.3	71	1.06	3.58	60.1	1	1	8603	2	1
64	14.6	68	1.26	2.95	47.6	1	2	793.6	1	1
67	10.7	101	0.99	4.39	118	1	2	2.6	2	1
69	11.5	97	0.99	2.37	70	1	2	9.1	1	1
88	11.3	81	0.99	4.63	87.2	1	1	3.6	1	1
63	11.7	91.8	1.01	3.63	139.1	1	2	2972	1	1
84	13.1	65	1.11	5.27	106.1	1	1	42.9	1	1
73	10.6	90	0.94	4.6	168	1	2	5	1	1
66	14.1	64	1.3	3.85	36	1	2	5.1	2	1
60	14.1	64	1.3	2.93	51.7	1	2	2.1	2	1
73.8	11.9	94	1.04	3.8	94.9	1	2	110.5	2	1
68	15	62.1	1.29	4.07	129	1	2	175.1	1	1
46	11.1	93	1.03	3.45	45.4	1	2	3.1	1	1
87	12.8	79.7	1.1	3.25	56.2	1	2	1110	1	1
69	13.8	75	1.19	2.56	73.1	1	2	3.2	2	1
71	12.2	86	1.05	4.24	112	1	2	3.8	1	1
53	12.8	79.7	1.1	3.28	193	1	1	268.6	1	1
52	12.3	83	1.14	3.82	394.1	1	3	3.2	2	1
70	11.6	96	1	4.74	136.5	1	1	3048	2	1
44.9	13.4	74.2	1.15	3.02	98	1	2	39.7	1	1
76	11.1	93	1.03	3.64	66	1	2	2.1	1	1
70	11.8	94	1.02	3.21	93.9	1	2	22.8	1	0
67	10.6	111	0.91	4.91	169.6	1	2	170.1	2	1
76	10.6	111	0.91	5.24	123.5	1	2	388.6	2	0
65	15.8	57.2	1.36	2.91	47.6	1	2	29.6	1	0
73	11.7	95	1.01	4.43	185	0	2	2364	1	1
49	14.1	68.5	1.21	3.03	115.6	1	3	3.4	2	0
59	14.7	64.1	1.26	3.33	65.5	1	1	50.1	1	1
66	13.6	72.5	1.17	2.79	84	1	1	20766	1	1
39	12.3	88	1.06	2.03	104.6	1	2	213.8	1	1
63	12	82	1.11	0.88	50.6	1	3	10.4	1	0
70	11.7	95	1.01	6.55	149.1	1	1	18.2	2	1
84	13.5	73.3	1.16	2.72	32.5	1	2	92.2	1	1
68	12.2	86	1.05	7.81	257	1	1	38.8	2	1
60	15.3	60.2	1.32	4.36	58.5	1	2	13.7	1	0

63	11.7	95	1.01	2.57	55.9	0	2	460.4	1	0
69	13.2	76	1.14	5.4	92.4	1	1	63.1	2	0
48.6	15.5	62	1.44	2.62	61.6	1	2	1	2	1
56	12.1	90	1.04	3.8	31.6	1	1	11.5	2	1
62	11.2	98.4	0.97	8.03	189	1	3	3.2	1	1
71	15.8	54	1.45	3.05	35	1	2	6.7	2	1
111	11.2	102	0.96	5.73	90.9	1	1	25.8	2	1
48	12.7	80.7	1.09	4.81	118.5	1	1	2.4	2	1
60	13.1	76	1.21	2.72	40.6	1	2	46.9	1	1
86	10.9	106	0.94	7.22	174.5	1	2	4.3	1	1
81	11.9	92	1.03	5.43	215	1	2	3.2	1	1
68.3	12.7	80.7	1.09	6.21	86.5	1	1	26.6	2	1
77	10.2	119	0.88	5.95	186.9	1	2	8.7	1	1
76	10.5	113	0.9	3.95	81.9	1	2	3.8	1	1
73	12.7	75	1.17	5.06	61	1	2	9.6	1	1
74	11	105	0.95	3.56	129.5	1	2	1.7	1	1
55	10.6	111	0.91	7.34	292.5	1	2	4.1	1	0
87	15.3	57	1.41	3.09	62.2	1	3	45.7	2	1
58	13	81	1.12	5.02	102.4	1	1	54.2	1	0
74	13.9	74	1.19	2.69	106.3	1	1	3.6	2	1
52	12.1	87.1	1.04	6.11	173.8	1	1	8.4	1	1
48	14.7	64.1	1.26	1.46	23.5	1	2	1.8	1	1
73	9.8	127	0.84	5.16	200.2	1	3	4.9	1	1
60	13.3	75.1	1.15	4.07	80	1	1	10.3	2	1
70	11.2	102	0.96	7.09	88.4	1	2	2.2	1	0
47	11.3	100	0.97	3.71	113.2	1	1	131.9	1	1
75	13.8	74	1.19	3.08	55.4	1	1	118.6	1	0
105	10.5	104	0.97	5.12	145	1	2	97.7	1	1
76	14.4	70	1.25	3.68	68.4	1	2	15.8	1	1
65	13.1	76.9	1.13	5.67	90.3	1	1	104.1	2	0
69	11.2	102	0.96	6.38	146.7	1	3	2	1	1
62	11.6	90	1.07	5.5	74	1	2	3	1	1
60	12.7	84	1.1	2.96	97.9	1	1	14.1	2	1
73	12.4	82	1.15	6.31	89.7	1	3	8.5	2	1
63	11	105	0.95	4.76	173.1	1	1	2.2	2	1
64	12.6	81.7	1.09	4.13	161.9	1	2	4.3	1	1
73	13	83.8	1.07	2.84	29.4	1	2	134.1	1	1
66.3	13.8	70.8	1.19	2.92	148.4	1	1	3.9	1	0
64	10.9	106	0.94	3.31	105.3	1	2	3.3	1	1
75	12.1	90	1.04	3.71	96.6	1	1	203.4	1	1
53	11.6	96	1	6.19	115.6	1	2	409.6	2	1
58.1	15.6	58.4	1.34	4.55	58	1	2	3.3	1	0
50	13.3	77	1.16	2.89	55	1	2	150.3	1	1
63	13.5	73.3	1.16	4.39	131.6	1	1	1370	1	1

70	11.3	100	0.97	3.08	141.8	1	2	74.6	2	1
55	10.5	113	0.9	3.87	165.7	1	1	322.3	2	1
68	14	69	1.3	1.75	21.9	1	2	6.6	2	1
86	13.8	70.8	1.19	5.59	227.7	1	1	52918	2	0
65	10.8	100	1	5.43	128	1	2	8.4	2	0
73	14.7	64.1	1.26	8.62	187	1	1	3.3	2	1
78	11.3	100	0.97	5.92	133.8	1	2	26.5	1	1
80	11.8	84	1.09	3.68	49.4	1	2	314.4	1	1
62	12.5	82.8	1.08	4.73	165.9	1	1	>2000.0	1	0
72	11.3	97	0.97	6.77	201.1	1	2	1556	2	0
67	13.5	73.3	1.16	4.5	116.2	1	1	21.3	1	1
54.5	12.5	82.8	1.08	1.57	43	1	3	22	1	1
73	14.7	64.1	1.26	3.41	37.5	0	1	27	2	0
67	20.5	44	1.79	2.17	18.7	1	2	32.9	1	1
78	11.7	93	1.01	5.71	119.3	1	2	83.2	2	1
85	16.2	58	1.38	3.72	43	1	2	27.5	2	1
66	10.7	107	0.93	5.37	82	1	1	52.1	2	0
80	11.5	95	0.99	5.91	163.8	1	1	24.3	1	1
49	13.5	75	1.16	3.09	13.4	1	1	675	1	1
64	12.5	82.8	1.08	3.5	158.7	1	2	261.2	1	0
93	13	82	1.12	3.35	49.1	1	1	3.9	1	0
57	10.1	118	0.88	6.64	152.8	1	2	2.8	1	1
41	14.3	72	1.23	2.31	63	1	3	2.9	1	1
60	14.9	68	1.28	4.06	87.2	1	1	584.6	2	1
57	12.9	83	1.11	6.77	139.1	1	1	>80000.	2	0
66	11.9	89.4	1.03	5.35	102.3	1	1	132.4	2	1
76	14.7	64.1	1.26	9.72	98.6	1	3	1.2	1	1
70	12.1	85	1.12	3.2	66	1	2	1.9	1	1
53	11.5	92	1.06	5.2	209	1	2	10.9	1	1
79	11.5	88	1.06	6.23	176.9	1	2	5.2	2	1
75	12.5	77	1.15	9.17	26.3	1	3	11.4	1	1
56	14.1	68.5	1.21	3.74	58.6	1	1	74.6	1	1
79.1	13.4	77	1.17	1.42	30.2	1	3	24.5	2	1
53	10.7	98	0.99	3.64	96.1	1	2	2.8	2	1
67	13.3	75.1	1.15	1.77	31.4	1	1	6.1	2	1
86	10.3	106	0.95	9.33	218.6	1	1	1002	1	0
77	12.7	75	1.17	5.98	172.5	1	2	110.1	1	0
76	11.3	97	0.97	5.92	168.2	1	2	70.5	1	0
61	11.5	88	1.06	3.22	73.8	1	1	9.1	1	1
65	12.3	84.9	1.06	3.57	111.3	1	1	21.9	2	1
55	15.3	60.2	1.32	3.01	39.6	1	1	244.7	1	0
84	13.5	68	1.24	5.51	97.3	1	2	6.9	1	1
62.8	13.4	74.2	1.15	2.7	58	1	3	10.1	1	1
95	10.2	108	0.95	7.57	85	1	2	4	1	0

72	10.9	95	1.01	7.03	181.8	1	2	89.8	2	1
78	14.6	64.8	1.26	3.03	51	1	1	90.6	1	1
72	10.4	103	0.96	5.8	139.3	1	2	15.5	1	1
66	16	56.1	1.38	3.75	48.9	1	1	64.6	1	0
81	13	73	1.19	8.46	110.1	1	2	18.2	1	0
80	12.7	75	1.17	4	90.9	1	3	4.9	1	1
76	12.6	81.7	1.09	3.57	58.2	1	2	5	1	1
66	13	82	1.13	1.86	19.4	1	3	17.4	1	1
68	10.7	101	0.99	3.16	85	1	2	960.6	1	0
74	12.2	86	1.05	5.03	183.8	1	2	102.6	1	1
78	14.2	67.7	1.22	1.86	48.4	1	1	4	1	1
72	13	77.8	1.12	6.43	129.7	1	1	31.1	2	1
87	12.8	79.7	1.1	5.87	146.3	1	1	4.6	2	1
67	11.5	87	1.06	4.64	94.4	1	2	177.8	1	1
75	12.1	87.1	1.04	11	234.6	1	2	54.3	2	1
60	11.6	93.1	1	9.18	110	1	2	2.4	2	1
74	12.3	84.9	1.06	5.87	83	1	2	2.8	2	1
58	12.7	80.7	1.09	4.81	113.9	1	1	8.3	2	1
62	16.4	56	1.52	3.3	66	1	2	3451	1	0
65	11	95	1.02	2.42	93.7	1	1	3277	2	1
51	12.1	85	1.12	6.22	156.4	1	1	891.7	1	1
77	10.9	96	1.01	7.92	139	1	1	216	1	1
55	11	95	1.02	8.14	284.4	1	1	4	1	0
98	10.6	100	0.98	6.01	181.7	1	2	2.4	1	0
85	10.6	100	0.98	5.64	129.7	1	2	3.8	1	0
71	12.7	80.7	1.09	8.02	120	1	2	2.1	1	0
75	12.2	90	1.06	3.58	54	1	3	6.8	1	1
72	10.7	99	0.99	7.22	105.1	1	2	5.3	1	1
59	10.7	99	0.99	5.96	199.3	1	3	2.7	2	1
68	13.2	76	1.14	4.34	71.8	1	1	40.8	1	1
66	13.7	67	1.26	6.29	70	1	2	1893	1	1
60	12	82	1.11	2.92	69.3	1	2	15.6	2	1
52	13.3	70	1.23	2.62	41.8	1	1	460.3	1	0
66.2	14.8	63.5	1.27	4	35.4	0	1	7.1	2	1
71	10.9	95	1.01	3.71	139.4	0	3	195.8	2	1
48	12.2	84	1.13	3.4	65	0	3	3.5	1	1
67	10.7	99	0.99	3.65	146.8	0	1	35	1	0
92	11.5	87	1.06	5.98	160.5	0	1	10.1	1	1
59	12.1	87.1	1.04	5.36	75.3	0	1	10	1	1
77	12	82	1.11	3.75	60.4	0	1	552.8	2	1
56	10.4	104	0.96	4.1	127.4	0	2	3.2	2	1
63.2	13	77.8	1.12	2.97	94	0	1	753.1	1	1
72.4	12.8	79.7	1.1	2.36	112	0	1	>2000.(	2	0
77	11.4	89	1.05	5.77	140.2	0	1	191.6	1	1

68	12.1	87.1	1.04	3.22	53.3	0	2	5.3	1	1
58	13.3	70	1.23	2.28	65	0	1	>2000.0	1	1
67	12.2	80	1.13	3.17	62.9	0	2	15.9	2	1
63	11.9	83	1.1	3.73	55.6	0	2	8.2	2	1
79	12.6	81.7	1.09	2	42.4	0	3	45.6	2	1
59.4	12.7	80.7	1.09	2.66	83	0	2	773.2	2	1
70	11.9	83	1.1	6.46	108.4	0	3	1986	2	0
62	12	82	1.11	4.95	107.5	0	1	21.5	2	1
72	12.6	81.7	1.09	5.58	103.4	0	1	5.3	2	1
79	12.2	80	1.13	5.81	126	0	1	193.6	2	1
68	12.3	79	1.14	9.1	122.4	0	2	141.2	2	1
69	12.3	84.9	1.06	5.95	127.2	0	3	173.7	1	0
52	11.5	88	1.06	4.57	75.2	0	2	>2000.0	2	1
69.7	12.2	91	1.06	4.27	102.9	0	2	3	1	1
63	13.7	71.7	1.18	9.27	253	0	1	152.6	2	0
67	12.3	79	1.14	6.18	193	0	1	20.9	2	1
49	11.8	84	1.09	2.49	63.3	0	2	239.9	2	1
103	13.9	65	1.28	8.11	181.8	0	1	38.6	2	1
73	13.6	68	1.25	3.02	61.7	0	1	36.7	2	1
105	13	89	1.15	6.67	130	0	2	3.2	1	0
61.7	15.1	61.5	1.3	2.44	38.4	0	2	152.8	1	1
44	14.8	60	1.36	4.72	68.5	0	1	3.5	1	0
66	12.2	84	1.13	2.38	148	0	2	5.9	2	1
54	10.9	99	1.01	6.4	148	0	2	5.2	2	1
89	11.7	91.8	1.01	5.43	174.5	0	2	21.3	1	0
56	12.7	85	1.11	3.99	73	0	2	2	1	0
60	10.6	101	0.98	4.55	196.1	0	2	528.2	1	0
55	11.5	92	1.06	4.03	181.5	0	3	5.3	2	1
62	10.9	99	1.01	4.81	121.2	0	2	787.2	1	1
47	13.6	72.5	1.17	2.78	82	0	1	3493	2	1
75	11.8	90.6	1.02	5.23	146.4	0	3	2.4	1	0
63	11.9	87	1.1	3.16	114	0	1	29.3	1	0
60	11	97	1.02	4.65	193.5	0	1	>2000.0	1	0
57.9	11.7	91.8	1.01	5.81	84	0	2	11.5	1	1
50	16.3	56	1.51	2.12	35.4	0	2	1052	2	1
46	13.8	70	1.28	2.79	41.6	0	3	165.9	1	0
60	12.9	77	1.19	5.73	198.3	0	1	4.4	1	1
54	13.2	75	1.22	7.37	61.4	0	1	11.8	2	1
70	13.1	76.9	1.13	3.59	77	0	1	239.5	2	1
77	12.2	86	1.05	4.7	99	0	1	4.5	2	1
64.7	12.2	86	1.05	6.97	196	0	2	6.4	2	1
52.7	13.6	72.5	1.17	5.45	82	0	2	60.3	2	1
75	12.7	80	1.18	4.09	167	0	1	69.7	1	0
66	14.8	63.5	1.27	2.02	67	0	1	48.8	1	0

63	14.2	68	1.31	3.47	117.4	0	2	1.6	1	1
90	12.8	78	1.19	3.76	66	0	2	9.6	1	0
66	11.8	88	1.09	6.38	108	0	2	>2000.0	1	0
56.6	11.8	90.6	1.02	5.33	141	0	2	19.2	1	1
75	11.6	90	1.07	3.95	92.3	0	1	1714	2	1
74	12	86	1.11	4.14	189	0	2	538.6	1	0
73	11.2	95	1.04	10.51	125.6	0	3	1241	2	1
62	12.3	83	1.14	5.81	179.5	0	1	15.3	2	1
64	14.4	66.2	1.24	3.6	112	0	1	249.5	2	1
77	11.7	89	1.08	6.38	178.3	0	1	5.3	1	0
65	13.4	83	1.24	6.96	134	0	2	6.2	1	1
53	13.7	73	1.27	2.2	28.9	0	2	689.4	2	1
58	12	86	1.11	2.17	95.7	0	2	5.7	1	0
124	11.3	103	0.98	6.31	148.2	0	2	2	2	1
61	10.7	101	0.99	5.19	117.5	0	2	1167	1	1
67	11.6	90	1.07	4.65	192	0	1	2.4	1	1
47	14	74	1.22	3.43	42	0	2	4.8	2	1
150.6	13	74.2	1.15	4.09	129	0	3	3.5	1	1
75	12.6	80	1.17	5.7	112	0	1	>2000.0	2	0
63	12	88.3	1.03	3.42	152	0	1	8.6	2	1
77	11.5	91	1.06	3.7	69	0	3	260.8	1	0
73.5	11.7	91.8	1.01	4.56	141	0	2	114.4	2	1
47	13.2	75	1.22	3.28	63.4	0	1	2.6	1	1
62	12.6	85	1.1	5.79	109.9	0	2	122.2	1	0
68	11.1	96	1.03	6.95	150	0	3	236.5	2	0
74.8	17.5	48.7	1.5	5.31	128	0	1	88.5	1	1
67	11.9	87	1.1	5	66	0	2	3.8	1	1
50	12.5	82	1.16	5.06	94	0	1	17	1	1
81	12	86	1.11	5.1	45	0	3	11.1	1	1
73.9	11.9	89.4	1.03	4.5	168	0	3	2.9	1	0
60	13	76	1.2	2.21	67	0	2	34.5	1	0
79	14.6	66	1.35	3.01	37	0	3	45.3	1	1
99	13	62.1	1.29	4	69	0	3	348	2	1
68	11.8	88	1.09	5.6	164	0	1	4.3	1	1
54	16.6	55	1.54	3.5	52	0	2	7.5	1	1
53	13.7	71	1.27	4.8	53	0	1	3422	1	1
90	12.8	78	1.19	5.24	151.6	0	1	539.6	1	1
46	12.2	86	1.05	6.2	156	0	2	349.6	1	0
106	10.8	100	1	6.2	125	0	2	158	1	1
61	11.5	94.4	0.99	5.61	113.6	0	1	4.8	1	0
71	12.4	82	1.15	3.68	63.2	0	1	8	2	1
68	10.3	108	0.95	5.7	189	0	1	4.3	1	1
57	10.5	104	0.97	6.9	275.1	0	2	16.6	1	1
82	10.9	99	1.01	9.68	156	0	1	74.3	1	1

36.4	16.1	55.6	1.38	3.08	48.4	0	1	47.3	2	0
72	13.2	75	1.22	6.1	245	0	1	47.4	2	1
66	12.1	86	1.12	1.86	38.1	0	2	47.9	1	1
69	10.3	108	0.95	6.3	118	0	1	12.2	2	1
77	13	73.3	1.16	3.35	40.8	0	1	104.3	1	1
42.9	11.7	89	1.08	3.5	95	0	1	>2000.0	1	1
65	11.4	95.7	0.98	5.3	150.2	0	1	>2000.0	1	0
75	10.4	106	0.96	5.41	117.9	0	1	27.5	2	1
46	12.8	78	1.19	2.4	59	0	1	176.9	2	1
75	11.5	91	1.06	5	169	0	2	133.8	1	1
73	10.9	99	1.01	4.77	153.7	0	3	5.6	2	1
52.4	15.6	58.4	1.34	3.41	42.4	0	2	4.1	1	1
69	11.7	89	1.08	5.3	90	0	1	>2000.0	1	0
60	12.7	90	1.18	3.46	33.4	0	2	3	1	1
65	15.3	61	1.42	4.46	98	0	1	30.4	1	1
76.9	12.6	81.7	1.09	5.88	172	0	1	29.9	1	0
44	11.2	95	1.04	6.56	112	0	2	51	1	1
72.3	12.4	83.8	1.07	8.9	202	0	2	3	1	0
67	11.2	95	1.04	5	106	0	2	92	1	1
51	11.4	92	1.06	4.01	109.2	0	1	1548	2	1
70	11.7	89	1.08	3.5	254	0	2	2.8	1	1
54	10.9	99	1.01	6.13	116.2	0	1	5.6	1	1
102	10.2	109	0.94	7.92	214.5	0	2	2	1	0
70	11.6	93.1	1	3.36	166.1	0	2	2	1	1
76	12.6	80	1.17	1.95	60	0	1	100.7	1	0
76	10.8	100	1	9.74	167	0	2	46.5	2	1
72	13	76	1.2	3.32	83	0	2	722.9	2	1
63	14	69.3	1.21	2.95	69	0	1	99.5	2	1
65	12.2	84	1.13	3.8	93	0	2	3	2	0
65	10.3	108	0.95	5.2	220	0	2	233	1	1
81	11.9	87	1.1	7.35	129.8	0	1	18.9	1	1
63	12	86	1.11	4.45	128.5	0	1	742.4	1	1
81	11.1	99.8	0.96	7.71	174.8	0	3	1.5	1	0
59	11.7	89	1.08	6.98	189	0	2	1.6	1	1
60	11.6	93.1	1	15.15	251	0	1	4.8	1	1
81	11.9	87	1.1	4.27	137.7	0	1	3.2	1	0
41	12.6	80	1.17	5.03	117	0	3	1.8	2	1
67	11.2	98.4	0.97	5.35	140	0	2	18.3	1	1
67.4	14.9	63	1.38	2.28	80	0	1	1.8	1	1
53	13.7	76	1.19	2.15	82	0	2	22.7	2	1
60	11	97	1.02	4.24	168	0	2	189.9	1	1
82	11.4	92	1.06	9.5	156	0	2	2.3	2	1
67	11	97	1.02	6.9	168	0	3	47.7	1	1
59	11	101.2	0.95	4.56	159	0	1	6.1	1	0

72	11.3	93	1.05	4.54	124	0	1	255.6	2	1
76	10.9	102.7	0.94	4.14	179	0	2	2.5	1	1
73	13.8	70	1.28	7.43	71	0	2	3.7	1	1
63	11.4	92	1.06	8.61	76	0	1	13	2	1
81	17.9	52	1.66	3.41	60	0	1	202.7	1	1
103	13.4	73	1.24	2.38	57	0	2	151.8	2	1
70	15.8	59	1.46	3.16	49	0	3	2.4	1	0
46	13.2	75	1.22	2.09	67.6	0	1	12839	1	0
81	12.4	82	1.15	6	75	0	3	1.7	1	1
73	12.2	84	1.13	4.9	99	0	2	5.6	1	0
62	12.4	89	1.15	4	103	0	2	33.8	1	1
49.6	12.7	79	1.18	3.7	62.4	0	2	357.7	2	0
62	12.2	91	1.13	2.6	72	0	1	238	1	1
62	13.3	80	1.23	2.66	113	0	2	4.5	1	0
45	11.2	103	1.04	5.21	129	0	2	10.6	1	0
62	10.6	103	0.98	2.82	53.6	0	1	15.6	1	1
68	12.2	84	1.13	2.85	90.8	0	2	21.7	1	1
75	12.1	85	1.12	3.15	69.5	0	2	606.8	1	1
53	16.2	57	1.49	2.7	55	0	2	5.1	1	1
84	11.6	98	1.07	4.32	94.8	0	2	3.2	1	1
65	11.9	87	1.1	6.49	128.7	0	2	346	1	1
66	12.9	78	1.19	5.03	111	0	1	32.1	2	1
72	10.7	113	0.93	3.25	153	0	2	163.4	1	0
41	13.2	75	1.22	2.71	77.8	0	2	1.7	2	1
60	15.5	61	1.43	2.68	41	0	2	49	1	1
42	11.4	92	1.05	3.74	48	0	3	2377	2	0
48	11.1	96	1.03	3.56	125.7	0	3	3.3	1	0
76	11.4	100	1.06	5.9	148	0	2	28	2	1
78	10.7	101	0.99	8.97	140	0	2	4.3	2	1
121	10.6	103	0.98	5.97	182	0	1	1.9	1	1
45	14.1	69	1.3	4.38	54	0	3	8.5	1	0
53	10.4	116	0.96	4.48	114	0	2	3.6	1	1
94	11.3	102	1.05	10.09	229	0	2	3617	1	1
86	11.8	88	1.09	3.98	64	0	2	3	2	1
74	10	112	0.93	5.12	154	0	3	108.5	1	0
67	13.6	78	1.26	2.84	91	0	1	11.8	2	1
57	11.1	96	1.03	7.26	126.8	0	2	426.4	1	1
124	13.5	73	1.25	6.8	105	2	3	2.3	2	1
49	14.6	66	1.35	5.08	221.9	2	1	>2000.0	1	0
49	14	69	1.29	3.22	67.9	2	2	11.7	1	0
86.3	11.1	105	1.03	8.73	127	2	3	2.3	1	1
64	11.8	88	1.09	5.25	144	2	2	406	1	1
63	10.5	104	0.97	4.15	48	2	1	5.3	2	1
74	11.1	96	1.03	7.76	204	2	3	36.6	1	1

79.1	10.4	106	0.96	8.65	199	2	2	3.4	1	1
69	12.1	85	1.12	4.95	165	2	2	1.9	1	0
88	12.6	87	1.17	3.86	51	2	1	159.2	2	1
72	11.6	98	1.07	5.05	86.3	2	2	2.5	2	1
58	11.6	90	1.07	6.79	143.7	2	2	0.9	1	1
61	13.3	74	1.23	3.24	123	2	1	>2000.(	2	1
81	14.5	71	1.34	2.49	62	2	1	15.7	1	1
79	10.6	112	0.98	6.42	136	2	2	22	1	1
78	11.3	102	1.05	6.98	138.5	2	2	306.4	1	1
87	11.5	91	1.06	3.8	165	2	2	3.8	1	1
77	11.8	88	1.09	7.3	129	2	3	11.4	1	1
59	12.9	84	1.19	4.83	86.7	2	2	1.7	1	1
82	13.3	80	1.23	6.9	115	2	1	294.9	2	1
66	11	106	1.02	7.43	248	2	2	1.8	2	1
78.2	10.9	99	1.01	6.74	146	2	2	2.3	1	0
84	13.2	81	1.22	4.2	135	2	1	470.9	1	1
55	16	62	1.48	2.07	51	2	1	4.1	1	0
69	13.2	75	1.22	3.69	128	2	1	341	2	1
58	14.8	64	1.37	3.71	111	2	1	>2000.(	2	1
62	11.5	91	1.06	8.11	244	2	1	>2000.(	1	0
87	15.4	64	1.34	5.9	73	2	2	13.7	1	0
71	12.2	91	1.06	5.2	158	2	1	13.8	1	1
60	13.1	81	1.14	3.66	79	2	3	7.6	2	1
51	12.6	86	1.1	5.81	217	2	2	42.9	1	1
69	13.9	75	1.21	4.56	84.2	2	3	12.6	2	1
52	11.4	101	0.99	4.99	139	2	1	160.2	1	1
68	18.6	50	1.63	3	37	2	3	10.7	1	1
77	11.9	95	1.04	5.4	180	2	1	73.5	1	0
66	16.1	60	1.4	2.85	46.6	2	2	10.1	1	0
74	12	93	1.04	5.12	64	2	1	9351	1	1
67	11.8	96	1.03	4.4	68	2	3	2.3	1	1
65	18.9	49	1.65	2.94	78	0	1	7.5	1	1
56.3	14.6	73	1.35	3.03	81	0	1	2.7	1	1
53	14.5	70	1.26	3.8	112	0	1	5.3	1	1
86	12.4	89	1.08	4.41	84	0	1	6.8	2	1
80	11.5	100	1	7.14	258	0	2	346.6	2	1
88	12.6	86	1.1	4.93	91.7	0	2	55.4	1	0
96	11.3	108	1.05	11.29	264	0	2	4.6	1	1
72	12.4	93	1.15	2.69	111	0	2	2.5	2	1
56.7	16.2	63	1.5	5.33	161	0	1	111.3	2	1
66	13.6	81	1.26	2.63	49	0	2	5.8	1	1
73	11.9	99	1.1	4.58	95.5	0	2	2.6	1	1
58	14.6	69	1.27	3.83	70	0	2	8.5	2	1
58	12.3	90	1.07	4.79	114.9	0	1	18	2	1

74	11.5	105	1.06	3.98	115	0	2	19.3	2	1
61	15.7	63	1.37	7.23	183	0	1	564.2	1	0
62	11.2	110	1.04	5.49	139.2	0	1	4.8	2	1
46.6	14	78	1.3	3.6	92	0	1	487.8	2	1
68	11.6	103	1.07	8.06	164	0	2	2.7	2	1
76	9.9	135	0.92	7.23	270	0	2	2.9	1	1
64	12.8	84	1.11	6.35	180	0	2	392.5	2	1
95	13.1	81	1.14	3.43	43	0	1	114.5	1	1
68.2	11.6	98	1.07	6.9	221	0	2	34698	2	0
51	13.4	79	1.17	5.69	39.1	0	3	4.3	1	0
49	13	82	1.13	4.3	134.6	0	1	1.8	1	1
56.3	11.8	95	1.09	3.02	97	0	3	17.5	1	1
56	11.7	96	1.08	4.73	104.7	0	2	5.5	2	1
59	12.5	86	1.09	5.27	68.5	0	1	86.3	2	1
75	13.1	80	1.21	4.43	110.4	0	1	98.1	1	1
57	14.3	69	1.25	1.96	38	0	1	13.1	1	1
72	12.2	90	1.06	2.74	79.7	0	1	62.4	2	1
67	11.3	103	0.98	5.13	177	0	1	978.2	1	0
185	11.6	98	1.07	5.04	89	0	3	3.5	1	1
74	11.9	94	1.04	5.2	190	0	1	158.6	2	1
84	12.6	85	1.1	4.96	88.7	0	2	74.4	1	1
64	11	109	0.96	3.09	123.1	0	3	>2000.0	1	1
64	13.6	76	1.26	4.15	108.2	0	3	37.6	1	1
69.6	13.3	78	1.23	8.58	108.4	0	2	14603	1	0
62	10.7	112	0.99	3.9	138	0	3	2.8	1	1
85	11.7	96	1.08	4.98	107	0	2	666.9	2	1
93.1	12.7	84	1.18	5.77	152.7	0	2	42.9	1	1
57.2	16.3	56	1.42	2.32	48	0	1	138.5	2	1
70.9	11.5	100	1	4.14	121	0	2	1.5	1	1
43.9	10.8	113	0.94	5.72	97.8	0	2	317.5	2	1
53.7	13.3	78	1.23	2.84	102	0	3	25053	1	0
64	12.3	98	0.95	6.6	167.7	0	2	57.7	1	0
90.6	12.1	91	1.12	3.81	143.7	0	1	>2000.0	2	0
65.9	15.7	63	1.36	4.14	60	0	1	5.6	1	1
96.4	15.5	62	1.44	3.86	142	0	2	>80000.	2	0
66.8	13.9	68	1.28	4.68	550.1	0	1	29.7	2	0
57.3	14.9	71	1.29	3.28	72.3	0	1	89.4	2	1
67.6	9.8	150	0.91	5.79	163	0	3	1241	1	0
46.4	16.2	65	1.5	3.81	80	0	1	159.1	2	0
63.4	15.9	59	1.47	26.47	185	0	2	1.4	2	0
70	13.1	76.9	1.13	4.66	109.7	0	1	392.5	2	1
76	11.9	92	1.03	2.91	65.7	0	2	5.6	1	1
68	12.4	78	1.14	5.51	146.7	0	3	74371	2	0
74	13.6	80	1.18	6.77	133.9	0	3	4	2	1

68	11.6	110	0.9	5.39	259	0	2	11.1	2	1
69.6	13	81.7	1.09	5.52	181	0	2	3.9	2	1
65	12.9	83	1	7.8	210.3	0	2	19.5	1	1
63.5	12.4	82.2	1.02	11.41	166	0	1	>350.0	2	1
87	14.6	68	1.26	3.54	53	0	2	96.2	2	1
64	11.4	98	0.88	4.62	134.9	0	3	5.5	2	1
66.4	11.4	102	0.99	7.72	193.1	0	2	2.4	1	1
55	14.6	70	1.1	3.03	63.6	0	2	494.9	1	1
80	10.7	111	0.86	5.69	188.4	2	3	21.2	1	0
44	11.9	95	1.03	4.24	59.2	2	2	49.1	2	1
76	15.9	63	1.47	6.88	109	2	2	>2000.0	1	0
73	14.6	70	1.1	3.87	86.6	2	1	20.8	1	0
57.6	19.4	41.1	1.56	3.02	30.2	2	2	264.6	2	1
82	12.2	86	1.05	5.59	187	2	2	1071	1	1
70	10.5	103	0.97	7.93	170.2	2	1	>2000.0	1	0
82.8	11.1	107	1.03	6.54	132	2	2	7.4	2	1
56	13.5	73.3	1.16	3.31	61.6	2	2	9.2	2	1
60	12.5	87	0.98	1.12	21.1	2	2	132.3	1	1
64	14.2	72	1.24	2.37	33.2	2	1	27.4	2	1
87	11.8	88	1.09	4.72	149.4	2	2	2.3	1	1
72	12.9	83	1	2.42	43.4	2	2	225.1	1	1
66.4	16	55.5	1.3	3.47	63	2	2	5.6	1	1
59	11.8	95	0.93	3.9	119.5	2	1	4.7	2	1
70	12.1	85.4	0.99	5.14	207.1	2	2	501.6	1	1
58	11.7	89	0.91	4.78	97.9	2	1	186.5	2	0
67	12.1	101	0.94	5.2	35.5	2	2	3.8	2	1
59	13.8	70.8	1.19	3.63	140.4	2	2	1.8	1	1
63	14.3	67.5	1.23	2.06	48.7	2	3	122	1	0
46.1	11.1	103	0.96	5.33	151	2	3	1905	2	1
52	11.7	108	0.91	5.28	193.1	2	2	6542	1	1
72	14.4	75	1.12	2.83	47.5	2	2	3.5	1	1
58	10.7	107	0.83	5.29	194.2	2	2	3.3	1	1
63	11.8	106	0.91	7.63	169	2	2	16.1	1	1
95	10.5	102	0.97	5.93	114.8	2	1	2	1	1
54.2	13.8	69.6	1.13	3.02	64	2	2	142.6	1	0
66	13	67	1.01	4.44	93.2	2	1	149.5	2	1
71	11.9	94	0.94	6.42	220	2	2	131	2	0
64	12.4	97	0.96	7.83	150.4	2	1	>350.0	2	0
70	11.7	89	1.08	5.67	181	2	2	1.8	1	1
86.4	13.3	73.7	1.09	5.8	112	0	1	12.8	1	1
77	16.2	61	1.2	2.53	60.7	0	1	43.8	1	1
50	12.9	83	1.11	3.42	90	0	2	4.3	1	1
65	12.2	100	0.95	4.21	148.9	0	2	29.8	2	1
77	11.9	94	0.94	3.7	154.5	0	2	2.1	1	1

65	11.9	83	1.1	3.94	123.1	0	2	1.6	2	0
49	11.2	102	0.96	9.15	267.1	0	2	6.1	1	1
69	12	82	1.11	4.82	88.6	0	2	31795	2	1
62	13.1	75.5	1.07	6.62	59.6	0	1	115.4	1	1
70	12.4	89	1.15	4.1	156	0	1	640.1	2	1
79	12.9	78.8	1.11	3.41	36.6	0	2	7	2	1
82	11.3	93	1.05	4.3	195	0	2	33	1	0
82	12.3	90	0.96	5.69	130.5	0	1	7393	2	1
52	12.1	85	1.12	6.2	125	0	2	3	2	0
73	12.7	85	0.99	2.49	92.1	0	2	123.2	1	1
82	12.8	81.1	1.1	6.79	131.9	0	2	4.6	1	1
65	12.8	78.3	1.05	5.43	139.7	0	2	302.7	1	1
90	10.7	101	0.99	7.15	260	0	1	8.5	1	1
66	12.4	83.8	1.07	5.68	67.3	0	1	279.7	1	0
112	13	72	1.2	1.73	36.8	0	2	1.6	1	0
84.2	14.1	67.7	1.21	3.72	109	0	2	42.6	1	0
210.1	24.8	28.1	1.98	5.86	120.6	0	3	>350.0	1	1
64	12.9	73	1.19	5.66	264	0	2	441.5	2	1
107	11.7	77	1.01	5.45	83	0	2	2.7	1	1
42.6	16.4	52	1.51	3.57	46.4	0	1	2239	2	1
73	13.4	72.9	1.09	2.07	68.9	2	3	3.4	2	1
84	12.3	88	1.06	3.66	157.1	2	2	2485	2	1
69.9	14.9	67	1.23	2.08	33.4	2	2	2.8	2	1
52	12.2	89	1.05	8.84	242.7	2	2	172.8	2	1
62	11.6	90	1.07	4.3	111	2	2	5.1	2	1
65.4	12.1	97	1.12	4.6	231	2	2	2.1	1	1
99	12.6	80	1.17	5.1	81	2	1	52.1	1	1
79	10.5	104	0.97	4.35	133	2	2	>2000.0	2	1
59.7	21.1	35.4	1.81	1.19	42.9	2	2	4.1	2	1
70	12.3	98	0.95	4.83	67.4	2	2	5.3	2	1
38	13.8	70	1.07	2.01	49.4	2	2	34.5	1	1
49	12.9	78.8	1.11	6.99	253	2	2	2.7	1	1
91	10.9	99	1.01	6.3	240	2	2	169.5	1	1
62	11.8	94	1.02	7.15	262.7	2	2	2	2	1
71	11.4	99	0.98	5.78	159.3	2	2	1.9	2	1
60	10.5	115	0.85	3.6	79.6	2	1	2592	2	1
61	12.1	97	1.12	4.66	149	2	3	7.1	1	1
79	16	60	1.48	2.02	54	2	2	2.4	1	1
73	11.4	99	0.98	3.6	59.3	2	2	3.2	1	1
75	13.2	76	1.14	1.93	57.7	2	2	2.1	2	1
98	10.5	104	0.97	5.21	197	2	2	1.1	1	1
46	13.2	87	1.02	2.41	50.6	2	2	5.4	1	0
77	11.2	104	0.89	5.41	192.5	2	2	2.7	2	1
56	15.1	60.8	1.23	4.21	59.6	2	2	315.6	1	0

76	12	103	0.94	4.32	107.9	1	2	23	2	1
69	12.3	90	1.07	4.9	91	2	1	10.4	1	1
65	11.9	95	1.04	5.75	90.9	2	2	1.8	2	1
54	15	62.3	1.29	1.72	52.9	2	2	8	1	1
67	12.5	86	1.08	2.75	44.1	2	2	240.8	1	1
69	11	85	0.97	5.2	107.9	2	2	5	1	1
64	11.8	88	1.09	5.9	159.3	2	2	1.6	1	1
60	14.3	72	1.09	1.94	60	2	2	3.8	1	1
76	14.3	63	1.32	2.35	49.4	2	2	1.4	1	0
57	13.6	82	1.05	3.06	43.8	2	2	88.9	1	1
68	14	68	1.09	5.63	150.1	2	2	>350.0	2	0
62	16.1	57	1.49	1.32	32.4	2	1	9.6	1	0
159	11.1	96	1.03	4.66	110	2	2	145.4	1	1
58	11.7	93	1.01	3.43	160.7	2	2	83.1	2	1
56	12.1	87	0.94	4.05	116.2	2	2	6.4	2	1
71	13.6	77	1.04	2.36	46.4	2	1	32	1	1
79	13.7	76	1.19	2.43	64.7	2	2	60.4	1	1
104	12.2	91	0.96	2.32	103.3	2	2	5	1	1
65.4	13.4	74	1.04	3.87	78.4	2	2	3.2	1	1
57	12.7	93	0.99	3.86	72.9	2	2	802.9	1	1
63	11.7	89	1.08	5.43	114.6	2	2	4.3	2	1
84	11.7	85	1.08	4.23	142	2	2	7.1	1	0
63	12.6	81.7	1.09	7.52	166	2	2	1	1	1
50	12.5	72	0.97	2.72	88.7	2	2	>350.0	1	1
79	16.4	54	1.41	2.13	48	2	2	3.5	2	1
102	12.2	89	1.05	4.28	165.9	2	2	3.9	1	1
51	12.8	78	1.18	2.9	72	2	2	4.2	1	1
78	13.9	79	1.06	1.71	42.1	2	2	2.5	1	1
45	12.1	88	0.94	3.74	156.9	2	3	177.4	1	1
61.5	17.6	49.4	1.51	2.61	69.1	2	2	2.2	1	0
68	13.2	87	1.02	5.18	147.3	2	1	3.5	1	0
125	13.4	63	1.13	1.62	34.9	2	2	4.2	1	1
101	10.6	101	0.98	3.81	89.4	2	2	9.4	2	1
55	15.5	59	1.33	5.02	81	2	3	130.8	2	1
66.6	13.1	76.9	1.13	2.47	49.4	2	2	29.4	1	0
70	13.4	85	1.03	2.26	34	2	2	200.2	1	1
79	10.8	110	0.87	3.2	62.9	1	2	94.6	1	0
72	11	105	0.95	5.82	99.7	2	2	22.2	2	1
67	12.7	82	1.09	4.92	102.3	2	1	4	1	1
78	14.3	76	1.09	1.67	34.7	2	2	10.6	1	1
79.6	14	69.3	1.21	3.17	59	2	2	22	1	0
81.7	14.9	62.1	1.21	4.7	62	2	2	4.4	2	1
56	14.6	67	1.27	2.52	60	2	2	3.1	1	0
70	15.5	58.2	1.26	4.6	84	2	1	315.7	1	1

70	12.4	97	0.96	2.11	45.7	2	2	42.8	2	1
83.4	19	42.3	1.53	6.6	49	2	1	7.2	1	1
81	13.3	73.6	1.15	2.83	56.3	2	1	9.2	2	1
54.5	18.4	44.6	1.58	2.02	47.2	2	2	2.8	1	1
53.1	14.1	68.5	1.21	5.03	123.2	2	2	17.6	1	1
57	16.3	53.8	1.32	3.39	101.1	2	2	6.9	1	1
56	16.4	53.8	1.41	7.65	157	2	3	69.1	2	1
46.7	19	42.1	1.63	4.38	56	2	2	23.6	1	1
137.5	18.5	44.7	1.59	2	36.4	2	1	170.6	1	1
63	17.6	54	1.28	3.65	48.4	2	2	10.9	2	1
49.3	14.8	56.6	1.19	17.61	169	2	1	>5845.0	1	1
49.9	13.7	71.7	1.18	7.59	211	2	2	13.1	1	0
75	15	62.1	1.29	2.95	39.4	0	3	1.5	1	0
59	17.8	41	1.38	5.58	72.4	0	2	12.5	1	1
60	16.6	59	1.22	5.88	72.8	0	2	6	1	1
78.7	18	46.1	1.55	4.44	44.5	0	3	8.6	2	1
60	12.4	70	1.06	4.33	107.6	0	2	172.6	2	1
63.3	13.1	78	1.13	2.94	48.2	0	1	13	2	1
63.3	14.4	65.2	1.17	4.1	67	0	1	2.9	1	1
52	12.4	78	1.14	2.55	29.7	0	3	4.6	1	1
44	16.8	56	1.56	1.56	42.4	0	2	223.7	2	1
76	15.1	61	1.17	4.56	55.4	0	3	2.2	1	1
77	14.6	63.8	1.26	4.08	62.3	0	1	44.4	1	1
87	14.6	69	1.35	2.44	46.8	0	2	1.8	1	1
101	17.5	51	1.53	5.14	110	0	3	114.3	2	0
68	14.2	69	1.31	1.96	37.4	0	2	4.1	2	1
50	11.7	89	1.08	2.31	38.2	0	2	2.2	2	1
56	12.5	77	1.15	4.33	214.6	0	1	>2000.0	2	0
67.8	11.7	97	1.02	3.88	194.6	0	3	5.8	1	0
62	12.6	81.7	1.09	2.23	33.6	0	3	1.2	1	1
46	11.7	90	1.08	3.26	50.4	0	3	27.7	2	1
71.5	14.3	67	1.23	7.73	385.8	0	1	44.7	2	1
55	12.1	85	1.12	2.27	83.9	1	1	284	2	1
68.6	11.8	88	1.09	2.94	78.4	0	2	2.2	2	1
63.4	14.1	68.5	1.21	14.7	205	0	1	32633	2	1
92	10.8	109	1	2.3	136	0	3	325.6	2	1
52.4	12.7	86	1.18	6.37	245	0	2	3.1	2	0
65	10.7	101	0.99	5.1	156	0	3	3	2	1
106	11.1	106	0.96	5.26	127	0	1	35.2	1	0
64	11.1	106	0.96	3.78	101	0	1	409.4	1	1
60	15.9	62	1.39	7.76	162	0	1	8	2	0
52	13.1	79	1.14	4	98.7	0	3	14	1	1
78.3	14.7	67	1.36	8.15	86	0	3	>2000.0	2	1
74.8	16	59	1.48	10.92	138	0	1	27.6	2	0

109	12.6	81.7	1.09	4.29	79.6	0	2	10.6	2	1
60	12.8	84	0.99	7.4	224	0	1	25	1	0
50	12.3	88	1.06	2.99	145.3	0	2	2.1	1	1
64	10.3	108	0.95	3.45	108.9	0	2	25.7	2	1
43.7	14	69.9	1.21	6.87	173	0	1	51	2	0
73	11.9	94	0.94	5.1	66.5	0	3	>1000.0	2	1
52	15.5	59	1.33	1.66	55.5	0	1	18.4	2	1
97	14.3	70	1.24	4.15	77.8	0	2	2.9	1	1
65	16.2	60	1.32	11.65	281.5	0	1	413.9	2	1
71	12.1	87.1	1.04	7.13	145.6	0	2	5.7	2	1
65	15	66	1.3	4.57	145.6	0	1	>2000.0	2	1
45	15	62.1	1.29	13.7	300	0	3	1.3	2	0
64	12.4	83	0.96	4.57	156.6	0	1	46	2	1
119.5	12.3	90	0.96	4.28	70	0	1	>1000.0	2	0
50	12.2	86	0.95	3.48	83.8	0	2	>350.0	2	1
90	13.9	68.9	1.13	7.07	162.4	0	3	41242	2	1
46	12.8	84	1.09	2.42	29.7	0	2	6.6	1	1
134.1	16.6	53	1.43	3.26	46.4	0	2	4.5	1	1
57	12.5	86	1.08	1.63	18.7	0	2	1.6	1	1
49.3	15.2	60.8	1.31	7.41	161.5	0	3	1.3	2	0
122.6	12.1	86	1.12	16.38	169.4	0	3	448.5	1	1
128	14.5	65.5	1.25	2.03	62.6	0	2	2.2	2	1
135.8	15.5	58.2	1.26	3.52	38.2	0	3	290.1	2	0
55	12.8	74	1.18	9.17	137	0	1	76.9	2	0
78	12.9	83	1	1.24	22.1	0	3	18.2	2	1
57.2	12.7	75.6	1.02	2.02	43.2	0	3	7.7	2	0
69.6	16.5	46.1	1.33	3.93	52.7	1	2	2.4	1	1
72	18.6	36.7	1.5	9.25	187.1	0	2	126.3	2	1
71.2	12.7	69	0.98	4.22	130.1	1	1	>350.0	1	0
53	12.7	75.6	1.02	9.37	159	0	1	>350.0	2	0
73.3	20.5	34	1.59	22.32	244.3	0	1	34	2	1
36	15.2	52	1.18	5.05	132.3	1	1	>58344.	2	1
63	17	44	1.32	5.05	94.8	1	1	>350.0	1	0
79	14.1	59	1.09	4.7	55	0	1	5	1	0
47.6	15.3	53.1	1.23	7.3	70	0	2	1.2	1	0
31.1	20.6	30.3	1.66	5.74	73.9	1	1	>350.0	2	1
62	15.3	59	1.19	3.44	53.4	0	1	78.2	1	0
99	13.9	69	1.08	7.99	260.8	0	2	105.1	2	1
126	15.1	60.8	1.23	4.4	118	0	3	8	1	1
109.3	13.6	66.4	1.09	6.1	138	1	1	>350.0	2	0
41	19.1	41.9	1.54	8.4	53	0	3	38.1	2	1
64.8	14.1	62	1.13	8.87	100.1	0	1	>350.0	2	1
90	17	51	1.32	2.64	51.4	0	1	>350.0	1	0
62	15.4	59	1.19	15.58	299.6	0	3	2.9	2	1

67	15.4	59	1.19	4.26	58.9	0	3	4.9	2	1
51.3	14.1	68	1.09	5.5	166.9	1	1	150.1	1	0
67.8	15.4	52.5	1.24	6.5	302	0	3	2.6	2	1
55.7	15	61.4	1.22	2.52	47.2	0	2	19.6	1	0
64.1	20.6	30.3	1.66	1.82	67.4	1	1	37	2	1
70.9	15.2	52	1.18	2.04	23.8	0	3	2.7	1	1
121	13.1	77	1.02	4.6	81.9	0	2	>350.0	1	0
295	15	55.2	1.2	2.92	106	1	1	>350.0	1	0
60	16.3	54	1.26	4.51	92.9	1	1	120	2	0
59	21.8	36	1.69	4.78	38.5	0	1	210	2	1
97.2	20.1	38.7	1.61	3.22	24.4	0	1	2.5	2	1
57	15.4	59	1.19	5.52	70.9	0	1	344	1	0
80	13.4	74	1.04	4.12	86.3	0	3	2.2	1	1
63.2	16.8	52	1.44	5.61	72	0	2	218.9	2	1
214	20.6	39	1.6	10.05	102.4	0	1	60.5	2	1
104	18.7	43.4	1.51	4.54	59.3	0	2	5	2	1
75	14.6	64	1.13	7.89	128.8	0	1	90	1	0
86	16.2	55	1.26	6.39	77.1	0	1	326.4	2	0
58	13.1	76.1	1.07	2.45	106.4	0	2	68.7	1	1
275.9	14.5	64.8	1.18	7.9	106	0	3	134.6	2	1
70	16.1	54.9	1.31	2.45	51.3	1	2	16107	2	1
59.4	18.6	45	1.44	4.2	53	1	1	114.3	2	1
673	13	77	1.06	7.3	332	0	1	10.1	1	0
44	14.6	64.1	1.19	4.03	60.3	0	1	90.4	1	1
56	16.8	45	1.3	6.19	37.1	0	1	7.9	2	1
71	15.7	57	1.18	4.54	36.7	1	1	32724	1	0
74	15.5	58.3	1.26	3.69	76.5	0	1	209.2	2	1
89.4	16.8	52	1.3	2.02	24.4	0	2	340.1	2	0
56	14.4	65	1.12	4.6	119	0	2	344.7	2	0
93.1	20.9	36.4	1.68	2.8	58	0	1	15.6	2	0
52	14.9	62.1	1.21	1.93	50.2	0	1	8.1	2	1
65	14.2	67	1.1	5.93	233.6	0	2	332.1	2	0
71.2	14.9	62.1	1.21	6.57	120.1	1	3	>350.0	2	0
66.2	16.4	53.4	1.33	4.5	49.2	0	1	50.5	2	1
71	16	55.5	1.3	3.48	62.4	1	1	13.8	2	1
69	12	85	0.93	4.61	132.6	0	2	>350.0	1	0
54	13.5	71	1.05	4.42	126.4	0	1	49384	2	1
70	15.5	58.3	1.26	2.68	38.6	0	3	342.9	1	0
64	13.8	68	1.07	4.82	58.8	0	1	>350.0	2	0
113.2	16.3	53.9	1.32	9.2	167	1	3	67	1	0
123	13.8	70.1	1.13	2.72	47.7	0	3	98.1	2	0
69	16.8	51.4	1.36	3.52	226	1	1	19.3	2	1
87	15.9	56	1.23	2.55	48.1	0	2	11.1	1	1
76	20.3	38.1	1.63	3.04	118.5	0	3	>58344.	2	0

61.5	14.8	62.8	1.2	6.42	132.1	0	2	57656	2	0
62.7	14.1	67.8	1.15	6	108	1	3	>350.0	2	0
77.8	12	87.4	0.98	10.21	131	0	3	4	2	1
54.8	13.3	74.3	1.09	6.2	75	0	1	3814	2	0
81	16	55.5	1.3	23.11	155	0	1	2078	1	0
60.1	14.7	64	1.14	5.8	87	1	1	>350.0	2	1
64.9	13.8	71.6	1.19	8.05	219	0	2	477.9	2	1
73.6	24.4	28.5	1.95	10.61	226.4	0	2	2.1	1	0
39	13.9	68	1.08	3.85	112	0	3	19.2	2	1
52	12.9	78	1	2.58	83.8	0	3	>350.0	1	0
120.8	16.3	53.8	1.32	9	106	1	1	>350.0	2	0
69	18.4	44.6	1.48	17.81	100	1	1	17.6	2	0
53	15	61.2	1.22	4.4	62	0	1	>350.0	2	1
59.2	21.4	35.3	1.72	1.9	29	1	1	4825	2	1
46.9	13.6	71.2	1.11	3.92	123	0	1	8.7	2	1
57	17	50.4	1.38	6.8	52	0	2	184.2	2	0
72.4	20	39.2	1.61	6.84	70.5	1	1	>350.0	1	0
66.3	19.6	40.4	1.58	4.8	53	0	2	295.6	2	1
64	14.9	63	1.16	7.36	153.6	0	2	>350.0	2	1
104	15.6	66	1.21	4.56	107.7	0	2	4.4	2	1
68	16	55.4	1.3	2.14	67.4	0	2	3.1	1	0
65.4	14.8	62.5	1.2	3.9	130.3	0	1	53.8	2	1
159.3	34.7	16.7	2.73	5.34	15.4	0	3	5.6	1	0
54	13.7	70.4	1.12	6.33	289.8	0	2	2.4	1	0
58.7	18.4	44.6	1.48	4.39	64.6	1	1	41.1	2	1
61	25.3	27.2	2.01	3.22	64	0	1	328.8	2	0
70	14.4	65.2	1.17	8.9	235	1	1	>350.0	2	0
66.5	16.8	51.3	1.36	6	51	0	1	14.8	2	1
165.2	16.8	51.3	1.36	3.48	49.8	0	1	4.9	2	1
54.6	13.7	70.4	1.12	7.8	112	0	3	7.6	2	1
69	15.6	57.6	1.27	2.12	37.2	0	3	294.4	1	1
101	19.9	39.5	1.6	5.72	71.8	1	1	>350.0	1	0
142.2	23.5	30.5	1.88	4.6	59	0	2	15.6	2	0
64	13.8	80	1.07	3.57	12.5	1	1	75.4	2	1
52.1	16.9	50.9	1.37	7.3	82	1	1	124.1	2	1
80	14.2	66.6	1.16	7.4	218	0	1	140.3	2	0
68.5	13.5	83	1.05	3.31	67.1	0	1	52.6	1	1
73.1	14.7	63.2	1.2	3.52	73	1	1	15.5	2	1
59.8	14.5	64.5	1.18	4.5	100	1	1	3548	2	0
76.7	15.3	59.4	1.24	5.3	165	1	1	21.8	2	1
75	16.2	54.3	1.31	3.57	47	0	2	39.6	2	1
51.8	13.6	71.2	1.11	2.82	85	0	2	4952	1	1
64.9	13.4	72.9	1.09	8.1	139.4	0	1	180.4	2	1
67	13.6	71.2	1.11	2.83	48.3	0	2	3.6	2	1

79.5	18.6	43.9	1.5	9.4	93	0	2	90.4	1	1
62	18.4	44.6	1.48	1.87	31.9	1	1	17	1	0
70.1	13.1	75.5	1.07	7.8	89	0	1	3.5	2	1
67	14	68.1	1.14	2.43	66.8	1	1	9.1	1	1
196	33.7	17.5	2.65	12.88	25.9	0	1	2.7	2	1
62.8	12.5	81.2	1.02	7.2	170	0	1	5.9	2	0
80.8	12.9	90	1	5.8	85.4	0	1	>350.0	1	0
57.6	19.4	41.1	1.56	3.02	30.2	0	2	264.6	2	1
80	17.5	56	1.36	6.4	52	0	2	>350.0	2	1
75.9	16.5	52.8	1.34	4.02	90	0	2	71.3	1	0
49.1	14.4	75	1.12	2.48	97.4	1	1	4.8	2	1
129.4	15	61.2	1.22	4.32	37.3	1	2	5	1	0
60.2	15.4	58.8	1.25	2.92	74	1	1	>350.0	1	1
58	13.1	88	1.02	6.62	159.3	1	1	>58344.	2	1
56	14.4	65.2	1.17	8	224	0	2	9.9	1	0
187	14.1	78	1.09	19.6	410.9	0	1	50.8	1	0
103.1	18	46.1	1.45	3.52	133	0	3	57.7	2	1
63	18.9	50	1.47	7.85	120.5	1	1	253.9	1	0
68	13.6	71.2	1.11	5.6	147	0	1	>350.0	2	0
61.8	13.8	69.6	1.13	7.7	145	0	1	14.7	2	0
70	12	103	0.93	4.95	99	0	3	724.8	1	1
65.7	15.9	55.9	1.29	5.9	69	0	1	4.8	2	0
81.6	17.3	49.1	1.4	9.14	141	0	2	8	2	0
83.2	12.7	93	0.98	5.3	119	0	3	>350.0	1	0
73	14.1	78	1.08	6.72	98.6	1	1	>350.0	2	0
67	14.3	76	1.09	3.59	44.5	1	1	>350.0	1	0
58	11.7	108	0.91	6.4	138	0	2	8.6	2	0
41.1	13.6	71.2	1.11	6.2	244	1	1	>350.0	2	1
68	15.5	58.2	1.26	4.81	83.3	0	2	>350.0	2	1
68.5	17.6	47.8	1.42	5.6	79	0	1	5296	2	0
76	15.4	58.8	1.25	2.9	85	1	2	5.8	1	0
75	15	61.2	1.22	7.5	228	0	1	248.1	2	0
56	20	46	1.45	3.29	67.4	0	1	47.8	2	0
53.8	18.9	42.8	1.52	8.8	46.2	0	2	>350.0	2	1
66	14.1	78	1.08	6.3	77	0	2	74	1	0
143	11.3	116	0.89	3.9	110.2	1	1	65.4	2	1
56	12.7	68	1.08	2.38	41.3	0	2	64.5	2	1
209.6	13.3	73.7	1.09	7	128	0	3	7427	2	0
104	13.3	86	1.03	7.18	80.8	0	3	3240	2	1
66.6	17.5	48.2	1.41	4.6	202	1	1	14549	2	0
54.1	13.9	68.9	1.13	6.8	60	0	1	2517	2	0
172.6	15	61.2	1.29	5.2	40.2	1	1	222.1	1	1
69	18.9	42.8	1.52	13.02	183.4	1	2	6.8	2	0
70	17.3	49.1	1.4	8.75	277.9	0	2	>58344.	2	0

55	15.7	57	1.27	1.6	30.9	0	1	31.4	2	1
71.9	18.7	43.5	1.51	9.8	163	0	1	46.1	1	0
62	14.5	74	1.1	6.31	117.2	1	1	114.1	2	0
62	14.2	66.6	1.16	6.2	148	0	1	>350.0	2	0
67	13.6	82	1.05	6.21	103.4	0	3	>350.0	2	0
154	15.7	57	1.27	2.32	35.2	0	2	229.4	2	1
84	12.9	90	1	4.61	148	0	2	>350.0	1	0
58	13	76.4	1.06	2.46	50.5	1	3	272.2	1	1
55	17	50.4	1.46	5.1	104	0	1	>350.0	2	0
52.6	15.1	60.6	1.23	3.12	124	1	1	17.1	2	0
74	15.8	63	1.18	1.72	101	1	1	>350.0	2	0
97	14.1	73	1.07	2.67	74.7	1	2	13	1	1
95	14.6	63.8	1.26	3.92	93	0	1	103.8	2	1
58	13.3	73.7	1.14	5.79	140.3	0	1	190.2	2	1
91.4	15	61.2	1.29	6.9	165	0	1	>350.0	2	0
228	19.7	47	1.4	10.14	132.9	0	1	>350.0	1	0
66	18.9	42.8	1.62	14.11	92	1	1	90.6	2	0
80	18.6	43.9	1.6	2.12	29.2	0	2	77.1	2	0
47	14.9	68	1.12	14.14	73.5	1	1	150.8	2	0
65	13.7	76	1.05	1.38	55.1	1	1	81.6	1	1
95	15.5	64	1.16	3.68	72	1	1	5.8	2	1
141	25.6	26.7	2.19	8	172	0	3	5.4	2	1
72	13.6	71.3	1.17	2.74	128.7	0	1	13.9	2	0
44.9	15.2	60	1.31	3.62	72	0	1	2252	1	1
76	12.1	92	0.95	11.3	256.2	0	2	3448	1	0
64	12.6	86	0.98	2.52	48.4	1	1	2727	2	0
80	13.1	75.2	1.13	3.03	243.5	0	2	127.5	2	0
67	13.1	75.2	1.13	7.89	145.9	0	1	20.4	1	0
100	13.2	81	1.02	3.55	57.2	1	1	2.4	1	1
168	17.5	49.8	1.5	8.47	91	0	3	>1000.0	1	0
55	12.2	91	0.96	3.13	63.3	0	2	8.6	1	1
53.1	13.3	73.6	1.15	2.16	74	0	3	>50000.	2	1
89	13	76	1.12	9.08	198	0	1	8.2	2	0
82.2	15.6	58.6	1.34	5.38	100.2	1	1	>1000.0	2	0
71.4	18	47.8	1.55	11.24	69	0	3	5.2	2	0
59.9	15.6	58.6	1.34	2.32	26.2	0	3	4.6	1	1
54	14.1	67.7	1.21	9.06	186.3	0	2	87.9	2	1
55.9	17.5	49.8	1.5	2.47	50	1	1	8561	1	0
62	14.1	67.7	1.21	3.72	131.9	0	1	>1000.0	2	0
71.6	18.4	46.4	1.58	4.66	93.9	1	1	>1000.0	1	0
68	15.6	58.6	1.34	3.92	260.2	1	1	>1000.0	2	0
93	12.6	79.5	1.09	3.52	105.4	0	1	47.3	2	1
135.9	15.8	57.6	1.36	14.2	73	1	1	>1000.0	2	0
52	14.9	68	1.12	4.18	209.9	0	3	22.1	1	1

58.2	16.4	54.6	1.41	4.59	95.3	0	2	>1000.0	2	1
67	17.5	49.8	1.5	7.4	210	0	1	220.9	2	0
64	15.5	64	1.16	2.46	40.9	1	1	2.6	2	1
47	11.6	98	0.92	4.01	88.7	0	2	290.1	1	0
70	17.2	49.7	1.48	8.02	103.2	0	1	22.3	1	0
90	14.4	71	1.09	2.01	68.2	1	2	6.4	1	1
104.1	13.5	78	1.04	11.53	106.4	0	1	10582	1	0
104.2	14.3	67.5	1.23	16.89	91	1	1	5.1	2	1
121	18.9	50	1.36	17.64	170	0	3	1.6	2	0
62	12.6	86	0.98	2.05	50.3	1	1	24.6	2	1
80.2	13.5	74.2	1.16	5.65	147.4	0	3	>1000.0	2	1
48	12.8	84	0.99	5.38	177.6	0	3	25.8	2	0
68	14.9	63	1.28	4.49	91	1	2	18.9	1	1
69	18	46.1	1.55	17.71	115.2	0	3	29	2	1
67	15.5	59	1.33	4.38	59	0	2	21.7	1	0
68	11.3	102	0.9	4.13	121.7	0	2	4.6	1	0
51	15.8	57.2	1.36	6.63	68	0	1	>1000.0	2	1
48	14.1	69.1	1.21	5.54	241.8	0	3	3.6	2	1
120	21.2	35.1	1.82	4.83	64.9	0	1	15.5	1	1
49	16	62	1.19	2.09	43	0	3	4.5	2	1
51	15.2	61	1.31	6.37	107	1	1	>1000.0	1	0
64.9	15	62.3	1.29	5.35	63	1	1	8.9	1	1
58	11.2	104	0.89	4.15	66.6	0	3	4.2	1	0
72.9	14.1	69.1	1.21	8.39	187	0	1	>1000.0	1	0
58.1	17.2	49.7	1.48	1.58	28.4	0	2	4.7	1	0
76.7	16.2	54.9	1.39	7.3	84	0	1	43.7	1	1
56	25.3	26.2	2.16	6.54	68.1	0	2	>1000.0	2	0
75	14.6	70	1.1	4.11	62	0	1	38.3	2	1
77.8	14.5	65.9	1.25	4.24	52.4	1	2	104.6	1	0
70	18.5	51	1.33	2.12	26	0	1	40	1	1
76.5	14.4	66.7	1.24	3.59	96.4	1	1	18.7	1	1
54	11.4	101	0.91	3.18	78.2	1	1	26.3	1	1
53	14	69.9	1.21	8.1	152.2	1	1	10.2	1	0
53	13.6	73.3	1.17	7.4	217.9	0	3	487.6	2	0
73	16.8	58	1.24	1.99	50.6	0	2	>1000.0	2	0
45.6	14.6	65.2	1.26	17.91	171	0	1	122.9	2	1
96	14.2	73	1.08	4.6	105.2	0	1	30.8	1	1
69.4	16.8	51.7	1.44	4.3	115	1	1	>1000.0	2	0
63	12.8	81.1	1.1	4.61	154.3	0	2	>50000.	2	0
86.7	15.3	60.3	1.32	4.8	42.4	0	3	>1000.0	2	1
55	14.6	70	1.1	2.25	62.5	1	1	283.9	1	1
48	16.4	60	1.21	2.33	42	0	2	17	1	1
62	12.5	87	0.98	14.28	211.6	0	3	4.7	2	0
68.8	16.9	57	1.24	4.34	77.2	0	1	158.9	2	0

175.2	14.1	69.1	1.21	12.93	312	0	3	1.8	2	1
41	18	53	1.31	7.2	113.5	1	1	>1000.0	2	0
77.6	12.2	87.8	1.05	9.36	77.6	0	2	38.9	1	1
81.6	17.2	49.7	1.48	3	38.3	1	2	30.2	1	1
96.5	15.7	57.8	1.35	2.95	46.4	1	1	>1000.0	1	0
52.1	14.8	63.7	1.27	8.33	243	1	1	>1000.0	2	0
93	13.6	73.3	1.17	6.15	189.4	1	1	117.5	2	0
77	14.9	63	1.28	5.64	68.2	1	1	>1000.0	2	0
75	15.3	60.3	1.32	3.2	71.3	0	2	5.4	2	0
70.7	17.1	56	1.25	5.61	120.5	0	3	44.5	2	0
74	11.3	102	0.9	4.27	146.3	1	1	23.6	1	1
69	14.6	65.2	1.26	8.09	150	0	2	20.5	1	0
51	15.5	64	1.16	2.16	34.1	0	3	39.1	2	1
51	11.4	101	0.91	5.08	84.7	1	2	64.6	1	1
54	15.2	66	1.14	7.63	294	0	2	>1000.0	1	0
76	14.5	71	1.1	7.28	161.7	0	3	>1000.0	1	0
39.2	17.9	46.5	1.54	2.76	62	1	1	651	2	0
108	15.3	60.3	1.32	6.02	146	1	2	8.8	2	0
190.1	20.4	37.4	1.75	3.74	28.4	0	1	>1000.0	2	1
625.1	12.8	81.1	1.1	4.73	269	0	3	3.2	1	0
64	11.1	105	0.89	7.57	193.8	0	3	3	2	1
44.8	17.6	47.8	1.51	5.92	100	0	1	>1000.0	2	1
60	22.2	40	1.54	2.69	37.3	0	3	16.8	1	1
69.2	16.5	53.2	1.42	3.98	50	0	1	321.6	2	1
76	11.4	101	0.91	3.75	206.8	0	2	2.9	1	1
132	14.8	63.7	1.27	5.04	96.2	0	1	>1000.0	2	1
303.9	17.6	47.8	1.51	2.47	52.4	1	1	652.4	2	0
705	13.3	80	1.02	2.25	42.2	1	1	795.3	2	1
61.4	14.6	65.2	1.26	3.81	58.4	1	1	87.5	1	0
53.7	22.7	39	1.57	9.51	37	1	1	3.7	2	1
59	14.3	72	1.09	4.9	117	1	1	>1000.0	2	0
61.8	15.5	59	1.33	2.34	63.1	1	2	>1000.0	2	1
448	14.6	64.8	1.26	32.88	323	0	1	>1000.0	2	0
274	13.9	70.7	1.2	5.2	115.3	0	3	999.3	2	1
81.2	20.3	45	1.44	3.56	26.1	0	2	1.8	2	1
45	21.2	36	1.82	3.66	46.4	0	3	15.5	2	1
69	10.7	111	0.86	4.33	90.3	0	1	21.7	1	0
62	11	107	0.88	4.81	102.5	1	1	6.1	1	0
68	13.3	76.1	1.15	8.09	194.5	0	1	27.7	2	0
81.8	14.1	68.5	1.21	3.48	88	0	3	650	1	0
50	14.7	69	1.11	1.57	47.2	1	1	20.7	1	1
61	15.8	57.2	1.36	2.72	78.3	0	3	3.5	2	1
930.1	15.6	58.4	1.34	5.63	113	0	2	65.3	2	0
108.5	59.3	7.1	5.03	24.12	305	0	3	>1000.0	2	0

645.7	13.1	76.9	1.13	6.31	329	0	3	1.7	1	0
67.5	9.4	139	0.78	6.86	258.5	0	2	4.4	1	0
71.4	12.5	82.8	1.08	5.59	149	0	1	2.9	2	1
72	10.7	111	0.86	4.34	104.1	0	2	77.1	1	1
62.6	16	56.1	1.38	4.24	70	0	2	74.4	1	1
93	14.6	70	1.1	6.73	79.4	0	2	48.8	2	1
109.9	18.6	44.3	1.6	8.66	71	1	1	>1000.0	2	0
55	17.6	54	1.28	3.95	98.8	0	2	>1000.0	2	0
67	13.7	76	1.05	4.11	51.9	1	1	7.3	2	1
53	14.8	68	1.22	2.59	60.5	0	3	5.2	1	1
50.2	18.6	44.3	1.6	7.34	31.4	0	2	7.7	2	1
56	24.3	36	1.66	5.15	58.4	1	1	615.2	2	1
123	17.6	48.3	1.51	10.12	231	0	3	>1000.0	2	0
36.5	17	51	1.46	4.65	61	0	1	>1000.0	1	1
70	10.7	113	0.94	4.97	139.7	0	3	108	2	0
65.3	15.7	57.8	1.35	6.32	63.7	1	1	34.6	2	0
57	17.9	52	1.42	5.62	75.5	1	1	49.5	2	0
50	12.5	87	1.07	4.65	78.2	0	2	118.8	2	0
74	13.4	79	1.13	3.91	107.1	0	1	19296	1	1
59	12.2	91	1.05	5.71	43.9	0	3	9068	1	1
56	14.5	70	1.2	3.71	80.3	1	1	>1000.0	2	0
75	13.7	76	1.15	2.13	33.7	0	2	2.2	2	1
93.6	18.7	43.9	1.6	12.69	105	0	3	>1000.0	1	0
68	17.5	53	1.52	8.39	61.6	0	2	5.4	1	1
64.9	12.8	84	1.09	6.47	148	0	3	3.4	2	1
62	11.6	99	1.01	4.24	108.8	0	2	3.1	1	0
62	16.4	54	1.41	5.53	55	0	1	323.2	1	1
71	13	53.5	1.42	6.53	103.9	1	2	8.7	1	1
97	11.2	104	0.98	2.94	81.7	0	2	>1000.0	2	1
61	15.9	56.7	1.37	4.09	88	0	1	6.6	2	1
64.8	15.5	59	1.33	3.75	131	0	2	>1000.0	2	0
118.8	13.8	75	1.16	4.85	97.9	0	3	2.9	1	1
90	11.1	84	0.97	3.4	82.5	0	2	3.2	1	0
54	13.3	80	1.12	4.6	79	0	2	8.1	2	1
96.4	12.9	78.8	1.11	5.46	142	0	1	6	1	0
115	12.3	71	1.06	5.35	95	0	2	>1000.0	2	1
53.7	14.5	65.5	1.25	5.86	85	0	3	7	1	0
73	12.9	66	1.1	3.55	103	0	3	105.5	1	1
79.6	13.4	63	1.13	4.57	56	0	1	13	2	1
160.6	14.1	68.5	1.21	4.54	47.4	0	2	7.4	1	1
42.7	17.2	55	1.38	2.76	27.4	1	1	6.4	2	1
69	16.5	47	1.33	5.11	62.2	1	1	119.7	2	0
42.7	19.8	37	1.54	2.66	46.4	0	1	503.3	1	0
67	9.1	118	0.83	4.83	71.9	0	2	6.7	1	0

56.9	13	77.8	1.12	2	74	0	3	4.9	1	1
71.1	11.3	97	0.97	2.67	102	0	2	170.8	2	1
64	11.3	81	0.99	6.58	112	0	3	10	1	1
46.4	13.5	73.3	1.16	3.68	258	0	1	25.7	2	0
42.5	14.9	62.8	1.28	4.64	74	0	3	10.7	2	0
48.1	9.9	102	0.89	6	227	0	3	13.9	1	0
45.4	14.9	54	1.23	2.66	98	0	3	17	2	0
55.5	13.6	72.5	1.17	1.51	30.4	0	2	>1000.0	2	0
84	14.1	68.5	1.21	5.21	87	0	3	972.2	2	1
99	11.5	79	1	7.04	96.1	0	3	55.8	2	1
91	13.1	76.9	1.13	3.95	184.1	1	1	>1000.0	2	1
1295.9	15.1	61.5	1.3	1.8	34.4	0	1	198.4	1	0
211	44.9	17	3.98	17.42	87	1	1	43.7	1	1
89	12.9	82	1.11	4.42	88.3	1	1	181	1	1
51	13	81	1.12	3.27	160.7	0	1	636	2	1
54	11.9	92	1.03	7.09	270.5	0	2	>1000.0	2	1
58	11.8	94	1.02	4.94	97.9	0	2	3.7	2	1
113	27	31	2.37	8.94	91	0	1	>1000.0	2	0
66	14.5	65.5	1.25	3.36	92.2	0	3	3.1	1	0
45.5	15.8	57.2	1.36	2.94	68	0	3	54.8	1	0
172.1	12.8	83	1.11	2.55	38.2	0	3	3.7	1	1
36.9	15	62.1	1.29	8.31	216	0	1	>1000.0	2	0
1068.3	12.9	78.8	1.11	6.95	144	0	2	586.9	1	0
59	12.1	90	1.04	6.36	94.5	0	1	>1000.0	2	1
71	15.4	59.6	1.32	5.98	55.7	0	1	4.4	2	0
112	14.8	67	1.28	4.16	64	0	3	74.5	1	1
65	12.6	81.7	1.09	3.93	30.6	0	3	14.6	2	1
64	12	91	1.04	5.47	51.5	1	1	285.4	1	1
81.7	20.4	38.3	1.75	4.39	68	0	1	44398	2	0
66.4	13.5	73.3	1.16	9.34	243	1	1	>1000.0	2	1
67	11.2	102	0.96	7.76	204.2	0	3	60.7	1	0
62	15.8	61	1.37	4.76	56.6	0	2	14636	2	1
71.7	16.2	59	1.41	3.85	50.5	0	1	4.8	2	1
52.4	14.8	67	1.28	7.15	65	0	3	9.8	1	1
55.5	16.5	53.5	1.42	3.44	43.2	0	1	9.3	2	1
77	11.4	99	0.98	6.67	161.8	0	2	3958	1	0
54	20.7	43	1.81	2.32	37.3	1	1	755	1	1
87	14.1	68.5	1.21	4.36	46.6	0	2	>50000.	2	1
73	10.4	115	0.89	5.2	105.2	0	1	43.2	2	1
97	12.7	80.7	1.09	4.44	106	0	1	38.7	2	0
63	16.9	56	1.47	11.4	104	1	1	1884	1	0
72.7	17	51	1.46	6.69	84	0	3	3.7	2	1
37.7	13.3	75.1	1.15	7.54	377.4	0	2	16.8	1	0
396.1	13.9	70	1.2	7.83	217	0	2	3.2	1	0

73	19.4	47	1.69	5.71	94.2	0	3	>2000.0	2	0
69	11.1	103	0.96	3.63	121.6	0	3	4.7	1	0
59	12.7	84	1.1	4.68	129.1	0	1	>2000.0	2	1
95.6	21.4	35.5	1.83	24.4	88	0	1	21	1	0
172.6	15.5	59	1.33	10.31	218	0	3	>2000.0	2	0
94.6	12.6	81.7	1.09	2.06	61	0	3	3.6	1	0
99	14.1	68.5	1.21	3.38	112	0	3	2.3	2	1
74	15.4	62	1.31	4.67	112.7	0	1	42.4	1	1
78	11.6	93.1	1	5.67	322.1	0	3	25.3	1	0
61	11	103	0.95	6.21	227	0	2	42.3	1	1
58	12.5	82.8	1.08	7.06	201.5	0	2	77.3	2	0
78	12.1	88	1.04	2.32	68.4	1	2	10.3	1	0
86.8	17.3	49.6	1.49	11.06	134	0	3	4.1	2	1
31	13.4	78	1.15	3.76	185.3	0	2	40765	2	0
60.5	15.7	57.8	1.35	4.5	36.4	0	1	296.8	2	1
37	15.4	65	1.32	10.26	168.4	0	3	1.6	2	0
83.5	17.1	50.6	1.47	5.88	64	0	2	1.9	2	1
72	13.2	76	1.14	6.92	121.3	0	1	>2000.0	2	1
68	16.7	52.5	1.43	4.46	66.2	1	1	88.5	2	0
59.3	13.5	73.3	1.16	5.8	124	0	1	>2000.0	2	0
51	12	92	1.03	5.94	143.7	0	2	2.5	1	1
73	11.9	93	1.03	3.07	132.2	0	3	44.4	2	1
53.4	12.9	73	1.19	6.16	147.9	0	1	>2000.0	2	0
88	13.8	70.8	1.19	4.54	52.2	0	3	1.6	1	1
71.6	11.1	99.8	0.96	7.6	207	0	3	1337	1	1
67.5	20.5	38	1.76	2.86	64	1	1	142.6	2	1
51	10.7	99	0.99	5.3	201.1	0	1	1196	1	0
53	13	72	1.2	2.88	56.5	0	3	78.4	2	1
47	14.7	64.1	1.26	4.21	37.2	0	3	7.1	1	1
68	12.6	85	1.08	6.25	170.2	0	2	5.4	2	0
51	12.3	79	1.14	4.68	82.7	1	2	102.4	1	0
193	13.8	70.8	1.19	9.26	188.9	0	3	>2000.0	2	0
56	12.8	79.7	1.1	2.82	92.3	0	2	1.3	1	0
79.4	12.6	81.7	1.09	2.88	84	0	2	2.5	1	1
76	19.6	40.8	1.68	10.24	67.4	0	3	>2000.0	2	1
53.5	15.7	57.8	1.35	2.57	44.4	1	1	1.8	1	1
60.5	13.3	75.1	1.15	8.31	128	1	1	7.8	1	0
55	13.5	73.3	1.16	3.13	68.3	0	3	1484	2	1
76.1	16.6	53	1.43	9.77	72	1	3	15.9	1	1
57.8	13.2	76	1.14	4.53	109	0	3	>2000.0	2	1
64	14.9	62.8	1.28	6.04	80	1	1	349.9	2	1
64.5	15	62.1	1.29	9.94	58.7	1	2	56	1	1
40	14.7	60	1.35	7.55	76.4	0	1	>80000.	1	0
63	11.5	88	1.06	3.13	53.9	0	2	195.1	2	1

61	12.9	73	1.19	3.62	119	0	1	3.7	1	0
86	12.8	74	1.18	8.61	155.8	0	3	>2000.0	2	0
46.3	13.6	72.5	1.17	2.37	48.3	0	1	53.6	1	1
78.3	16.5	53.5	1.42	20.46	164	0	2	73.1	2	0
70.5	14.6	64.8	1.26	5.06	219	0	3	1.9	2	1
45.7	14	69.3	1.21	2.83	86	0	1	37.7	2	0
52.3	14.9	62.8	1.28	4.37	116	1	1	8.4	2	0
71	14.3	63	1.32	9.32	267	1	1	>2000.0	2	0
82	16.6	51	1.52	10.94	94	1	1	3.2	2	0
75	13.3	75.1	1.15	2.99	75	1	1	13.8	1	1
45	16.4	54	1.41	5.06	68.4	0	3	2.2	1	1
85.2	13.8	66	1.27	3.19	85	0	2	1711	2	0
41.9	13	77.8	1.12	7.84	163	1	1	>2000.0	2	0
126	10.6	100	0.98	4.53	89.4	0	2	2.6	2	1
53	15.3	57	1.41	6.5	124.1	0	1	1639	2	1
77	16	56.1	1.38	2.27	39.1	0	3	5.1	2	1
64	12	88.3	1.03	4.55	145.3	0	2	4.1	1	1
73	11	95	1.02	2.84	144	0	2	1.3	2	1
50	19.1	42	1.75	5.62	77.3	0	1	299.4	2	0
86	12.9	73	1.19	6.69	142.7	0	2	>2000.0	1	0
55.7	13.1	76.9	1.13	3.18	67.4	0	2	2.6	1	1
77	13.8	70.8	1.19	1.56	6.4	0	2	1462	2	1
48.4	16.9	51.5	1.45	8.55	226	1	1	>2000.0	2	0
73	13.8	70.8	1.19	2.63	52.8	0	1	19943	2	1
58	16.7	50	1.53	3.6	139	1	1	>80000.	2	1
79.4	11	95	1.02	8.64	81	0	2	4.8	1	0
56.9	13.6	72.5	1.17	6.3	68	0	3	36	2	1
81	19.1	42	1.75	3.4	86.4	1	2	574.1	1	0
174	21.4	42	1.98	10.51	73	1	1	>2000.	1	0
80	21.1	37	1.93	6.39	43.4	0	1	5703	2	0
77	14.6	64.8	1.26	6.04	124	0	1	25.9	1	1
137.7	18.1	46.2	1.55	4.54	25.4	0	3	2	1	1
84	13.1	76	1.21	1.49	78	0	2	77	1	1
41.1	16.5	53.5	1.42	7.89	82	0	1	11	2	0
66.8	12.1	87.1	1.04	7.78	233	0	1	1335	1	0
96	13.8	71	1.27	3.67	45	0	3	2.2	1	0
137.9	25.8	26.5	2.21	20.09	155	0	3	29.5	2	1
74.7	17.8	47.5	1.53	7.92	108	1	1	>2000.0	2	0
66.8	15.1	61.5	1.3	2.86	50.4	1	1	>2000.0	1	0
64	12.8	84	1.11	4.38	75	0	2	10.9	1	1
65	14.4	68	1.33	3.85	53.3	0	1	210.1	1	1
61.1	13.9	70	1.2	18.1	181	0	2	125.1	2	1
76.9	11.2	98.4	0.97	4.76	110	1	1	1.3	2	1
61.7	19.9	39.8	1.71	6.3	56.4	1	2	6.2	1	1

58.6	11.9	89.4	1.03	6.09	185	1	1	9.8	2	0
68.2	17.2	50.1	1.48	3.86	46.4	0	2	5.8	2	1
55	11	97	1.02	4.07	161	0	3	4.4	1	0
95.3	15.3	60.2	1.32	5.2	78	0	1	>2000.0	2	1
71	18.3	49	1.69	1.87	48.4	0	2	2.9	1	1
69	10.9	99	1.01	11.61	202	1	1	35.7	1	0
52	17.9	50	1.66	6.9	66	1	1	1664	2	1
68	13.9	70	1.2	7.31	55	1	2	5	1	0
85.3	12.8	79.7	1.1	5.19	73.4	0	2	589.5	1	0
58.4	16.7	52.5	1.43	12.78	82	0	1	289.4	2	1
60.9	14.1	68.5	1.21	2.6	41.4	0	2	10.3	1	1
68	17.3	52	1.6	4.01	101.5	1	1	51.4	2	0
53.2	12.6	81.7	1.09	3.58	74	0	2	4	1	1
64.4	13.5	73.3	1.16	19.4	209	0	1	14.9	2	0
100	18	46.6	1.55	2.18	95	0	3	>2000.0	2	0
98	11	97	1.02	3.35	76.3	1	2	2.2	1	1
62	11.9	87	1.1	8.9	63	0	2	4.4	1	1
115.6	15.8	57.2	1.36	14.84	108.4	1	1	94.5	2	0
123	16.2	55	1.39	19.2	338	0	3	159.3	2	1
81	13.6	72.5	1.17	6.83	145	1	1	>80000.0	1	0
104	13.7	71	1.27	2	22	0	3	4.4	2	1
48	11.8	88	1.09	5.84	495.9	0	1	11.7	2	0
88.4	13.9	70	1.29	2.39	72	0	3	4.6	1	1
79	11.2	95	1.04	3.68	182.1	0	1	10.6	1	0
62	11.2	95	1.04	4.16	111.4	1	2	212.3	2	0
45.8	19.3	41.8	1.66	4.75	45.4	0	3	3.2	1	0
62	11.4	92	1.06	5.3	111	0	2	109.9	2	1
56	15.4	61	1.43	1.4	12	1	2	295.2	2	0
113	12.6	81.7	1.09	15.5	251	0	1	134.7	2	1
47.5	16	56.1	1.38	9.34	295	0	1	>2000.0	2	0
80	14.3	67	1.32	3.23	71.8	1	2	37.8	2	1
77	10.2	109	0.94	3.97	124	0	2	4.4	1	1
39	12.6	81.7	1.09	7.07	280	0	1	>2000.0	2	1
431.9	18.5	44.7	1.59	10.03	66	0	3	69	1	0
151	12.5	82.8	1.08	6.6	168	0	1	6.7	2	0
59	11.1	96	1.03	2.9	81	0	3	2.3	1	1
60.5	12.9	78.8	1.11	2.88	91	0	3	3.2	1	1
81	12	86	1.11	3.9	147	0	2	3	2	1
50	13.2	75	1.22	6.76	158.3	0	1	68.1	2	0
59	11.1	96	1.03	6.39	170.1	0	2	2.1	1	0
84	13.1	76.9	1.13	4.16	64	0	1	>2000.0	2	0
48	13.5	78	1.25	4.54	108	0	3	20.5	1	1
52.2	14.3	67	1.32	3.01	70	0	3	1.9	2	1
69	12.2	84	1.13	2.29	51	0	2	1.5	1	1

78	14.4	72	1.33	10.24	90	1	1	17.1	2	1
74.9	10.6	112	0.98	3.35	233	0	2	16.4	2	1
50	11.7	89	1.08	4.3	85	1	2	3.7	1	1
74	13.7	71	1.27	1.97	21	0	3	2.9	2	1
77	11.1	96	1.03	4.56	154.9	0	2	1.6	1	1
73	13.9	70	1.28	6.89	135.6	0	1	1153	1	0
130.2	18.8	50	1.74	7.87	73.4	0	3	>2000.0	1	0
263.3	13	83	1.2	1.31	40.4	0	3	393	1	1
56	11.8	95	1.09	2.83	47	0	1	27.3	2	0
73	14.4	72	1.33	7.21	138.9	0	2	4.1	1	1
107	15	63	1.38	1.37	36	0	3	255	1	1
87.5	14	74	1.3	10	152	0	2	1.1	1	0
50.5	20.2	44	1.86	4.31	37.4	0	3	>2000.0	2	0
161	13.9	70	1.28	9.11	366	0	3	1.3	1	0
51.3	22.1	40	2.05	12.77	175	0	1	448.7	2	1
59	14.6	66	1.35	4.46	52	0	1	1.3	1	1
42	13.6	78	1.26	7.39	179.2	1	1	5784	2	0
68	12.7	79	1.17	4.3	153	0	1	>2000.0	1	0
66	12.8	85	1.19	9.06	216.4	0	3	4	2	0
53	13.6	78	1.26	11.47	198.4	0	1	>2000.0	2	0
65.1	15.6	64	1.44	8.56	125	0	3	1.8	2	1
56.9	13.9	75	1.29	5.99	129	0	2	>2000.0	2	0
102.5	15.4	65	1.43	4.29	46.4	0	1	227.1	2	1
74.2	21.3	43	1.97	2.83	87	0	2	>2000.0	2	0
70	11	108	0.96	6.67	165.4	0	2	2.2	2	1
62.2	13.9	78	1.29	4.12	92	0	1	201.1	2	0
52	14	74	1.22	4.54	143	0	2	1.3	2	1
86.7	15.3	68	1.42	5.07	110	0	3	539.8	2	1
73	11.7	97	1.02	4.83	186	0	1	10.2	2	0
61	16.6	58	1.45	4.72	92	0	1	4.4	1	0
73	12.6	91	1.17	10.41	206	0	3	>2000.0	2	0
61.6	13.5	82	1.25	6.84	168	0	3	32.6	1	0
96.5	13.5	82	1.25	4.93	52	0	3	13.8	1	1
52.9	13.2	81	1.15	11.24	252	1	1	1629	2	0
73	11	113	1.02	5.27	148	0	2	1980	1	1
61	14	74	1.22	4.18	54	0	3	3.1	1	1
79	19.7	49	1.82	4.93	120	0	3	488	2	0
38.9	12.5	92	1.16	7.66	262	0	3	18.4	2	1
44.9	21.4	44	1.98	3.72	49.4	0	2	2.1	1	1
71.8	16.1	64	1.49	5.45	76	0	2	4.1	1	0
80.9	10.8	116	1	12.81	211	0	3	4.4	1	1
72	11.6	99	1.01	5.14	143.1	0	2	8.4	1	1
72	11.9	95	1.04	4.36	76.4	0	1	771	1	0
81.9	13.7	80	1.27	6.51	156	0	2	1.5	1	1

106	11.8	101	1.09	1.66	57	0	3	26.2	2	1
63.3	14.1	77	1.31	9.12	256	0	1	1.4	2	0
114	11.6	99	1.01	4.31	164	1	1	3.2	2	0
50.4	15.4	61	1.34	1.73	59	0	3	3.7	1	1
89.3	12.1	97	1.12	8.96	217	1	1	196.2	1	0
681	10.6	114	0.98	3.71	152.4	0	2	57337	1	0
67	16.9	53	1.48	3.1	63.8	0	2	0.8	1	1
67.4	14.7	67	1.36	4.75	163	0	1	31725	2	0
65	14.2	70	1.24	7.76	260.4	0	1	11	2	0
61.6	13.8	79	1.28	5.99	117	0	2	3	2	0
79	13.6	75	1.18	3.26	76.5	1	2	447.8	1	1
43.5	13.6	76	1.26	6.03	82.4	0	3	5771	1	0
77	18.8	48	1.74	1.77	62	1	1	>2000.0	2	1
69	10.5	119	0.91	6.77	134.5	0	3	2.3	1	0
83.8	12.1	91	1.05	2.94	136.4	1	1	24.5	1	1
85.5	13.6	76	1.26	4.44	152	0	1	409.9	2	1
79.4	13	81	1.2	9.21	166	0	2	2.1	2	0
63.2	10.3	121	0.95	10.43	216	0	3	66.1	1	0
46.5	15.1	63	1.32	2.82	67	0	2	790.5	1	1
68.7	15.2	63	1.33	2.59	43	1	2	6.7	1	0
72.1	15.1	63	1.32	2.78	41	1	2	6	1	1
76.4	14.6	68	1.35	9.43	234	0	1	119.3	2	0
52.2	16.8	59	1.56	1.61	54	0	3	15.2	2	1
91.8	12.9	83	1.12	3.32	158.2	0	2	2257	2	1
71.1	12.4	86	1.15	4.99	126	0	3	64.7	2	0
47.4	15.6	61	1.44	5.09	63.4	0	3	>2000.0	2	0
39.3	14.2	70	1.31	2.69	134.2	1	1	1076	1	1
891.2	15.7	61	1.45	12.04	93.6	0	1	>2000.0	2	1
51.8	12.8	84	1.11	3.75	98	0	2	33.9	2	1
60.8	12.9	83	1.12	4.34	35.4	1	2	1.1	2	1
519.6	17	54	1.57	12.08	114.4	0	1	54.1	2	1
54.4	15	71	1.3	9.25	257.3	1	1	>80000.	2	0
64.2	15.5	64	1.34	1.99	53	0	3	1.4	2	1
81.5	17.3	54	1.59	3.97	98	1	1	20.8	2	1
54.6	13.8	80	1.28	5.46	213	0	1	12758	2	0
73	12.3	90	1.07	5.39	168	0	1	>2000.0	2	1
115.1	17.4	57	1.61	8.57	160	0	3	4.8	2	1
61.4	13.3	80	1.15	3.11	57	1	2	594.8	1	0
73	17.9	51	1.65	3.6	101	0	1	58	2	0
71.7	16.1	63	1.49	8.29	140	1	1	>2000.0	2	1
91.8	12.9	78	1.19	17.54	270	0	2	1	2	0
67.6	15.9	63	1.48	10.56	304	0	1	3.3	1	0
68.1	14.7	69	1.37	1.78	47	0	2	8	1	0
95.7	15.7	65	1.45	5.13	28.7	0	3	2.6	2	0

79	17.2	58	1.59	4.1	114.9	0	2	39.9	2	1
130.9	13.6	77	1.18	4.51	238	0	2	1116	2	1
343.9	16	59	1.47	5.89	64	0	3	38.3	2	1
61.3	12.1	102	1.12	3.68	118	0	1	>2000.0	1	0
48	12.8	79	1.18	5.16	101.4	0	2	>2000.0	2	0
94.1	11.8	88	1.09	9.49	253	0	3	184.2	1	0
83.8	12.8	85	1.11	10.22	220	0	1	238.5	2	0
159.7	20.1	45	1.84	4.9	113.4	0	2	>2000.0	2	0
76.2	20.5	46	1.9	14.79	157.7	0	1	43178	2	1
161.9	12.6	87	1.09	6.63	231	0	1	>2000.0	1	0
205.1	15.7	61	1.45	14.44	337	1	1	>2000.0	2	1
62.3	16.8	62	1.56	5.31	208	0	1	5.27	2	0

stage1(0,	treamen
2	2
1	2
3	3
1	2
3	2
3	3
1	1
2	2
1	2
1	1
1	1
2	2
1	2
3	2
2	2
1	1
2	2
2	2
1	2
1	2
4	3
1	2
3	3
2	2
2	3
2	2
1	2
3	3
1	2
1	2
2	2
2	2
2	2
1	2
1	2
2	1
1	1
2	2
1	2
2	2
1	1
1	2
1	1

2	1
3	2
3	3
3	2
1	2
1	2
2	2
2	2
2	1
1	1
1	2
1	2
3	3
2	2
3	2
1	2
1	1
2	2
1	2
2	2
1	2
1	2
2	2
2	2
2	1
1	1
2	2
1	2
1	2
1	2
4	2
1	1
1	2
1	2
1	2
2	2
1	2
1	1
1	1
1	2
1	2
1	2
2	2
3	2

4	2
1	2
1	2
3	2
1	1
1	1
2	2
1	2
1	2
0	2
3	2
1	2
1	2
2	2
2	2
2	1
1	2
1	2
1	2
2	2
1	2
1	1
1	2
2	2
3	3
1	1
1	2
2	2
3	2
2	1
1	1
1	1
2	1
3	2
3	2
3	3
1	2
2	2
4	2
3	2
1	2
1	2
1	2
0	1

1	2
1	2
2	1
1	2
2	2
1	2
2	2
1	1
1	2
1	2
3	2
1	2
2	2
2	2
1	2
1	2
1	2
1	2
2	2
1	2
1	2
2	2
0	2
1	2
0	2
4	3
3	2
1	2
2	2
3	2
2	2
4	3
1	2
1	2
3	3
2	2
2	3
2	2
2	2
1	2
1	1
1	2
1	2
2	2

1	2
2	2
2	2
1	2
1	2
1	2
1	2
1	1
4	3
2	2
2	2
3	2
1	2
1	2
3	2
4	2
2	2
2	2
1	2
1	2
1	2
3	3
2	2
2	2
1	1
3	2
1	2
1	2
1	2
2	2
2	2
1	2
2	2
1	2
1	2
1	2
1	2
4	2
2	1
1	2
1	2
3	3
0	2
1	2

3	2
3	2
1	2
1	2
2	2
1	2
2	2
1	2
2	2
1	1
1	2
1	2
2	2
2	2
1	2
4	2
2	2
1	2
3	2
1	1
1	2
2	2
1	2
2	2
3	2
1	2
1	1
3	2
1	2
1	1
0	2
0	2
2	2
2	1
4	2
1	1
1	2
1	2
1	1
1	2
1	2
2	2
3	2
1	2

2	2
0	1
2	2
1	2
1	2
0	2
1	3
2	2
3	2
1	1
2	2
0	2
2	2
2	2
2	2
2	2
2	3
2	2
2	2
2	2
2	2
1	2
2	2
4	3
2	2
1	2
1	2
2	2
4	2
2	2
1	2
1	2
2	2
2	2
1	2
1	2
1	2
1	2
1	1
0	2
1	2
3	3
2	2
3	2

2	2
1	2
1	2
1	2
2	2
2	2
1	2
1	2
1	1
2	1
2	2
1	2
1	2
2	2
2	2
2	1
3	2
1	1
3	2
3	2
1	1
1	1
2	2
1	1
3	2
2	2
1	2
2	2
1	2
1	2
2	2
1	2
2	2
1	2
1	1
1	2
2	2
2	3
2	2
2	2
0	2
1	2
2	2
3	3

1	2
2	2
0	2
0	2
3	3
1	2
1	2
2	2
3	2
2	3
2	2
1	1
2	2
2	2
1	2
3	2
1	2
2	2
3	2
0	2
1	2
1	2
0	2
1	1
1	2
3	2
2	2
1	2
1	2
2	2
1	2
1	2
2	2
2	2
1	2
3	2
3	3
1	2
1	2
2	2
2	2
1	2
2	2
2	2

3	2
2	2
2	2
2	2
1	2
0	2
1	2
4	3
2	2
3	2
1	2
2	2
1	2
2	2
1	2
1	2
2	2
2	2
2	2
2	2
2	2
0	2
1	2
2	2
0	1
1	2
2	2
2	2
1	2
1	2
1	2
2	2
2	2
1	2
1	2
2	2
0	2
2	2
1	2
1	2
2	2
1	2
2	2
1	2

2	2
2	2
2	2
2	2
2	2
2	2
2	2
2	2
1	2
2	1
0	1
2	2
1	2
1	2
1	2
1	2
2	2
2	2
1	2
2	2
1	2
1	2
0	1
2	2
2	2
0	2
1	2
1	2
1	2
3	2
1	1
1	2
3	3
2	2
2	2
0	2
1	2
1	2
1	2
1	2
2	2
1	2
1	2
1	1

2	2
3	2
2	2
3	3
3	2
3	2
2	2
2	2
2	2
2	2
1	1
0	2
3	2
4	1
2	2
2	2
2	2
1	2
1	1
1	2
1	2
1	2
1	1
2	2
3	2
2	2
1	2
1	2
1	2
2	2
1	2
0	2
2	2
2	2
2	2
2	1
1	2
1	2
0	1
2	2
1	2
0	2
1	2
1	2

2	2
2	2
1	2
1	2
1	2
1	2
1	2
1	2
1	2
1	2
1	1
2	2
2	2
0	2
2	2
2	2
2	2
2	2
3	2
2	1
1	1
1	2
2	1
2	2
1	2
1	2
1	2
2	2
2	2
1	2
2	2
2	2
2	2
2	2
2	2
0	2
2	2
1	2
1	2
2	2
2	2
1	2
3	2
1	2

2	2
1	2
2	2
2	2
2	2
2	2
3	2
2	2
2	2
2	2
2	2
1	2
2	2
1	2
3	3
2	2
2	2
2	2
2	2
1	2
2	2
1	2
2	2
2	2
1	2
1	2
2	2
2	2
2	2
2	2
3	2
1	2
3	2
1	1
2	2
1	2
2	2
2	2
2	2
2	2
2	2
2	2
1	2
1	2

2	2
1	2
2	1
1	2
2	2
3	2
2	2
2	2
2	2
2	2
1	2
2	2
2	2
2	2
2	1
2	2
2	2
1	1
3	2
2	2
1	2
2	2
2	1
1	2
2	2
4	2
1	2
1	2
1	2
1	2
1	2
1	2
2	2
1	1
1	2
2	2
2	2
1	2
1	1
2	1
2	2
2	1
2	2
0	2

2	2
2	2
1	2
2	2
1	2
2	1
2	2
2	2
2	2
2	2
2	2
1	2
2	2
1	2
2	2
1	2
0	2
1	2
1	2
3	3
0	1
2	2
2	2
1	2
1	2
2	2
2	2
2	2
2	2
2	1
1	2
2	2
1	2
1	1
1	2
2	1
2	2
1	2
2	2
2	2
1	2
2	2
2	1
1	2

3	3
2	2
2	2
2	2
1	2
2	2
2	1
1	2
1	2
1	2
1	2
2	2
2	2
1	2
1	2
2	1
2	2
2	2
1	2
1	2
2	2
2	2
1	2
2	2
1	2
2	2
1	2
2	2
2	2
1	2
2	2
1	1
2	2
2	2
2	2
2	2
1	2
3	3
3	2
1	2
0	2
1	1
2	2
1	2

0	2
2	2
2	2
2	2
0	2
3	2
2	2
1	2
1	2
2	2
0	2
1	2
2	2
2	2
2	2
2	2
2	2
3	2
4	3
2	2
1	2
1	2
2	2
1	2
2	2
0	2
4	2
1	2
1	2
1	2
1	2
2	2
1	2
1	1
2	2
2	2
1	2
1	2
2	2
3	2
1	2
1	2
2	2
2	2

2	2
2	2
2	2
2	2
2	2
2	2
2	2
1	2
2	2
1	2
1	2
1	1
2	2
2	2
1	2
1	2
2	2
2	2
1	2
2	2
1	2
1	2
1	2
3	2
2	2
3	2
2	1
2	2
1	2
3	2
3	2
1	2
3	2
2	2
3	3
3	3
2	2
3	2
3	2
4	3
2	2
2	1
3	2
3	3

2	3
2	2
1	1
3	2
2	2
2	2
1	2
2	2
1	2
2	2
2	2
1	1
4	2
2	2
3	3
2	2
2	2
1	2
2	3
1	2
2	2
2	1
2	2
1	2
2	2
3	2
2	2
1	3
3	3
2	1
1	2
1	1
1	1
1	1
2	3
2	2
2	2
3	2
0	1
1	2
1	2
1	2
3	2
0	2

3	2
1	1
3	2
1	2
2	2
2	2
2	1
3	2
3	2
1	2
1	1
1	1
1	2
1	2
1	2
3	2
1	1
2	2
1	2
2	2
2	2
2	2
2	2
3	2
2	2
2	2
1	1
3	2
4	2
2	2
1	2
1	1
1	2
2	1
2	2
3	2
1	2
1	2
1	2
2	2
0	1
1	2
2	2
1	1

2	2
1	2
2	1
1	2
2	2
1	1
0	2
1	2
1	2
1	1
3	3
3	3
1	2
3	2
2	2
1	1
1	2
1	1
1	2
1	1
2	2
1	2
1	1
1	1
2	2
2	2
1	2
1	1
1	2
1	2
1	2
1	2
2	2
2	2
1	2
1	1
1	2
2	1
1	1
1	1
1	2
2	2
2	2
1	2

2	2
4	2
2	2
1	2
1	2
1	2
4	2
4	2
4	2
4	2
1	1
3	3
4	3
4	2
4	2
4	2
3	3
4	3
4	2
1	2
3	2
2	3
1	2
1	2
2	3
2	2
2	2
3	3
1	2
1	2
2	2
3	3
2	2
2	2
3	2
2	2
3	2
3	2
1	2
2	2
3	3
2	2
3	3
4	3

3	2
2	2
1	2
2	2
3	2
3	3
2	2
2	2
3	2
3	2
3	3
3	2
3	2
3	3
3	2
3	2
1	2
2	2
1	2
3	3
4	3
3	2
4	3
3	2
2	2
3	3
4	3
4	3
4	3
4	3
4	3
3	3
4	3
3	2
1	2
4	3
1	2
3	3
1	3
3	3
4	3
3	3
4	2
3	3

4	3
2	2
3	3
2	2
4	3
1	2
4	3
1	3
3	2
4	3
4	3
4	2
2	3
4	3
4	3
4	2
4	3
3	3
1	1
4	3
2	2
4	3
3	3
1	1
4	3
2	2
3	2
4	3
4	3
4	3
3	2
4	3
3	3
2	1
2	3
2	2
3	3
3	2
3	3
4	3
3	3
4	2
4	3
4	3

3	2
3	2
3	2
4	3
4	3
4	3
2	3
4	3
4	2
2	2
4	2
4	3
4	3
4	3
2	2
4	3
4	2
4	3
4	3
3	2
2	1
2	2
4	3
1	2
4	2
4	3
3	3
2	2
4	3
4	3
1	2
4	3
4	3
2	1
4	3
3	3
2	3
2	2
3	3
3	2
4	2
3	3
4	3
2	2

4	3
1	2
2	2
1	1
4	3
3	2
3	3
4	2
4	3
3	3
3	3
3	2
2	2
3	3
3	2
4	3
4	2
4	3
4	3
3	2
2	2
3	2
4	3
3	2
3	2
3	2
3	3
3	2
2	3
3	2
1	1
2	2
4	3
4	3
4	3
3	2
2	2
3	3
4	3
4	3
3	3
1	2
4	3
3	3

2	2
4	1
4	3
4	3
4	3
3	2
1	1
1	1
4	3
4	3
3	2
1	2
4	3
2	2
4	3
4	3
4	3
4	3
4	3
3	2
4	2
4	3
2	2
1	2
3	3
3	2
3	3
2	2
1	2
4	3
1	2
2	3
3	2
4	3
4	3
1	2
3	3
2	2
3	2
4	2
4	3
2	2
3	3
1	2

4	3
3	3
2	2
1	2
4	3
1	1
1	3
4	3
4	3
2	2
4	3
3	3
1	2
4	3
4	2
2	2
4	3
2	3
4	3
2	3
3	3
4	2
3	3
3	2
1	2
1	2
4	2
4	3
3	2
2	2
1	3
1	2
3	3
3	3
4	3
4	3
1	2
4	3
3	2
4	3
1	2
1	2
3	3
4	2

4	3
4	3
1	2
4	2
2	3
3	3
3	3
4	2
3	2
4	3
2	1
2	2
4	2
1	2
2	3
3	2
4	3
3	3
4	3
2	3
2	3
4	3
4	3
2	2
1	2
3	3
4	3
2	2
3	2
4	3
3	3
3	3
4	3
2	2
4	2
2	2
1	2
2	2
2	2
3	2
1	2
4	2
2	3
4	3

2	3
2	3
2	2
2	1
1	2
3	3
4	3
3	2
2	2
1	2
4	2
4	3
4	3
1	2
2	2
4	3
4	2
2	2
2	1
3	2
3	2
2	2
4	3
4	3
4	3
1	2
1	2
1	2
3	2
2	2
4	3
4	3
2	2
2	2
2	3
3	3
1	2
1	2
3	2
4	3
3	2
4	2
4	2
3	2

2	2
2	2
0	2
3	2
4	3
2	3
2	2
3	2
2	2
2	2
3	3
3	3
4	3
2	1
3	3
2	1
2	2
4	3
1	2
2	2
1	2
3	3
1	2
3	3
3	2
1	2
2	2
1	1
4	3
4	3
2	3
4	3
2	3
1	2
4	3
2	2
4	2
4	3
2	2
3	3
3	2
4	2
2	2
3	3

4	3
2	2
3	3
4	3
4	3
1	2
3	3
1	2
1	2
1	1
3	3
1	2
4	3
3	3
4	3
2	3
4	3
3	3
4	3
3	3
1	2
2	2
3	3
1	2
2	2
4	3
3	2
2	2
1	2
4	2
3	2
4	3
2	2
1	2
4	3
1	2
2	2
3	2
4	2
2	3
3	3
2	1
3	3
2	1

1	3
3	3
4	2
4	3
2	2
2	2
3	3
3	2
4	3
1	1
4	3
4	3
3	3
2	2
3	2
2	2
1	2
2	2
4	3
3	2
1	2
3	2
3	3
3	2
4	3
2	1
2	2
4	3
4	3
4	2
4	2
4	2
1	2
3	3
3	3
2	2
4	3
4	3
1	3
1	2
2	1
4	3
2	2
4	3

2	2
4	3
1	2
4	3
2	2
1	2
4	3
2	2
3	3
2	2
1	2
4	2
1	2
4	3
4	3
1	1
1	2
3	3
3	3
3	2
2	2
3	2
2	3
2	2
3	2
4	3
3	2
2	2
2	3
4	2
2	1
1	2
3	3
4	3
3	2
1	2
1	2
2	2
4	3
2	2
4	3
1	2
3	2
2	2

3	3
2	2
1	2
2	2
1	2
3	2
4	3
1	3
3	3
1	1
1	2
1	2
4	3
2	3
4	3
1	2
3	2
3	2
3	3
4	3
3	3
3	3
4	3
4	3
2	2
3	3
3	3
2	3
2	2
4	3
2	3
3	3
1	2
4	2
1	2
1	2
4	3
3	2
4	2
1	2
1	2
1	2
3	3
2	2

3	2
3	2
2	3
1	2
3	2
2	2
1	3
3	2
3	3
3	3
1	2
2	2
4	2
2	1
1	2
4	3
2	2
2	3
1	3
2	3
2	2
3	3
2	3
3	2
2	2
4	3
2	3
4	3
3	2
4	3
4	3
3	3
3	3
4	3
3	2
3	3
4	3
2	2
4	3
3	3
4	3
3	2
3	2
3	3

4	3
3	3
4	3
3	2
3	3
3	3
3	2
4	3
4	3
3	3
4	3
4	3
