# Supplementary material for: Neutrophil-to-high-density-lipoprotein-cholesterol ratio and mortality among patients with hepatocellular carcinoma
Source: Front Nutr. 2023 May 5;10:1127913. doi: 10.3389/fnut.2023.1127913 (PMC10198653; doi:10.3389/fnut.2023.1127913)
Supplement: Supplementary file 2 [file Data_Sheet_2.PDF]

Relevant code/script

```
library(ggpubr) #
setwd("Desktop/R work")
dev<-read.csv("dev.csv")
dev<-na.omit(dev)
View(dev)
plots <- ggscatterhist(dev,
                       x = "MELD", y = "NHR",
                       color = "#004CFFFF",
                       margin.params = list(fill = "#004CFFFF")) #
plots$sp <- plots$sp +
  geom_hline(yintercept =3.5, linetype = "dashed", color = "red") +
  geom_vline(xintercept = 9, linetype = "dashed", color = "red")
plots
```

```
library(foreign)
library(survival)
library(rms)
setwd("Desktop/R work")

dev<-read.csv("dev.csv")
dev <- na.omit(dev)
names(dev)
attach(dev)
dev<-data.frame(NHR,time,status)

dd <- datadist(dev)
options(datadist='dd')

fit<- cph(Surv(time,status) ~ rcs(NHR,4),data=dev)
an<-anova(fit)
plot(Predict(fit, NHR,fun=exp), anova=an, pval=T)

HR<-Predict(fit, NHR,fun=exp,ref.zero = TRUE)
ggplot(HR)
```

```
library("survival")
library("survminer")
library(foreign)
setwd("Desktop/R work")
```

```
dev<-read.csv("dev.csv")
dev<- na.omit(dev)
names(dev)

fit <- survfit(Surv(time,status) ~Group,
                data = dev) #  拟合方程
summary(fit)
ggsurvplot(fit, data = dev)
```
